# Asymmetric Dirhodium-Catalyzed Modification of Immunomodulatory Imide Drugs and Their Biological Assessment

**DOI:** 10.1021/acsmedchemlett.4c00297

**Published:** 2024-08-23

**Authors:** William F. Tracy, Geraint H. M. Davies, Lei Jia, Ethan D. Evans, Zhenghang Sun, Jennifer Buenviaje, Gody Khambatta, Shan Yu, Lihong Shi, Veerabahu Shanmugasundaram, Jesus Moreno, Emily C. Cherney, Huw M. L. Davies

**Affiliations:** # Department of Chemistry, Emory University, Atlanta, Georgia 30322, United States; ∇ Small Molecule Drug Discovery, Bristol Myers Squibb, Cambridge, Massachusetts 02143, United States; § Small Molecule Drug Discovery, Bristol Myers Squibb, San Diego, California 92121, United States; ∥ Small Molecule Drug Discovery, Bristol Myers Squibb, Redwood City, California 94063, United States; ⊥ Small Molecule Drug Discovery, Bristol Myers Squibb, Princeton, New Jersey 08543, United States

**Keywords:** Cereblon, targeted protein degradation, molecular glue degraders, neosubstrate selectivity, cyclopropanation, stereoretention, enantioselective, immunomodulatory imide drugs

## Abstract

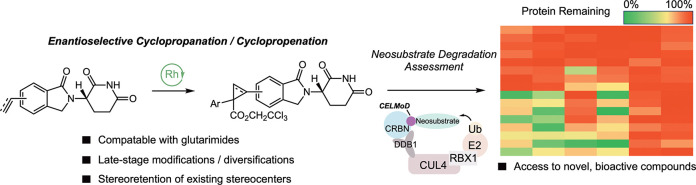

Cereblon (CRBN) has been successfully co-opted to affect the targeted degradation of “undruggable” proteins with immunomodulatory imide drugs (IMiDs). IMiDs act as molecule glues that facilitate ternary complex formation between CRBN and a target protein, leading to ubiquitination and proteasomal degradation. Subtle structural modifications often cause profound and sometimes unpredictable changes in the degradation selectivity. Herein, we successfully utilize enantioselective cyclopropanation and cyclopropenation on intact glutarimides to enable the preparation of stereochemically and regiochemically matched molecular pairs for structure–activity relationship (SAR) analysis across several classical CRBN neosubstrates. The resulting glutarimide analogs were found to reside in unique chemical space when compared to other IMiDs in the public domain. SAR studies revealed that, in addition to the more precedented impacts of regiochemistry, stereochemical modifications far from the glutarimide can lead to divergent neosubstrate selectivity. These findings emphasize the importance of enabling enantioselective methods for glutarimide-containing compounds to tune the degradation selectivity.

Immunomodulatory imide drugs (IMiDs) are clinically proven cancer medicines and are among the most well-characterized targeted protein degraders. Over the past several decades, the field has moved from phenotypic observations,^1^ to target elucidation,^2^ to solving ternary complex structures of cereblon (CRBN) with multiple neosubstrates.^3^ Despite these noteworthy advances, many challenges remain on the path to achieving a rational, structure-based design of IMiDs to selectively degrade a singular neosubstrate of choice. Prospective design of IMiDs to increase degradation potency or selectivity remains semiempirical, even against structurally enabled G-loop containing neosubstrates. More systematic neosubstrate selectivity correlations have recently been established.^3c,4^ However, reports of systematic structure–activity relationship (SAR) correlations for distal modifications of IMiDs across a range of neosubstrates are comparatively rare. This, coupled with the limited predictive power of structure-based models leaves much to be understood.^5^ Underlying these uncertainties is the capability of IMiDs to degrade previously undruggable proteins implicated in disease.^6^ This promise has driven the field forward exponentially, and chemistry has satisfied this demand by meeting the synthetic challenges unique to the glutarimide pharmacophore.^7,8^ The glutarimide itself is susceptible to hydrolytic or nucleophilic opening (Figure 1a).^9^ When the glutarimide is attached to an electron-deficient substituent typical of IMiDs, the alpha-stereocenter is vulnerable to epimerization.^10^ Achiral dihydrouracil ligands can circumvent this issue.^11^ As a result of these liabilities, the glutarimide moiety is often installed or unmasked late in a synthesis. One common approach is to install l-glutamine *tert*-butyl esters which can be deprotected and cyclized late in the synthesis.^12^ However, the acidic cyclization can be difficult to affect without eroding alpha-position stereochemistry (Figure 1b).^13^ This can also be true for protecting group strategies, such as PMB protection of glutarimide N–H, which can require strongly acidic conditions for deprotection. Another approach is to install a 2,6-dibenzyloxypyridine, which can be subjected to hydrogenation to unmask the glutarimide.^14^ The hydrogenation often requires forcing conditions to achieve the final reduction of the intermediate dihydroxypyridine, and this method does not provide stereocontrol. Glutarimides often have poor solubility, which can limit compatibility with certain methods.^7a^ As a result, there has been interest in the field to (1) demonstrate glutarimide compatibility with known methods, (2) reoptimize protocols for established synthetic methods to achieve compatibility, and (3) develop new methods that are glutarimide-compatible.

**Figure 1:**
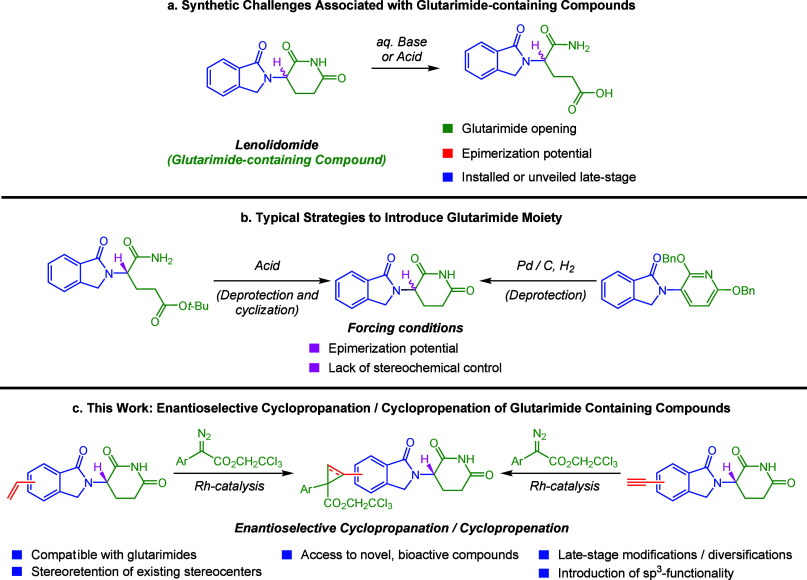
Navigating the synthetic challenges of introducing glutarimides and functionalizing glutarimide-containing compounds via enantioselective cyclopropanation/cyclopropenation.

We were particularly interested in the late-stage enantioselective cyclopropanation of glutarimides because few methods exist for the late-stage enantioselective installation of sp^3^-dense functionality onto glutarimide derivatives. We previously developed an anhydrous, stereoretentive Suzuki–Miyaura coupling to prepare vinyl glutarimide derivatives in high enantiopurity.^15^ Herein, we show that these vinyl-substituted glutarimides can be successfully employed in enantioselective cyclopropanation reactions while achieving high levels of stereoretention. Our method enables the preparation of sets of stereoisomeric glutarimide analogs with complete stereocontrol. Elaborated diazo precursors enable highly convergent syntheses, and the resulting esters can also be cleaved and subsequently derivatized. We then compared the resulting glutarimide derivatives with glutarimides reported in the public domain and found that our method offers access to glutarimides that diverge significantly in terms of chemical space (UMAP) and moment of inertia (i.e., overall shape: rod/sphere/flat). Finally, the compounds were profiled for degradation potency and selectivity across several G-loop-containing neosubstrates, including IKZF3 (Aiolos), CK1α, GSPT1, and SALL4. While the impact of small substituents directly off the phthalimide or isoindolinone core of glutarimides on degradation selectivity has been reported previously (*vide supra*), this is to our knowledge the first report investigating the impact of systematic distal changes in stereochemistry on degradation selectivity.

The rhodium-catalyzed enantioselective cyclopropanation and cyclopropenation of donor/acceptor carbenes were selected as the key reactions for the late-stage derivatization of the glutarimide derivatives. Cyclopropanes are important motifs in medicinally relevant compounds and introduce potentially valuable sp^3^-rich elements.^16^ In the context of CRBN molecular glue degraders, increasing sp^3^ character has been identified as a general strategy to avoid off-target degradation.^17^ These reactions are conducted under mild conditions and can result in very high levels of asymmetric induction. Initially, it was unclear whether glutarimides would have acceptable solubility in the nonpolar solvents typical of dirhodium-catalyzed [2+1] cycloaddition chemistry.

The first stage of the project was to determine whether enantioselective cyclopropanation could be conducted effectively on vinyl thalidomide and isoindolinone-based derivatives agnostic to the positional attachment of the vinyl group. Test reactions were conducted using 2,2,2-trichloroethyl 2-(4-bromophenyl)-2-diazoacetate, which is a robust carbene precursor amenable to high asymmetric induction with potential for further derivatization of its ester and aryl bromide functionality.^18^ The starting glutarimide is a racemate, and in these cyclopropanations two new stereogenic centers are generated. Thus, in the reported results, two diastereomeric ratios are included (Scheme 1a). One is for the relative configuration of the two stereogenic centers formed during the cyclopropanation (in red), and the second is for the level of asymmetric induction achieved by the chiral catalyst (in green). A catalyst screen showed (Scheme S1) that Rh_2_(*p*-PhTPCP)_4_, a recently developed catalyst, is most effective for these reactions.^19^ The “**a**” series are products derived from Rh_2_(*S*-*p*-PhTPCP)_4_-catalyzed reactions and the “**b**” series are products derived from Rh_2_(*R*-*p*-PhTPCP)_4_-catalyzed reactions. The absolute stereochemistry of the cyclopropane for **16a** was determined by X-ray crystallography (see the Supporting Information (SI) for more information). In general, the reactions proceed in good yield, except when the vinyl group is adjacent to the carbonyl group (**14a,b** and **18a,b**). Presumably, steric interference is responsible. All of the reactions proceeded with high levels of diastereoselectivity and asymmetric induction, further underscoring the performance of Rh_2_(*p*-PhTPCP)_4_ as a chiral catalyst for donor/acceptor carbene reactions.^20^

**Scheme 1:**
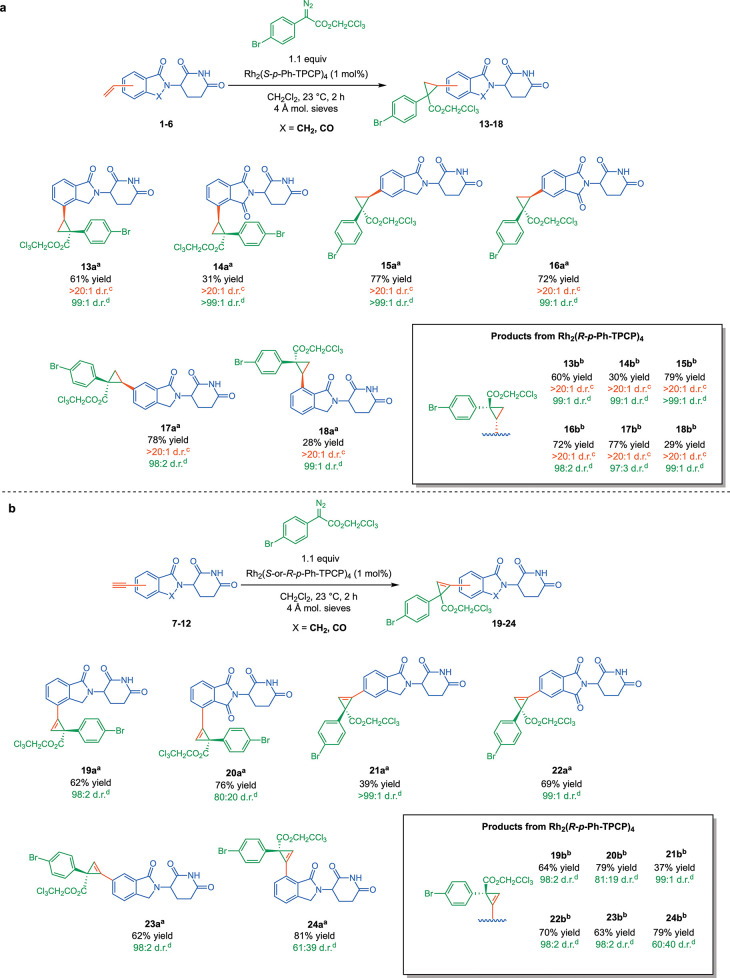
Cyclopropanation and Cyclopropenation of Vinyl and Ethynyl IMiD Derivatives Product arising from reaction with Rh_2_(*S*-p-Ph-TPCP)_4_. Product arising from reaction with Rh_2_(*R*-p-Ph-TPCP)_4_. Diastereomeric ratio for the relative configuration of the two new stereogenic centers, determined by ^1^H NMR analysis. Asymmetric induction arising from formation of the major relative diastereomer, determined by SFC analysis. All reactions were performed on racemic glutarimide intermediates. Yields are reported as isolated yields of purified product.

The next series of experiments focused on the asymmetric cyclopropenation reaction. All reactions proceeded in good yield, irrespective of the alkyne position. High levels of asymmetric induction were obtained (up to >99:1 d.r.), except in the cases in which the alkyne was positioned adjacent to the carbonyl, as seen with **20a,b** and **24a,b** which are produced with a poor diastereomeric ratios. Since analogous products from olefins (**14a,b** and **18a,b**) are produced with good diastereoselectivity, and cyclopropenation without the proximal carbonyl are as well (**19a,b**), we hypothesize that this effect may be due to an “end-on” approach of the substrate to the carbene which places the site of reaction closer to the adjacent carbonyl in cyclopropenation than cyclopropanation.^21^

The rhodium-catalyzed carbene reactions are conducted under very mild conditions, and we wondered whether the labile glutarimide stereocenter could be retained. The reaction with enantiomerically pure glutarimides, prepared using our previously published Suzuki–Miyaura reaction,^15^ are shown in Scheme 2. As shown in supercritical fluid chromatography (SFC) traces of the purified products (Scheme S2), no epimerization occurs under the reaction conditions, and all four stereoisomers are cleanly formed.

**Scheme 2:**
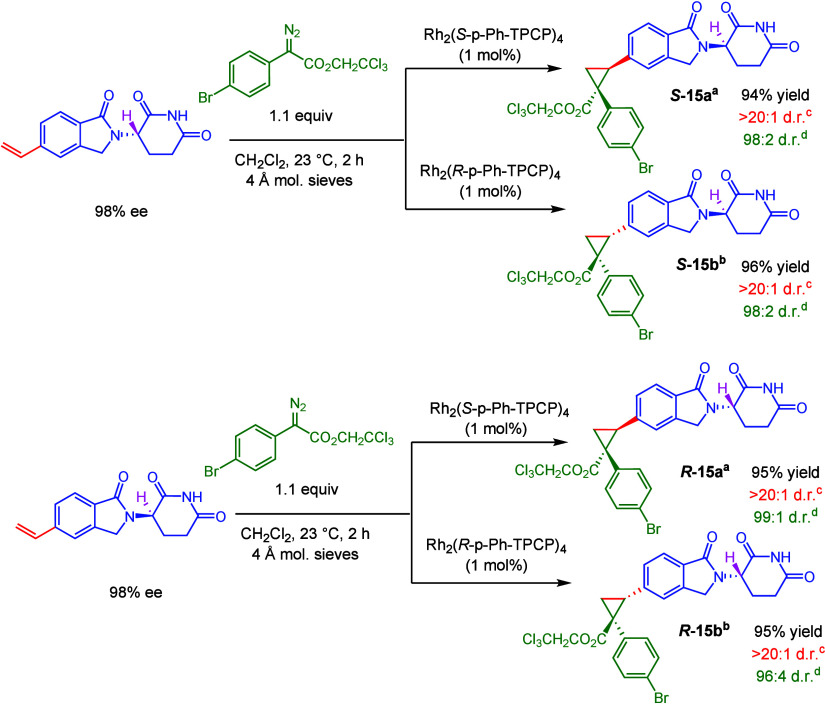
Stereoretentive Cyclopropanation of (*S*)- and (*R*)-3-(1-oxo-5-vinylisoindolin-2-yl)piperidine-2,6-dione Product arising from reaction with Rh_2_(*S*-p-Ph-TPCP)_4_. Product arising from reaction with Rh_2_(*R*-p-Ph-TPCP)_4_. Diastereomeric ratio for the relative configuration of the two new stereogenic centers, determined by ^1^H NMR analysis. Asymmetric induction arising from formation of the major relative diastereomer, determined by SFC analysis. Stereochemical information from the starting material was retained in all cases, as determined by ^1^H NMR and SFC. Yields are reported as isolated yields of purified product.

Having established the asymmetric cyclopropanation and cyclopropenation of IMiD derivatives, we began exploring the synthetic utility of the products (Scheme 3a). We were interested in introducing drug-like moieties, including those with nucleophilic sites that could react with the carbene. We recently demonstrated that using hexafluoroisopropanol (HFIP) as a solvent helps mask these nucleophilic sites and renders them compatible.^22^ Employing this tactic, we prepared a trichloroethyl diazoacetate with a benzyl morpholine element reminiscent of that present in Iberdomide (CC-220).^23^ When this diazoacetate was used under the standard conditions (Scheme 1a), no reaction is observed. However, using Rh_2_(*S*-*tetra*-*p*-Br-PPTTL)_4_ in conjunction with 10 equiv of HFIP, the cyclopropanation of the 4-vinyl isoindolinone core (**25a,b**) proceeds with modest asymmetric induction and good yield. Alternatively, the trichloroethyl ester can be leveraged via the conversion to the acid. Standard reductive conditions (zinc dust in acetic acid) led to low conversion and degradation. Fortunately, the acid forms readily via treatment with zinc dust in a solution of tetrahydrofuran and acetate buffer (**26a,b**). The acid can then be readily converted to the amide (**27a,b**) with complete retention of the stereochemical information introduced by cyclopropanation, highlighting another way to generate complexity.

**Scheme 3:**
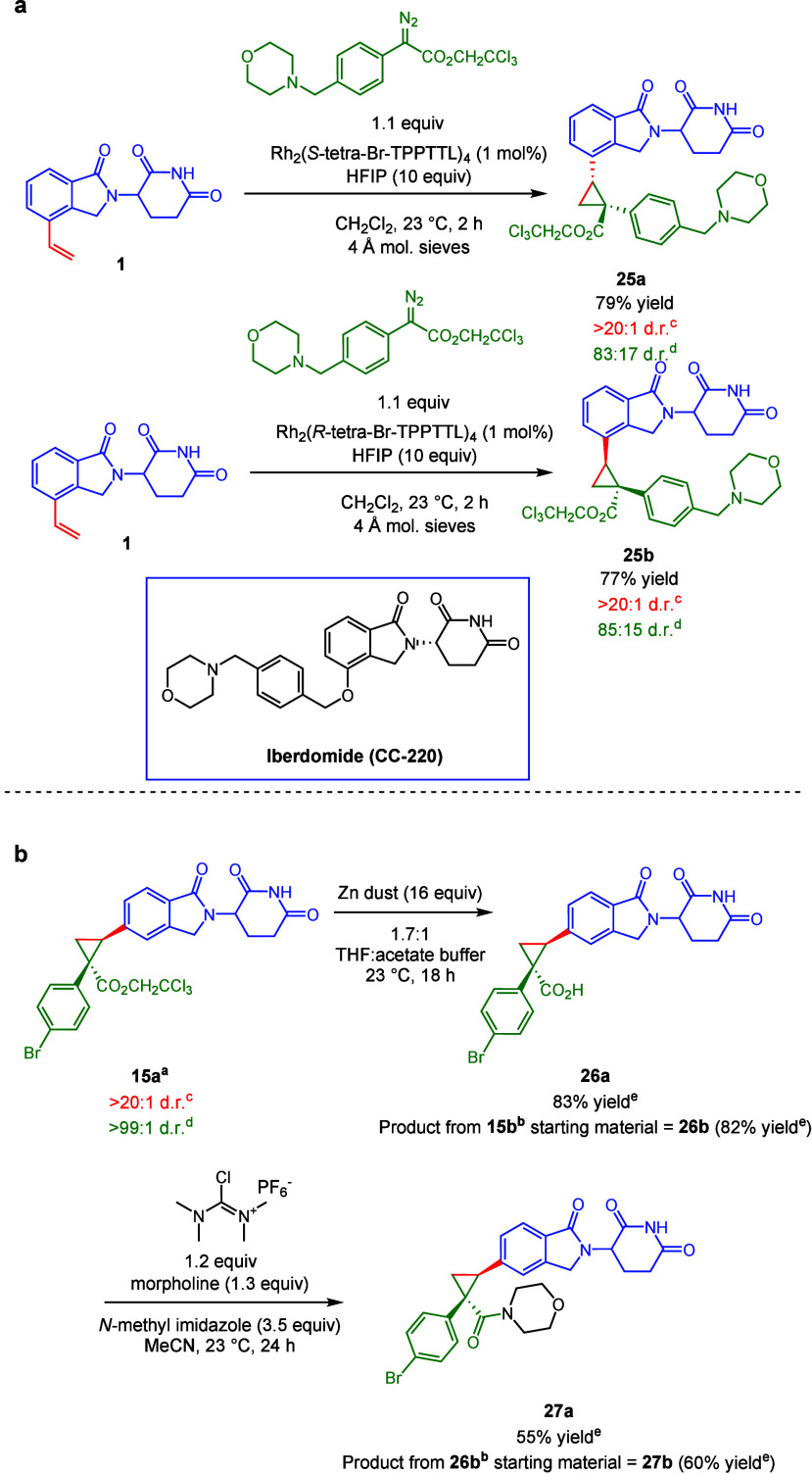
Introduction of Further Complexity Product arising from reaction with Rh_2_(*S*-p-Ph-TPCP)_4_ Product arising from reaction with Rh_2_(*R*-p-Ph-TPCP)_4_ Diastereomeric ratio for the relative configuration of the two new stereogenic centers, determined by ^1^H NMR analysis d Asymmetric induction arising from formation of the major relative diastereomer, determined by SFC analysis Stereochemical information from the starting material was retained, as determined by ^1^H NMR and SFC analysis. 1.2 M acetate buffer: calcd pH = 3.7. All reactions were performed on racemic glutarimide intermediates. Yields are reported as isolated yields of purified product.

Having demonstrated that this method provides access to novel IMiD structures, we were interested in assessing how these structures compare with IMiDs in the public domain. Figures 2a and 2b are two visualizations that benchmark our IMiDs versus a collection of 18,175 external IMiDs. Using Uniform Manifold Approximation and Projection (UMAP) (Figure 2a), a two component UMAP projection was constructed by embedding 2048 bit ECFP4 fingerprints.^24a^ As anticipated, our IMiDs cluster relative to one another due to their similarity. However, taken as a whole, our compounds venture into pockets of chemical space with minimal overlap versus what has been previously reported. Due to the potential value in adding sp^3^-rich elements using this method, we wanted to confirm that these compounds are occupying additional 3-D space. Using well-known molecular descriptors to characterize 3-D shape, the principal moments of intertia (PMI) plot in Figure 2b describes the extent to which our compounds are either more rod-shaped (upper left), disk-shaped (bottom), sphere-shaped (upper right), or any combination of the three.^24b^ Relative to the external collection, which is highly localized in the rod-like space, our compounds shift toward a more spherical shape. Perhaps more importantly, our collection encompasses a broad distribution of shapes, suggesting diversity despite such a small sample size. Based on the accepted notion that scaffold diversity increases the likelihood of targeting a broader range of biological targets, it is important to leverage synthetic methods that facilitate the generation of differentiated chemical matter. This is especially true in the targeted protein degradation space, where the discovery of novel degraders relies heavily on library synthesis.

**Figure 2:**
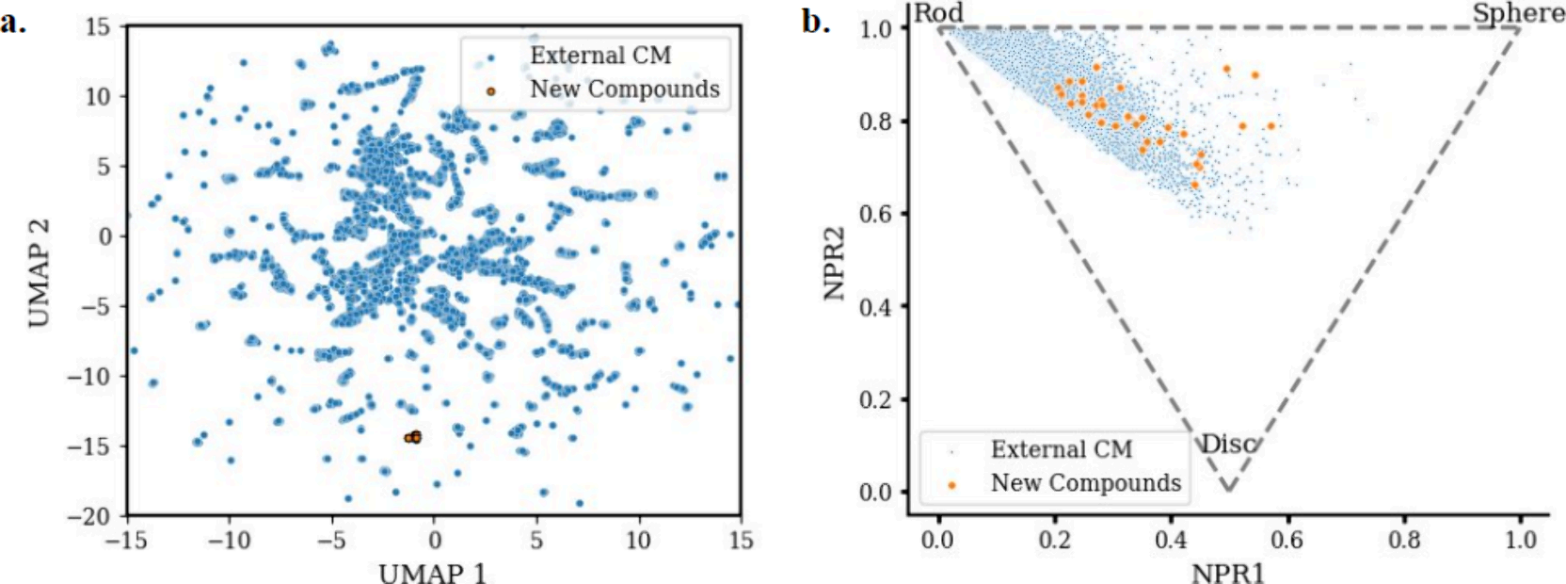
Representation of chemical and structural space accessed by the novel IMiDs relative to literature precedence (18,175 compounds). (a) Two-dimensional UMAP projection from 2048 bit ECFP4 fingerprints. (b) Principal moments of inertia analysis. Both plots depict the new IMiDs shown in orange relative to existing compounds shown in blue.

To assess SAR trends for CBRN binding and neosubstrate selectivity across several G-loop containing neosubstrates (IKZF3, CK1α, GSPT1, and SALL4), compounds **13a,b** to **24a,b** were profiled. Figure 3a correlates CRBN binding (HTRF IC_50_) to neosubstrate degradation (*Y*_min_ indicates depth of degradation with 100% representing no reduction in protein level and 0% representing complete degradation). Figure 3b reports trends in neosubstrate activity with EC_50_ (concentration required to achieve 50% of total degradation effect) reported and boxes colored by *Y*_min_ (with red showing very shallow or weak depth of degradation and green showing deeper or stronger depth of degradation). These matched molecular pairs allowed for the assessment of trends based on (1) core (isoindolinone “Len” vs phthalimide “Thal”, (2) regiochemistry of substitution off the core, (3) cyclopropane vs cyclopropene, and (4) distal stereochemistry. Largely, these compounds maintained measurable binding to CRBN, with only a small fraction (4 of 24 compounds) showing CRBN IC_50_s > 10 μM. There was no correlation between CRBN binding potency and degradation across neosubstrates (Figure 3a). This lack of correlation reflects the importance of forming a productive ternary complex between CRBN and a neosubstrate vs simply binding to CRBN.^5^

**Figure 3:**
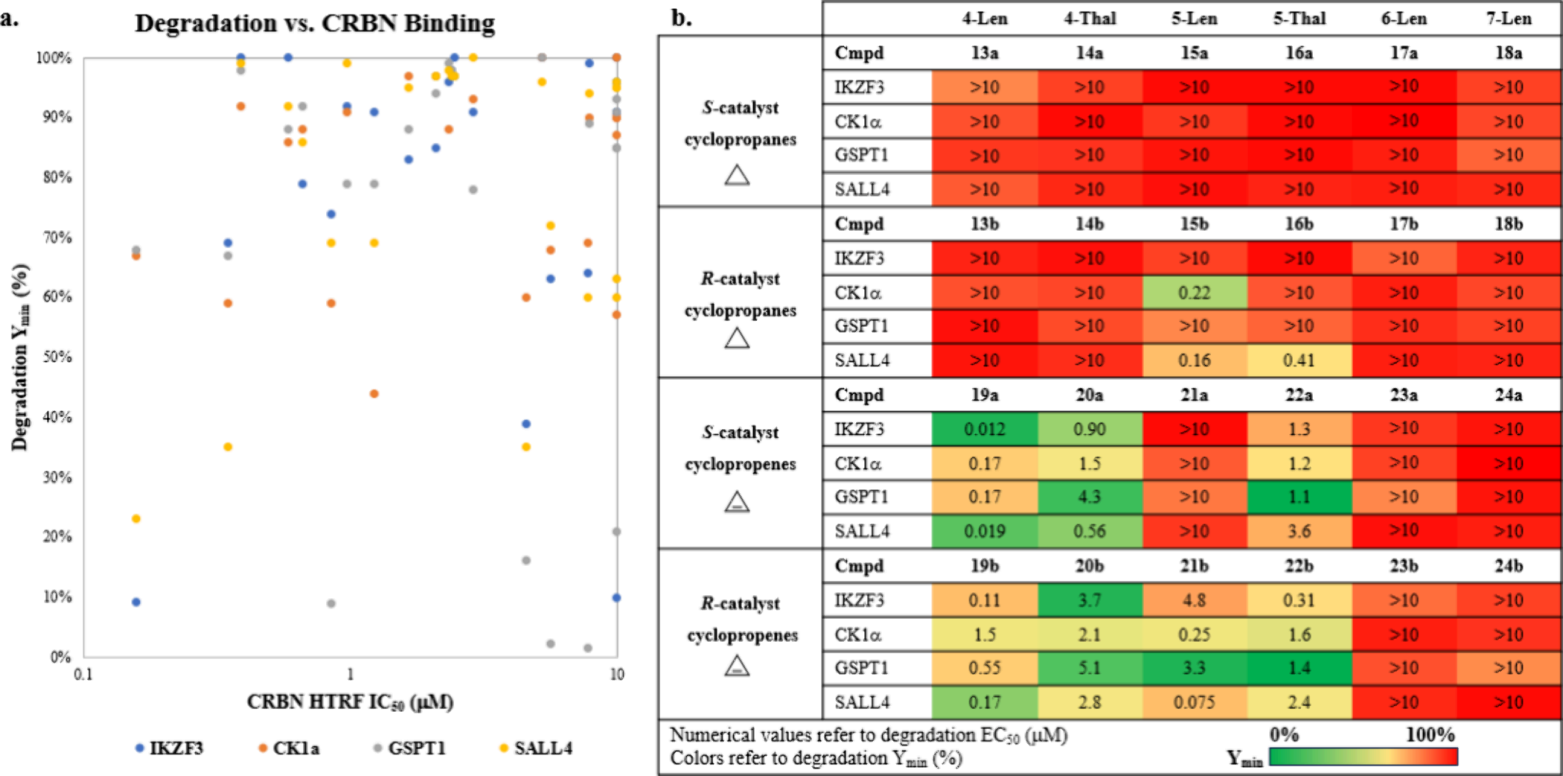
Biological activity and trends. (a) Correlation of CRBN binding (HTRF IC_50_) to neosubstrate degradation (*Y*_min_). (b) Trends in neosubstrate activity with EC_50_ (concentration required to achieve 50% of total degradation effect) reported in μM and boxes colored by *Y*_min_ (with red showing weak depth of degradation and green showing strong depth of degradation); data reported as an average of *N* ≥ 3 test occasions.

When comparing effects of the “Len” vs “Thal” cores (**13a,b** vs **14a,b**, **15a,b** vs **16a,b**, **19a,b** vs **20a,b**, and **21a,b** vs **22a,b**), the matched pairs show similar activity trends with matched pairs being either inactive (**13a,b** and **14a,b**; **15a** and **16a**) or showing some level of activity across neosubstrates (**19a,b** and **20a,b**; **21b** and **22b**). Two exceptions to this trend were 1) **15b** and **16b**, where **15b** was more active than **16b** against CK1α and 2) **21a** and **22a**, where **21a** was inactive across all neosubstrates tested whereas **22a** consistently showed some level of activity across neosubstrates. One hypothesis for the difference in CK1α activity for **15b** and **16b** is that the carbonyl could be interfering with ternary complex formation based on previously reported rationale.^3a^ It is interesting, however, that this carbonyl is better tolerated for CK1α recruitment and degradation for cyclopropene-containing Thal cores (**20a,b** and **22a,b**).

Trends in regiochemistry of the substitution patterns (4- vs 5- vs 6- vs 7-position) were the most universal, with 6- and 7-substituted “Len” cores being generally inactive. However, these compounds maintain binding to CRBN (data available in SI). This suggests that the lack of degradation is due to the inability to form a productive ternary complex between CRBN and the neosubstrates investigated, all of which contain G-loop degrons. While these compounds may be ineffective for recruiting G-loop-containing neosubstrates, this feature could provide degradation selectivity against G-loop-containing off-targets, like GSPT1, which can confound data interpretation.^25^

The general preference for degradation for cyclopropenes over cyclopropanes was not anticipated a priori. Setting aside the inactivity of the 6- and 7-substituted “Len” cores discussed previously, the trend for the cyclopropanes to be less active holds true for 4- and 5-substituted cyclopropanated cores (compounds **13**–**16**) regardless of stereochemistry with the exception of compound **15b**, where (compared to **15a**) stereochemical effects on neosubstrate degradation are observed (*vide infra*). The trend is more striking when comparing the degradation of a singular neosubstrate for matched pairs, such as the difference in IKZF3 degradation between **14b** (EC_50_ > 10 μM, 99% *Y*_min_) and **20b** (EC_50_ = 3.67 μM, 10% *Y*_min_) or **13a** (EC_50_ = 1.86 μM, 79% *Y*_min_) and **19a** (EC_50_ = 0.012 μM, 9.2% *Y*_min_). Other examples highlighting GSPT1 selectivity are **16a** (EC_50_ > 10 μM, 100% *Y*_min_) vs **22a** (EC_50_ 1.4 μM, 2.3% *Y*_min_) and **16b** (EC_50_ = 3.1 μM, 85% *Y*_min_) vs **22b** (EC_50_ = 1.4 μM, 1.4% *Y*_min_). One notable outlier for the cyclopropenes is **21a**, which is the only 4- or 5-substituted cyclopropene that does not significantly degrade any of the neosubstrates tested.

Finally, distal stereochemistry effects on neosubstrate degradation were analyzed. While these changes may appear less significant from a structural perspective, they lead to significant changes in the neosubstrate selectivity. For instance, cyclopropene compound **19a** is a significantly deeper degrader of IKZF3 (9.2% *Y*_min_) than **19b** (69% *Y*_min_). Another pair worth highlighting is **15a** and **15b** against CK1α (92% *Y*_min_ and 44% *Y*_min_ respectively) where the opposite stereochemical preference is observed vs the cyclopropenes. The most conspicuous stereochemical pair is **21a** and **21b**, for which one is universally less active than the other. While the SAR arising from distal changes in stereochemistry may be more nuanced, the demonstration of the ability of distal stereochemistry alone to strongly impact neosubstrate selectivity is in itself significant. This finding highlights the importance of enabling enantioselective methodologies on glutarimide-containing molecules to help medicinal chemists fine-tune neosubstrate selectivity.

We attempted to rationalize the observed binding and degradation data using multiple molecular docking methods. Using publicly available structures for three of the four neosubstrates (PDB IDs: 5FQD, 6XK9, 8U15) and the structure of IKZF1, which has an identical G-loop sequence to IKZF3, in complex with CRBN to replace the unavailable IKZF3 (PDB ID: 8D7Z), we attempted docking using both Glide (SP and induced fit) and MOE-based induced fit docking. For all neosubstrates and methods, there was no trend between docking success or score with binding affinity or *Y*_min_ values. The small molecule focused docking scores likely do not fully capture the intricacies of the ternary complex in which water and protein–protein interactions play a key role as recently suggested, requiring more sophisticated modeling approaches.^26^

In conclusion, we have demonstrated the utility of asymmetric rhodium-catalyzed cyclopropanation and cyclopropenation on glutarimide substrates to yield structurally distinct IMiDs. The reaction conditions are extremely mild, allowing these transformations to occur with high levels of diastereoselectivity and enantioselectivity in the presence of a sensitive functionality innate to IMiDs. This method enables late-stage functionalization and is amenable to diversification. Biological evaluation showed that many of the compounds maintained CRBN binding and degraded G-loop-containing neosubstrates. In addition to identifying general SAR trends, systematic analysis revealed that distal stereochemistry can dramatically influence neosubstrate activity, which can significantly impact efforts to prospectively design new IMiDs. Overall, this work not only reinforces the use of rhodium-carbene chemistry as an enabling synthetic technology for drug discovery but also underscores the importance of leveraging synthetic methods that facilitate the generation of complexity as one of the many ways to catalyze the discovery of novel molecular glue degraders.

## Safety Statement

***Caution!*** Glutarimide-containing compounds such as thalidomide are known reproductive, neurological, and hematological toxins. Care must be exercised to avoid direct contact with glutarimide-containing compounds. The neat compounds and their solutions must only be handled in a chemical fume hood. Any glassware used with glutarimide-containing material should be treated with a 2 M aqueous solution of a strong base such as sodium hydroxide to destroy the material.

## Supporting Information Available

Experimental procedures, NMR spectra, chromatograms, and biological assay protocols and data (PDF)

## Abbreviations

CRBN: Cereblon
CK1α: Casein kinase 1 alpha
EC_50_: Half-maxmimal effective concentration
GSPT1: G1-to-S phase transition 1
HFIP: hexafluoroisopropanol
HTRF: Homogeneous time-resolved fluorescence
IC_50_: Half-maximal inhibitory concentration
IKZF1: Ikaros family zinc finger protein 1
IKZF3: Ikaros family zinc finger protein 3
MOE: Molecular operating environment
PDB: Protein Data Bank
PMI: Principal moments of inertia
p-PhTPCP: *para*-phenyl 1,2,2-triarylcyclopropanecarboxylate
SAR: Structure–activity relationship
SFC: Supercritical fluid chromatography
*S*-tetra-p-Br-PPTTL: (*S*)-(4,5,6,7-tetrakis(4-bromophenyl)phthalimido *tert*-leucine
TBP: Tritryptophan binding pocking
UMAP: Uniform manifold approximation and projection
*Y*_min_: Depth of degradation (i.e., 100% = no reduction of protein levels, 0% = complete degradation)
IMiD: Immunomodulatory imide drug
