# Supplementary material for: Asymmetric Dirhodium-Catalyzed Modification of Immunomodulatory Imide Drugs and Their Biological Assessment
Source: ACS Med Chem Lett. 2024 Aug 23;15(9):1575–83. doi: 10.1021/acsmedchemlett.4c00297 (PMC11403733; doi:10.1021/acsmedchemlett.4c00297)
Supplement: Supplementary file 1 — ml4c00297_si_001.pdf [file ml4c00297_si_001.pdf]

Asymmetric Dirhodium-Catalyzed Modification of Immunomodulatory Imide Drugs and their Biological Assessment

Supporting Information
(92 Pages)

William F. Tracy,[a] Geraint H. M. Davies,[b,†] Lei Jia,[c,†] Ethan D. Evans,[d] Zhenghang Sun,[c] Jennifer Buenviaje,[c] Gody Khambatta,[c] Shan Yu,[c] Lihong Shi,[c] Veerabahu Shanmugasundaram,[b] Jesus Moreno*,[c] Emily C. Cherney*,[e] Huw M. L. Davies*[a]

[a] Department of Chemistry, Emory University, Atlanta, Georgia 30322, United States

[b] Small Molecule Drug Discovery, Bristol Myers Squibb, Cambridge, Massachusetts 02140, United States

[c] Small Molecule Drug Discovery, Bristol Myers Squibb, San Diego, California 92121, United States

[d] Small Molecule Drug Discovery, Bristol Myers Squibb, Redwood City, California 94063, United States

[e] Small Molecule Drug Discovery, Bristol Myers Squibb, Princeton, New Jersey 08543, United States

*Corresponding authors:

Huw M. L. Davies − Department of Chemistry, Emory University, Atlanta, Georgia 30322, United States; Email: hmdavie@emory.edu
Emily C. Cherney – Small Molecule Drug Discovery- Integrated Discovery Medicinal Chemistry, Bristol Myers Squibb, Princeton, New Jersey 08543, United States;
Email: emily.cherney@bms.com
Jesus Moreno – Small Molecule Drug Discovery- Integrated Discovery Medicinal Chemistry, Bristol Myers Squibb, San Diego, California 92121, United States;
Email: jesus.moreno@bms.com

Table of Contents

Scheme S1. Catalyst screen for the cyclopropanation of 4. .... S2
Scheme S2. Combined chromatograms of the stereoisomers of 15b S2
Section 1: General Information S3
Section 2: Synthetic Procedures and Compound Characterization S4
Synthesis of Starting Materials S4
Figure S1. Aryl alkenes used in this study S4
Figure S2. Aryl alkynes used in this study S4
Figure S3. Aryl diazoacetates used in this study S5
Synthesis of Final Compounds S5
Section 3: X-ray Crystallographic Data for *R*-15a S18
Section 4: Spectroscopic Data S29
^{1}H NMR Spectra S29
^{13}C{^{1}H} NMR Spectra S39
Section 5: Chromatographic Data S48
Chiral SFC Chromatograms S48
Purity Chromatograms S72
Section 6: Assay Protocols S89
Section 7: Biological Data Tables S90
Section 8: References S92

Scheme S1. Catalyst screen for the cyclopropanation of 4.

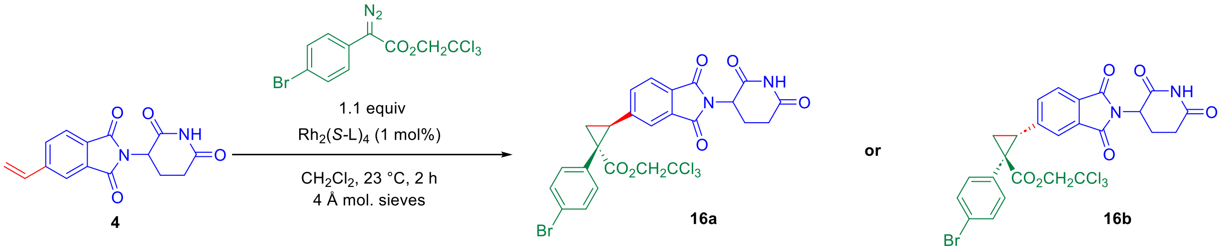

Entry	L	% yield	d.r.[a]	d.r.[b] (16x)
1	TPPTTL	77	>20:1	73:27 (16b)
2	PTAD	80	>20:1	87:13 (16a)
3[c]	NTTL	82	>20:1	95:5 (16b)
4	*p*-Ph-TPCP	72	>20:1	99:1 (16a)

Reactions conducted on material in which the glutarimimide stereocenter is racemic. Entries 1 and 3 gave the stereoisomers of product 16b, equivalent to those produced from the reaction with Rh_2(*R*-p-Ph-TPCP)$_4$. [a] Diastereomeric ratio for the relative configuration of the two new stereogenic centers, determined by ^{1}H NMR analysis. [b] Asymmetric induction arising from formation of the major relative diastereomer, determined by SFC analysis. [c] Reaction run with HFIP as solvent instead of CH_2Cl_2.

Scheme S2. Combined chromatograms of the stereoisomers of 15b

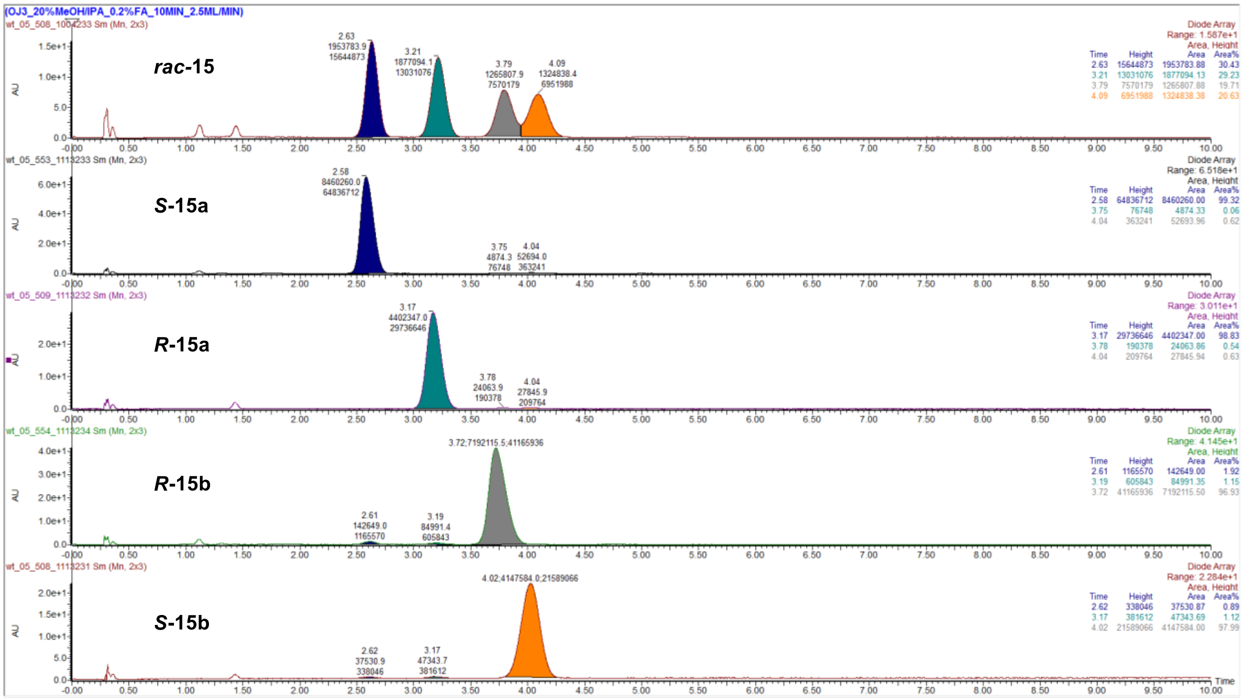

Section 1: General Information

All reactions were conducted in flame-dried glassware under an inert atmosphere of dry nitrogen or argon, unless otherwise noted. All reagents were purchased from Oakwood, CombiBlocks, Millipore Sigma, Ambeed, and WuXi and used as received. Anhydrous dichloromethane was obtained from a Grubbs-type solvent purification system and further dried under an argon atmosphere for 24 hours over 4 Å molecular sieves. Molecular sieves were activated by heating under vacuum (<1 torr) for three hours at 300 °C.

Proton (^{1}H) NMR spectra were recorded at 400 MHz on Varian and Bruker spectrometers, 500 MHz on an Inova-500 spectrometer, or 600 MHz on an Inova-600 spectrometer. Proton-decoupled carbon (^{13}C{^{1}H}) NMR spectra were recorded at 100 MHz on Varian and Bruker spectrometers or 151 MHz on an Inova-600 spectrometer. NMR spectra were recorded in deuterated solvents and were referenced to tetramethylsilane (in the case of CDCl3) or the respective residual solvent signal. NMR chemical shifts were reported in parts per million (ppm). Abbreviations for signal couplings are as follows: s, singlet; d, doublet; t, triplet; q, quartet; quin, quintet; sex, sextet; sep, septet; and m, multiplet. The coupling constants were taken from the spectra directly and are uncorrected.

Mass spectrometric determinations were carried out on a Thermo Finnigan LTQ-FTMS spectrometer with atmospheric pressure chemical ionization (APCI), using a Fourier transform ion cyclotron resonance mass analyzer. Optical rotations were measured on a Rudolph Research Analytical Automatic Polarimeter, APIV-1W. Analytical thin layer chromatography was performed on silica gel plates using ultraviolet light to visualize analytes. Normal phase column chromatography was performed with SiliCycle silica gel 60 Å (50 µm) hand-packed in Biotage Sfaї columns on Biotage Isolera Four chromatographs, with SiliCycle silica gel 60 Å or Celite® as a dry-loading absorbent. Reverse phase chromatography was conducted using Teledyne ISCO RediSep Gold 18 reverse-phase columns using Teledyne ISCO CombiFlash or Biotage Isolera chromatographs. Supercritical fluid chromatography (SFC) analysis was performed on a Waters Acquity UPC2 instrument.

Purity of key compounds was assessed using SFC analysis (Waters Acquity UPC2 instrument), high-performance liquid chromatography analysis (HPLC) on an Agilent 1260 Infinity HPLC, or Waters AutoPurification System. All key compounds were ≥95% pure by HPLC or SFC analysis.

Dirhodium catalysts Rh_2(*S*-p-Ph-TPCP)$_4$,[1] Rh_2(*R*-p-Ph-TPCP)$_4$,[1] Rh_2(*S-tetra*-p-Br-PPTL)$_4$,[2] and Rh_2(*R-tetra*-p-Br-PPTL)$_4$[2] were prepared according to their respective literature procedures. Racemic standards were prepared by using a 1:1 mixture of *R* and *S* catalyst under the same reaction conditions used to prepare enantioenriched compounds.

Caution! Glutarimide-containing compounds such as thalidomide are known reproductive, neurological, and hematological toxins. Care must be exercised to avoid direct contact with glutarimide-containing material; any glassware used with glutarimide-containing material should be treated with a 2 M aqueous solution of a strong base such as sodium hydroxide to destroy the material.

Section 2: Synthetic Procedures and Compound Characterization

Synthesis of Starting Materials

Preparation of Aryl Alkenes.

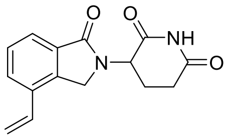

1

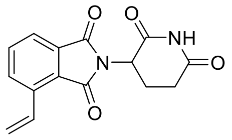

2

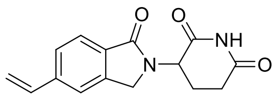

3

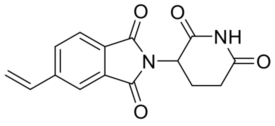

4

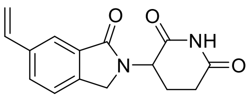

5

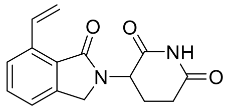

6

Figure S1. Aryl alkenes used in this study

Aryl alkenes **1-6** were prepared following the literature procedure,[3] in 84-93% yield.

Preparation of Aryl Alkynes.

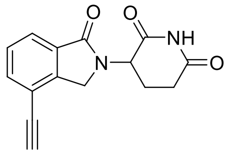

7

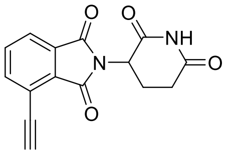

8

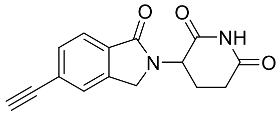

9

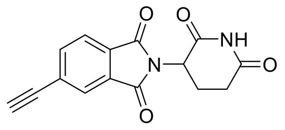

10

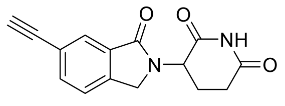

11

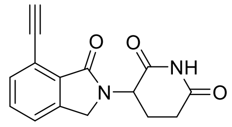

12

Figure S2. Aryl alkynes used in this study

Aryl alkynes **7-12** were prepared in 25-68% yield over two steps following an adapted patent procedure.[4] A flame-dried round-bottom flask was charged with aryl bromide (1.0 equiv), $Pd(PPh_3)_2Cl_2$ (10 mol%), diisopropylethylamine (3.0 equiv), and CuI (10 mol%) under inert atmosphere. Dry *N*,*N*-dimethylformamide (0.20 M) and ethynyltrimethylsilane (5.0 equiv) were charged by syringe, and the reaction was heated to 65 °C overnight. The reaction was concentrated under vacuum and dry-mounted onto silica gel, then purified by flash column chromatography. The isolated material was carried on to the next step without further purification.

The trimethylsilyl-protected alkyne was charged to a flame-dried flask under inert atmosphere. Dry tetrahydrofuran (THF, 0.15 M) was charged via syringe, and the solution was cooled to 0 °C in an ice bath. 1.0 M tetrabutylammonium fluoride in THF (1.2 equiv) was added over thirty minutes via syringe pump to the cooled reaction. The reaction was stirred for thirty minutes longer, after which the reaction was concentrated and purified by flash column chromatography.

Preparation of Aryl Diazoacetates.

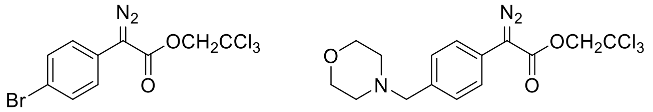

A **B**

Figure S3. Aryl diazoacetates used in this study

2,2,2-trichloroethyl 2-(4-bromophenyl)-2-diazoacetate (**A**) was prepared via the literature procedure, in 90% yield.[5]

2,2,2-trichloroethyl 2-diazo-2-(4-(morpholinomethyl)phenyl)acetate (**B**) was prepared from 2-(4-(bromomethyl)phenyl)acetic acid in three steps. Following an adaptation of the literature procedure,[5] 2-(4-(bromomethyl)phenyl)acetic acid (1.0 g, 1.0 equiv, 4.4 mmol), 2,2,2-trichloroethanol (0.51 mL, 1.2 equiv, 5.2 mmol), and 4-(dimethylamino)pyridine (53 mg, 10 mol%, 0.44 mmol) were dissolved in dry CH_2Cl_2 (10 mL), and cooled to 0 °C. A solution of dicyclohexylcarbodiimide (1.0 g, 4.8 mmol, 1.1 equiv) in CH_2Cl_2 (5.7 mL) was added dropwise to the cooled reaction, which was allowed to stir and come to room temperature overnight. The reaction was filtered through Celite® to remove the white precipitate that had formed, rinsing with diethyl ether, and the filtrate was concentrated in vacuuo. The material was filtered through a short plug of silica gel with diethyl ether, and concentrated in vacuuo. The product obtained was used in the next step without further purification.

Following a procedure from the literature,[6] potassium carbonate (359 mg, 1.3 equiv, 2.6 mmol), morpholine (0.17 mL, 1.0 equiv, 2.0 mmol), and 2,2,2-trichloroethyl 2-(4-(bromomethyl)phenyl)acetate (793 mg, 1.1 equiv, 2.2 mmol) were combined in a flame-dried vial under inert atmosphere, dissolved in dry acetonitrile (6.7 mL), and stirred at room temperature overnight. The acetonitrile was removed in vacuuo and the residue was partitioned between water and ethyl acetate. The aqueous layer was extracted thrice with ethyl acetate, after which the organic layers were washed with brine, dried over magnesium sulfate, and concentrated in vacuuo. The material was filtered through a silica plug with diethyl ether. The product obtained was used in the next step without further purification.

Following the literature procedure,[5] 2,2,2-trichloroethyl 2-(4-(morpholinomethyl)phenyl)acetate (475 mg, 1 equiv, 1.3 mmol) and 2-nitrobenzenesulfonyl azide (443 mg, 1.5 equiv, 1.9 mmol) were taken up in dry acetonitrile (7.2 mL) and cooled to 0 °C. 2,3,4,6,7,8,9,10-octahydropyrimido[1,2-a]azepine (0.43 mL, 2.2 equiv, 2.9 mmol) was added dropwise to the cooled reaction. The reaction was allowed to stir for three hours at 0 °C, after which the reaction was quenched with a saturated solution of ammonium chloride in water. The reaction was extracted with three portions of diethyl ether. The combined organic layers were washed with brine, dried over magnesium sulfate, and concentrated in vacuuo. The crude residue was purified via flash column chromatography (SiO_2, 10-30% EtOAc in hexanes), which gave 2,2,2-trichloroethyl 2-diazo-2-(4-(morpholinomethyl)phenyl)acetate (345 mg, 0.88 mmol, 68% yield) as a yellow-orange amorphous solid.

^{1}H NMR (400 MHz, $CDCl_3$) δ 7.45 (d, J = 8.4 Hz, 1H), 7.38 (d, J = 8.3 Hz, 1H), 4.92 (s, 1H), 3.71 (t, J = 4.7 Hz, 3H), 3.49 (s, 1H), 2.44 (t, J = 4.7 Hz, 2H).

Synthesis of Final Compounds

General Cyclopropanation Procedure (GP 1)

A 10 mL round-bottom flask was charged with a PTFE magnetic stir bar and ca. 200% w/w 4 Å molecular sieves, then flame dried under vacuum. The flask was then backfilled with dry nitrogen. Rh_2(*S*-p-Ph-TPCP)$_4$ (1 mol%) and the aryl alkene (1.0 equiv) were charged to the flask, and the flask was evacuated and backfilled with nitrogen. The flask was equipped with an argon balloon and rubber septum, and dry CH_2Cl_2

(0.2 M) was charged via syringe. A solution of 2,2,2-trichloroethyl 2-(4-bromophenyl)-2-diazoacetate (1.1 equiv) in dry CH_2Cl_2 (0.2 M, prepared in the same manner under inert atmosphere) was added dropwise to the stirred reaction at room temperature. The reaction was allowed to stir for two hours, after which the reaction was filtered through Celite, rinsing the filter pad with CH_2Cl_2. The crude residue was dry-mounted onto silica gel and was purified by flash column chromatography (SiO_2).

General Cyclopropenation Procedure (GP 2)

A 10 mL round-bottom flask was charged with a PTFE magnetic stir bar and ca. 200% w/w 4 Å molecular sieves, then flame dried under vacuum. The flask was then backfilled with dry nitrogen. Rh_2(*S*-p-Ph-TPCP)$_4$ (1 mol%) and the aryl alkyne (1.0 equiv) were charged to the flask, and the flask was evacuated and backfilled with nitrogen. The flask was equipped with an argon balloon and rubber septum, and dry CH_2Cl_2 (0.2 M) was charged via syringe. A solution of 2,2,2-trichloroethyl 2-(4-bromophenyl)-2-diazoacetate (1.1 equiv) in dry CH_2Cl_2 (0.2 M, prepared in the same manner under inert atmosphere) was added over 15 minutes to the stirred reaction at room temperature, via syringe pump. The reaction was allowed to stir for two hours, after which the reaction was filtered through Celite, rinsing the filter pad with CH_2Cl_2. The crude residue was dry-mounted onto silica gel and was purified by flash column chromatography (SiO_2).

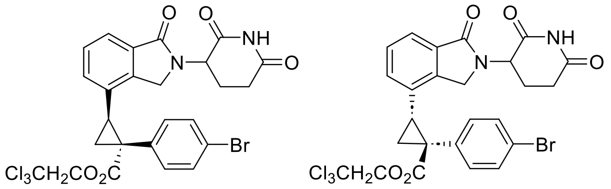

13a **13b**

2,2,2-trichloroethyl (1*R*,2*S*)-1-(4-bromophenyl)-2-(2-(2,6-dioxopiperidin-3-yl)-1-oxoisoindolin-4-yl)cyclopropane-1-carboxylate (13a).

2,2,2-trichloroethyl (1*S*,2*R*)-1-(4-bromophenyl)-2-(2-(2,6-dioxopiperidin-3-yl)-1-oxoisoindolin-4-yl)cyclopropane-1-carboxylate (13b).

Compound **13a** was prepared following **GP 1**, using Rh_2(*S*-p-Ph-TPCP)$_4$ (3.5 mg, 1 mol%, 2.0 μmol), 3-(1-oxo-4-vinylisoindolin-2-yl)piperidine-2,6-dione (54 mg, 1.0 equiv, 0.20 mmol), 2,2,2-trichloroethyl 2-(4-bromophenyl)-2-diazoacetate (82 mg, 1.1 equiv, 0.22 mmol), and CH_2Cl_2 (2 mL) at room temperature for two hours. Purification via flash column chromatography (SiO_2, gradient of 40% to 100% EtOAc in hexanes) afforded 2,2,2-trichloroethyl (1*R*,2*S*)-1-(4-bromophenyl)-2-(2-(2,6-dioxopiperidin-3-yl)-1-oxoisoindolin-4-yl)cyclopropane-1-carboxylate as an amorphous white solid. (78 mg, 0.12 mmol, 62% yield).
Compound **13b** was prepared in the same manner using Rh_2(*R*-p-Ph-TPCP)$_4$, which afforded 2,2,2-trichloroethyl (1*S*,2*R*)-1-(4-bromophenyl)-2-(2-(2,6-dioxopiperidin-3-yl)-1-oxoisoindolin-4-yl)cyclopropane-1-carboxylate as an amorphous white solid. (77 mg, 0.12 mmol, 61% yield).

HRMS (APCI): *m/z* 612.9702 [(M+H)$^+$ requires 612.9694], calc'd for $C_{25}H_{20}{}^{79}Br^{35}Cl_3N_2O_5$
^{1}**H NMR** (600 MHz, $CDCl_3$, reported as a mixture of diasteromers) δ 8.26 and 8.25 (s, 1H), 7.655 and 7.652 (d, J = 7.6 Hz, 1H), 7.28 and 7.26 (d, J = 8.5 Hz, 2H, partially obscured by solvent signal), 7.16-7.10 (m, 1H), 6.89 and 6.86 (d, J = 8.5 Hz, 2H), 6.49 and 6.43 (d, J = 7.7 Hz, 1H), 5.29 and 5.26 (dd, J = 13.2, 5.2 Hz, 1H), 4.88 and 4.87 (d, J = 12.0 Hz, 1H), 4.66 and 4.65 (d, J = 12.0 Hz, 1H), 4.59 and 4.54 (d, J = 16.0 Hz, 1H), 4.39 and 4.37 (d, J = 16.0 Hz, 1H), 3.07 (dd, J = 9.3, 7.3 Hz, 1H), 2.98-2.81 (m, 2H), 2.40-2.20 (m, 3H), 2.12-2.06 (m, 1H).
^{13}**C{^{1}H} NMR** (126 MHz, $CDCl_3$, reported as a mixture of diastereomers) δ 171.4, 171.1, 169.63, 169.59, 169.42, 169.37, 141.6, 133.3, 133.2, 132.3, 131.5, 131.4, 130.7, 129.0, 128.9, 128.4, 122.93, 122.91, 122.2, 74.4, 74.3, 52.1, 52.0, 46.4, 36.8, 36.7, 31.7, 29.9, 29.7, 23.7, 23.6, 19.8, 19.7.
SFC analysis: **13a** (Trefoil® AMY1, 25% 1:1 MeOH:^{i}PrOH with 0.2% formic acid in CO_2, 2.5 mL/min, diode array) indicated 99:1 d.r.: t_R (major diastereomers) = 2.89 and 4.82 min, t_R (minor diastereomers) = 3.24 and 3.96 min. **13b** indicated the opposite diastereomers in 99:1 d.r.
Purity (SFC): **13a** – 99%; **13b** – 99%

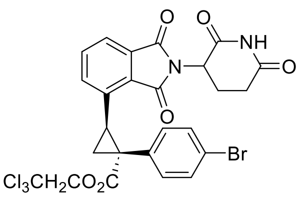

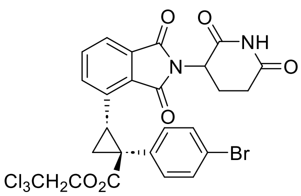

14a **14b**

2,2,2-trichloroethyl (1*R*,2*S*)-1-(4-bromophenyl)-2-(2-(2,6-dioxopiperidin-3-yl)-1,3-dioxoisoindolin-4-yl)cyclopropane-1-carboxylate (14a).

2,2,2-trichloroethyl (1*S*,2*R*)-1-(4-bromophenyl)-2-(2-(2,6-dioxopiperidin-3-yl)-1,3-dioxoisoindolin-4-yl)cyclopropane-1-carboxylate (14b).

Compound **14a** was prepared following **GP 1**, using Rh_2(*S*-p-Ph-TPCP)$_4$ (3.5 mg, 1 mol%, 2.0 μmol), 2-(2,6-dioxopiperidin-3-yl)-4-vinylisoindoline-1,3-dione (57 mg, 1.0 equiv, 0.20 mmol), 2,2,2-trichloroethyl 2-(4-bromophenyl)-2-diazoacetate (82 mg, 1.1 equiv, 0.22 mmol), and CH_2Cl_2 (2 mL) at room temperature for two hours. Purification via flash column chromatography (SiO_2, gradient of 40% to 100% EtOAc in hexanes) afforded 2,2,2-trichloroethyl (1*R*,2*S*)-1-(4-bromophenyl)-2-(2-(2,6-dioxopiperidin-3-yl)-1,3-dioxoisoindolin-4-yl)cyclopropane-1-carboxylate as an amorphous white solid. (40 mg, 62 μmol, 31% yield).
Compound **14b** was prepared in the same manner using Rh_2(*R*-p-Ph-TPCP)$_4$, which afforded 2,2,2-trichloroethyl (1*S*,2*R*)-1-(4-bromophenyl)-2-(2-(2,6-dioxopiperidin-3-yl)-1,3-dioxoisoindolin-4-yl)cyclopropane-1-carboxylate as an amorphous white solid. (38 mg, 59 μmol, 30% yield).
HRMS (APCI): *m/z* 625.9417 ($[M]^+$ requires 625.9422), calc'd for $C_{25}H_{18}BrCl_3N_2O_6$.
^{1}H NMR (500 MHz, $CDCl_3$, reported as a mixture of diastereomers) δ 8.22 and 8.21 (s, 1H), 7.624 and 7.617 (dd, *J* = 7.40, 0.70 Hz, 1H), 7.38-7.33 (m, 1H), 7.27 (d, *J* =7.9 Hz, 2H, partially obscured by solvent signal), 6.95 (t, *J*=8.2 Hz, 2H), 6.77 and 6.68 (d, *J* =8.0 Hz, 1H), 5.00 (dt, *J* = 12.4, 6.1 Hz, 1H), 4.95 and 4.93 (d, J =11.9 Hz, 1H), 4.64 and 4.61 (d, *J* =11.2 Hz, 1H), 4.21-4.09 (m, 1H) 2.95-2.72 (m, 3H), 2.39 and 2.35 (dd, *J* = 9.1, 5.4 Hz, 1H), 2.20-2.13 (m, 2H).
^{13}C{^{1}H} NMR (101 MHz, $CDCl_3$, reported as a mixture of diastereomers) δ 171.1, 171.0, 170.9, 168.1, 168.0, 167.9, 166.95, 166.93, 136.3, 136.1, 134.0, 133.9, 133.2, 133.1, 132.4, 132.37, 132.32, 132.19, 132.17, 131.40, 131.37, 130.0, 129.8, 122.4, 122.3, 122.1, 122.0, 94.8, 74.81, 74.76, 49.5, 37.1, 36.8, 31.5, 29.5, 29.0, 22.8, 22.7, 19.7, 19.3.
SFC analysis: 14a (Chiralcel® OX-3, 35% 1:1 MeOH:^{i}PrOH with 0.2% formic acid in CO_2, 2.5 mL/min, diode array) indicated >99:1 d.r.: t_R (major diastereomers) = 2.16 and 2.36 min, t_R (minor diastereomers) = 4.09 and 7.32 min. **14b** indicated the opposite diastereomers in 99:1 d.r.
Purity (SFC): 14a – 99%; **14b** – 96%

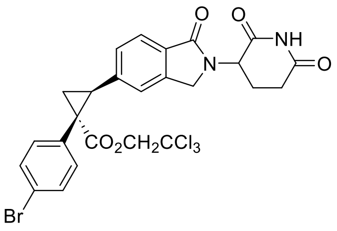

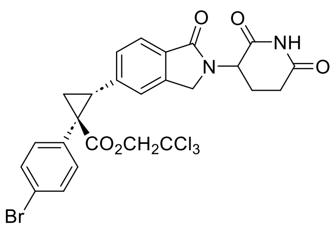

15a **15b**

2,2,2-trichloroethyl (1*R*,2*S*)-1-(4-bromophenyl)-2-(2-(2,6-dioxopiperidin-3-yl)-1-oxoisoindolin-5-yl)cyclopropane-1-carboxylate (15a).

2,2,2-trichloroethyl (1*S*,2*R*)-1-(4-bromophenyl)-2-(2-(2,6-dioxopiperidin-3-yl)-1-oxoisoindolin-5-yl)cyclopropane-1-carboxylate (15b).

Compound **15a** was prepared following **GP 1**, using Rh_2(*S*-p-Ph-TPCP)$_4$ (3.5 mg, 1 mol%, 2.0 μmol), 3-(1-oxo-5-vinylisoindolin-2-yl)piperidine-2,6-dione (54 mg, 1.0 equiv, 0.20 mmol), 2,2,2-trichloroethyl 2-(4-bromophenyl)-2-diazoacetate (82 mg, 1.1 equiv, 0.22 mmol), and CH_2Cl_2 (2 mL) at room temperature for two hours. Purification via flash column chromatography (SiO_2, gradient of 25% to 75% EtOAc in hexanes)

afforded 2,2,2-trichloroethyl (1*R*,2*S*)-1-(4-bromophenyl)-2-(2-(2,6-dioxopiperidin-3-yl)-1-oxoisoindolin-5-yl)cyclopropane-1-carboxylate as an amorphous white solid. (95 mg, 0.16 mmol, 77% yield).
Compound **15b** was prepared in the same manner using Rh_2(*R*-p-Ph-TPCP)$_4$, which afforded 2,2,2-trichloroethyl (1*S*,2*R*)-1-(4-bromophenyl)-2-(2-(2,6-dioxopiperidin-3-yl)-1-oxoisoindolin-5-yl)cyclopropane-1-carboxylate as an amorphous white solid. (97 mg, 0.16 mmol, 79% yield).

HRMS (APCI): *m/z* 612.9702 $[(M+H)^+]$ requires 612.9686], calc'd for $C_{25}H_{20}BrCl_3N_2O_5$
^{1}H NMR (500 MHz, $CDCl_3$, reported as a mixture of diastereomers) 8.42 (s, 1H), 7.60 (12.0, 8.0 Hz, 1H), 7.28 and 7.27 (d, *J*=8.5 Hz, 2H), 6.96-6.93 (m, 3H), 6.86-6.85 (m, 1H), 5.16 (ddd, *J*=18.0, 13.3, 5.2 Hz), 4.83 and 4.81 (d, *J*=2.8 Hz, 1H), 4.66 and 4.64 (d, *J*=3.2 Hz, 1H), 4.34 and 4.31 (d, *J*=16.0 Hz, 1H), 4.21 and 4.10 (d, 16.0 Hz, 1H), 3.29 (dd, *J*=9.4, 7.2 Hz, 1H), 2.89-2.76 (m, 2H), 2.36-2.23 (m, 2H), 2.23-2.18 (m, 1H), 2.03-2.00 (m, 1H).
^{13}C{^{1}H} NMR (101 MHz, $CDCl_3$, reported as a mixture of diastereomers) δ 171.33, 171.30, 171.3, 169.72, 169.66, 169.0, 141.50, 141.48, 140.4, 133.7, 133.6, 132.5, 132.4, 131.46, 131.38, 130.33, 130.27, 128.7, 128.3, 123.8, 122.9, 122.4, 122.1, 122.0, 94.9, 74.6, 52.0, 51.8, 47.0, 46.8, 37.4, 37.3, 33.9, 33.8, 31.6, 23.54, 23.47, 20.91, 20.86.
SFC analysis: 15a (Chiralcel® OJ-3, 30% 1:1 MeOH:^{i}PrOH with 0.2% formic acid in CO_2, 2.5 mL/min, diode array) indicated >99:1 d.r.: t_R (major diastereomers) = 2.72 and 4.60 min, t_R (minor diastereomers) = 3.38 and 3.98 min. **15b** indicated the opposite diastereomers in >99:1 d.r.
Purity (SFC): 15a – 99%; **15b** – 99%

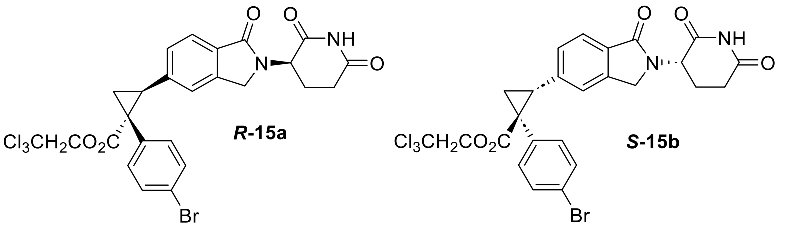

***R*-15a** ***S*-15b**

2,2,2-trichloroethyl (1*R*,2*S*)-1-(4-bromophenyl)-2-(2-((*R*)-2,6-dioxopiperidin-3-yl)-1-oxoisoindolin-5-yl)cyclopropane-1-carboxylate (*R*-15a).

2,2,2-trichloroethyl (1*S*,2*R*)-1-(4-bromophenyl)-2-(2-((*S*)-2,6-dioxopiperidin-3-yl)-1-oxoisoindolin-5-yl)cyclopropane-1-carboxylate (*S*-15b).

Compound ***S*-15a** was prepared following **GP 1**, using Rh_2(*S*-p-Ph-TPCP)$_4$ (3.5 mg, 1 mol%, 2.0 μmol), (*S*)-3-(1-oxo-5-vinylisoindolin-2-yl)piperidine-2,6-dione (54 mg, 1.0 equiv, 0.20 mmol), 2,2,2-trichloroethyl 2-(4-bromophenyl)-2-diazoacetate (82 mg, 1.1 equiv, 0.22 mmol), and CH_2Cl_2 (2 mL) at room temperature for two hours. Purification via flash column chromatography (SiO_2, gradient of 25% to 75% EtOAc in hexanes) afforded 2,2,2-trichloroethyl (1*R*,2*S*)-1-(4-bromophenyl)-2-(2-((*S*)-2,6-dioxopiperidin-3-yl)-1-oxoisoindolin-5-yl)cyclopropane-1-carboxylate as an amorphous white solid. (115 mg, 0.19 mmol, 94% yield).
Compound ***R*-15b** was prepared in the same manner using (*R*)-3-(1-oxo-5-vinylisoindolin-2-yl)piperidine-2,6-dione and Rh_2(*R*-p-Ph-TPCP)$_4$, which afforded 2,2,2-trichloroethyl (1*S*,2*R*)-1-(4-bromophenyl)-2-(2-((*R*)-2,6-dioxopiperidin-3-yl)-1-oxoisoindolin-5-yl)cyclopropane-1-carboxylate as an amorphous white solid. (117 mg, 0.19 mmol, 95% yield).
^{1}H NMR (400 MHz, $CDCl_3$) δ 8.00 (s, 1H), 7.61 (d, *J* = 7.9 Hz, 1H), 7.29 (d, *J* = 8.5 Hz, 2H), 7.00 – 6.91 (m, 3H), 6.87 (dd, *J* = 7.9, 1.5 Hz, 1H), 5.15 (dd, *J* = 13.2, 5.2 Hz, 1H), 4.83 (d, *J* = 11.9 Hz, 1H), 4.65 (d, *J* = 11.9 Hz, 1H), 4.32 (d, *J* = 16.0 Hz, 1H), 4.22 (d, *J* = 16.0 Hz, 1H), 3.30 (dd, *J* = 9.3, 7.4 Hz, 1H), 2.94 – 2.87 (m, 1H), 2.87-2.73 (m, 1H), 2.41 – 2.26 (m, 2H), 2.25-2.13 (m, 1H), 2.06 – 1.95 (m, 1H).

^{13}C{^{1}H} NMR (101 MHz, $CDCl_3$) δ 171.5, 171.3, 169.8, 168.9, 141.5, 140.4, 133.6, 132.3, 131.3, 130.3, 128.2, 123.7, 122.9, 122.0, 94.9, 74.5, 51.9, 47.0, 37.3, 33.8, 31.6, 23.4, 20.9.

SFC analysis: *S*-15a (Chiralcel® OJ-3, 30% 1:1 MeOH:^{i}PrOH with 0.2% formic acid in CO_2, 2.5 mL/min, diode array) indicated 98:2 d.r.: t_R (major diastereomers) = 4.08 min (peak representing minor enantiomer of ***S*-15a** n.d.), t_R (minor diastereomers) = 2.64 and 3.22 min. **R-15b** indicated the opposite diastereomers in 96:4 d.r.

Specific rotation: ***S*-15a** [α]D[22] 12.7 (c 1, $CHCl_3$) **R-15b** [α]D[22] -17.6 (c 1.9, $CHCl_3$)
Purity (SFC): ***S*-15a** – 97%; ***R*-15b** – 98%

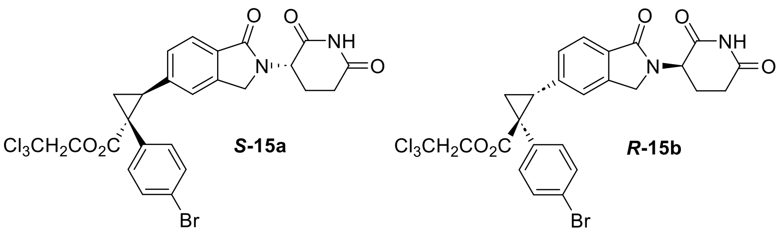

***S*-15a** ***R*-15b**

2,2,2-trichloroethyl (1*R*,2*S*)-1-(4-bromophenyl)-2-(2-((*S*)-2,6-dioxopiperidin-3-yl)-1-oxoisoindolin-5-yl)cyclopropane-1-carboxylate (*S*-15a).

2,2,2-trichloroethyl (1*S*,2*R*)-1-(4-bromophenyl)-2-(2-((*R*)-2,6-dioxopiperidin-3-yl)-1-oxoisoindolin-5-yl)cyclopropane-1-carboxylate (*R*-15b).

Compound ***R*-15a** was prepared following **GP 1**, using Rh_2(*S*-p-Ph-TPCP)$_4$ (3.5 mg, 1 mol%, 2.0 µmol), (*R*)-3-(1-oxo-5-vinylisoindolin-2-yl)piperidine-2,6-dione (54 mg, 1.0 equiv, 0.20 mmol), 2,2,2-trichloroethyl 2-(4-bromophenyl)-2-diazoacetate (82 mg, 1.1 equiv, 0.22 mmol), and CH_2Cl_2 (2 mL) at room temperature for two hours. Purification via flash column chromatography (SiO_2, gradient of 25% to 75% EtOAc in hexanes) afforded 2,2,2-trichloroethyl (1*R*,2*S*)-1-(4-bromophenyl)-2-(2-((*S*)-2,6-dioxopiperidin-3-yl)-1-oxoisoindolin-5-yl)cyclopropane-1-carboxylate as an amorphous white solid. (118 mg, 0.19 mmol, 96% yield).
Compound ***S*-15b** was prepared in the same manner using (*S*)-3-(1-oxo-5-vinylisoindolin-2-yl)piperidine-2,6-dione and Rh_2(*R*-p-Ph-TPCP)$_4$, which afforded 2,2,2-trichloroethyl (1*S*,2*R*)-1-(4-bromophenyl)-2-(2-((*S*)-2,6-dioxopiperidin-3-yl)-1-oxoisoindolin-5-yl)cyclopropane-1-carboxylate as an amorphous white solid. (117 mg, 0.19 mmol, 95% yield).
^{1}H NMR (400 MHz, $CDCl_3$) δ 7.97 (s, 1H), 7.63 (d, *J* = 7.9 Hz, 1H), 7.29 (d, *J* = 8.5 Hz, 2H), 6.98 – 6.93 (m, 3H), 6.86 (s, 1H), 5.19 (dd, *J* = 13.2, 5.3 Hz, 1H), 4.83 (d, *J* = 11.9 Hz, 1H), 4.66 (d, *J* = 11.9 Hz, 1H), 4.36 (d, *J* = 16.0 Hz, 1H), 4.12 (d, *J* = 16.1 Hz, 1H), 3.30 (dd, *J* = 9.3, 7.4 Hz, 1H), 2.97-2.87, (m, 1H), 2.87-2.72 (m, 1H), 2.41 – 2.27 (m, 2H), 2.27 – 2.15 (m, 1H), 2.08 – 1.94 (m, 1H).
^{13}C NMR (101 MHz, $CDCl_3$) δ 171.5, 171.3, 169.8, 169.0, 141.5, 140.4, 133.6, 132.4, 131.3, 130.2, 128.6, 123.7, 122.4, 121.9, 94.9, 74.6, 51.8, 46.8, 37.4, 33.8, 31.6, 23.5, 20.8.
SFC analysis: ***R*-15a** (Chiralcel® OJ-3, 30% 1:1 MeOH:^{i}PrOH with 0.2% formic acid in CO_2, 2.5 mL/min, diode array) indicated 99:1 d.r.: t_R (major diastereomers) = 2.53 min (peak representing minor enantiomer of ***R*-15a** n.d.), t_R (minor diastereomers) = 3.71 and 3.98 min. ***S*-15b** indicated the opposite diastereomers in 98:2 d.r.
Specific rotation: ***R*-15a** [α]D[22] -4.8 (c 1, $CHCl_3$) ***S*-15b** [α]D[22] 5.5 (c 1.9, $CHCl_3$)
Purity (SFC): ***R*-15a** – 96%; ***S*-15b** – 97%

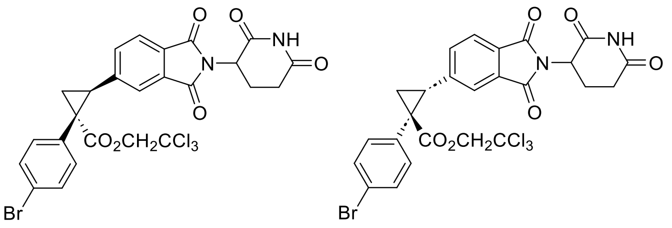

16a **16b**

2,2,2-trichloroethyl (1*R*,2*S*)-1-(4-bromophenyl)-2-(2-(2,6-dioxopiperidin-3-yl)-1,3-dioxoisoindolin-5-yl)cyclopropane-1-carboxylate (16a).

2,2,2-trichloroethyl (1*S*,2*R*)-1-(4-bromophenyl)-2-(2-(2,6-dioxopiperidin-3-yl)-1,3-dioxoisoindolin-5-yl)cyclopropane-1-carboxylate (16b).

Compound **16a** was prepared following **GP 1**, using Rh_2(*S*-p-Ph-TPCP)$_4$ (3.5 mg, 1 mol%, 2.0 µmol), 2-(2,6-dioxopiperidin-3-yl)-5-vinylisoindoline-1,3-dione (57 mg, 1.0 equiv, 0.20 mmol), 2,2,2-trichloroethyl 2-(4-bromophenyl)-2-diazoacetate (82 mg, 1.1 equiv, 0.22 mmol), and CH_2Cl_2 (2 mL) at room temperature for two

hours. Purification via flash column chromatography (SiO_2, gradient of 20% to 60% EtOAc in hexanes) afforded 2,2,2-trichloroethyl (1*R*,2*S*)-1-(4-bromophenyl)-2-(2-(2,6-dioxopiperidin-3-yl)-1,3-dioxoisoindolin-5-yl)cyclopropane-1-carboxylate as an amorphous white solid. (90 mg, 0.14 mmol, 72% yield).
Compound **16b** was prepared in the same manner using Rh_2(*R*-p-Ph-TPCP)$_4$, which afforded 2,2,2-trichloroethyl (1*S*,2*R*)-1-(4-bromophenyl)-2-(2-(2,6-dioxopiperidin-3-yl)-1,3-dioxoisoindolin-5-yl)cyclopropane-1-carboxylate as an amorphous white solid. (90 mg, 0.14 mmol, 72% yield).

HRMS (APCI): *m/z* 626.9492 $[(M+H)^+$ requires 625.9487], calc'd for $C_{25}H_{18}{}^{79}Br^{35}Cl_3N_2O_6$

^{1}H NMR (500 MHz, $CDCl_3$, reported as a mixture of diastereomers) 8.49 (s, 1H), 7.59 and 7.58 (d, *J*=2.0 Hz, 1H), 7.40 (d, 10.2 Hz, 1H), 7.29 (d, 8.0 Hz, 2H), 7.12-7.10 (m, 1H), 6.95 (d, *J*=8.0 Hz, 2H), 4.93 (dd, *J*=12.2, 5.3 Hz, 1H), 4.83 (d, 12.0 Hz, 1H), 4.65 (d, 12.0 Hz, 1H), 3.37-3.33 (m, 1H), 2.88-2.71 (m, 3H), 2.37 (dd, 9.2, 5.5 Hz, 1H), 2.13-2.08 (m, 2H).

^{13}C{^{1}H} NMR (126 MHz, $CDCl_3$, reported as a mixture of diastereomers) δ 171.2, 170.9, 168.2, 167.04, 167.02, 166.90, 166.88, 143.72, 143.70, 133.91, 133.88, 133.47, 133.46, 131.9, 131.8, 131.6, 130.1, 129.1, 128.3, 125.4, 123.4, 123.47, 123.44, 122.3, 94.8, 74.7, 49.42, 49.39, 37.76, 37.74, 33.4, 31.42, 31.40, 22.69, 22.64, 20.68, 20.64.

SFC analysis: 16a (Trefoil® CEL1, 20% 1:1 MeOH:^{i}PrOH with 0.2% formic acid in CO_2, 2.5 mL/min, diode array) indicated 99:1 d.r.: t_R (major diastereomers) = 5.17 and 5.62 min, t_R (minor diastereomers) = 4.31 and 4.92 min. **16b** indicated the opposite diastereomers in 98:2 d.r.

Purity (SFC): 16a – 98%; **16b** – 96%

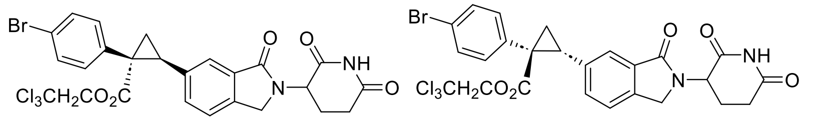

17a **17b**

2,2,2-trichloroethyl (1*R*,2*S*)-1-(4-bromophenyl)-2-(2-(2,6-dioxopiperidin-3-yl)-3-oxoisoindolin-5-yl)cyclopropane-1-carboxylate (17a).

2,2,2-trichloroethyl (1*S*,2*R*)-1-(4-bromophenyl)-2-(2-(2,6-dioxopiperidin-3-yl)-3-oxoisoindolin-5-yl)cyclopropane-1-carboxylate (17b).

Compound **17a** was prepared following **GP 1** using Rh_2(*S*-p-Ph-TPCP)$_4$ (3.5 mg, 1 mol%, 2.0 μmol), 3-(1-oxo-6-vinylisoindolin-2-yl)piperidine-2,6-dione (54 mg, 1.0 equiv, 0.20 mmol), 2,2,2-trichloroethyl 2-(4-bromophenyl)-2-diazoacetate (82 mg, 1.1 equiv, 0.22 mmol), and CH_2Cl_2 (2 mL) at room temperature for two hours. Purification via flash column chromatography (SiO_2, gradient of 20% to 75% EtOAc in hexanes) afforded 2,2,2-trichloroethyl (1*R*,2*S*)-1-(4-bromophenyl)-2-(2-(2,6-dioxopiperidin-3-yl)-3-oxoisoindolin-5-yl)cyclopropane-1-carboxylate as an amorphous white solid. (96 mg, 0.16 mmol, 78% yield).
Compound **17b** was prepared in the same manner using Rh_2(*R*-p-Ph-TPCP)$_4$, which afforded 2,2,2-trichloroethyl (1*S*,2*R*)-1-(4-bromophenyl)-2-(2-(2,6-dioxopiperidin-3-yl)-3-oxoisoindolin-5-yl)cyclopropane-1-carboxylate as an amorphous white solid. (95 mg, 0.15 mmol, 77% yield).

HRMS (APCI): *m/z* 612.9698 $[(M+H)^+$ requires 612.9694], calc'd for $C_{25}H_{21}O_5N_2{}^{79}Br^{35}Cl_3$

^{1}H NMR (600 MHz, $CDCl_3$, reported as a mixture of diastereomers) δ 7.94 (s, 1H), 7.53 and 7.43 (s, 1H), 7.26 (d, *J* = 8.6 Hz, 2H, partially obscured by solvent signal), 7.21 and 7.19 (d, *J* = 7.9 Hz, 1H), 7.02 and 6.92 (dd, *J* = 8.0, 1.7 Hz, 1H), 6.96 (d, *J* = 8.6 Hz, 2H), 5.20 and 5.17 (dd, *J* = 15.6, 5.0 Hz, 1H), 4.843 and 4.841 (d, *J* = 12.0 Hz, 1H), 4.65 and 4.648 (d, *J* = 11.9 Hz, 1H), 4.41 and 4.39 (d, *J* = 16.0 Hz, 1H), 4.25 and 4.24 (d, *J* = 15.9 Hz, 0H), 3.34 (t, *J* = 8.3 Hz, 1H), 2.97 – 2.88 (m, 1H), 2.86 – 2.77 (m, 1H), 2.45 – 2.26 (m, 2H), 2.24-2.18 (m, 1H), 2.10 – 1.97 (m, 1H).

^{13}C{^{1}H} NMR (101 MHz, $CDCl_3$, reported as a mixture of diastereomers) δ 171.4, 171.3, 169.72, 169.69, 169.1, 140.20, 140.18, 136.23, 136.21, 133.7, 133.6, 132.5, 132.2, 131.62, 131.58, 131.28, 131.26, 124.4, 123.7, 122.7, 121.9, 121.8, 95.0, 74.6, 51.98, 51.85, 47.0, 46.8, 36.94,36.87, 33.57, 31.64, 31.62, 23.5, 20.1, 20.0.

SFC analysis: 17a (Trefoil® CEL1, 25% 1:1 EtOH:^{i}PrOH with 0.2% formic acid in CO_2, 2.0 mL/min, diode array) indicated 98:2 d.r.: t_R (major diastereomers) = 4.85 and 5.60 min, t_R (minor diastereomers) = 4.34 and 4.72min. **17b** indicated the opposite diastereomers in 97:3 d.r.

Purity (SFC): 17a – 99%; **17b** – 95%

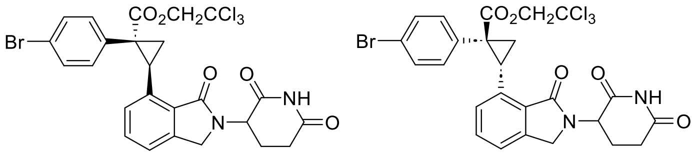

18a **18b**

2,2,2-trichloroethyl (1*R*,2*S*)-1-(4-bromophenyl)-2-(2-(2,6-dioxopiperidin-3-yl)-3-oxoisoindolin-4-yl)cyclopropane-1-carboxylate (18a).

2,2,2-trichloroethyl (1*S*,2*R*)-1-(4-bromophenyl)-2-(2-(2,6-dioxopiperidin-3-yl)-3-oxoisoindolin-4-yl)cyclopropane-1-carboxylate (18b).

Compound **18a** was prepared following **GP 1**, using Rh_2(*S*-p-Ph-TPCP)$_4$ (3.5 mg, 1 mol%, 2.0 μmol), 3-(1-oxo-7-vinylisoindolin-2-yl)piperidine-2,6-dione (54 mg, 1.0 equiv, 0.20 mmol), 2,2,2-trichloroethyl 2-(4-bromophenyl)-2-diazoacetate (82 mg, 1.1 equiv, 0.22 mmol), and CH_2Cl_2 (2 mL) at room temperature for two hours. Purification via flash column chromatography (SiO_2, gradient of 50% to 60% EtOAc in hexanes) afforded 2,2,2-trichloroethyl (1*R*,2*S*)-1-(4-bromophenyl)-2-(2-(2,6-dioxopiperidin-3-yl)-3-oxoisoindolin-4-yl)cyclopropane-1-carboxylate as an amorphous white solid. (35 mg, 56 μmol, 28% yield).
Compound **18b** was prepared in the same manner using Rh_2(*R*-p-Ph-TPCP)$_4$, which afforded 2,2,2-trichloroethyl (1*S*,2*R*)-1-(4-bromophenyl)-2-(2-(2,6-dioxopiperidin-3-yl)-3-oxoisoindolin-4-yl)cyclopropane-1-carboxylate as an amorphous white solid. (36 mg, 57 μmol, 29% yield).
HRMS (APCI): *m/z* 612.9698 $[(M+H)^+$ requires 612.9694], calc'd for $C_{25}H_{21}O_5N_2{}^{79}Br^{35}Cl_3$
^{1}H NMR (400 MHz, $CDCl_3$, reported as a mixture of diastereomers) δ 8.30 and 8.23 (s, 1H), 7.24 – 7.14 (m, 4H), 7.00 and 6.97 (d, *J* = 8.5 Hz, 2H), 6.60-6.52 and 6.52-6.45 (m, 1H), 5.27 and 5.19 (dd, *J* = 13.3, 5.1 Hz, 1H), 4.91 and 4.89, (d, *J* = 11.9 Hz, 1H), 4.67 and 4.65 (d, *J* = 11.9 Hz, 1H), 4.47-4.22 (m, 3H), 3.00 – 2.75 (m, 2H), 2.50 – 2.18 (m, 3H), 2.15-2.10 (m, 1H).
^{13}C{^{1}H} NMR (101 MHz, $CDCl_3$, reported as a mixture of diastereomers) δ 171.5 and 171.4, 171.34 and 171.30, 170.0 and 169.73, 169.72 and 169.61, 142.14 and 142.12, 135.47 and 135.45, 133.45 and 133.28. 133.42 and 133.38, 131.6 and 131.5, 131.0 and 130.9, 130.1 and 129.9, 126.1 and 126.0, 121.7 and 121.6, 121.49, 121.46, 95.1, 74.8 and 74.7, 52.1 and 51.9, 46.9 and 46.6, 36.8 and 36.5, 31.8 and 31.7, 29.6 and 29.3, 23.6 and 23.4, 19.2 and 19.0.
SFC analysis: 18a (Trefoil® AMY1, 30% 1:1 MeOH:^{i}PrOH with 0.2% formic acid in CO_2, 2.5 mL/min, diode array) indicated 99:1 d.r.: t_R (major diastereomers) = 3.65 and 5.13 min, t_R (minor diastereomers) = 3.17 and 4.24 min. **18b** indicated the opposite diastereomers in 99:1 d.r.
Purity (SFC): 18a – 98%; **18b** – 99%

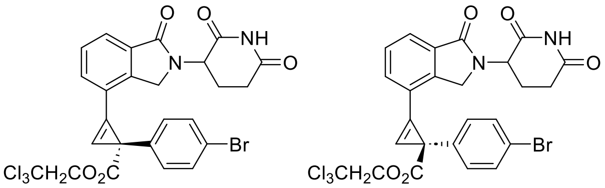

19a **19b**

2,2,2-trichloroethyl (1*S*)-1-(4-bromophenyl)-2-(2-(2,6-dioxopiperidin-3-yl)-1-oxoisoindolin-4-yl)cycloprop-2-ene-1-carboxylate (19a).

2,2,2-trichloroethyl (1*R*)-1-(4-bromophenyl)-2-(2-(2,6-dioxopiperidin-3-yl)-1-oxoisoindolin-4-yl)cycloprop-2-ene-1-carboxylate (19b).

Compound **19a** was prepared following **GP 2**, using Rh_2(*S*-p-Ph-TPCP)$_4$ (3.5 mg, 1 mol%, 2.0 μmol), 3-(4-ethynyl-1-oxoisoindolin-2-yl)piperidine-2,6-dione (54 mg, 1.0 equiv, 0.20 mmol), 2,2,2-trichloroethyl 2-(4-bromophenyl)-2-diazoacetate (82 mg, 1.1 equiv, 0.22 mmol), and CH_2Cl_2 (2 mL) at room temperature for two hours. Purification via flash column chromatography (SiO_2, gradient of 30% to 90% EtOAc in hexanes)

afforded 2,2,2-trichloroethyl (1*S*)-1-(4-bromophenyl)-2-(2-(2,6-dioxopiperidin-3-yl)-1-oxoisoindolin-4-yl)cycloprop-2-ene-1-carboxylate as an amorphous white solid. (76 mg, 0.12 mmol, 62% yield).
Compound **19b** was prepared in the same manner using Rh_2(*R*-p-Ph-TPCP)$_4$, which afforded 2,2,2-trichloroethyl (1*R*)-1-(4-bromophenyl)-2-(2-(2,6-dioxopiperidin-3-yl)-1-oxoisoindolin-4-yl)cycloprop-2-ene-1-carboxylate as an amorphous white solid. (79 mg, 0.13 mmol, 64% yield).
HRMS (APCI): *m/z* 610.9536 $[(M+H)^+$ requires 610.9537], calc'd for $C_{25}H_{19}O_5N_2{}^{79}Br^{35}Cl_3$
^{1}H NMR (600 MHz, $CDCl_3$, reported as a mixture of diastereomers) δ 8.27 (s, 1H), 7.96 (d, *J* = 7.6 Hz, 1H), 7.74 (t, *J* = 7.5 Hz, 1H), 7.58 (td, *J* = 7.6, 2.7 Hz, 1H), 7.444 and 7.437 (d, *J* = 8.5 Hz, 2H), 7.39 and 7.38 (s, 1H), 7.29 and 7.28 (d, *J* = 8.5 Hz, 2H), 5.25 and 5.22 (dd, *J* = 13.4, 5.2 Hz, 1H), 4.81 and 4.80 (d, *J* = 12 Hz, 1H), 4.762 and 4.760 (d, *J* = 12 Hz, 1H), 4.60 and 4.58 (d, *J* = 15.7 Hz, 1H), 4.43 and 4.40 (d, 16.5 Hz, 1H), 2.95-2.89 (m, 1H), 2.88-2.81 (m, 1H), 2.40-2.30 (m, 1H), 2.26 – 2.18 (m, 1H).
^{13}C{^{1}H} NMR (101 MHz, $CDCl_3$, reported as a mixture of diastereomers) δ 171.92, 171.90, 171.0, 169.5, 169.4, 168.60, 168.58, 142.41, 142.40, 138.6, 138.5, 133.02, 133.97, 132.60, 132.57, 131.6, 129.9, 129.32, 126.29, 121.3, 120.32, 120.29, 113.7, 113.3, 102.41, 102.37, 95.0, 74.5, 74.4, 52.1, 52.0, 46.8, 46.7, 32.5, 32.4, 31.6, 23.6.
SFC analysis: 19a (Trefoil® AMY1, 30% 1:1 MeOH:^{i}PrOH with 0.2% formic acid in CO_2, 2.5 mL/min, diode array) indicated 98:2 d.r.: t_R (major diastereomers) = 3.00 and 3.55 min, t_R (minor diastereomers) = 4.41 and 5.81 min. **19b** indicated the opposite diastereomers in 98:2 d.r.
Purity (SFC): 19a – 98%; **19b** – 95%

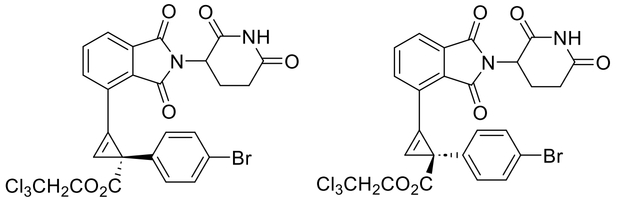

20a **20b**

2,2,2-trichloroethyl (1*S*)-1-(4-bromophenyl)-2-(2-(2,6-dioxopiperidin-3-yl)-1,3-dioxoisoindolin-4-yl)cycloprop-2-ene-1-carboxylate (20a).

2,2,2-trichloroethyl (1*R*)-1-(4-bromophenyl)-2-(2-(2,6-dioxopiperidin-3-yl)-1,3-dioxoisoindolin-4-yl)cycloprop-2-ene-1-carboxylate (20b).

Compound **20a** was prepared following **GP 2**, using Rh_2(*S*-p-Ph-TPCP)$_4$ (3.5 mg, 1 mol%, 2.0 μmol), 2-(2,6-dioxopiperidin-3-yl)-4-ethynylisoindoline-1,3-dione (56 mg, 1.0 equiv, 0.20 mmol), 2,2,2-trichloroethyl 2-(4-bromophenyl)-2-diazoacetate (82 mg, 1.1 equiv, 0.22 mmol), and CH_2Cl_2 (2 mL) at room temperature for two hours. Purification via flash column chromatography (SiO_2, gradient of 30% to 55% EtOAc in hexanes) afforded 2,2,2-trichloroethyl (1*S*)-1-(4-bromophenyl)-2-(2-(2,6-dioxopiperidin-3-yl)-1,3-dioxoisoindolin-4-yl)cycloprop-2-ene-1-carboxylate as an amorphous white solid. (93 mg, 0.15 mmol, 76% yield).
Compound **20b** was prepared in the same manner using Rh_2(*R*-p-Ph-TPCP)$_4$, which afforded 2,2,2-trichloroethyl (1*R*)-1-(4-bromophenyl)-2-(2-(2,6-dioxopiperidin-3-yl)-1,3-dioxoisoindolin-4-yl)cycloprop-2-ene-1-carboxylate as an amorphous white solid. (99 mg, 0.16 mmol, 79% yield).
HRMS (APCI): *m/z* 624.9330 $[(M+H)^+$ requires 624.9330], calc'd for $C_{25}H_{17}O_6N_2{}^{79}Br^{35}Cl_3$
^{1}H NMR (600 MHz, $CDCl_3$, reported as a mixture of diastereomers) δ 8.29 (s, 1H), 7.95 (d, *J* = 7.7 Hz, 1H), 7.74 (t, *J* = 7.6 Hz, 1H), 7.584 and 7.58 (t, *J* = 7.7 Hz, 1H), 7.443 and 7.436 (d, *J* = 8.6 Hz, 2H), 7.39 and 7.38 (s, 1H), 7.29 and 7.28 (d, *J* = 8.6 Hz, 2H), 5.26 and 5.22 (dd, *J* = 13.4, 5.0 Hz, 1H), 4.81 and 4.79 (d, *J* = 12.0 Hz, 1H), 4.761 and 4.760 (d, *J* = 11.9 Hz, 1H), 4.61 and 4.56 (d, *J* = 16.6 Hz, 1H), 4.43 and 4.40 (d, *J* = 16.5 Hz, 1H), 2.97-2.87 (m, 1H), 2.87-2.79 (m, 1H), 2.41-2.29 (m, 1H), 2.27 – 2.11 (m, 1H).
^{13}C{^{1}H} NMR (101 MHz, $CDCl_3$, reported as a mixture of diastereomers) δ 171.8, 170.9, 168.0, 166.59, 166.57, 166.24, 166.22, 138.3, 138.2, 135.8, 135.7, 134.9, 133.1, 131.5, 130.1, 129.91, 129.86, 125.2, 122.70, 122.68, 121.2, 112.48, 112.45, 108.11, 108.06, 95.11, 95.08, 74.49, 74.47, 49.6, 33.92, 33.91, 31.5, 22.7.
SFC analysis: 20a (Chiralcel® OX-3, 30% 1:1 MeOH:^{i}PrOH with 0.2% formic acid in CO_2, 2.5 mL/min, diode array) indicated 80:20 d.r.: t_R (major diastereomers) = 6.92 and 12.88 min, t_R (minor diastereomers) = 4.57 and 4.97 min. **20b** indicated the opposite diastereomers in 81:19 d.r.

Purity (SFC): 20a – 99%; **20b** – 98%

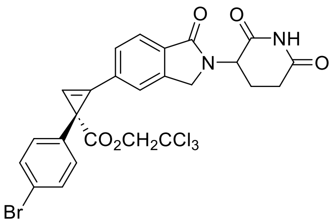

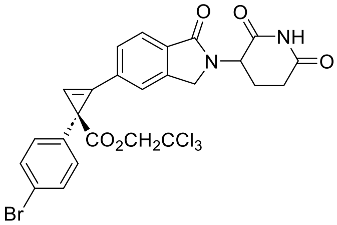

21a **21b**

2,2,2-trichloroethyl (1*S*)-1-(4-bromophenyl)-2-(2-(2,6-dioxopiperidin-3-yl)-1-oxoisoindolin-5-yl)cycloprop-2-ene-1-carboxylate (21a).

2,2,2-trichloroethyl (1*R*)-1-(4-bromophenyl)-2-(2-(2,6-dioxopiperidin-3-yl)-1-oxoisoindolin-5-yl)cycloprop-2-ene-1-carboxylate (21b).

Compound **21a** was prepared following **GP 2**, using Rh_2(*S*-p-Ph-TPCP)$_4$ (3.5 mg, 1 mol%, 2.0 μmol), 3-(5-ethynyl-1-oxoisoindolin-2-yl)piperidine-2,6-dione (54 mg, 1.0 equiv, 0.20 mmol), 2,2,2-trichloroethyl 2-(4-bromophenyl)-2-diazoacetate (82 mg, 1.1 equiv, 0.22 mmol), and CH_2Cl_2 (2 mL) at room temperature for two hours. Purification via flash column chromatography (SiO_2, gradient of 35% to 55% EtOAc in hexanes) afforded 2,2,2-trichloroethyl (*S*)-1-(4-bromophenyl)-2-(2-(2,6-dioxopiperidin-3-yl)-1-oxoisoindolin-5-yl)cycloprop-2-ene-1-carboxylate as an amorphous white solid. (48 mg, 78 μmol, 39% yield).
Compound **21b** was prepared in the same manner using Rh_2(*R*-p-Ph-TPCP)$_4$, which afforded 2,2,2-trichloroethyl (1*R*)-1-(4-bromophenyl)-2-(2-(2,6-dioxopiperidin-3-yl)-1-oxoisoindolin-5-yl)cycloprop-2-ene-1-carboxylate as an amorphous white solid. (46 mg, 74 μmol, 37% yield).
HRMS (APCI): *m/z* 610.9536 $[(M+H)^+]$ requires 610.9537], calc'd for $C_{25}H_{19}O_5N_2{}^{79}Br^{35}Cl_3$
^{1}H NMR (400 MHz, $CDCl_3$, reported as a mixture of diastereomers) δ 8.27 (s, 1H), 7.952 and 7.950 (d, *J* = 7.9 Hz, 1H), 7.79 – 7.71 (m, 1H), 7.69 – 7.64 (m, 1H), 7.43 (d, *J* = 8.5 Hz, 2H), 7.39 (s, 1H), 7.29 (d, *J* = 8.6 Hz, 2H), 5.23 (dd, *J* = 13.3, 5.2 Hz, 1H), 4.823 and 4.816 (d, *J* = 12.0 Hz, 1H), 4.764 and 4.760 (d, *J* = 12.0 Hz, 1H), 4.52 and 4.51 (d, *J* = 16.3 Hz, 1H), 4.37 and 4.35 (d, *J* = 16.3 Hz, 1H), 2.98 – 2.88 (m, 2H), 2.88 – 2.76 (m, 1H), 2.35 (qd, *J* = 13.1, 5.1 Hz, 1H), 2.26 – 2.15 (m, 1H).
^{13}C{^{1}H} NMR (101 MHz, $CDCl_3$, reported as a mixture of diastereomers) δ ^{13}C NMR (101 MHz, $CDCl_3$) δ 172.08, 172.06, 171.1, 169.5, 168.5, 142.1, 138.5, 133.17, 133.15, 131.50, 131.48, 130.32, 130.30, 129.95, 128.4, 125.0, 124.5, 121.13, 121.10, 116.2, 102.2, 102.1, 95.1, 74.42, 74.39, 52.1, 47.1, 33.3, 31.6, 23.5.
SFC analysis: 21a (Chiralpak® AS3, 20% 1:1:1 EtOH:^{i}PrOH:MeCN with 20 mM NH_4HCO_2 in CO_2, 2.5 mL/min, diode array) indicated >99:1 d.r.: t_R (major diastereomers) = 3.94 and 6.41 min, t_R (minor diastereomers) = 3.41 and 4.88 min. **21b** indicated the opposite diastereomers in 99:1 d.r.
Purity (SFC): 21a – 97%; **21b** – 95%

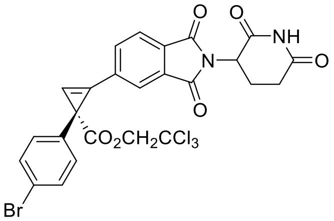

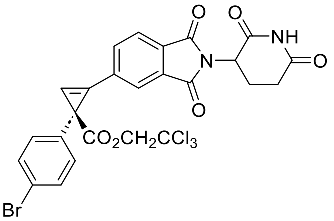

22a **22b**

2,2,2-trichloroethyl (1*S*)-1-(4-bromophenyl)-2-(2-(2,6-dioxopiperidin-3-yl)-1,3-dioxoisoindolin-5-yl)cycloprop-2-ene-1-carboxylate (22a).

2,2,2-trichloroethyl (1*R*)-1-(4-bromophenyl)-2-(2-(2,6-dioxopiperidin-3-yl)-1,3-dioxoisoindolin-5-yl)cycloprop-2-ene-1-carboxylate (22b).

Compound **22a** was prepared following **GP 2**, using Rh_2(*S*-p-Ph-TPCP)$_4$ (3.5 mg, 1 mol%, 2.0 μmol), 2-(2,6-dioxopiperidin-3-yl)-5-ethynylisoindoline-1,3-dione (56 mg, 1.0 equiv, 0.20 mmol), 2,2,2-trichloroethyl 2-(4-bromophenyl)-2-diazoacetate (82 mg, 1.1 equiv, 0.22 mmol), and CH_2Cl_2 (2 mL) at room temperature for two

hours. Purification via flash column chromatography (SiO_2, gradient of 20% to 50% EtOAc in hexanes) afforded 2,2,2-trichloroethyl (1*S*)-1-(4-bromophenyl)-2-(2-(2,6-dioxopiperidin-3-yl)-1,3-dioxoisoindolin-5-yl)cycloprop-2-ene-1-carboxylate as an amorphous white solid. (87 mg, 0.14 mmol, 69% yield).
Compound **22b** was prepared in the same manner using Rh_2(*R*-p-Ph-TPCP)$_4$, which afforded 2,2,2-trichloroethyl (1*R*)-1-(4-bromophenyl)-2-(2-(2,6-dioxopiperidin-3-yl)-1,3-dioxoisoindolin-5-yl)cycloprop-2-ene-1-carboxylate as an amorphous white solid. (88 mg, 0.14 mmol, 70% yield).
HRMS (APCI): m/z 624.9341 [(M+H)$^+$ requires 624.9344], calc'd for $C_{25}H_{16}{}^{79}Br_{35}Cl_3N_2O_6$
^{1}H NMR (500 MHz, $CDCl_3$, reported as a mixture of diastereomers) δ 8.26 (s, 1H), 8.11-8.10 (m, 1H), 7.98-7.94 (m, 2H), 7.54 (s, 1H), 7.45 (d, *J*=8.5 Hz, 2H), 7.28 (d, *J*=8.5 Hz, 2H), 5.01 (dd, *J*=12.5, 5.1 Hz, 1H), 4.84 and 4.83 (d, *J*=12.0 Hz, 1H), 4.76 (d, *J*=12.0 Hz, 1H), 2.94-2.72 (m, 3H), 2.19-2.14 (m, 1H).
^{13}C{^{1}H} NMR (126 MHz, $CDCl_3$, reported as a mixture of diastereomers) δ 171.5, 171.0, 167.9, 166.5, 166.4, 137.9, 135.7, 132.7, 132.6, 131.6, 131.2, 129.90, 129.89, 124.89, 124.88, 124.6, 121.4, 116.1, 116.0, 104.5, 95.0, 74.5, 60.5, 49.7, 33.7, 31.48, 31.46, 22.7, 22.6, 14.3.
SFC analysis: 22a (Chiralcel® OJ-3, 20% 1:1 MeOH:^{i}PrOH with 0.2% formic acid in CO_2, 2.5 mL/min, diode array) indicated 98:2 d.r.: t_R (major diastereomers) = 7.10 and 8.24 min, t_R (minor diastereomers) = 9.20 and 11.52 min. **22b** indicated the opposite diastereomers in 99:1 d.r.
Purity (SFC): 22a – 98%; **22b** – 95%

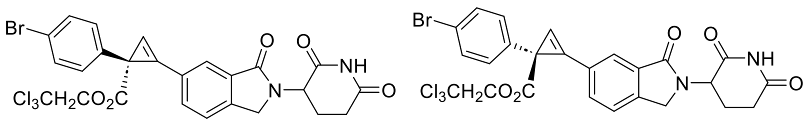

23a **23b**

2,2,2-trichloroethyl (1*S*)-1-(4-bromophenyl)-2-(2-(2,6-dioxopiperidin-3-yl)-3-oxoisoindolin-5-yl)cycloprop-2-ene-1-carboxylate (23a).

2,2,2-trichloroethyl (1*R*)-1-(4-bromophenyl)-2-(2-(2,6-dioxopiperidin-3-yl)-3-oxoisoindolin-5-yl)cycloprop-2-ene-1-carboxylate (23b).

Compound **23a** was prepared following **GP 2**, using Rh_2(*S*-p-Ph-TPCP)$_4$ (3.5 mg, 1 mol%, 2.0 μmol), 3-(6-ethynyl-1-oxoisoindolin-2-yl)piperidine-2,6-dione (54 mg, 1.0 equiv, 0.20 mmol), 2,2,2-trichloroethyl 2-(4-bromophenyl)-2-diazoacetate (82 mg, 1.1 equiv, 0.22 mmol), and CH_2Cl_2 (2 mL) at room temperature for two hours. Purification via flash column chromatography (SiO_2, gradient of 60% to 75% EtOAc in hexanes) afforded 2,2,2-trichloroethyl (1*S*)-1-(4-bromophenyl)-2-(2-(2,6-dioxopiperidin-3-yl)-3-oxoisoindolin-5-yl)cycloprop-2-ene-1-carboxylate as an amorphous white solid. (76 mg, 12 mmol, 62% yield).
Compound **23b** was prepared in the same manner using Rh_2(*R*-p-Ph-TPCP)$_4$, which afforded 2,2,2-trichloroethyl (1*R*)-1-(4-bromophenyl)-2-(2-(2,6-dioxopiperidin-3-yl)-3-oxoisoindolin-5-yl)cycloprop-2-ene-1-carboxylate as an amorphous white solid. (77 mg, 0.13 mmol, 63% yield).
HRMS (APCI): *m/z* 610.9540 [(M+H)$^+$ requires 610.9537], calc'd for $C_{25}H_{19}O_5N_2{}^{79}Br^{35}Cl_3$
^{1}H NMR (600 MHz, $CDCl_3$, reported as a mixture of diastereomers) δ 8.17 (s, 1H), 8.13 (d, *J* = 6.2 Hz, 1H), 7.81 (t, *J* = 7.9 Hz, 1H), 7.54 (d, *J* = 7.9 Hz, 1H), 7.42 (d, *J* = 8.6 Hz, 2H), 7.32 (s, 1H), 7.31 (d, *J* = 8.4 Hz, 2H), 5.29 – 5.17 (m, 1H), 4.82 and 4.81 (d, *J* = 11.9 Hz, 1H), 4.76 and 4.75 Hz (d, *J* = 11.9 Hz, 1H), 4.54 (d, *J* = 16.7 Hz, 1H), 4.39 (d, *J* = 16.5 Hz, 1H), 2.97 – 2.90 (m, 1H), 2.88 – 2.77 (m, 1H), 2.41 – 2.30 (m, 1H), 2.25 – 2.18 (m, 1H).
^{13}C{^{1}H} NMR (101 MHz, $CDCl_3$, reported as a mixture of diastereomers) δ 172.2, 169.5, 171.3, 168.5, 143.3, 138.6, 133.61, 133.57, 132.54, 132.52, 131.4, 130.1, 125.65, 125.61, 125.4, 123.9, 121.02, 121.00, 116.3, 116.2, 100.7, 95.1, 74.4, 52.03, 52.00, 47.3, 33.3, 31.6, 23.5.
SFC analysis: 23a (Chiralcel® OJ-3, 30% 1:1 MeOH:^{i}PrOH with 0.2% formic acid in CO_2, 2.5 mL/min, diode array) indicated 98:2 d.r.: t_R (major diastereomers) = 1.81 and 2.40 min, t_R (minor diastereomers) = 2.12 and 3.16 min. **23b** indicated the opposite diastereomers in 98:2 d.r.
Purity (SFC): 23a – 99%; **23b** – 99%

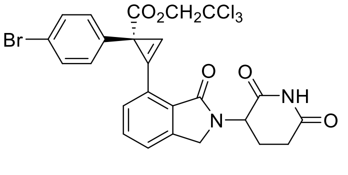

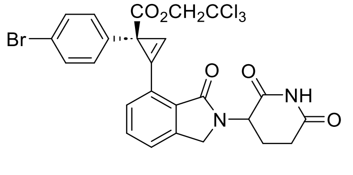

24a **24b**

2,2,2-trichloroethyl (1*S*)-1-(4-bromophenyl)-2-(2-(2,6-dioxopiperidin-3-yl)-3-oxoisoindolin-4-yl)cycloprop-2-ene-1-carboxylate (24a).

2,2,2-trichloroethyl (1*R*)-1-(4-bromophenyl)-2-(2-(2,6-dioxopiperidin-3-yl)-3-oxoisoindolin-4-yl)cycloprop-2-ene-1-carboxylate (24b).

Compound **24a** was prepared following **GP 2**, using Rh_2(*S*-p-Ph-TPCP)$_4$ (3.5 mg, 1 mol%, 2.0 μmol), 3-(7-ethynyl-1-oxoisoindolin-2-yl)piperidine-2,6-dione (54 mg, 1.0 equiv, 0.20 mmol), 2,2,2-trichloroethyl 2-(4-bromophenyl)-2-diazoacetate (82 mg, 1.1 equiv, 0.22 mmol), and CH_2Cl_2 (2 mL) at room temperature for two hours. Purification via flash column chromatography (SiO_2, gradient of 45% to 70% EtOAc in hexanes) afforded 2,2,2-trichloroethyl (1*S*)-1-(4-bromophenyl)-2-(2-(2,6-dioxopiperidin-3-yl)-3-oxoisoindolin-4-yl)cycloprop-2-ene-1-carboxylate as an amorphous white solid. (99 mg, 0.16 mmol, 81% yield).
Compound **24b** was prepared in the same manner using Rh_2(*R*-p-Ph-TPCP)$_4$, which afforded 2,2,2-trichloroethyl (1*R*)-1-(4-bromophenyl)-2-(2-(2,6-dioxopiperidin-3-yl)-3-oxoisoindolin-4-yl)cycloprop-2-ene-1-carboxylate as an amorphous white solid. (97 mg, 0.16 mmol, 79% yield).
HRMS (APCI): *m/z* 610.9545 $[(M+H)^+$ requires 610.9537], calc'd for $C_{25}H_{19}O_5N_2{}^{79}Br^{35}Cl_3$
^{1}H NMR (600 MHz, $CDCl_3$) δ 8.17 (s, 1H), 7.66 (d, *J* = 3.6 Hz, 1H), 7.63 – 7.54 (m, 2H), 7.51 and 7.50 (s, 1H), 7.41 and 7.40 (d, *J* = 8.6 Hz, 2H), 7.34 and 7.33 (d, *J* = 8.6 Hz, 2H), 5.25 and 5.23 (dd, *J* = 13.0, 5.1 Hz, 1H), 4.85 and 4.83 (d, *J* = 11.9 Hz, 1H), 4.74 and 4.73 (d, *J* = 12.0 Hz, 1H), 2.95-2.89 (m, 1H), 2.89-2.84 (m, 1H), 2.43-2.33 (m, 1H), 2.29-2.14 (m, 1H).
^{13}C{^{1}H} NMR (101 MHz, $CDCl_3$, reported as a mixture of diastereomers) δ 172.5, 172.4, 171.3, 169.7, 168.4, 143.1, 139.1, 139.0, 132.2, 131.29, 131.28, 130.45, 130.43, 130.37, 130.26, 130.24, 124.8, 122.5, 120.80, 120.77, 112.7, 112.5, 106.0, 105.9, 95.3, 74.4,74.3, 52.1, 52.0, 47.1, 47.0, 33.27, 33.26, 31.7, 23.5.
SFC analysis: **24a** (Chiralpak® AS3, 20% 1:1 MeOH:^{i}PrOH with 0.2% formic acid in CO_2, 2.5 mL/min, diode array) indicated 61:39 d.r.: t_R (major diastereomers) = 4.05 and 5.21 min, t_R (minor diastereomers) = 4.49 and 7.67 min. **24b** indicated the opposite diastereomers in 60:40 d.r.
Purity (SFC): 24a – 95%; **24b** – 95%

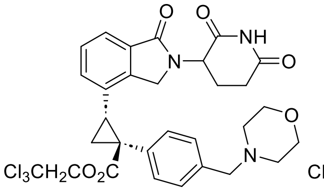

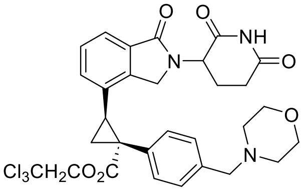

25a **25b**

2,2,2-trichloroethyl (1*S*,2*R*)-2-(2-(2,6-dioxopiperidin-3-yl)-1-oxoisoindolin-4-yl)-1-(4-(morpholinomethyl)phenyl)cyclopropane-1-carboxylate (25a).

2,2,2-trichloroethyl (1*R*,2*S*)-2-(2-(2,6-dioxopiperidin-3-yl)-1-oxoisoindolin-4-yl)-1-(4-(morpholinomethyl)phenyl)cyclopropane-1-carboxylate (25b).

A 10 mL round-bottom flask was charged with a PTFE magnetic stir bar and ca. 200% w/w 4 Å molecular sieves, then flame dried under vacuum. The flask was then backfilled with dry nitrogen. Rh_2(*S-tetra-p-*BrPPTTL)$_4$ (3.7 mg, 1 mol%, 1.0 μmol) and 3-(1-oxo-4-vinylisoindolin-2-yl)piperidine-2,6-dione (27 mg, 1.0 equiv, 0.10 mmol) were charged to the flask, and the flask was evacuated and backfilled with nitrogen. The flask was equipped with an argon balloon and rubber septum, and dry CH_2Cl_2 (0.5 mL) along with 0.10 mL 1,1,1,3,3,3-hexafluoroisopropanol (10 equiv, 1.0 mmol) were charged via syringe. A solution of 2,2,2-trichloroethyl 2-diazo-2-(4-(morpholinomethyl)phenyl)acetate (43 mg, 1.1 equiv, 0.11 mmol) in dry CH_2Cl_2 (0.5 mL, prepared in the same manner under inert atmosphere) was added dropwise to the stirred reaction at

room temperature. The reaction was allowed to stir for two hours, after which the reaction was filtered through Celite®, rinsing the filter pad with CH_2Cl_2. The crude residue was dry-mounted onto silica gel and was purified by flash column chromatography (SiO_2, 0-5% methanol in CH_2Cl_2), which afforded 2,2,2-trichloroethyl (1*S*,2*R*)-2-(2-(2,6-dioxopiperidin-3-yl)-1-oxoisoindolin-4-yl)-1-(4-(morpholinomethyl)phenyl)cyclopropane-1-carboxylate as an amorphous white solid (50 mg, 79 μmol, 79% yield).

Compound **25b** was prepared in the same manner using Rh_2(*R*-*tetra*-*p*-BrPPTTL)$_4$, which afforded 2,2,2-trichloroethyl (1*R*,2*S*)-2-(2-(2,6-dioxopiperidin-3-yl)-1-oxoisoindolin-4-yl)-1-(4-(morpholinomethyl)phenyl)cyclopropane-1-carboxylate as an amorphous white solid as an amorphous white solid. (49 mg, 77 μmol, 77% yield).

HRMS (APCI): *m/z* 634.1279 ($[M+H]^+$ requires 634.1273), calc'd for $C_{30}H_{31}O_6N_3{}^{35}Cl_3$

^{1}H NMR (400 MHz, $CDCl_3$, reported as a mixture of diastereomers) δ 8.54 (s, 1H), 7.595 and 7.593 (d, *J* = 7.6 Hz, 1H), 7.10 – 7.00 (m, 3H), 6.96 and 6.93 (d, *J* = 12.3 Hz, 2H), 5.29 and 5.25 (dd, *J* = 9.6, 5.2 Hz, 1H), 4.86 and 4.85 (d, *J* = 12.0 Hz, 1H), 4.664 and 4.658 (d, *J* = 12.0 Hz, 1H), 4.58 and 4.56 (d, *J* = 16.0 Hz, 1H), 4.42 and 4.38 (d, *J* = 16.2 Hz, 1H), 3.69 – 3.58 (m, 4H), 3.43 – 3.36 (m, 2H), 3.07 (ddd, *J* = 9.4, 7.4, 2.1 Hz, 1H), 3.01 – 2.77 (m, 2H), 2.42 – 2.19 (m, 7H), 2.15 (ddd, *J* = 7.5, 5.3, 2.2 Hz, 1H).

^{13}C{^{1}H} NMR (101 MHz, $CDCl_3$, reported as a mixture of diastereomers) δ 171.9, 171.8, 171.29, 171.27, 169.7, 169.53, 169.48, 141.7, 141.6, 137.3, 137.2, 132.0, 131.9, 131.5, 131.4, 131.30, 131.27, 131.19, 131.18, 129.12, 129.06, 129.05, 128.0, 122.6, 95.3, 95.2, 74.4, 74.3, 67.0, 62.9, 53.50, 53.46, 52.03, 51.96, 46.61, 46.50, 37.1, 37.0, 31.7, 29.8, 29.7, 29.6, 23.63, 23.60, 19.7, 19.6.

SFC analysis: 25a (Trefoil® CEL2, 35% 1:1 MeOH:^{i}PrOH with 0.2% formic acid in CO_2, 2.5 mL/min, diode array) indicated 83:17 d.r.: t_R (major diastereomers) = 6.04 and 8.83 min, t_R (minor diastereomers) = 6.80 and 13.50 min. **25b** indicated the opposite diastereomers in 85:15 d.r.

Purity (SFC): 25a – 99%; **25b** – 99%

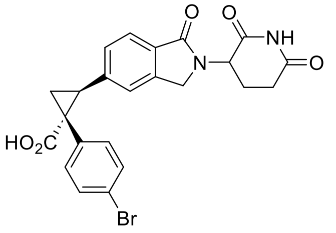

26a

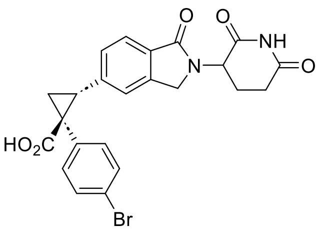

26b

(1*R*,2*S*)-1-(4-bromophenyl)-2-(2-(2,6-dioxopiperidin-3-yl)-1-oxoisoindolin-5-yl)cyclopropane-1-carboxylic acid (26a).

(1*S*,2*R*)-1-(4-bromophenyl)-2-(2-(2,6-dioxopiperidin-3-yl)-1-oxoisoindolin-5-yl)cyclopropane-1-carboxylic acid (26b).

A 100 mL round-bottom flask was charged with a PTFE magnetic stir bar and zinc dust (754 mg, 16 equiv, 11.5 mmol) under ambient conditions, followed by 2,2,2-trichloroethyl (1*S*,2*R*)-1-(4-bromophenyl)-2-(2-(2,6-dioxopiperidin-3-yl)-1-oxoisoindolin-5-yl)cyclopropane-1-carboxylate (443 mg, 1.0 equiv, 0.72 mmol). Tetrahydrofuran (16.6 mL) was charged via syringe followed by acetate buffer (10 mL, pH = 3.7, 1.2 M in H_2O, NaOAc/AcOH). The reaction was stirred at room temperature for 18 hours, after which the reaction was filtered through Celite®, rinsing with ethyl acetate. The aqueous layer was extracted thrice with 50 mL portions of ethyl acetate, after which the combined organics were washed with 150 mL brine. The combined organics were dried over magnesium sulfate, then filtered and concentrated in vacuuo. The crude material was purified via flash column chromatography (C18, 10-95% MeCN in H_2O, 0.1% trifluoroacetic acid buffer), which gave (1*R*,2*S*)-1-(4-bromophenyl)-2-(2-(2,6-dioxopiperidin-3-yl)-1-oxoisoindolin-5-yl)cyclopropane-1-carboxylic acid as an amorphous white solid (280 mg, 0.58 mmol, 83% yield).

Compound **26b** was prepared in the same manner using zinc dust (727 mg, 16 equiv, 11.1 mmol), 2,2,2-trichloroethyl (1*R*,2*S*)-1-(4-bromophenyl)-2-(2-(2,6-dioxopiperidin-3-yl)-1-oxoisoindolin-5-yl)cyclopropane-

1-carboxylate (427 mg, 1.0 equiv, 0.70 mmol), tetrahydrofuran (16.0 mL), and acetate buffer (9.6 mL), which gave (1*S*,2*R*)-1-(4-bromophenyl)-2-(2-(2,6-dioxopiperidin-3-yl)-1-oxoisoindolin-5-yl)cyclopropane-1-carboxylic acid (286 mg, 0.59 mmol, 82% yield) as an amorphous white solid.

HRMS (APCI): *m/z* 483.0559 ($[M+H]^+$ requires 483.0550), calc'd for $C_{23}H_{20}O_5N_2{}^{79}Br$
^{1}H NMR (600 MHz, CD_3OD, reported as a mixture of diastereomers) δ 7.55 – 7.42 (m, 1H), 7.26 (d, *J* = 8.1 Hz, 2H), 7.14 and 7.10 (s, 1H), 7.05-6.98 (m, 1H, partially obscured by signal at 7.00), 7.00 (d, *J* = 8.0 Hz, 3H), 5.10 and 5.07 (dd, *J* = 10.9, 5.2 Hz, 1H), 4.39 and 4.34 (d, *J* = 17.0 Hz, 1H), 4.27 and 4.26 (d, *J* = 17.0 Hz, 1H), 3.27 (t, *J* = 8.2 Hz, 1H), 2.87 (ddd, *J* = 18.5, 13.6, 5.4 Hz, 1H), 2.81 – 2.70 (m, 1H), 2.43 (pd, *J* = 13.5, 4.5 Hz, 1H), 2.17-2.08 (m, 3H).

^{13}C NMR (101 MHz, CD_3OD) δ 176.2, 174.7, 172.22, 172.20, 171.19, 171.17, 143.5, 143.01, 143.29, 135.7, 135.6, 135.0, 134.9, 131.9, 131.0, 130.9, 129.7, 129.3, 124.3, 124.0, 123.73, 123.71, 122.0, 53.6, 53.5, 38.6, 38.5, 34.0, 32.3, 24.0, 20.5, 20.4.

SFC analysis: 26a (Chiralpak® AS3, 20% 1:1 EtOH:^{i}PrOH with 0.2% formic acid in CO_2, 2.5 mL/min, diode array) indicated 99:1 d.r.: t_R (major diastereomers) = 4.56 and 6.30 min, t_R (minor diastereomers) = 5.73 and 8.81 min. **26b** indicated the opposite diastereomers in 99:1 d.r.
Purity (SFC): 26a – 98%; **26b** – 98%

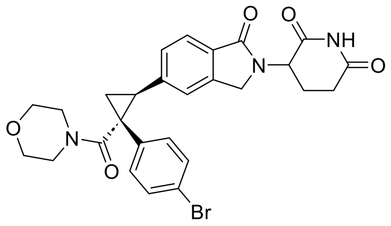

27a

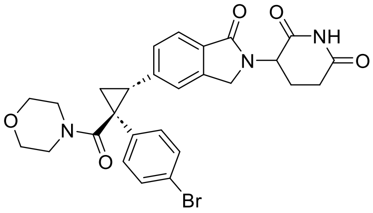

27b

3-(5-((1*S*,2*R*)-2-(4-bromophenyl)-2-(morpholine-4-carbonyl)cyclopropyl)-1-oxoisoindolin-2-yl)piperidine-2,6-dione (27a).

3-(5-((1*R*,2*S*)-2-(4-bromophenyl)-2-(morpholine-4-carbonyl)cyclopropyl)-1-oxoisoindolin-2-yl)piperidine-2,6-dione (27b).

A 4 mL scintillation vial equipped with a PTFE magnkjetic stir bar was flame-dried under vacuum and backfilled with dry nitrogen. The vial was charged with (1*S*,2*R*)-1-(4-bromophenyl)-2-(2-(2,6-dioxopiperidin-3-yl)-1-oxoisoindolin-5-yl)cyclopropane-1-carboxylic acid (50 mg, 1.0 equiv, 0.10 mmol) and N-(chloro(dimethylamino)methylene)-N-methylmethanaminium hexafluorophosphate(V) (34 mg, 1.2 equiv, 0.12 mmol). Morpholine (11 μL, 1.3 equiv, 0.13 mmol), 1-methyl-1H-imidazole (28 μL, 3.5 equiv, 0.35 mmol), and acetonitrile (0.25 mL) were charged successively by syringe, and the reaction was stirred at room temperature for 18 hours. The reaction was stirred at room temperature for 18 hours, after which the reaction was partitioned between water (50 mL) and ethyl acetate (50 mL). The aqueous layer was extracted thrice with 50 mL portions of ethyl acetate, after which the combined organics were washed with 150 mL of brine. The combined organics were dried over magnesium sulfate, then filtered and concentrated in vacuuo. The crude material was purified via flash column chromatography (SiO_2, 0-10% methanol in CH_2Cl_2), which gave 3-(5-((1*S*,2*R*)-2-(4-bromophenyl)-2-(morpholine-4-carbonyl)cyclopropyl)-1-oxoisoindolin-2-yl)piperidine-2,6-dione as an amorphous white solid (31 mg, 55 μmol, 55% yield).

Compound **27b** was prepared in the same manner from (1*S*,2*R*)-1-(4-bromophenyl)-2-(2-(2,6-dioxopiperidin-3-yl)-1-oxoisoindolin-5-yl)cyclopropane-1-carboxylic acid, which gave 3-(5-((1*R*,2*S*)-2-(4-bromophenyl)-2-(morpholine-4-carbonyl)cyclopropyl)-1-oxoisoindolin-2-yl)piperidine-2,6-dione as an amorphous white solid (34 mg, 60 μmol, 60% yield).

HRMS (APCI): *m/z* 552.1139 ($[M+H]^-$ requires 552.1129), calc'd for $C_{27}H_{27}O_5N_3{}^{79}Br$
^{1}H NMR (600 MHz, $CDCl_3$, reported as a mixture of diastereomers) δ 7.99 and 7.98 (s, 1H), 7.60 (d, *J* and 7.58 (d, *J* = 5.9 Hz, 1H), 7.29 – 7.26 (m, 2H, partially obscured by solvent signal), 7.17 and 7.10 (s, 1H), 7.07

and 7.02 (dd, J = 7.9, 1.4 Hz, 1H), 6.99 – 6.92 (m, 2H), 5.160 and 5.156 (dd, J = 13.3, 5.1 Hz, 1H), 4.343 and 4.338 (d, J = 15.9 Hz, 1H), 4.17 and 4.16 (d, J = 15.9 Hz, 1H), 3.74 – 3.37 (m, 7H), 3.30 (dt, J = 9.2, 6.9 Hz, 1H), 3.18 (s, 1H), 2.92 – 2.87 (m, 1H), 2.80 (ddd, J = 18.1, 13.4, 5.4 Hz, 1H), 2.37 – 2.25 (m, 1H), 2.23 – 2.13 (m, 1H), 2.08 (ddd, J = 7.0, 5.8, 3.2 Hz, 1H), 1.68 (ddd, J = 9.1, 5.8, 4.5 Hz, 1H).
^{13}C NMR (101 MHz, $CDCl_3$) δ 171.74, 171.72, 170.27, 170.23, 170.0, 169.1, 141.4, 141.25, 141.21, 134.26, 134.21, 131.74, 131.71, 129.87,129.7, 128.6, 128.3, 123.8, 123.51, 123.48, 123.2, 121.2, 121.1, 66.4, 51.8, 51.7, 46.80, 46.76, 38.1, 37.9, 31.6, 30.2, 30.1, 23.4, 16.1, 16.0.
SFC analysis: 27a (Chiralcel® OJ-3, 20% 1:1 MeOH:^{i}PrOH with 0.2% formic acid in CO_2, 2.5 mL/min, diode array) indicated 99:1 d.r.: t_R (major diastereomers) = 2.80 and 3.65 min, t_R (minor diastereomers) = 3.21 and 4.77 min. **27b** indicated the opposite diastereomers in 99:1 d.r.
Purity (SFC): 27a – 98%; **27b** – 98%

Section 3: X-ray Crystallographic Data for *R*-15a

Submitted by: **William Tracy, Huw Davies Lab**

Solved by: **John Bacsa, Mackenzie Young**

R_1=5.45%

Crystal Data and Experimental

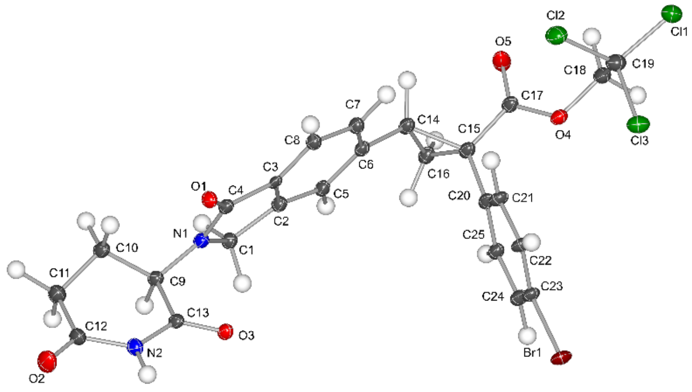

Experimental. Single colourless plate-shaped crystals of WT-05-553 were crystallized from methanol by slow evaporation. A suitable crystal with dimensions 0.13 × 0.10 × 0.05 mm^3 was selected and mounted on a loop with paratone on a XtaLAB Synergy, Dualflex, HyPix diffractometer. The crystal was kept at a steady T = 104(6) K during data collection. The structure was solved with the ShelXT (Sheldrick, 2015) solution program using dual methods and by using Olex2 1.5-alpha (Dolomanov et al., 2009) as the graphical interface. The model was refined with olex2.refine 1.5-alpha (Bourhis et al., 2015) using full matrix least squares minimisation on $\boldsymbol{F^2}$.

Crystal Data. $C_{25}H_{20}BrCl_3N_2O_5$, M_r = 614.709, monoclinic, $P2_1$ (No. 4), a = 6.1901(4) Å, b = 9.2069(6) Å, c = 22.1261(17) Å, β = 90.543(7)°, α = γ = 90°, V = 1260.94(16) Å^3, T = 104(6) K, Z = 2, Z' = 1, μ(Mo K$_\alpha$) = 1.988, 15376 reflections measured, 5610 unique (R_{int} = 0.0782) which were used in all calculations. The final wR_2 was 0.1153 (all data) and R_1 was 0.0545 (I≥2 σ(I)).

**Compound**	WT-05-553
Formula	$C_{25}H_{20}BrCl_3N_2O_5$
$D_{calc.}$/ g cm^{-3}	1.619
μ/mm^{-1}	1.988
Formula Weight	614.709
Color	colorless
Shape	plate-shaped
Size/mm^3	0.13×0.10×0.05
T/K	104(6)
Crystal System	monoclinic
Flack Parameter	-0.006(8)
Hooft Parameter	-0.006(8)
Space Group	$P2_1$
a/Å	6.1901(4)
b/Å	9.2069(6)
c/Å	22.1261(17)
α/°	90
β/°	90.543(7)
γ/°	90
V/Å^3	1260.94(16)
Z	2
Z'	1
Wavelength/Å	0.71073
Radiation type	Mo K_α
Θ_{min}/°	3.41
Θ_{max}/°	27.57
Measured Refl's.	15376
Indep't Refl's	5610
Refl's I≥2 σ(I)	4416
R_{int}	0.0782
Parameters	451
Restraints	823
Largest Peak	1.5961
Deepest Hole	-0.6735
GooF	1.0057
wR_2 (all data)	0.1153
wR_2	0.1076
R_1 (all data)	0.0786
R_1	0.0545

Structure Quality Indicators

**Reflections:**	d min (MoKα) 2Θ=55.1° 0.77	I/σ(I) 10.2	Rint m=2.69 7.82%	Full 50.5° 95.6	
**Refinement:**	Shift -0.001	Max Peak 1.6	Min Peak -0.7	GooF 1.006	Hooft -.006(8)

A colorless plate-shaped-shaped crystal with dimensions 0.13 × 0.10 × 0.05 mm^3 was mounted on a loop with paratone. Data were collected using a XtaLAB Synergy, Dualflex, HyPix diffractometer equipped with an Oxford Cryosystems low-temperature device operating at T = 104(6) K.

Data were measured using ω scans with Mo K_α radiation. The diffraction pattern was indexed and the total number of runs and images was based on the strategy calculation from the program CrysAlisPro system (CCD 43.92a 64-bit (release 05-10-2023)). The maximum resolution that was achieved was Θ = 27.57° (0.77 Å).

The unit cell was refined using CrysAlisPro 1.171.43.121a (Rigaku OD, 2024) on 3358 reflections, 22% of the observed reflections.

Data reduction, scaling and absorption corrections were performed using CrysAlisPro 1.171.43.121a (Rigaku OD, 2024). The final completeness is 98.65 % out to 27.57° in Θ. An analytical numeric absorption correction using a multifaceted crystal model based on expressions derived by R.C. Clark & J.S. Reid. (Clark, R. C. & Reid, J. S. (1995). Acta Cryst. A51, 887-897) was performed using CrysAlisPro 1.171.43.121a (Rigaku Oxford Diffraction, 2024). An empirical absorption correction using spherical harmonics, implemented in SCALE3 ABSPACK scaling algorithm was also applied. The absorption coefficient μ of this material is 1.988 mm^{-1} at this wavelength (λ = 0.71073Å) and the minimum and maximum transmissions are 0.821 and 0.924.

The structure was solved and the space group $P2_1$ (# 4) determined by the ShelXT (Sheldrick, 2015) structure solution program using using dual methods and refined by full matrix least squares minimisation on $\boldsymbol{F}^2$ using version of olex2.refine 1.5-alpha (Bourhis et al., 2015). All atoms, even hydrogen atoms, were refined anisotropically. Hydrogen atom positions were located from the electron densities and freely refined using Hirshfeld scattering factors. Refinement was by using NoSpherA2, an implementation of non-spherical atom-form-factors (F. Kleemiss, H. Puschmann, O. Dolomanov, S.Grabowsky - https://doi.org/10.1039/D0SC05526C – 2020). NoSpherA2 implementation of HAR makes use of tailor-made aspherical atomic form factors calculated from a Hirshfeld-partitioned electron density (ED) not from spherical-atom form factors. The ED was calculated from a Gaussian basis set single determinant SCF wavefunction from DFT using selected functionals for a fragment of this crystal. This fragment was embedded in an electrostatic crystal field by employing cluster charges. The following options were used: software: SOFTWARE: ORCA PARTITIONING: NoSpherA2 INT ACCURACY: Normal METHOD: PBE BASIS SET: x2c-SVP CHARGE: 0 MULTIPLICITY: 1 SOLVATION: Methanol RELATIVISTIC: DKH2 DATE: 2024-05-14_13-44-20

There is a single formula unit in the asymmetric unit, which is represented by the reported sum formula. In other words: Z is 2 and Z' is 1. The moiety formula is C25 H20 Br Cl3 N2 O5.

The Flack parameter was refined to -0.006(8). Determination of absolute structure using Bayesian statistics on Bijvoet differences using the Olex2 results in -0.006(8). The chiral atoms in this structure are: C9(R), C14(S), C15(R). Note: The Flack parameter is used to determine chirality of the crystal studied, the value should be near 0, a value of 1 means that the stereochemistry is wrong and the model should be inverted. A value of 0.5 means that the crystal consists of a racemic mixture of the two enantiomers.

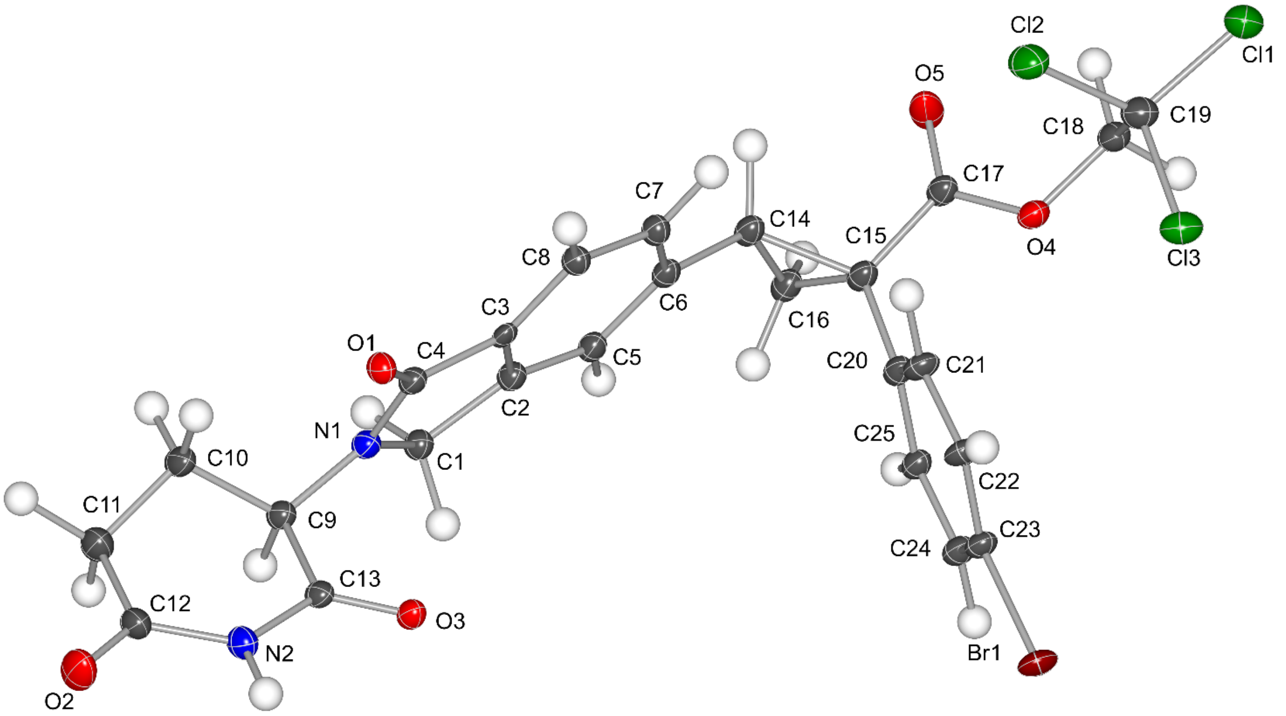

Figure 1 A thermal ellipsoidal representation of the asymmetric unit in the crystal structure (50% probability) which consists of one whole molecule. The chiral atoms in this structure are: C9(R), C14(S), and C15(R).

Data Plots: Diffraction Data

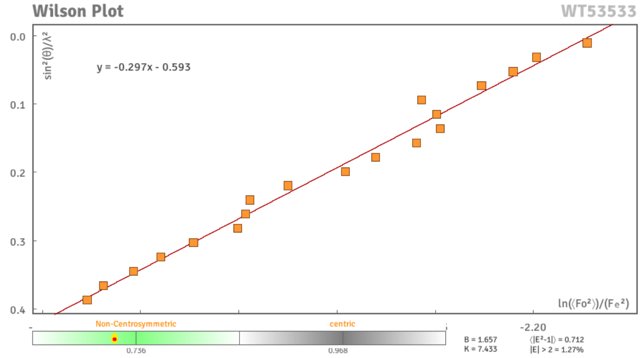

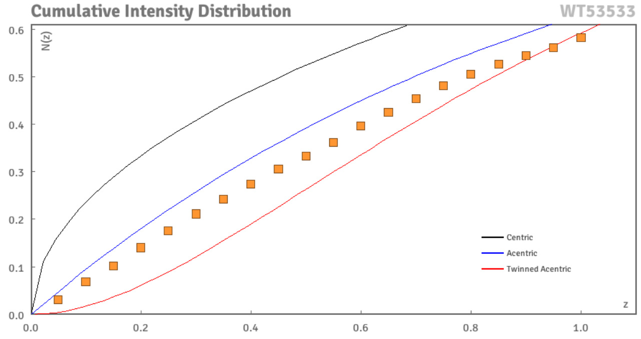

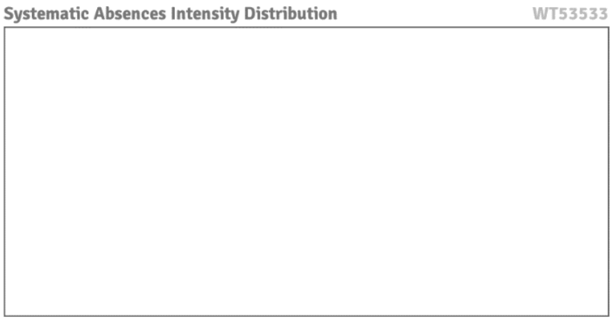

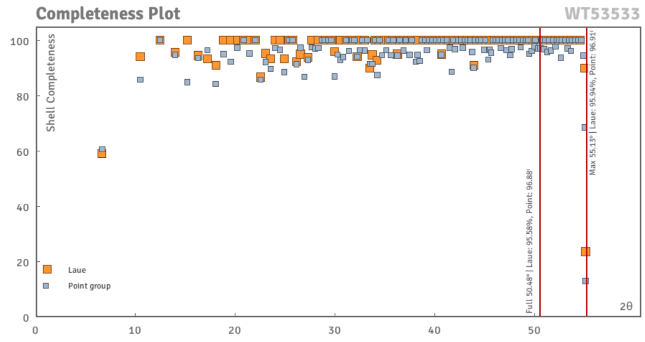

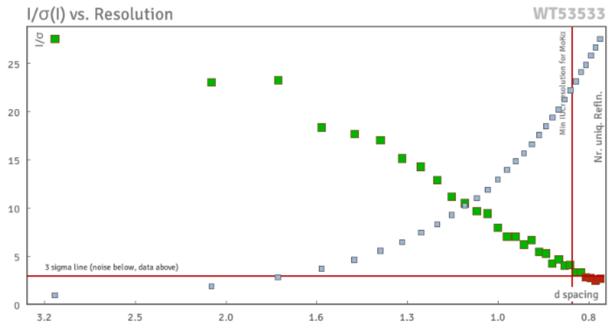

Data Plots: Refinement and Data

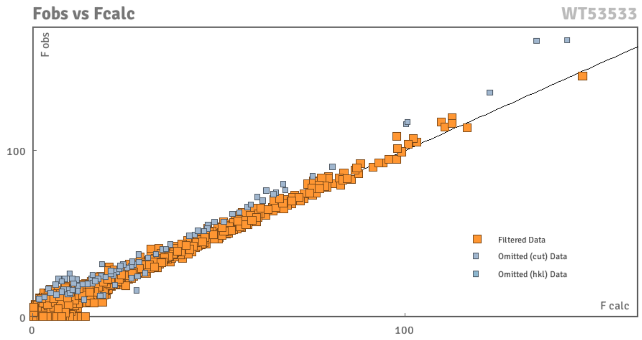

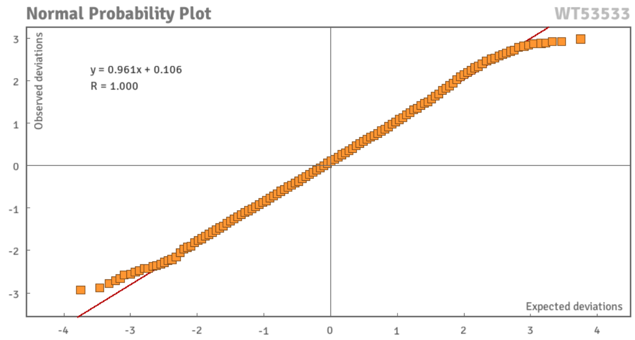

Reflection Statistics

Total reflections (after filtering)	15067	Unique reflections	5610
Completeness	0.969	Mean I/σ	9.61
hkl_{max} collected	(8, 11, 28)	hkl_{min} collected	(-8, -11, -28)
hkl_{max} used	(8, 11, 28)	hkl_{min} used	(-8, -11, 0)
Lim d_{max} collected	100.0	Lim d_{min} collected	0.36
d_{max} used	5.98	d_{min} used	0.77
Friedel pairs	3967	Friedel pairs merged	0
Inconsistent equivalents	5	R_{int}	0.079
R_{sigma}	0.0982	Intensity transformed	0
Omitted reflections	309	Omitted by user (OMIT hkl)	3
Multiplicity	(5283, 3149, 948, 182, 41, 3)	Maximum multiplicity	9
Removed systematic absences	0	Filtered off (Shel/OMIT)	0

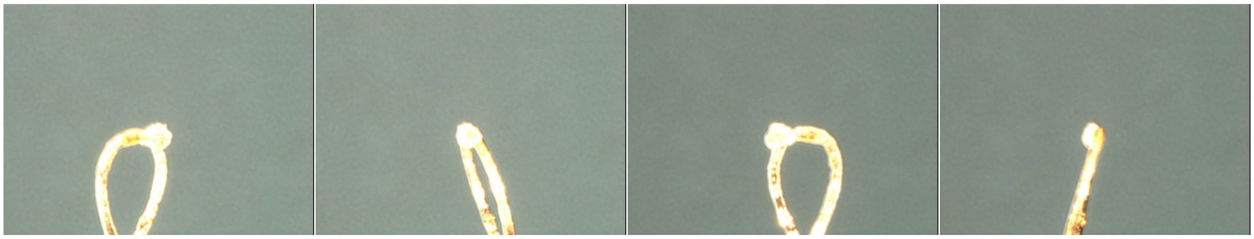

Table 1: Fractional Atomic Coordinates (×10[4]) and Equivalent Isotropic Displacement Parameters (Å[2]×10[3]) for WT-05-553. U_{eq} is defined as 1/3 of the trace of the orthogonalised U_{ij}.

Atom	x	y	z	U_{eq}
Br1	5371.5(7)	1137.5(3)	1682.0(2)	21.17(12)
Cl3	3100.3(17)	5997.6(11)	86.8(5)	26.1(3)
Cl1	761(2)	8186.8(11)	-588.1(6)	28.2(3)
Cl2	2633(2)	8864.4(11)	589.3(6)	29.2(3)
O1	7753(5)	8378(3)	4542.3(14)	17.2(6)
O2	10793(5)	6114(5)	6695.3(14)	32.8(8)
O3	7875(5)	4979(3)	4887.5(14)	17.5(7)
O4	-410(5)	6480(3)	1003.0(14)	19.3(5)
O5	-2542(6)	8132(4)	1452.0(16)	33.7(9)
N1	4848(6)	7116(4)	4900.2(18)	16.2(6)
N2	9217(6)	5538(4)	5810.9(18)	18.4(8)
C1	2699(7)	6635(5)	4700(2)	16.9(7)
C2	2630(7)	7101(4)	4056(2)	16.0(7)
C3	4518(7)	7884(4)	3935(2)	13.4(7)
C4	5927(7)	7865(4)	4472(2)	15.2(7)
C5	1035(7)	6939(4)	3619(2)	16.3(8)
C6	1334(7)	7600(5)	3054(2)	15.8(6)
C7	3204(7)	8426(4)	2945(2)	16.9(8)
C8	4842(7)	8574(4)	3376(2)	16.2(8)
C9	5785(7)	6673(4)	5473(2)	17.0(6)
C10	6482(8)	7933(5)	5882(2)	22.2(9)
C11	7441(9)	7325(5)	6465(2)	25.4(9)
C12	9287(7)	6297(5)	6349(2)	21.8(9)
C13	7694(7)	5653(4)	5353(2)	15.7(8)
C14	-410(8)	7585(5)	2582(2)	17.9(5)
C15	-528(7)	6447(4)	2067(2)	17.8(4)
C16	-2184(7)	6491(4)	2567(2)	19.1(5)
C17	-1285(7)	7124(5)	1485(2)	18.5(6)
C18	-833(8)	7092(5)	422(2)	21.0(5)
C19	1315(9)	7524(5)	144(2)	23.8(4)
C20	963(7)	5191(5)	2009(2)	16.9(5)
C21	3039(7)	5365(5)	1791(2)	18.8(8)
C22	4392(8)	4176(5)	1687(2)	20.1(9)
C23	3612(8)	2779(5)	1814(2)	17.2(8)
C24	1527(8)	2574(5)	2030(2)	18.4(8)
C25	228(8)	3780(5)	2133(2)	17.6(8)

Table 2: Anisotropic Displacement Parameters (×10[4]) for WT-05-553. The anisotropic displacement factor exponent takes the form: $-2\pi^2[h^2a^{*2} \times U_{11} + ... + 2hka^* \times b^* \times U_{12}]$

Atom	U_{11}	U_{22}	U_{33}	U_{23}	U_{13}	U_{12}
Br1	22.6(2)	11.99(18)	28.9(3)	5.0(2)	1.08(16)	-0.4(2)

Atom	U_{11}	U_{22}	U_{33}	U_{23}	U_{13}	U_{12}
Cl3	27.2(5)	19.5(5)	31.5(6)	1.8(4)	0.1(4)	3.2(4)
Cl1	36.4(7)	22.4(6)	25.6(5)	4.3(4)	-3.2(3)	2.4(3)
Cl2	35.7(7)	20.7(5)	31.0(6)	-1.6(4)	-7.2(4)	0.8(3)
O1	18.1(8)	17.2(14)	16.3(12)	-0.9(5)	-4.0(5)	-0.6(7)
O2	38.0(15)	32.9(17)	27.2(15)	10.1(13)	-15.7(8)	-6.6(14)
O3	18.8(17)	16.0(14)	17.6(11)	1.9(11)	-5.1(8)	-2.3(7)
O4	23.5(11)	13.6(10)	20.9(6)	3.4(5)	-3.4(3)	-2.2(3)
O5	45.3(17)	33.9(13)	21.7(12)	25.3(8)	-7.5(6)	-4.9(5)
N1	17.9(8)	13.8(13)	16.9(7)	0.8(5)	-3.2(4)	-1.3(5)
N2	18.5(15)	19.0(16)	17.6(11)	-0.5(8)	-4.7(7)	-0.3(8)
H2	17(8)	10(20)	18(9)	-3(6)	-2(4)	3(6)
C1	18.1(8)	15.1(16)	17.3(7)	0.4(6)	-3.2(4)	-1.6(5)
H1a	19(5)	15(2)	17(5)	0.3(10)	-2.3(19)	-1.9(10)
H1b	18(3)	16(5)	19(4)	0.1(15)	-3.0(14)	-2.9(17)
C2	17.2(8)	13.7(16)	17.2(7)	1.2(6)	-3.0(4)	-2.1(5)
C3	15.8(8)	9.0(15)	15.4(7)	3.7(6)	-2.3(4)	-3.9(5)
C4	17.1(8)	12.2(16)	16.2(7)	1.3(6)	-3.3(4)	-2.0(5)
C5	17.0(9)	14.3(16)	17.5(7)	2.2(6)	-3.1(4)	-3.2(5)
H5	27(8)	50(30)	23(6)	-15(7)	-10(3)	9(5)
C6	16.4(9)	13.5(12)	17.5(7)	3.4(5)	-2.6(4)	-3.4(4)
C7	17.3(9)	16.6(17)	16.5(8)	1.7(6)	-3.6(4)	-2.1(5)
H7	26(7)	50(20)	23(4)	-16(6)	-12(3)	13(5)
C8	17.3(9)	14.9(16)	16.4(8)	1.2(6)	-3.4(4)	-1.3(5)
H8	26(7)	50(20)	23(4)	-16(6)	-12(3)	13(5)
C9	18.9(10)	14.7(12)	17.2(8)	0.8(6)	-3.6(4)	-0.9(4)
H9	19(4)	15(4)	18(4)	1.6(16)	-3.1(15)	-0.8(16)
C10	30(2)	15.6(12)	20.6(13)	0.5(7)	-8.2(8)	-1.9(6)
H10a	30(4)	18(4)	20(5)	-0.4(16)	-9.0(17)	-1.6(17)
H10b	31(4)	17(4)	22(5)	1.8(16)	-7.6(16)	-2.0(18)
C11	32.4(16)	23.4(17)	20.3(14)	6.0(10)	-6.7(7)	-1.1(7)
H11a	32(5)	24(4)	21(4)	5.9(17)	-7.4(18)	-2.1(16)
H11b	32(4)	24(5)	22(5)	6.0(18)	-6.3(16)	0.5(18)
C12	29.2(15)	18.6(17)	17.6(12)	2.2(10)	-6.6(7)	-0.1(8)
C13	17.5(12)	12.9(15)	16.7(11)	-0.7(7)	-3.7(5)	-0.7(7)
C14	18.1(8)	15.7(8)	19.7(7)	3.7(4)	-4.6(3)	-3.8(3)
H14	19(6)	16(2)	22(6)	3.7(11)	-6(2)	-3.5(11)
C15	18.1(8)	15.3(7)	20.0(6)	3.9(3)	-4.8(3)	-3.7(3)
C16	18.7(8)	17.3(11)	21.1(9)	2.8(4)	-4.0(4)	-4.8(4)
H16a	19(5)	19(5)	22(5)	2.6(17)	-4.1(18)	-4(2)
H16b	20(2)	22(6)	26(7)	3.9(12)	-4.4(11)	-3(2)
C17	19.1(12)	15.8(10)	20.5(6)	3.4(5)	-5.5(3)	-3.3(3)
C18	26.4(7)	15.2(11)	21.4(7)	1.6(4)	-4.5(3)	-1.2(4)
H18a	27(5)	16(3)	21(4)	1.2(15)	-4.2(16)	-1.1(15)
H18b	26(4)	16(3)	23(6)	1.6(15)	-4.7(17)	-1.0(15)
C19	26.9(7)	18.8(6)	25.5(6)	1.4(3)	-2.9(3)	1.4(3)
C20	17.7(8)	14.8(7)	18.3(12)	3.4(3)	-4.9(4)	-3.4(4)
C21	18.6(8)	12.3(6)	25(2)	4.2(3)	-2.4(6)	-2.1(4)
H21	30(5)	11(6)	100(30)	6.7(15)	28(6)	5(3)
C22	19.2(9)	12.0(6)	29(2)	4.1(3)	-0.4(6)	-1.3(4)
H22	30(5)	11(6)	100(30)	6.7(15)	28(6)	5(3)
C23	19.1(8)	11.8(5)	21(2)	3.7(3)	-3.2(6)	-2.0(4)
C24	19.2(8)	14.4(7)	22(2)	3.2(3)	-2.7(6)	-2.6(4)
H24	29(8)	15(2)	90(40)	4.1(14)	22(9)	1.6(19)
C25	18.7(8)	14.8(7)	19(2)	3.3(3)	-4.3(6)	-3.3(4)
H25	38(9)	17(6)	140(50)	7.1(16)	44(11)	7(3)

Table 3: Bond Lengths in Å for WT-05-553.

Atom	Atom	Length/Å
Br1	C23	1.887(4)
Cl3	C19	1.793(5)
Cl1	C19	1.762(5)
Cl2	C19	1.773(5)
O1	C4	1.234(5)
O2	C12	1.213(5)
O3	C13	1.208(5)
O4	C17	1.340(6)
O4	C18	1.426(6)
O5	C17	1.213(5)
N1	C1	1.467(5)
N1	C4	1.353(6)
N1	C9	1.447(6)
N2	H2	0.97(5)
N2	C12	1.381(6)
N2	C13	1.382(5)
C1	H1a	1.04(3)
C1	H1b	1.04(3)
C1	C2	1.489(6)
C2	C3	1.402(6)
C2	C5	1.383(6)
C3	C4	1.469(6)
C3	C8	1.405(6)
C5	H5	1.06(5)
C5	C6	1.405(6)
C6	C7	1.407(6)
C6	C14	1.494(6)
C7	H7	1.0780
C7	C8	1.393(6)
C8	H8	1.0780
C9	H9	1.17(5)
C9	C10	1.531(6)
C9	C13	1.534(6)
C10	H10a	1.08(3)
C10	H10b	1.08(3)
C10	C11	1.523(6)
C11	H11a	1.12(4)
C11	H11b	1.12(4)
C11	C12	1.508(7)
C14	H14	1.070(3)
C14	C15	1.550(6)
C14	C16	1.490(6)
C15	C16	1.516(7)
C15	C17	1.500(6)
C15	C20	1.486(6)
C16	H16a	1.0702(18)
C16	H16b	1.0702(18)
C18	H18a	1.098(9)
C18	H18b	1.097(9)
C18	C19	1.522(7)
C20	C21	1.386(6)
C20	C25	1.405(6)
C21	H21	1.0780
C21	C22	1.399(6)
C22	H22	1.0780
C22	C23	1.403(6)
C23	C24	1.394(7)
C24	H24	1.0780
C24	C25	1.391(6)
C25	H25	1.0780

Table 4: Bond Angles in ° for WT-05-553.

Atom	Atom	Atom	Angle/°
C18	O4	C17	118.1(3)
C4	N1	C1	113.3(4)
C9	N1	C1	122.3(4)
C9	N1	C4	124.1(4)
C12	N2	H2	117(3)
C13	N2	H2	115(3)
C13	N2	C12	127.5(4)
H1a	C1	N1	111.2(2)
H1b	C1	N1	111.2(2)
H1b	C1	H1a	109.1
C2	C1	N1	102.7(4)
C2	C1	H1a	111.2(2)
C2	C1	H1b	111.2(2)
C3	C2	C1	108.4(4)
C5	C2	C1	130.7(4)
C5	C2	C3	120.9(4)
C4	C3	C2	109.2(4)
C8	C3	C2	121.8(4)
C8	C3	C4	129.0(4)
N1	C4	O1	124.4(4)
C3	C4	O1	129.3(4)
C3	C4	N1	106.3(4)
H5	C5	C2	120.8(3)
C6	C5	C2	118.4(4)
C6	C5	H5	120.8(3)
C7	C6	C5	120.1(4)
C14	C6	C5	121.1(4)
C14	C6	C7	118.4(4)
H7	C7	C6	119.0(3)
C8	C7	C6	122.0(4)

Atom	Atom	Atom	Angle/°
C8	C7	H7	119.0(3)
C7	C8	C3	116.7(4)
H8	C8	C3	121.6(3)
H8	C8	C7	121.6(3)
H9	C9	N1	107.5(2)
C10	C9	N1	114.4(4)
C10	C9	H9	107.5(3)
C13	C9	N1	108.9(4)
C13	C9	H9	107.5(2)
C13	C9	C10	110.7(4)
H10a	C10	C9	109.8(3)
H10b	C10	C9	109.8(3)
H10b	C10	H10a	108.3
C11	C10	C9	109.2(4)
C11	C10	H10a	109.8(3)
C11	C10	H10b	109.8(3)
H11a	C11	C10	109.2(3)
H11b	C11	C10	109.2(3)
H11b	C11	H11a	107.892608686(15)
C12	C11	C10	112.1(4)
C12	C11	H11a	109.2(3)
C12	C11	H11b	109.2(3)
N2	C12	O2	119.5(4)
C11	C12	O2	124.0(4)
C11	C12	N2	116.6(4)
N2	C13	O3	121.1(4)
C9	C13	O3	122.7(4)
C9	C13	N2	116.2(4)
H14	C14	C6	115(2)
C15	C14	C6	123.3(4)
C15	C14	H14	111(2)
C16	C14	C6	123.4(4)
C16	C14	H14	114(2)
C16	C14	C15	59.8(3)
C16	C15	C14	58.2(3)
C17	C15	C14	111.3(3)
C17	C15	C16	114.0(4)
C20	C15	C14	124.4(4)
C20	C15	C16	120.6(4)

Atom	Atom	Atom	Angle/°
C20	C15	C17	116.0(4)
C15	C16	C14	62.1(3)
H16a	C16	C14	117.6(2)
H16a	C16	C15	117.6(2)
H16b	C16	C14	117.6(2)
H16b	C16	C15	117.6(2)
H16b	C16	H16a	114.7
O5	C17	O4	123.6(4)
C15	C17	O4	112.0(4)
C15	C17	O5	124.4(4)
H18a	C18	O4	105(2)
H18b	C18	O4	113(2)
H18b	C18	H18a	107.6(11)
C19	C18	O4	108.3(4)
C19	C18	H18a	111(2)
C19	C18	H18b	111(2)
Cl1	C19	Cl3	108.7(3)
Cl2	C19	Cl3	107.7(3)
Cl2	C19	Cl1	110.7(3)
C18	C19	Cl3	111.4(3)
C18	C19	Cl1	107.3(3)
C18	C19	Cl2	110.9(4)
C21	C20	C15	121.3(4)
C25	C20	C15	120.0(4)
C25	C20	C21	118.6(4)
H21	C21	C20	119.1(2)
C22	C21	C20	121.7(4)
C22	C21	H21	119.1(3)
H22	C22	C21	120.8(3)
C23	C22	C21	118.4(4)
C23	C22	H22	120.8(3)
C22	C23	Br1	120.2(4)
C24	C23	Br1	118.8(3)
C24	C23	C22	121.0(4)
H24	C24	C23	120.4(3)
C25	C24	C23	119.1(4)
C25	C24	H24	120.4(3)
C24	C25	C20	121.1(4)
H25	C25	C20	119.4(3)
H25	C25	C24	119.4(3)

Table 5: Torsion Angles in ° for WT-05-553.

Atom	Atom	Atom	Atom	Angle/°
Br1	C23	C22	C21	-179.9(3)
Br1	C23	C24	C25	179.5(3)
Cl3	C19	C18	O4	58.1(3)
Cl1	C19	C18	O4	177.0(3)
Cl2	C19	C18	O4	-61.9(3)
O1	C4	N1	C1	179.6(4)
O1	C4	N1	C9	-7.4(5)
O1	C4	C3	C2	177.0(5)
O1	C4	C3	C8	-4.6(6)

Atom	Atom	Atom	Atom	Angle/˚
O2	C12	N2	C13	-176.8(4)
O2	C12	C11	C10	150.3(5)
O3	C13	N2	C12	176.1(4)
O3	C13	C9	N1	-22.4(5)
O3	C13	C9	C10	-148.9(5)
O4	C17	C15	C14	148.0(4)
O4	C17	C15	C16	-148.5(4)
O4	C17	C15	C20	-1.7(4)
O5	C17	C15	C14	-32.1(5)
O5	C17	C15	C16	31.4(5)
O5	C17	C15	C20	178.2(5)
N1	C1	C2	C3	-4.8(4)
N1	C1	C2	C5	179.0(3)
N1	C4	C3	C2	-1.5(4)
N1	C4	C3	C8	176.9(3)
N1	C9	C10	C11	-179.9(4)
N1	C9	C13	N2	157.8(3)
N2	C12	C11	C10	-29.4(5)
N2	C13	C9	C10	31.2(4)
C1	C2	C3	C4	4.0(4)
C1	C2	C3	C8	-174.5(3)
C1	C2	C5	C6	174.7(5)
C2	C3	C8	C7	-0.9(5)
C2	C5	C6	C7	-1.1(5)
C2	C5	C6	C14	-174.3(4)
C3	C8	C7	C6	-1.4(5)
C5	C6	C7	C8	2.4(5)
C5	C6	C14	C15	-95.8(4)
C5	C6	C14	C16	-22.6(5)
C6	C14	C15	C16	112.3(6)
C6	C14	C15	C17	-141.9(5)
C6	C14	C15	C20	4.8(6)
C6	C14	C16	C15	-112.2(6)
C9	C10	C11	C12	55.9(4)
C14	C15	C20	C21	-77.5(5)
C14	C15	C20	C25	107.2(5)
C14	C16	C15	C17	-101.0(3)
C14	C16	C15	C20	113.9(3)
C15	C20	C21	C22	-174.5(4)
C15	C20	C25	C24	174.1(4)
C20	C21	C22	C23	-0.6(5)
C20	C25	C24	C23	1.6(5)
C21	C22	C23	C24	0.9(5)
C22	C23	C24	C25	-1.4(5)

Table 6: Hydrogen Fractional Atomic Coordinates (×10[4]) and Equivalent Isotropic Displacement Parameters (Å[2]×10[3]) for WT-05-553. U_{eq} is defined as 1/3 of the trace of the orthogonalised U_{ij}.

Atom	x	y	z	U_{eq}
H2	10440(80)	4920(60)	5720(20)	16(11)
H1a	2539(9)	5520(40)	4738(2)	17(3)
H1b	1490(40)	7135(16)	4947(8)	17(3)
H5	-380(60)	6330(30)	3709(5)	32(13)
H7	3374(7)	8960(4)	2514(2)	33(10)
H8	6286(7)	9189(4)	3287(2)	33(10)
H9	4480(50)	6010(30)	5731(10)	17(3)
H10a	7660(40)	8590(20)	5656(8)	23(3)
H10b	5110(50)	8610(20)	5982(4)	23(3)
H11a	8030(20)	8240(30)	6755(10)	26(3)
H11b	6150(40)	6738(19)	6720(9)	26(3)
H14	-890(70)	8640(20)	2420(20)	19(3)
H16a	-2135(7)	5640(5)	2896(2)	20(3)
H16b	-3773(8)	6866(4)	2452(2)	22(3)
H18a	-1610(50)	6220(30)	163(17)	22(3)
H18b	-1960(50)	8010(30)	430(20)	22(3)
H21	3626(7)	6444(5)	1700(2)	48(13)
H22	5997(8)	4329(5)	1513(2)	48(13)
H24	929(8)	1495(5)	2117(2)	45(17)
H25	-1371(8)	3629(5)	2312(2)	70(20)

Citations

CrysAlisPro (ROD), Rigaku Oxford Diffraction, Poland (?).

CrysAlisPro Software System, Rigaku Oxford Diffraction, (2024).

L.J. Bourhis and O.V. Dolomanov and R.J. Gildea and J.A.K. Howard and H. Puschmann, The Anatomy of a Comprehensive Constrained, Restrained, Refinement Program for the Modern Computing Environment - Olex2 Disected, *Acta Cryst. A*, (2015), **A71**, 59-71.

O.V. Dolomanov and L.J. Bourhis and R.J. Gildea and J.A.K. Howard and H. Puschmann, Olex2: A complete structure solution, refinement and analysis program, *J. Appl. Cryst.*, (2009), **42**, 339-341.

Sheldrick, G.M., ShelXT-Integrated space-group and crystal-structure determination, *Acta Cryst.*, (2015), **A71**, 3-8.

Section 4: Spectroscopic Data

^{1}H NMR Spectra

^{1}H NMR Spectrum for Compound B

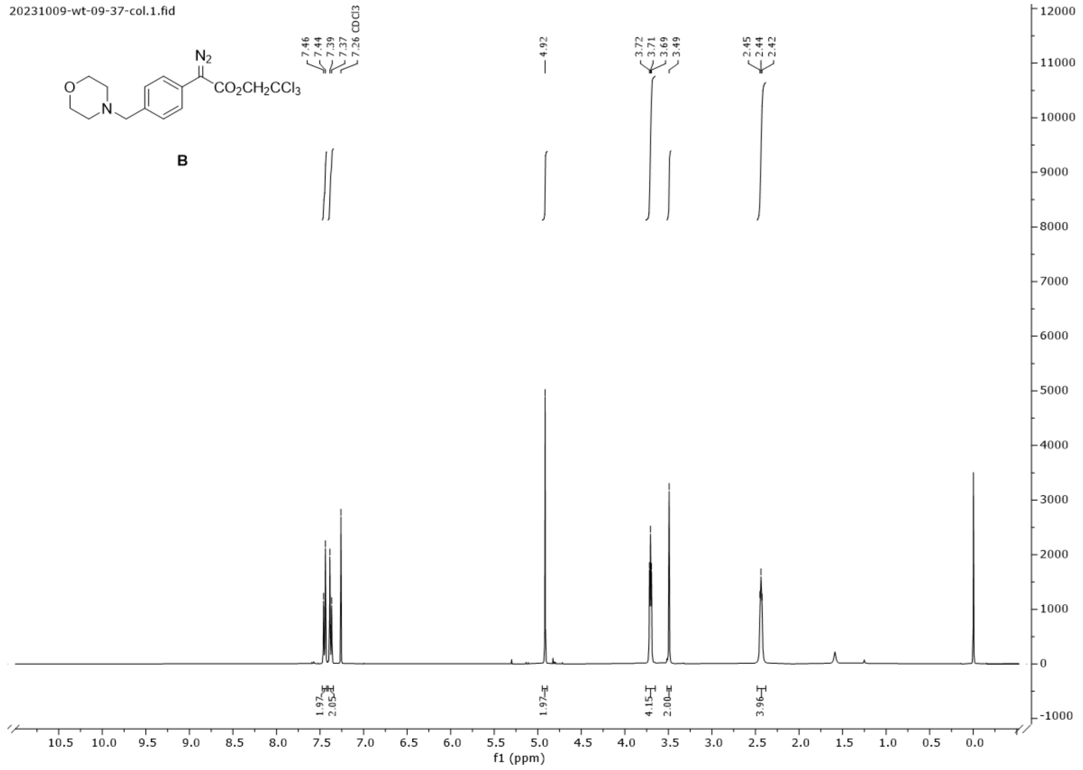

^{1}H NMR Spectrum for Compounds 13a and 13b

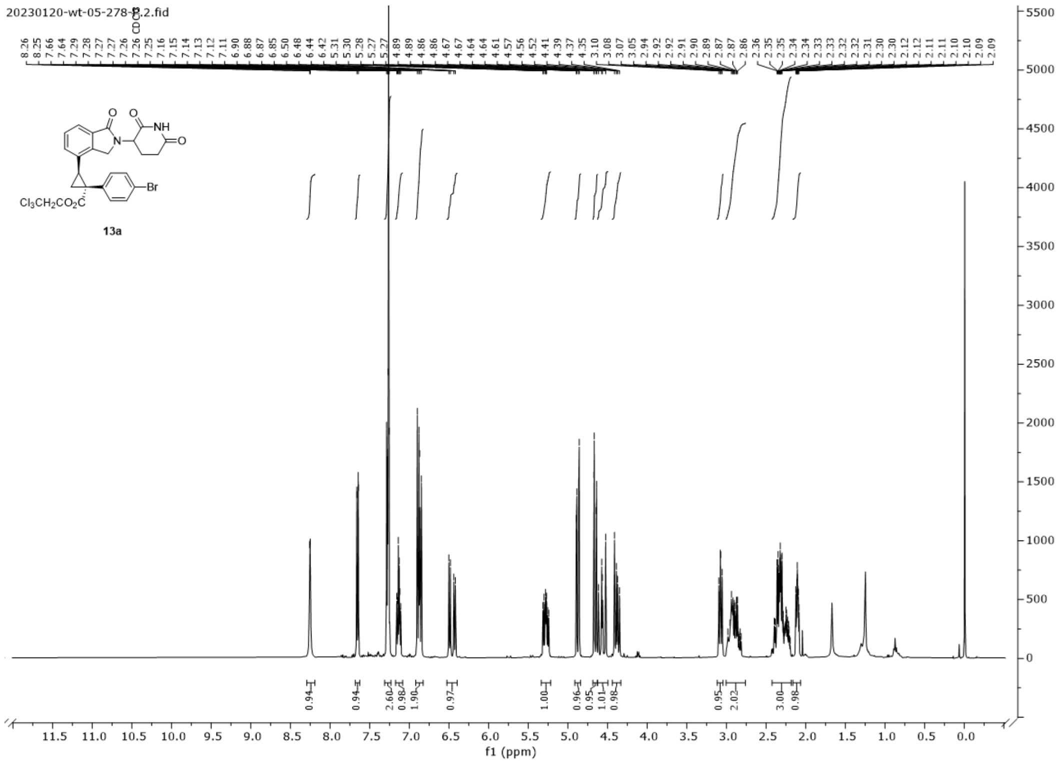

^{1}H NMR Spectrum for Compounds 14a and 14b

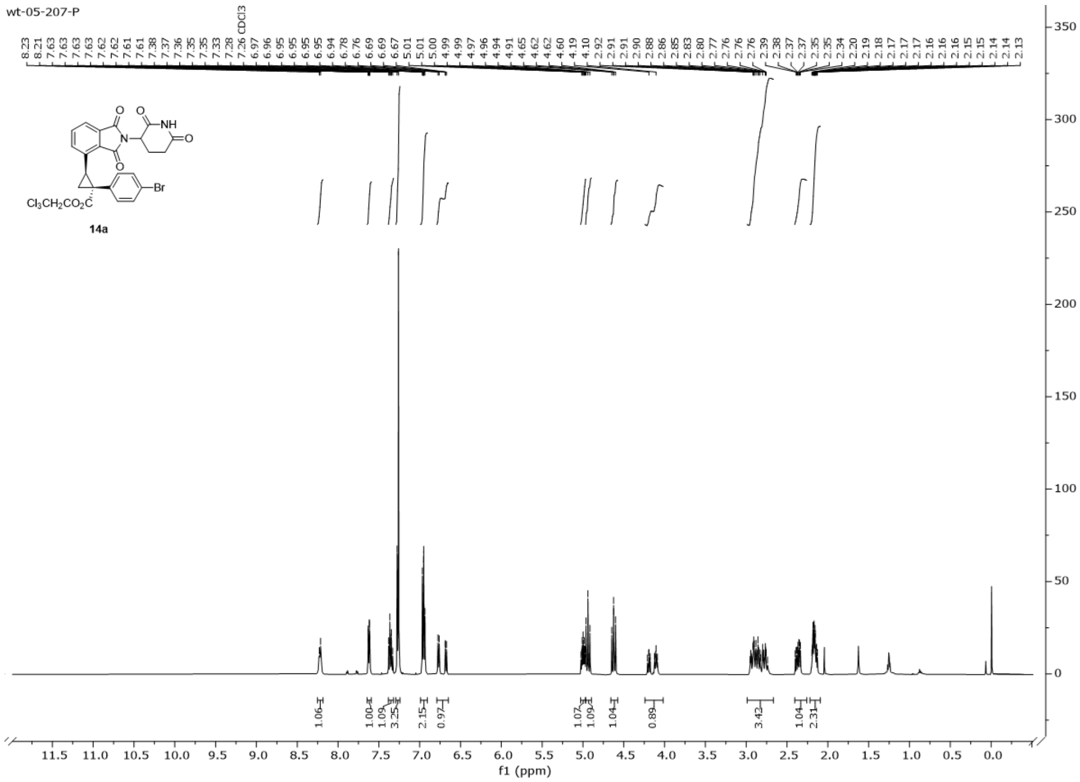

^{1}H NMR Spectrum for Compounds 15a and 15b

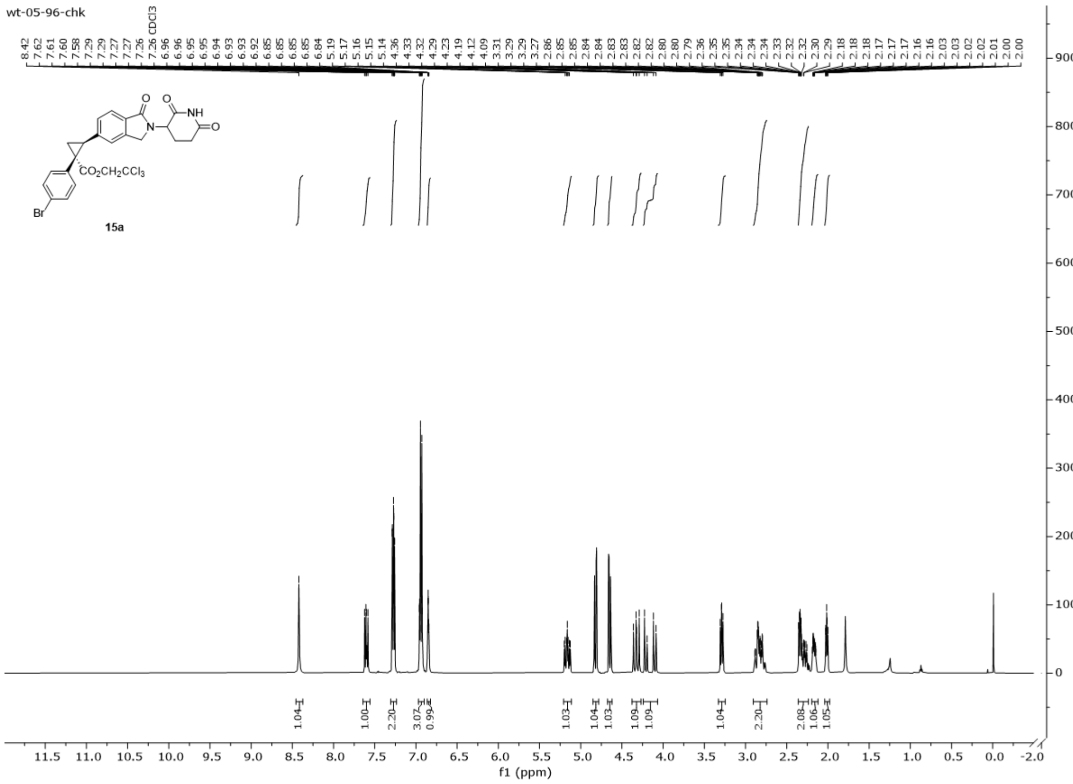

^{1}H NMR Spectrum for Compounds *S*-15a and *R*-15b

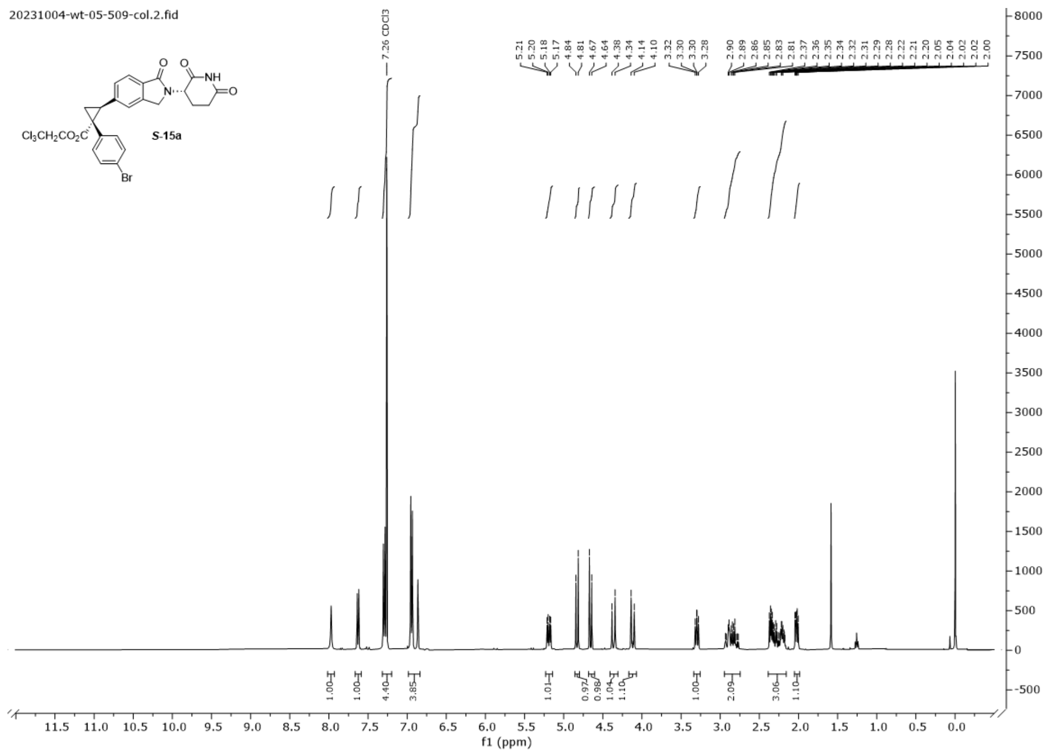

^{1}H NMR Spectrum for Compounds ***R*-15a and *S*-15b**

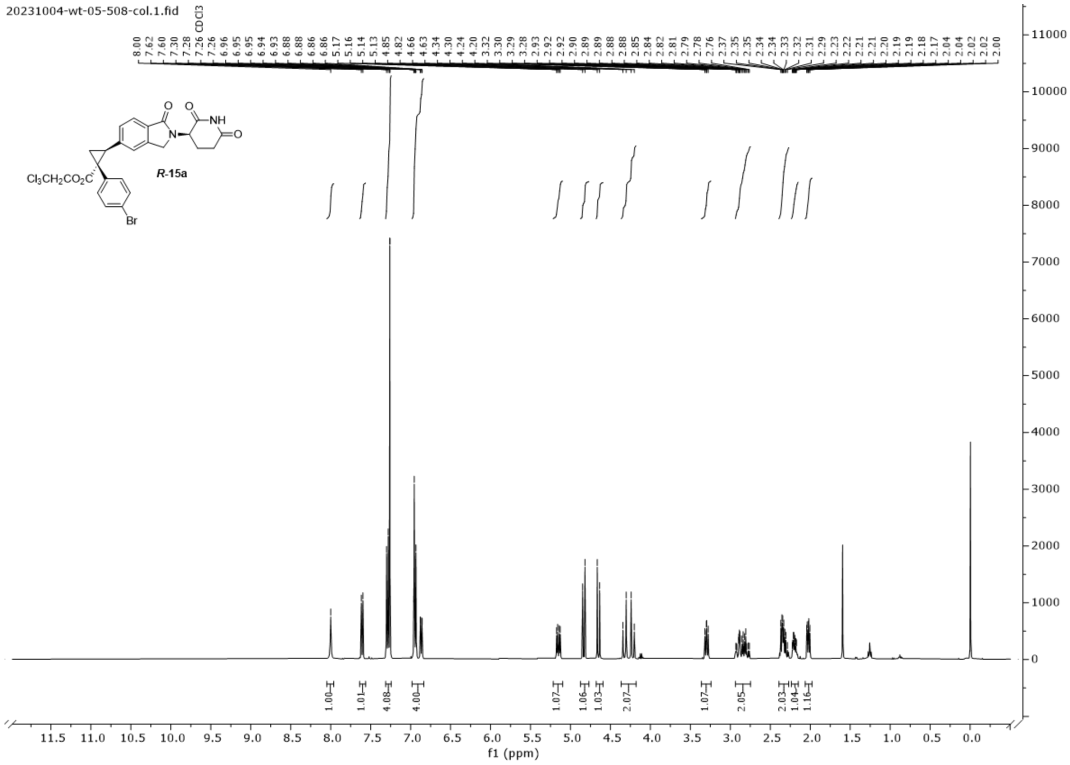

^{1}H NMR Spectrum for Compounds 16a and 16b

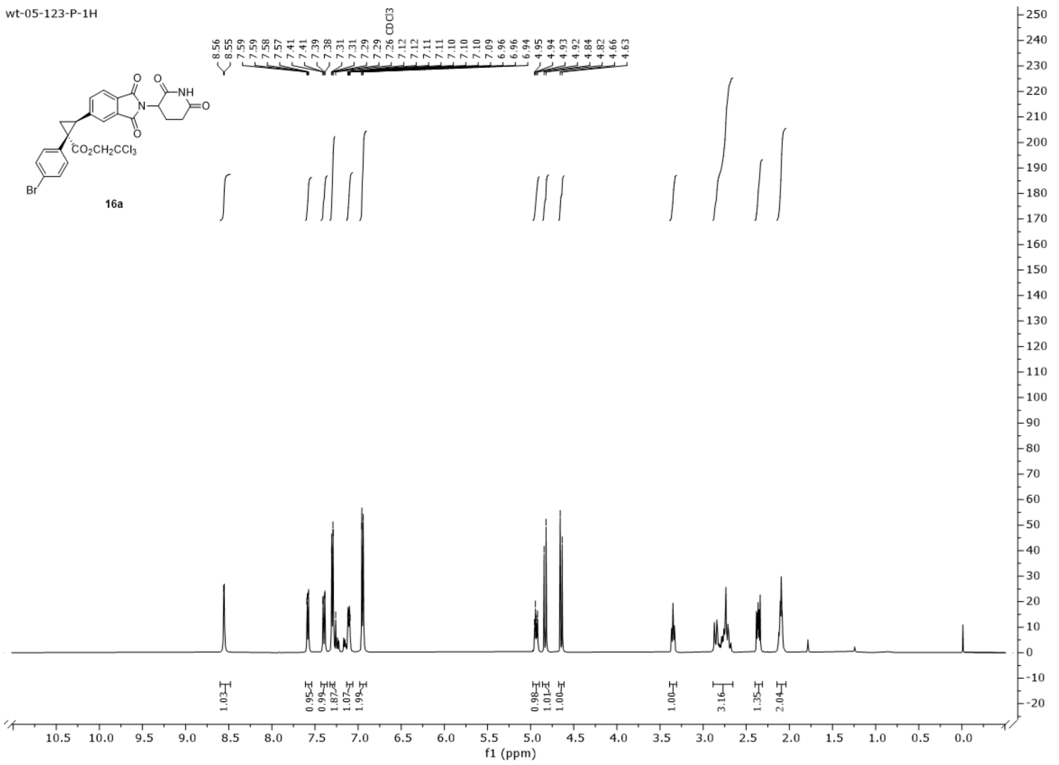

^{1}H NMR Spectrum for Compounds 17a and 17b

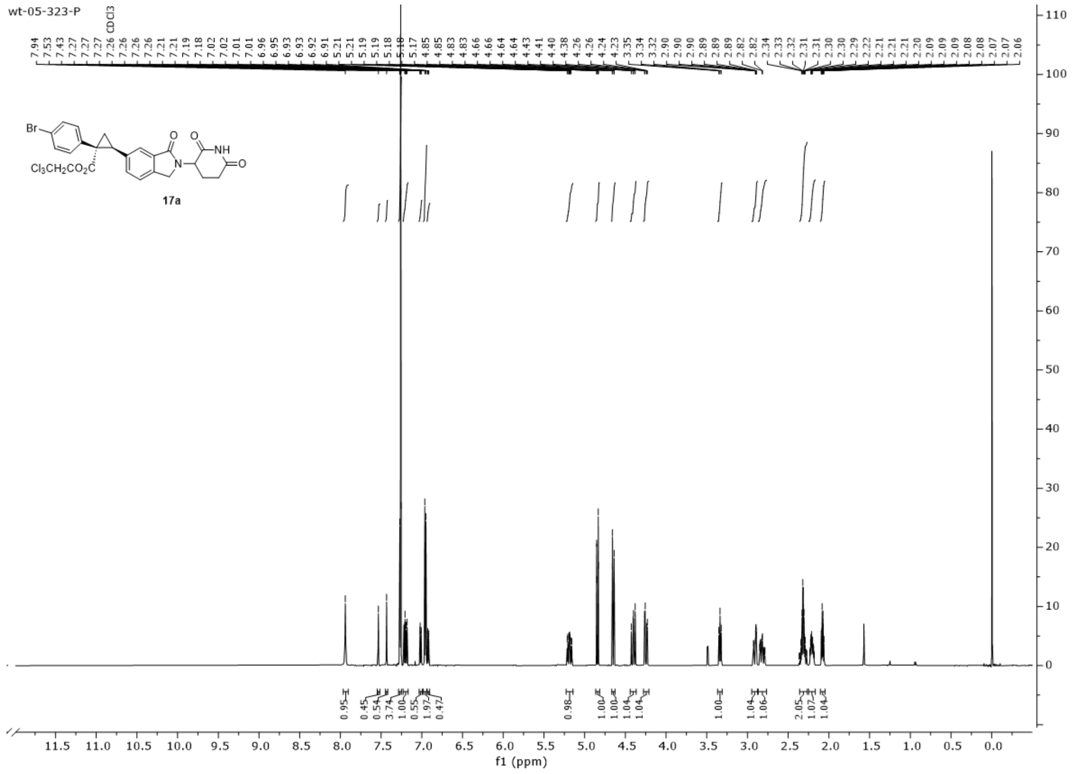

^{1}H NMR Spectrum for Compounds 18a and 18b

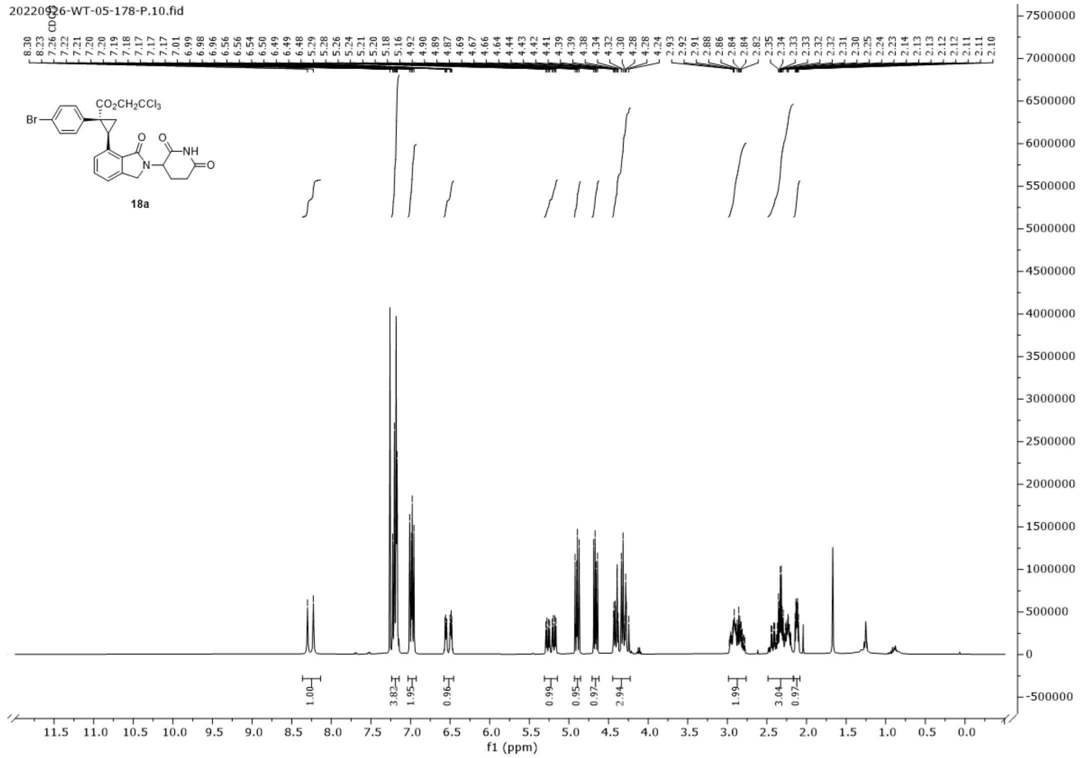

^{1}H NMR Spectrum for Compounds 19a and 19b

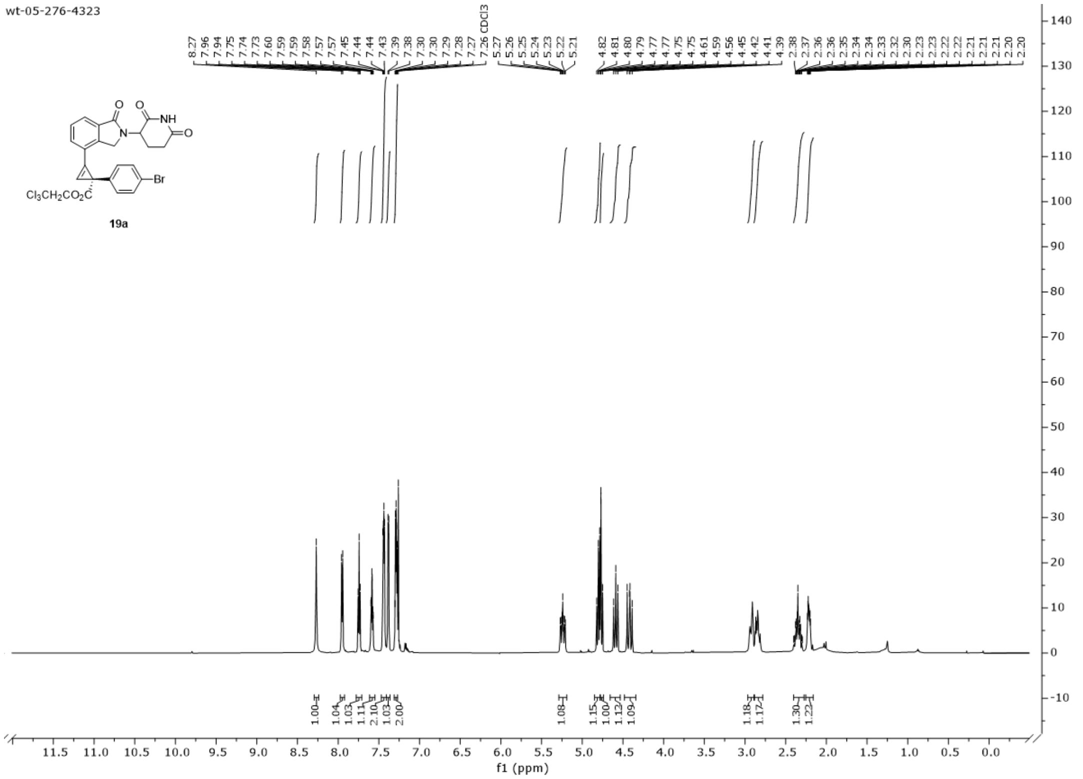

^{1}H NMR Spectrum for Compounds 20a and 20b

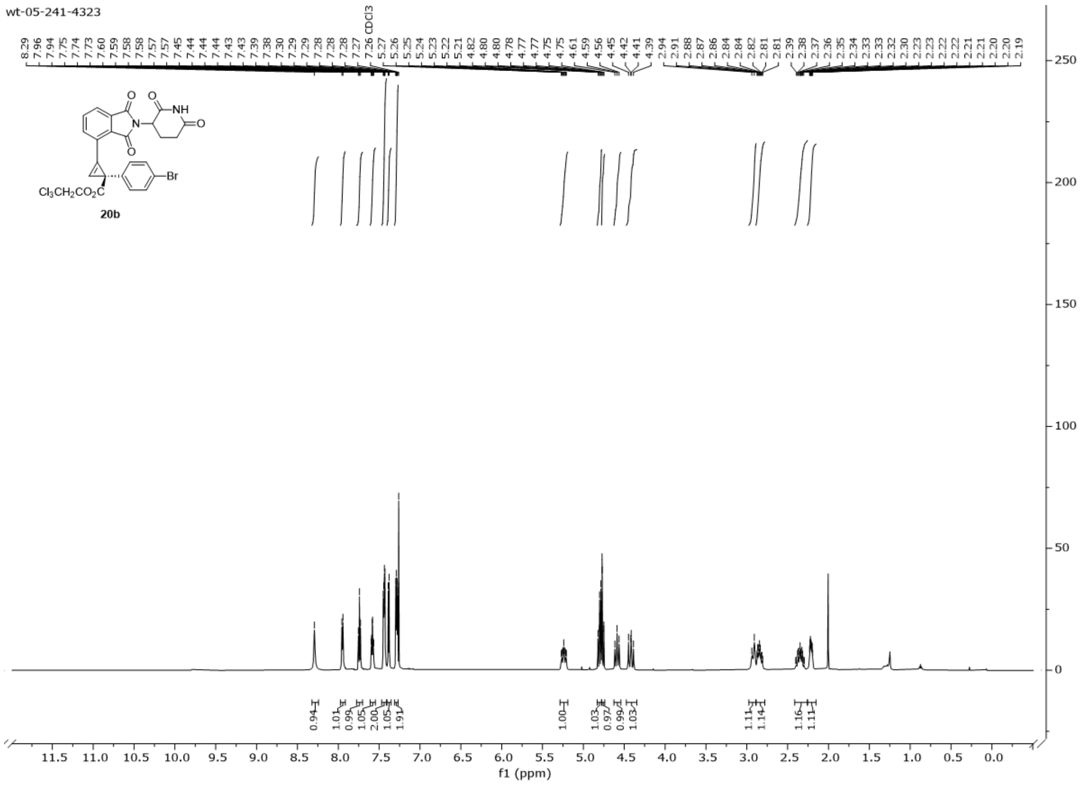

^{1}H NMR Spectrum for Compounds 21a and 21b

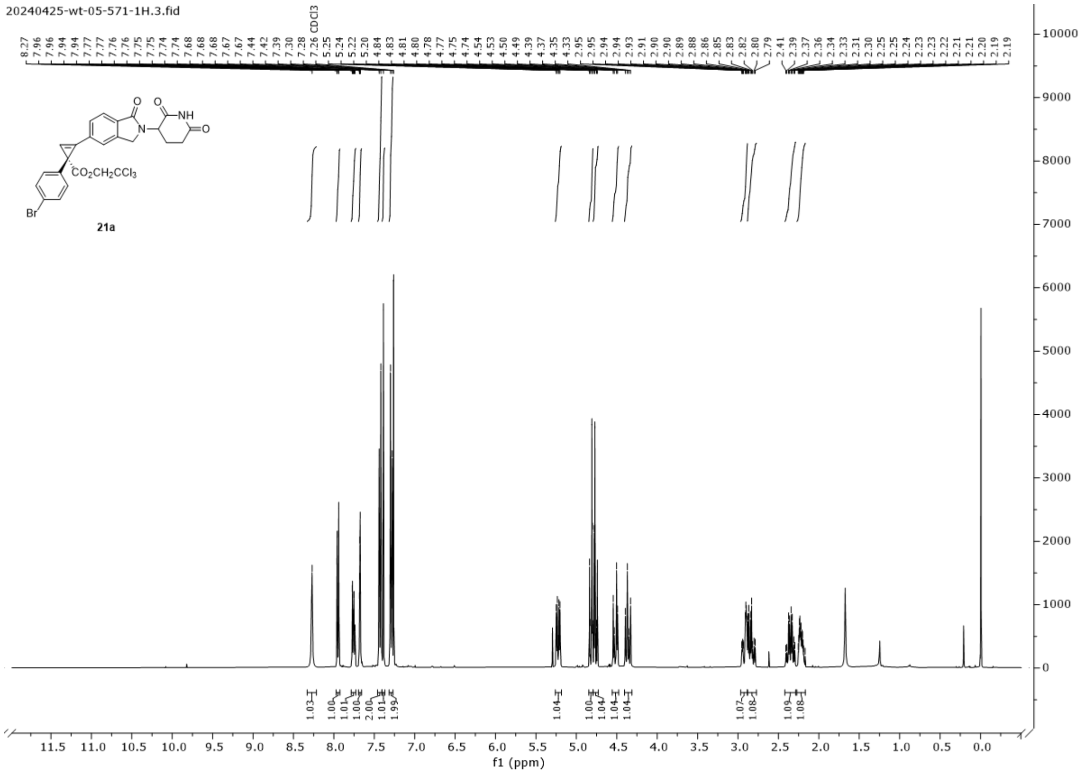

^{1}H NMR Spectrum for Compounds 22a and 22b

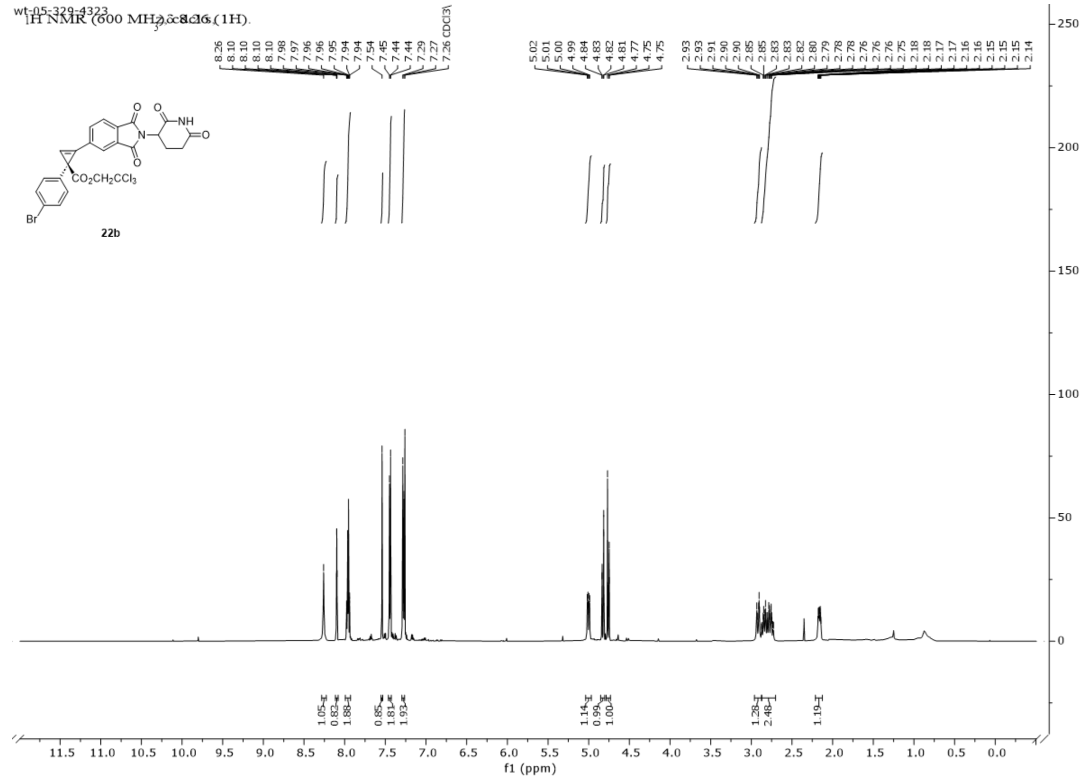

^{1}H NMR Spectrum for Compounds 23a and 23b

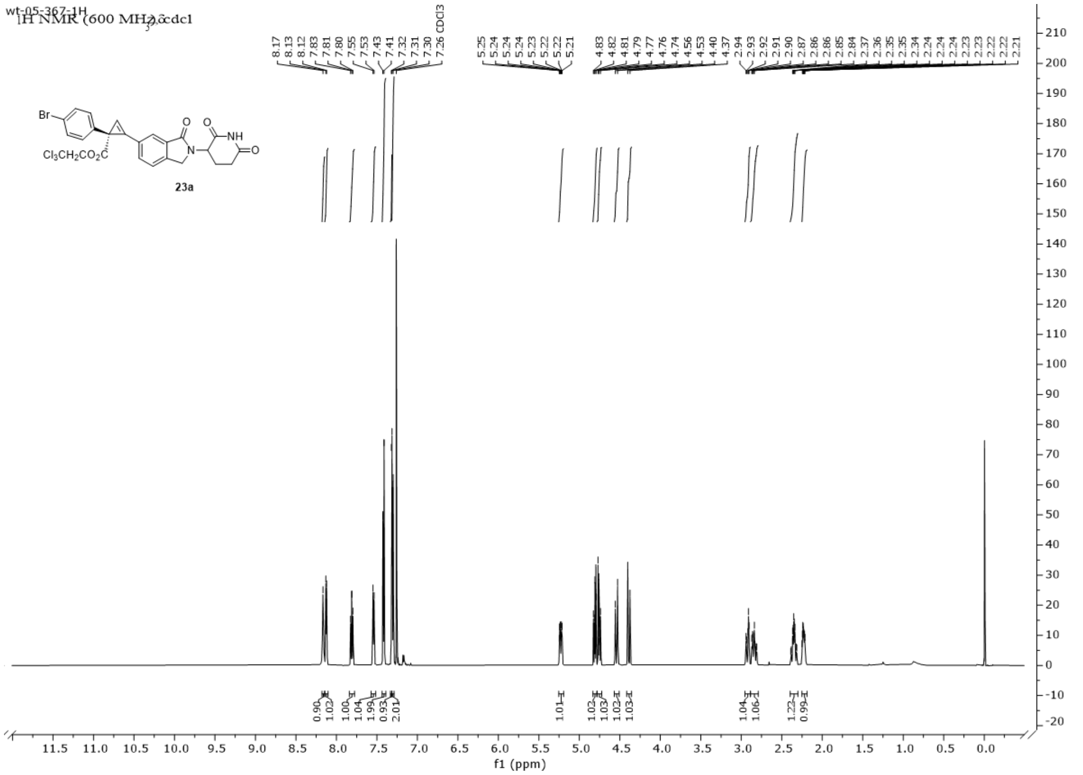

^{1}H NMR Spectrum for Compounds 24a and 24b

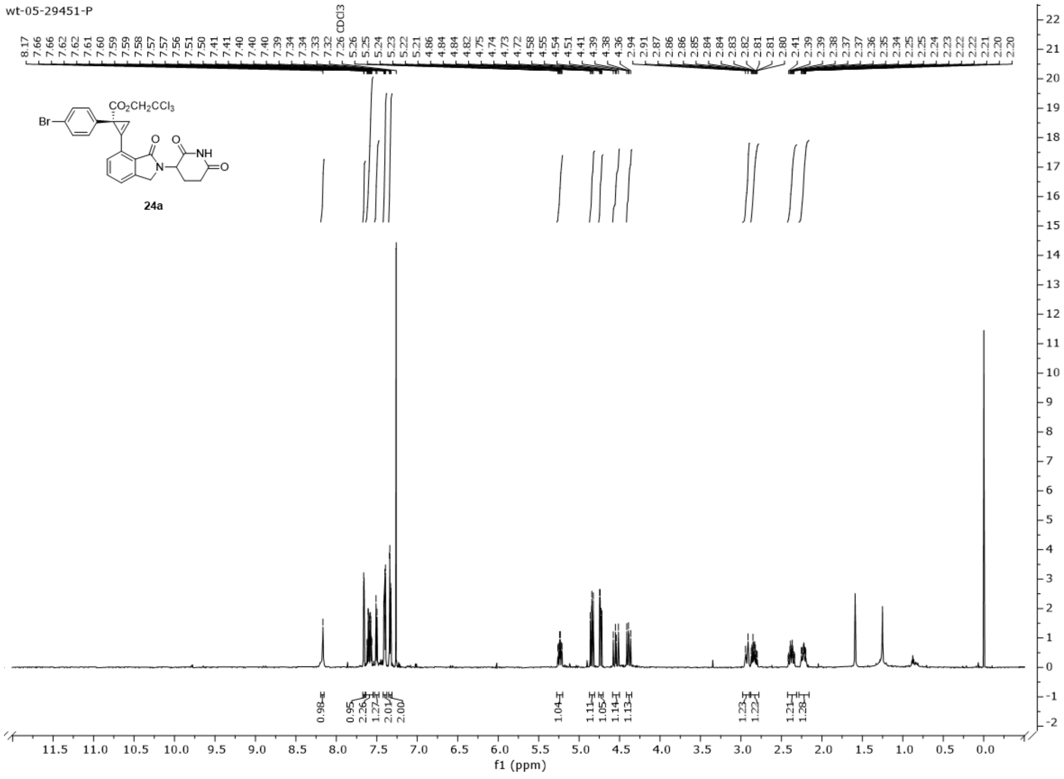

^{1}H NMR Spectrum for Compounds 25a and 25b

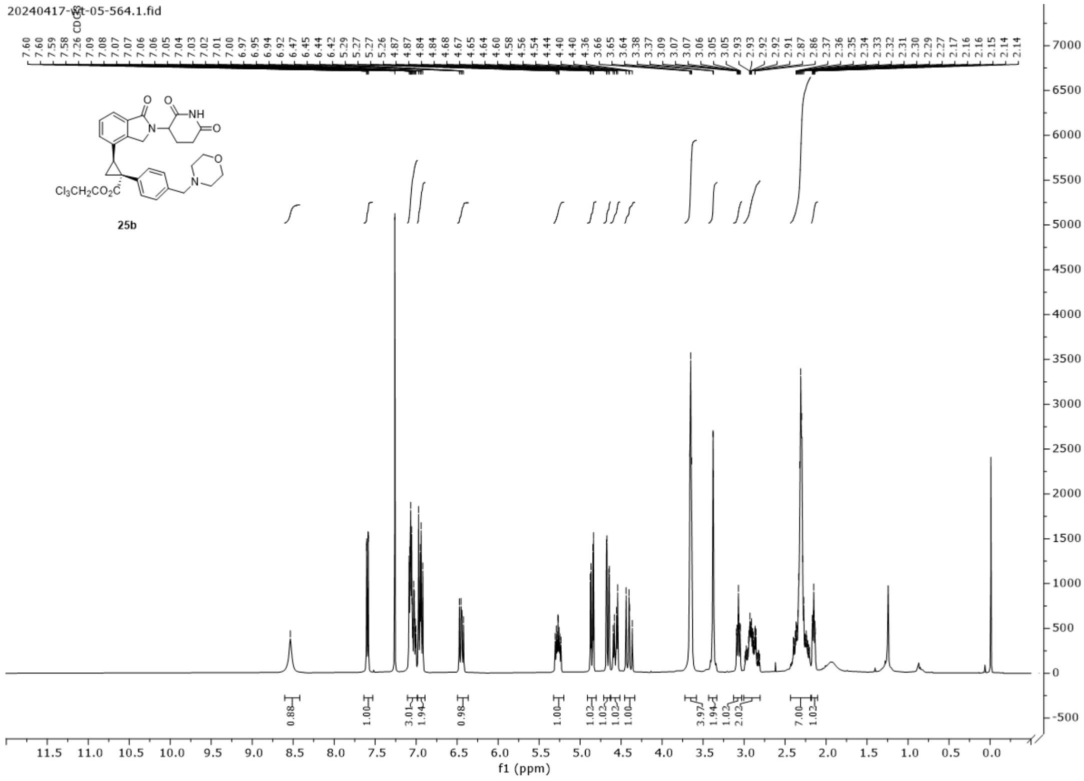

^{1}H NMR Spectrum for Compounds 26a and 26b

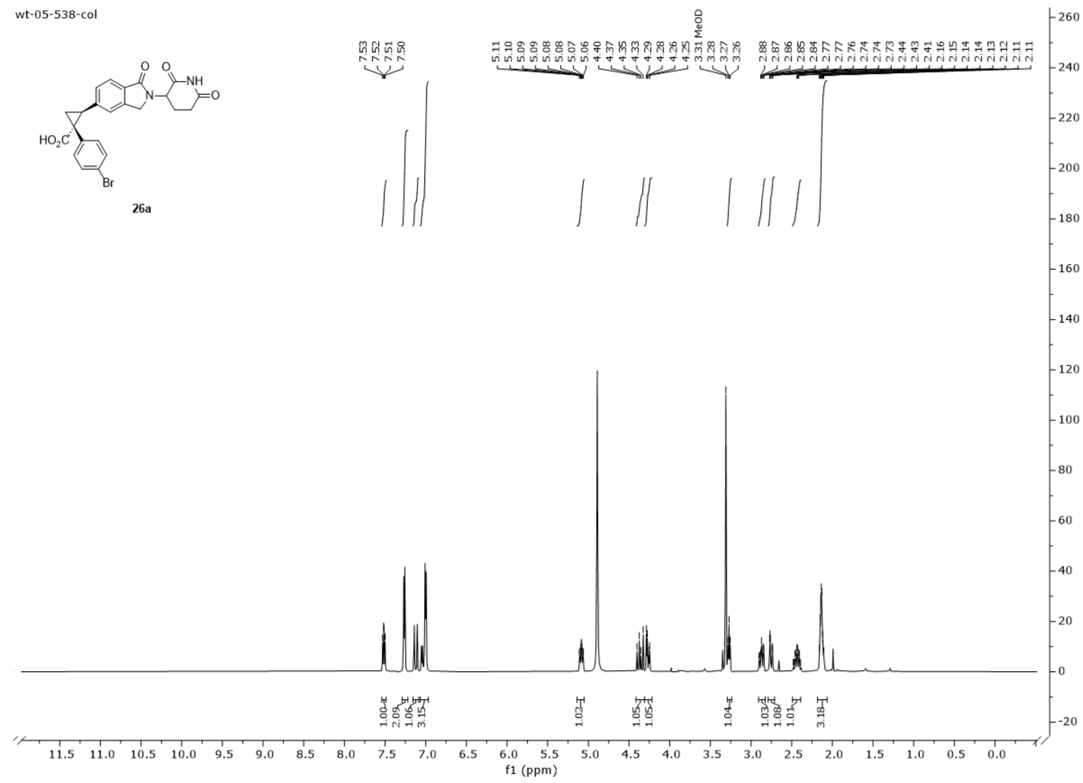

^{1}H NMR Spectrum for Compounds 27a and 27b

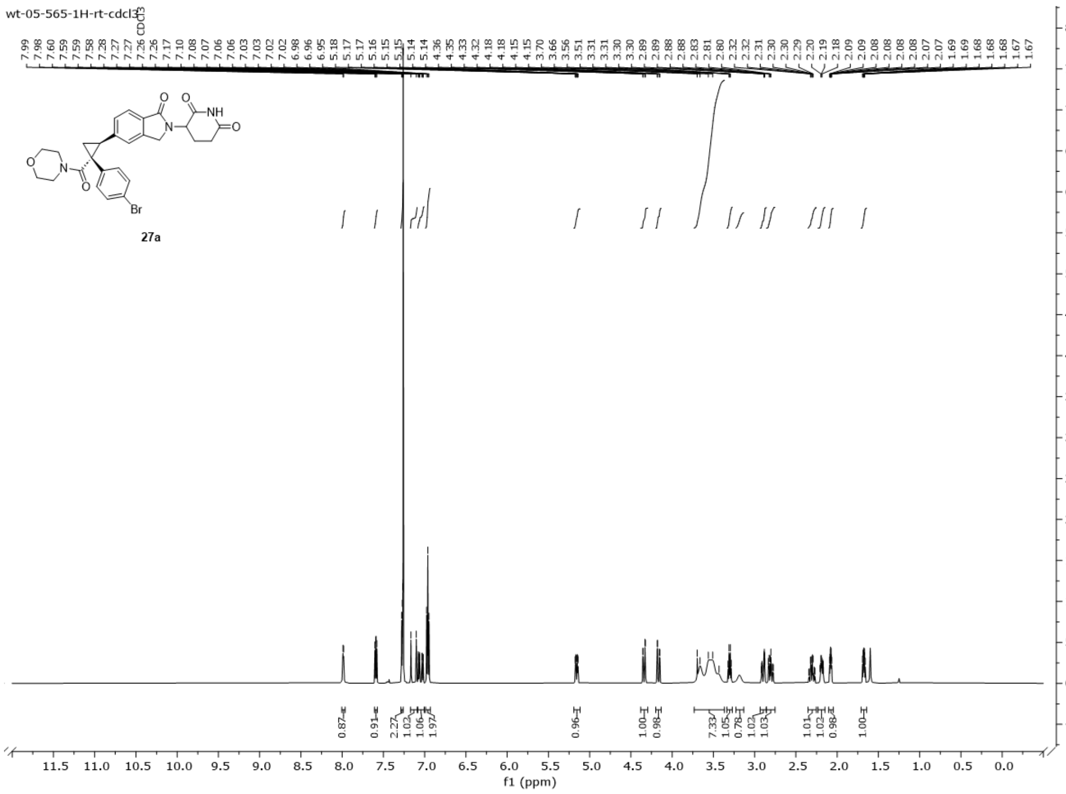

$^{13}C\{^1H\}$ NMR Spectra

$^{13}C\{^1H\}$ NMR Spectrum for Compounds 13a and 13b.

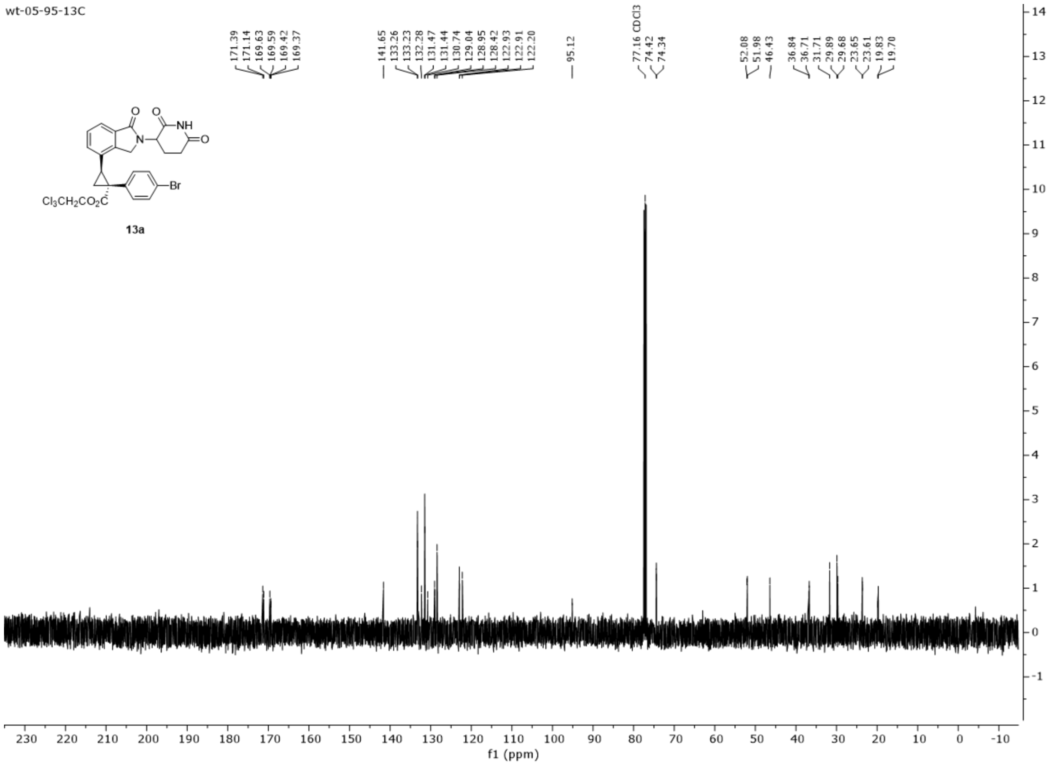

$^{13}C\{^1H\}$ NMR Spectrum for Compounds 14a and 14b.

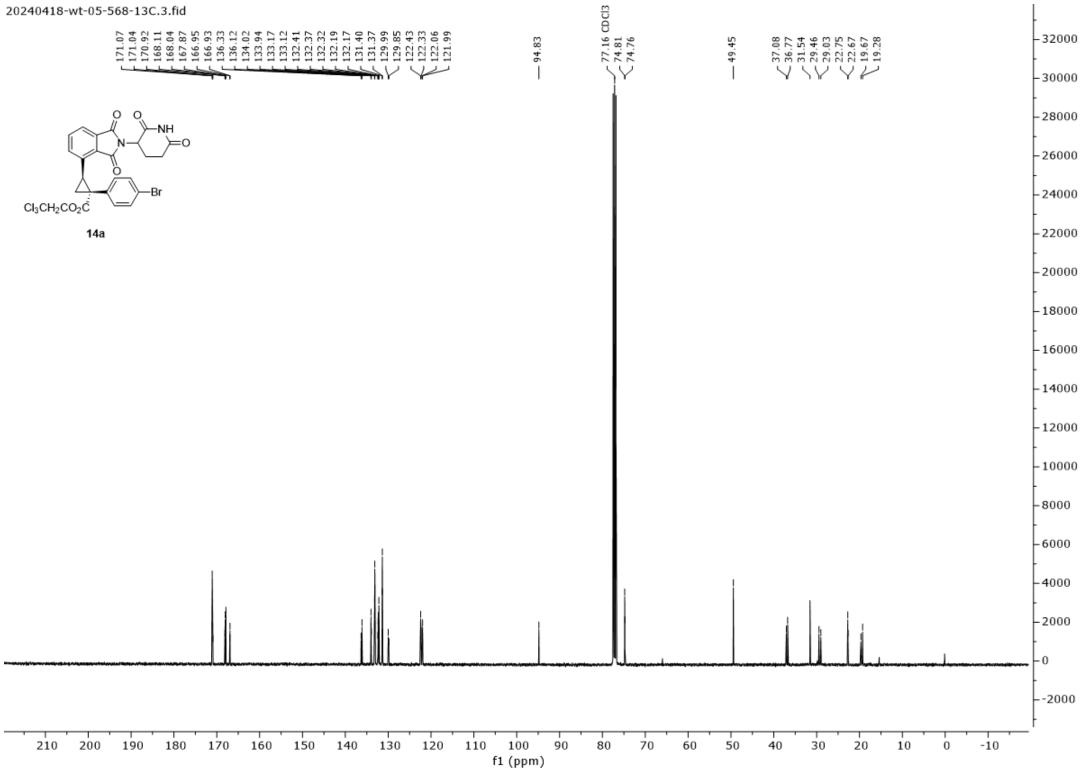

$^{13}C\{^1H\}$ NMR Spectrum for Compounds 15a and 15b.

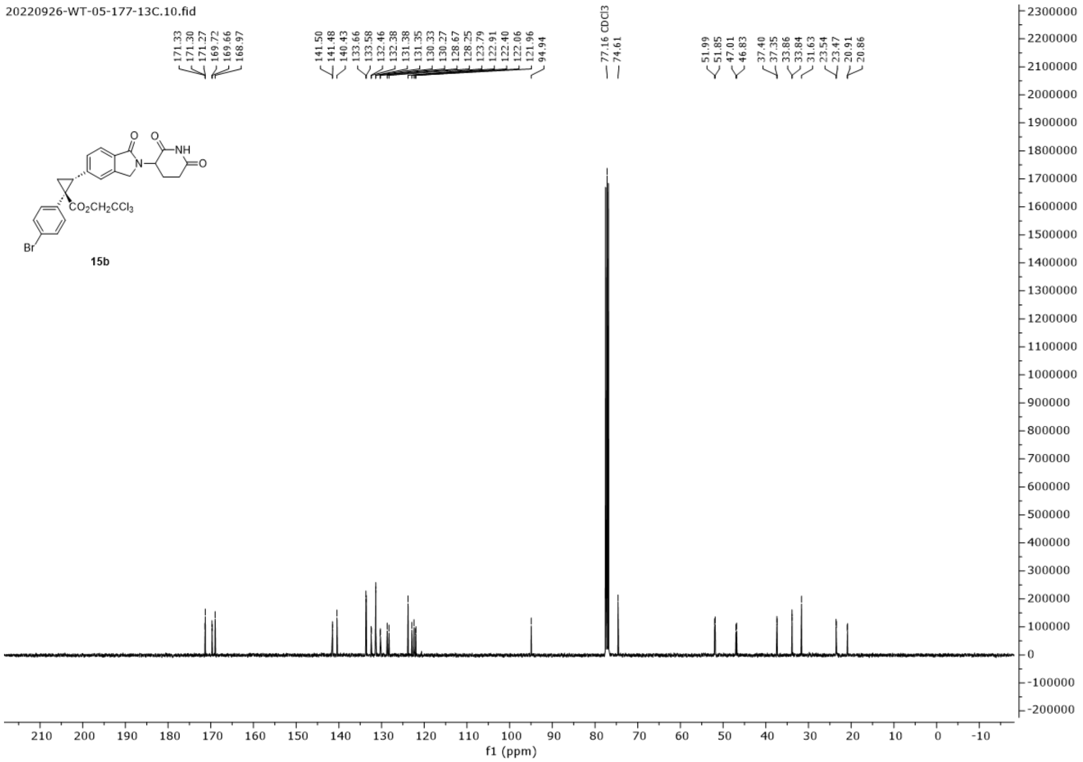

$^{13}C\{^1H\}$ NMR Spectrum for Compounds *S*-15a and *R*-15b.

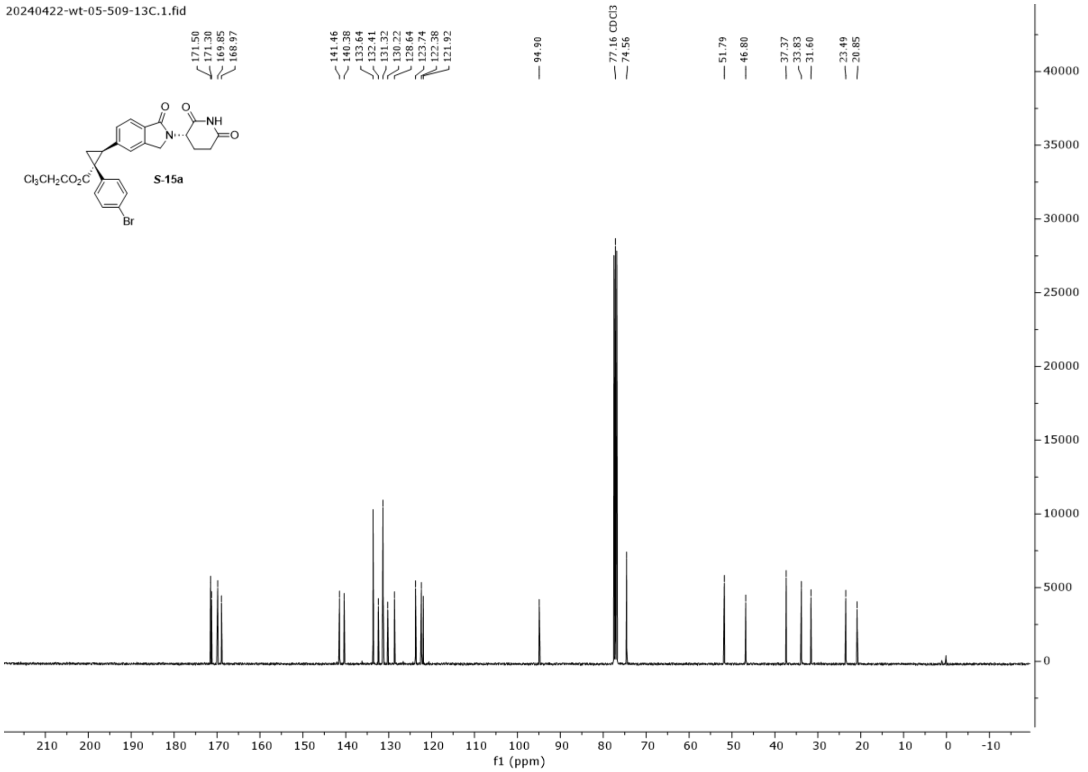

$^{13}C\{^1H\}$ NMR Spectrum for Compounds *R*-15a and *S*-15b.

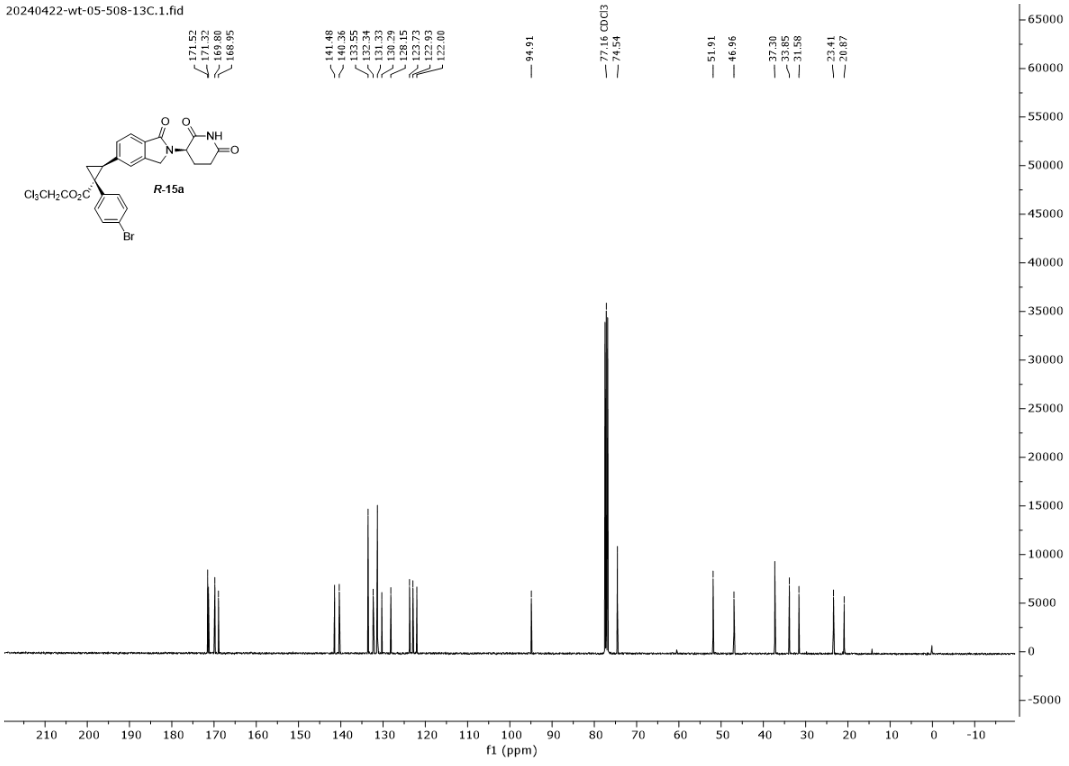

$^{13}C\{^1H\}$ NMR Spectrum for Compounds 16a and 16b.

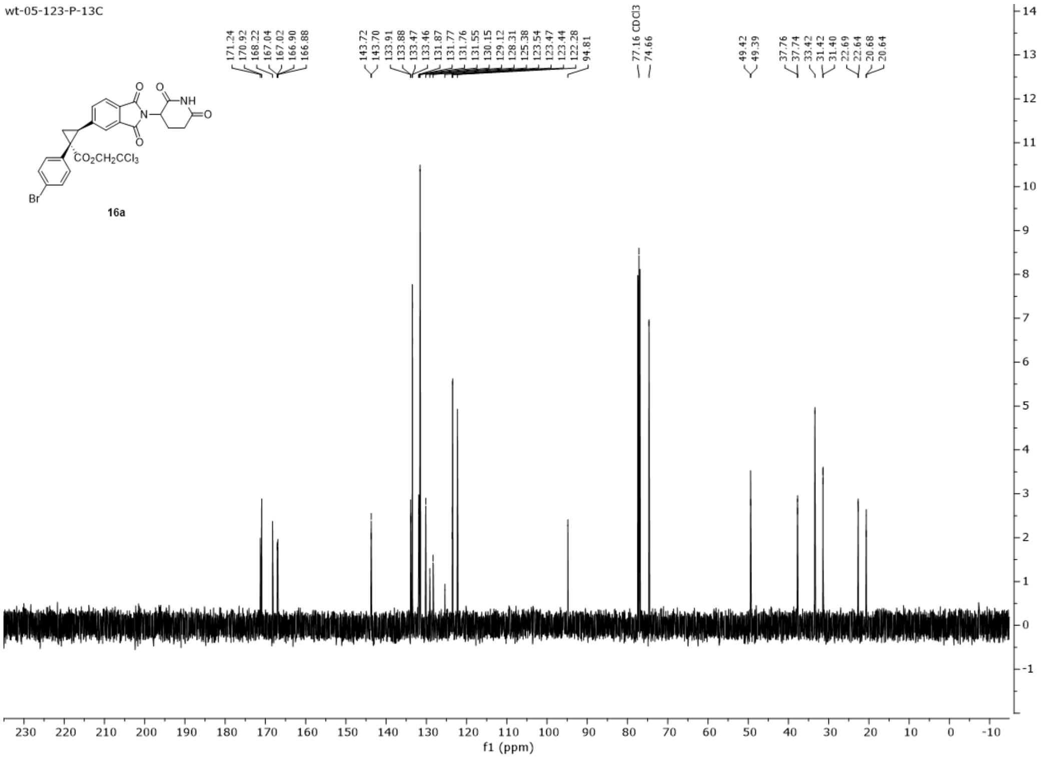

$^{13}C\{^1H\}$ NMR Spectrum for Compounds 17a and 17b.

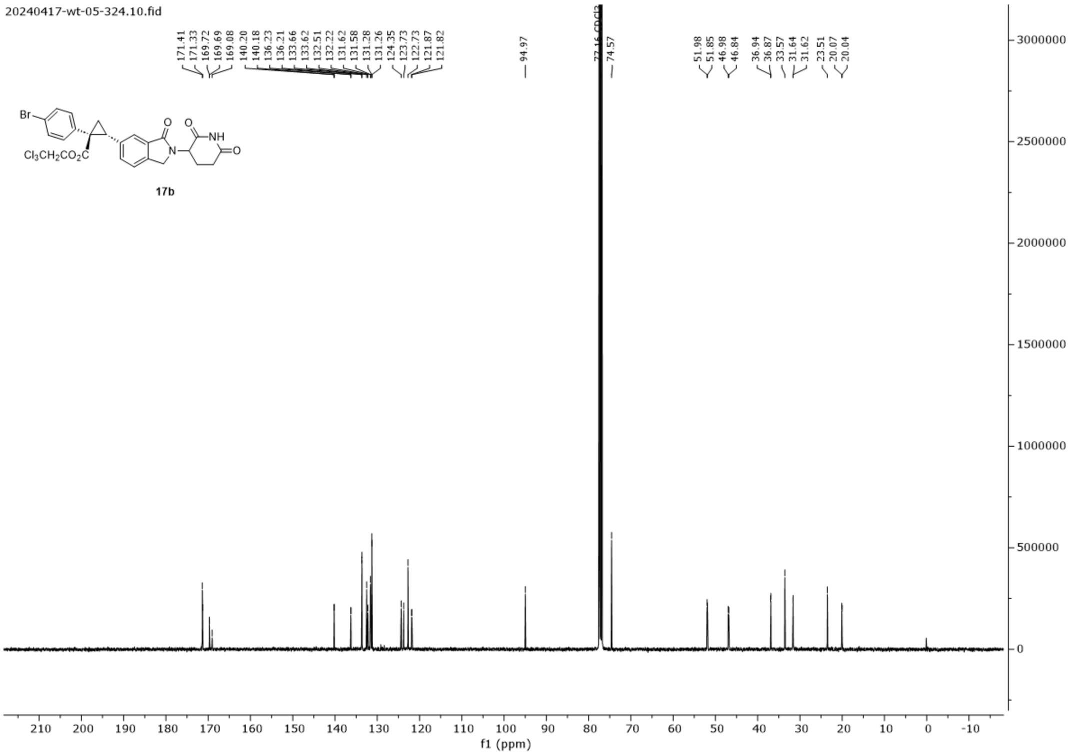

$^{13}C\{^1H\}$ NMR Spectrum for Compounds 18a and 18b.

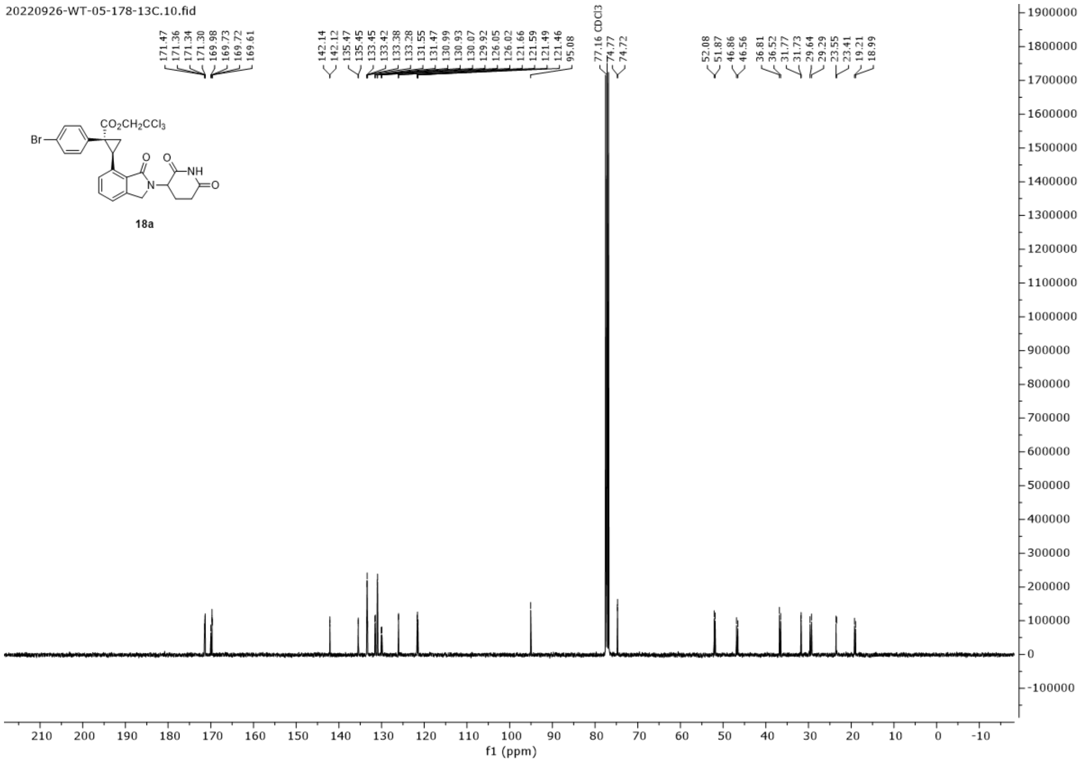

$^{13}C\{^1H\}$ NMR Spectrum for Compounds 19a and 19b.

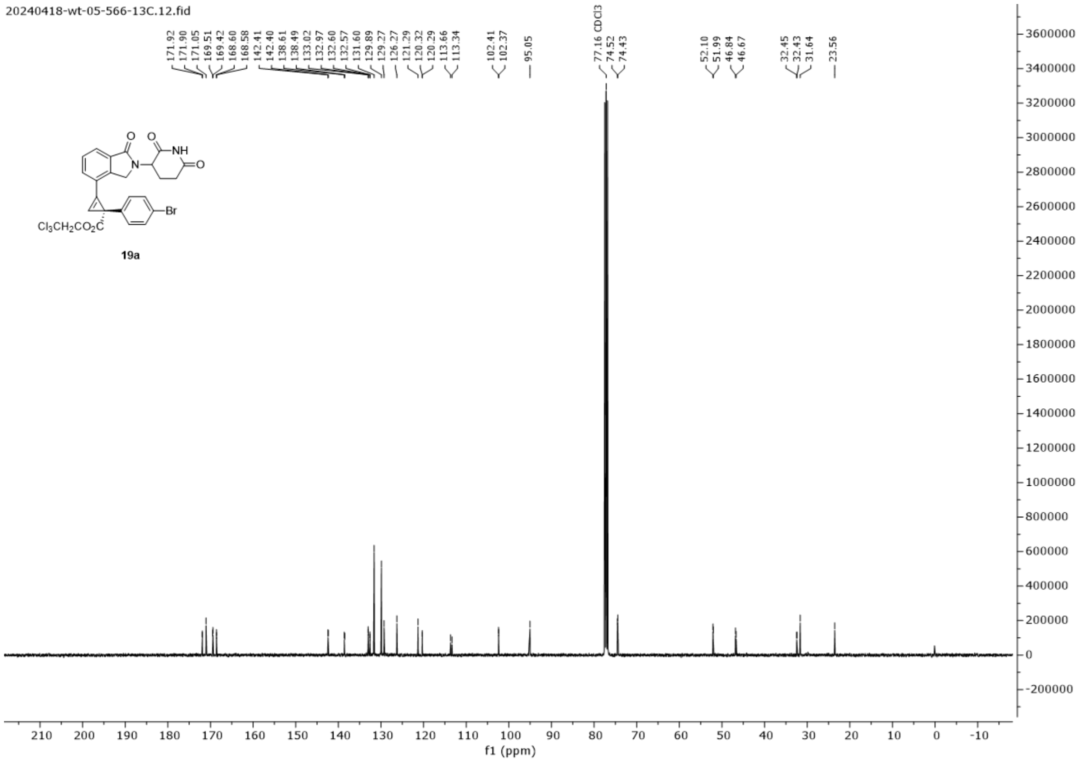

$^{13}C\{^1H\}$ NMR Spectrum for Compounds 20a and 20b.

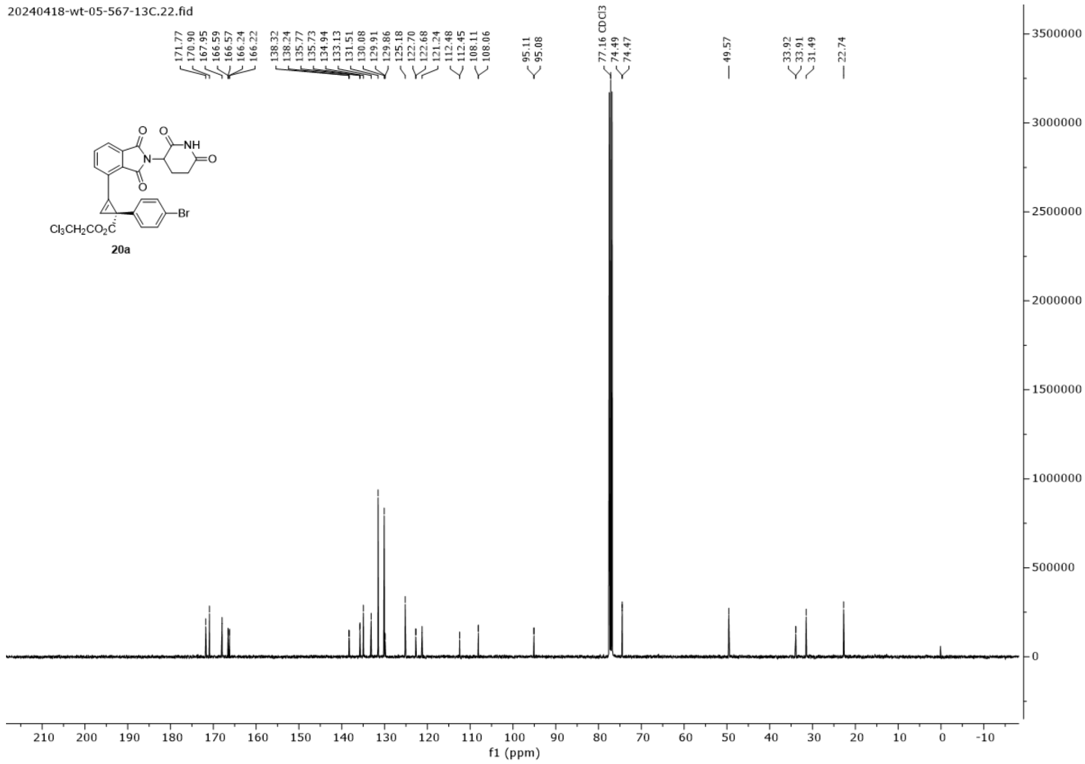

$^{13}C\{^1H\}$ NMR Spectrum for Compounds 21a and 21b.

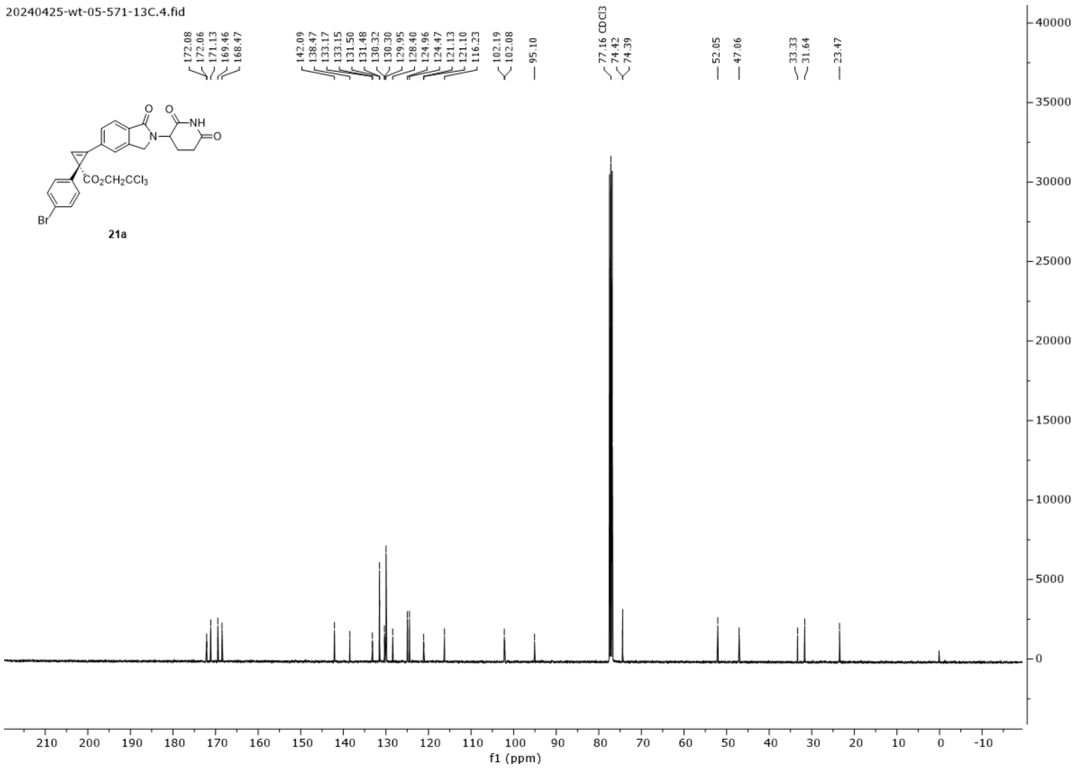

$^{13}C\{^1H\}$ NMR Spectrum for Compounds 22a and 22b.

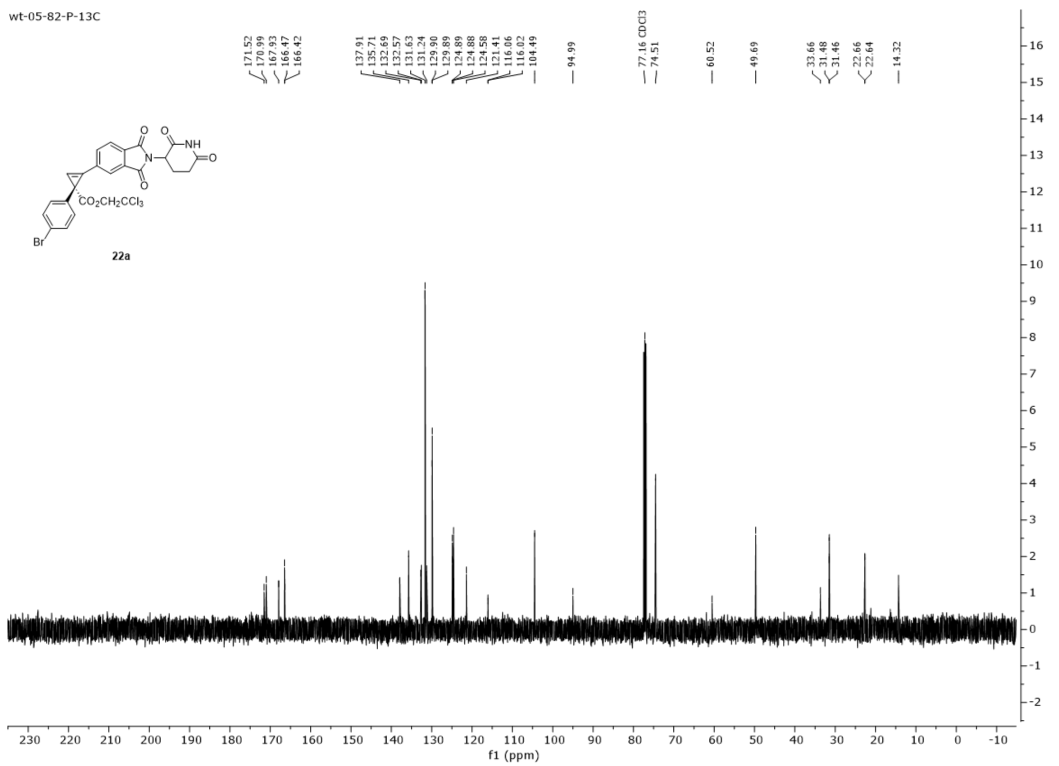

$^{13}C\{^1H\}$ NMR Spectrum for Compounds 23a and 23b.

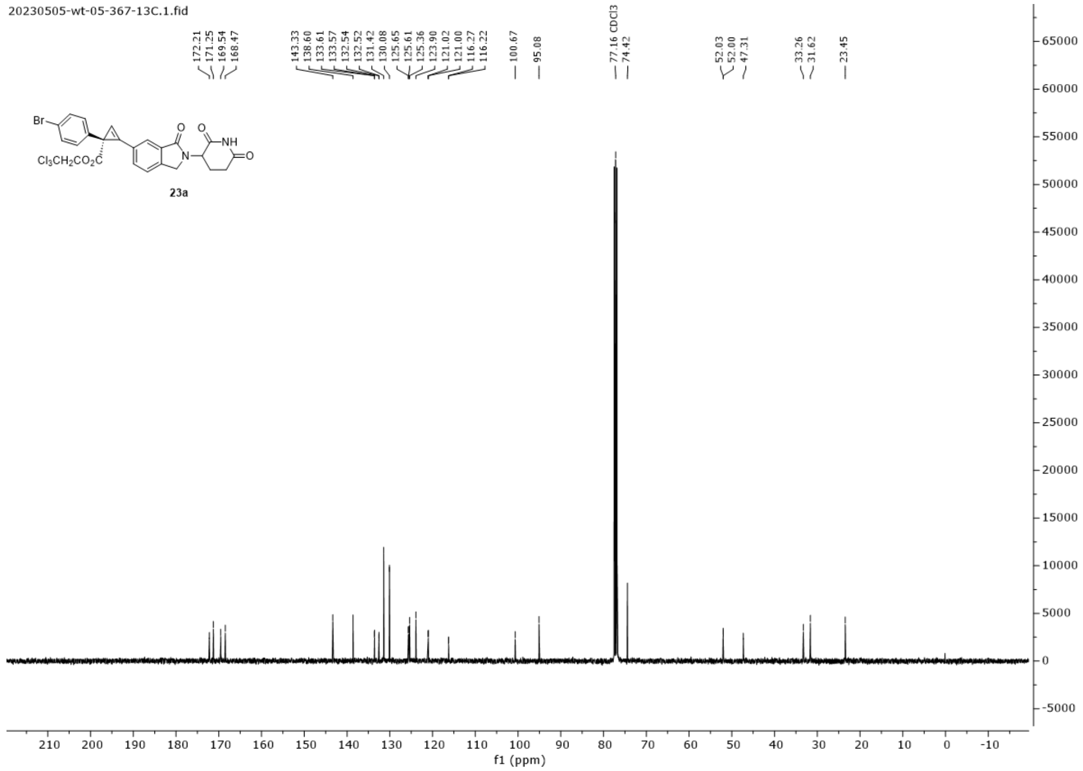

$^{13}C\{^{1}H\}$ NMR Spectrum for Compounds 24a and 24b.

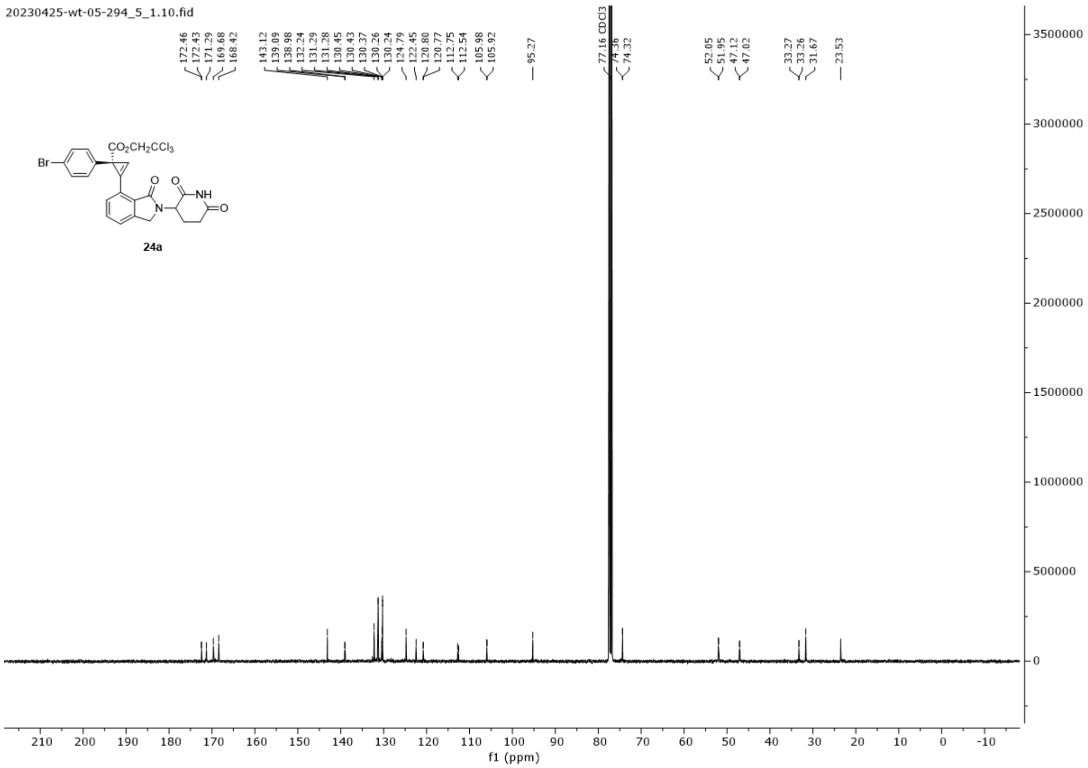

$^{13}C\{^{1}H\}$ NMR Spectrum for Compounds 25a and 25b.

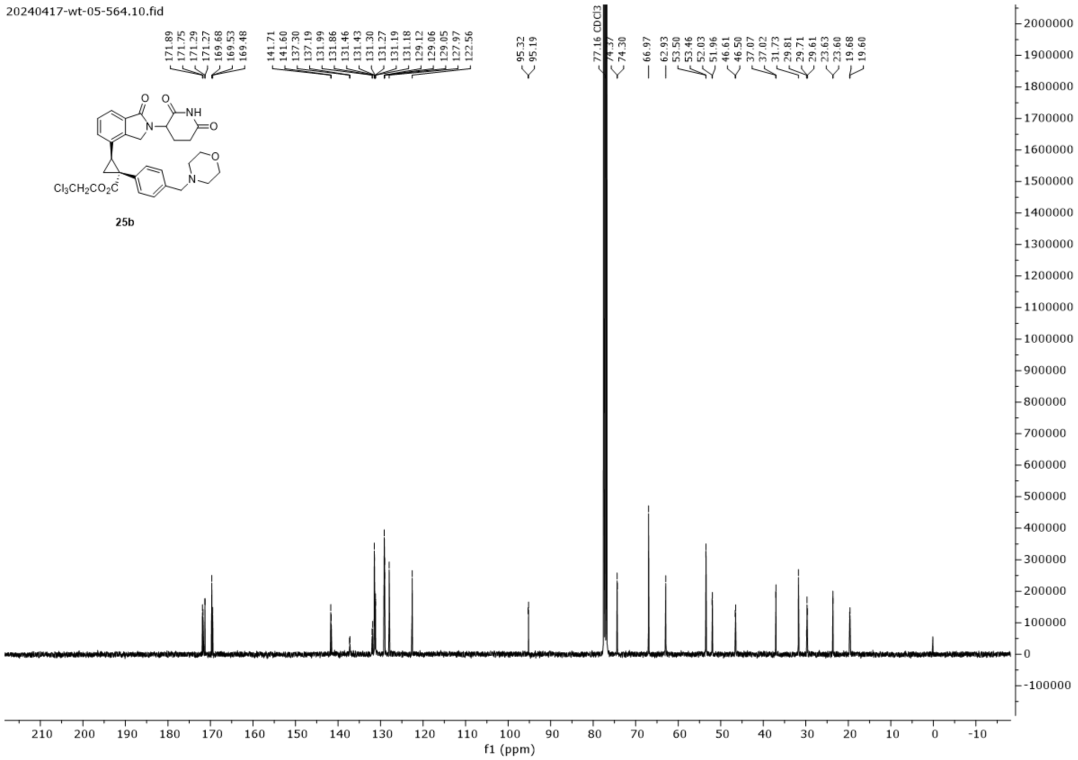

$^{13}C\{^{1}H\}$ NMR Spectrum for Compounds 26a and 26b.

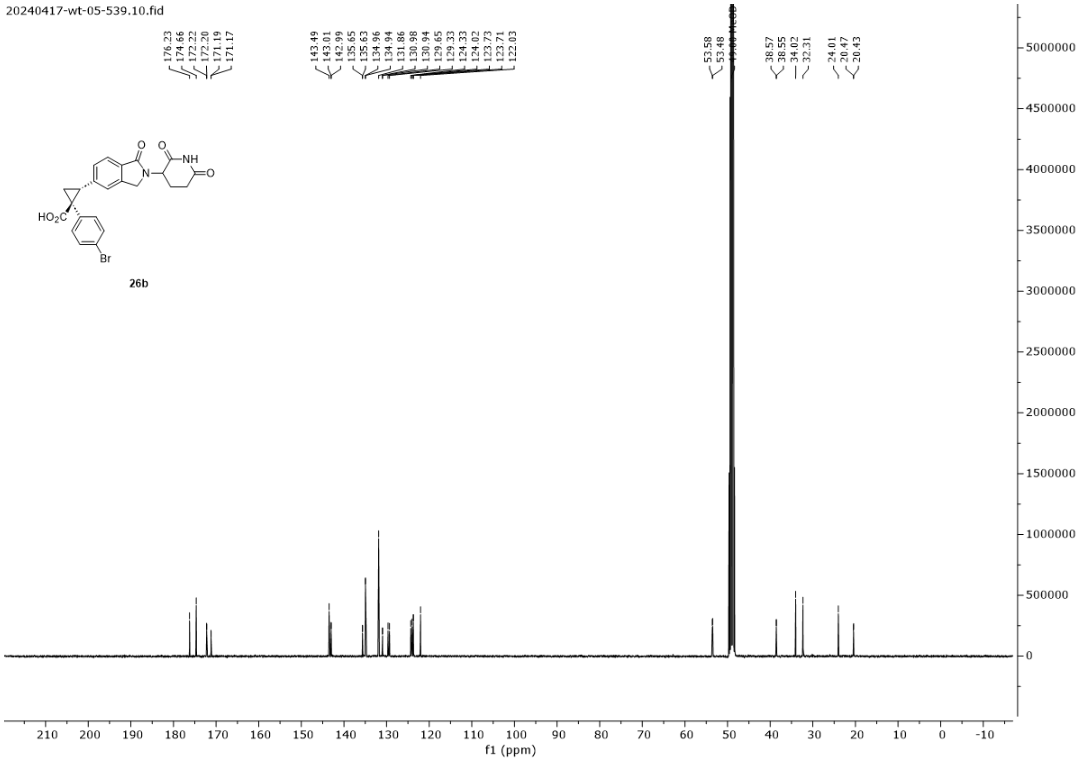

$^{13}C\{^{1}H\}$ NMR Spectrum for Compounds 27a and 27b.

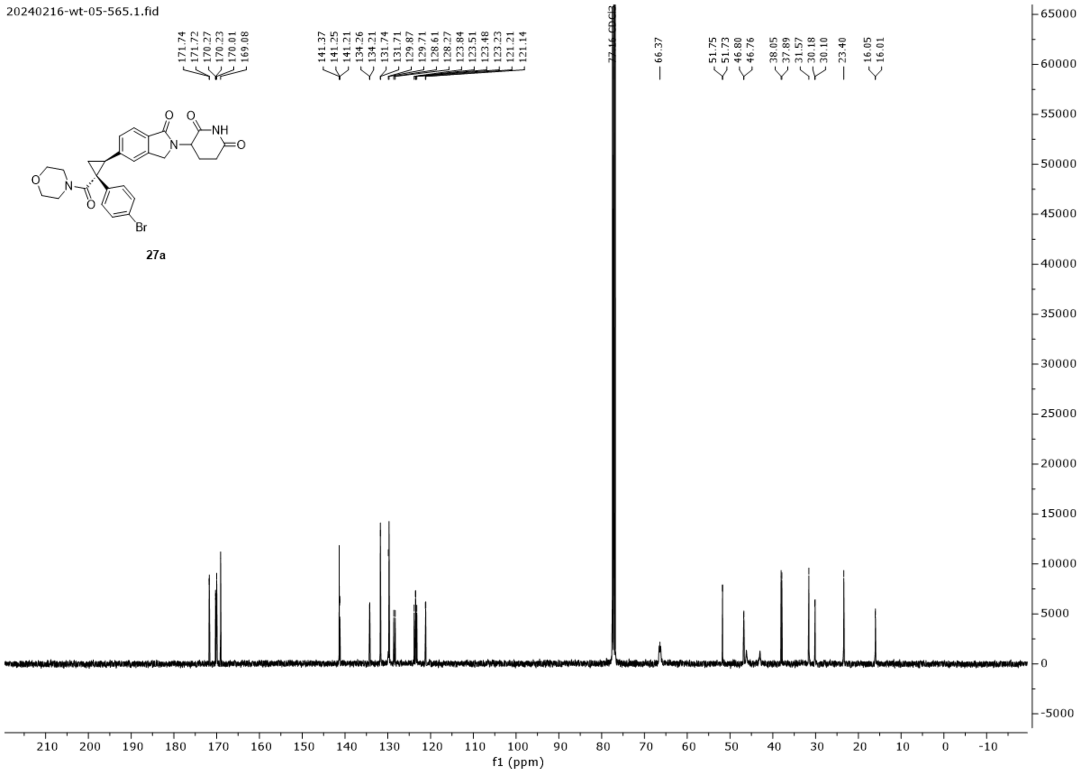

Section 5: Chromatographic Data

Chiral SFC Chromatograms

Racemic Chromatogram, 13

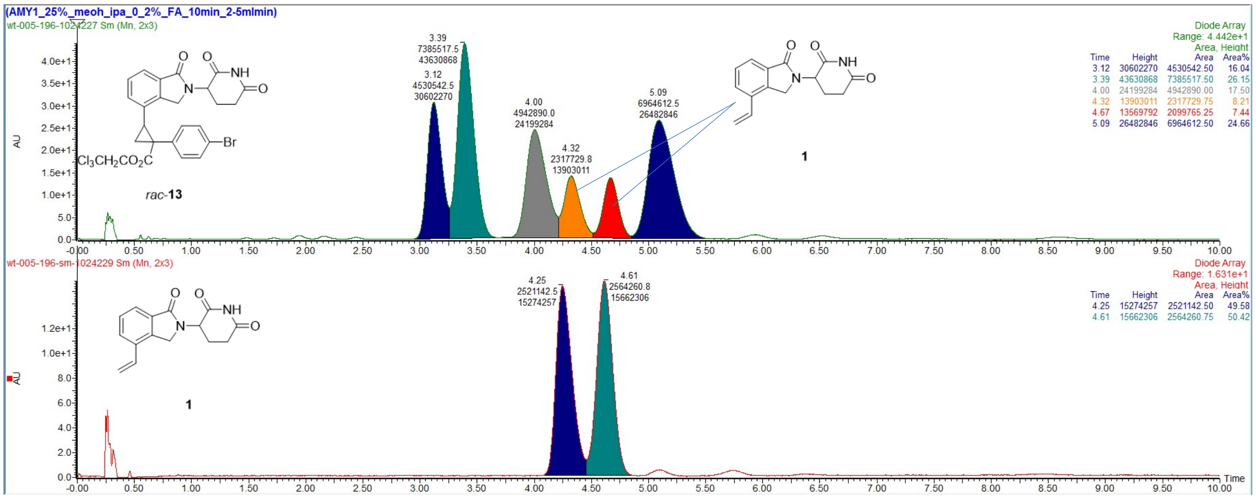

13a

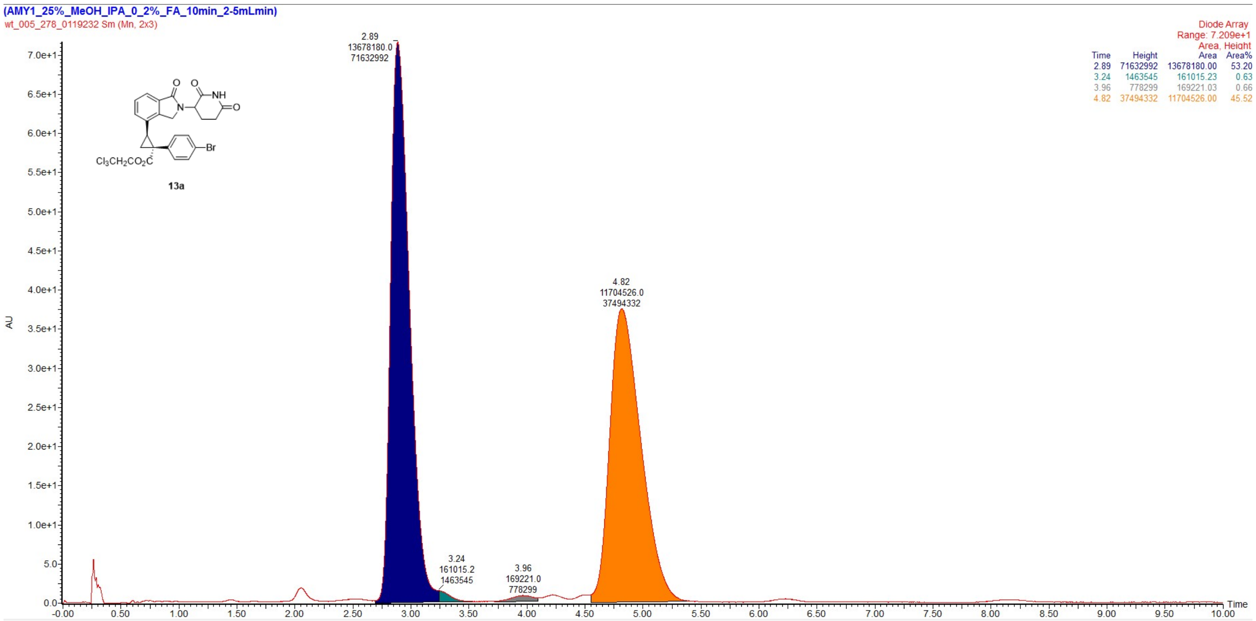

13b

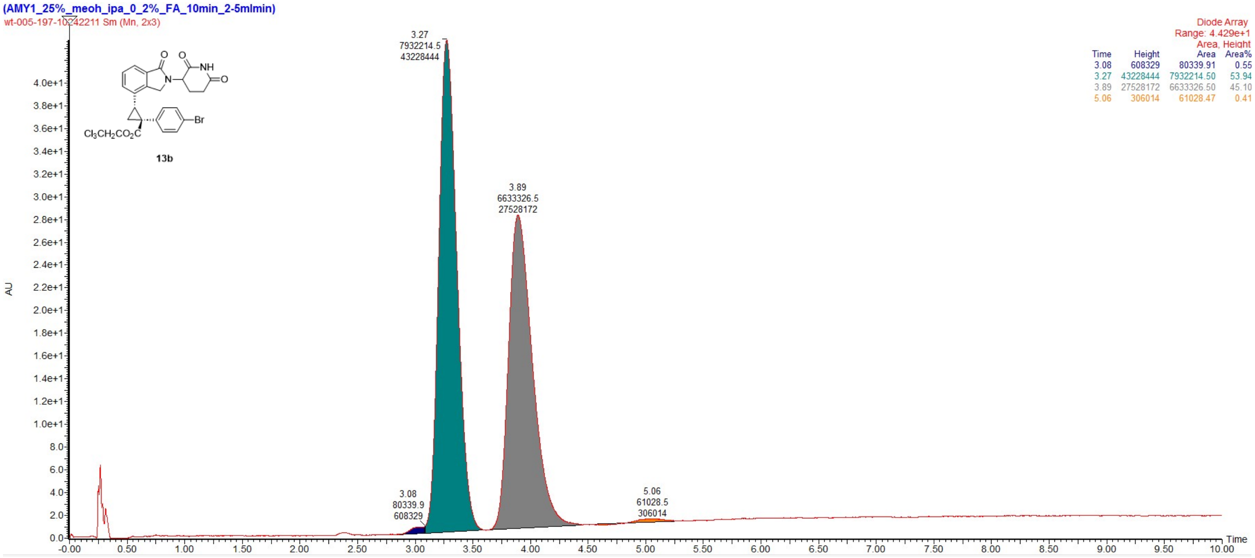

Racemic Chromatogram, 14

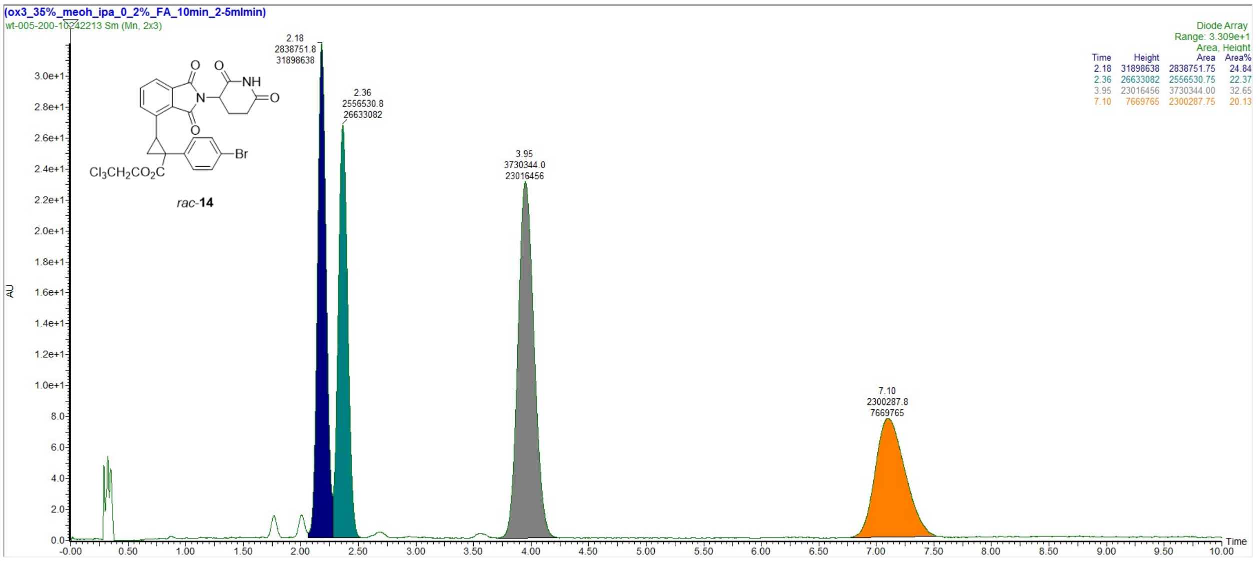

14a

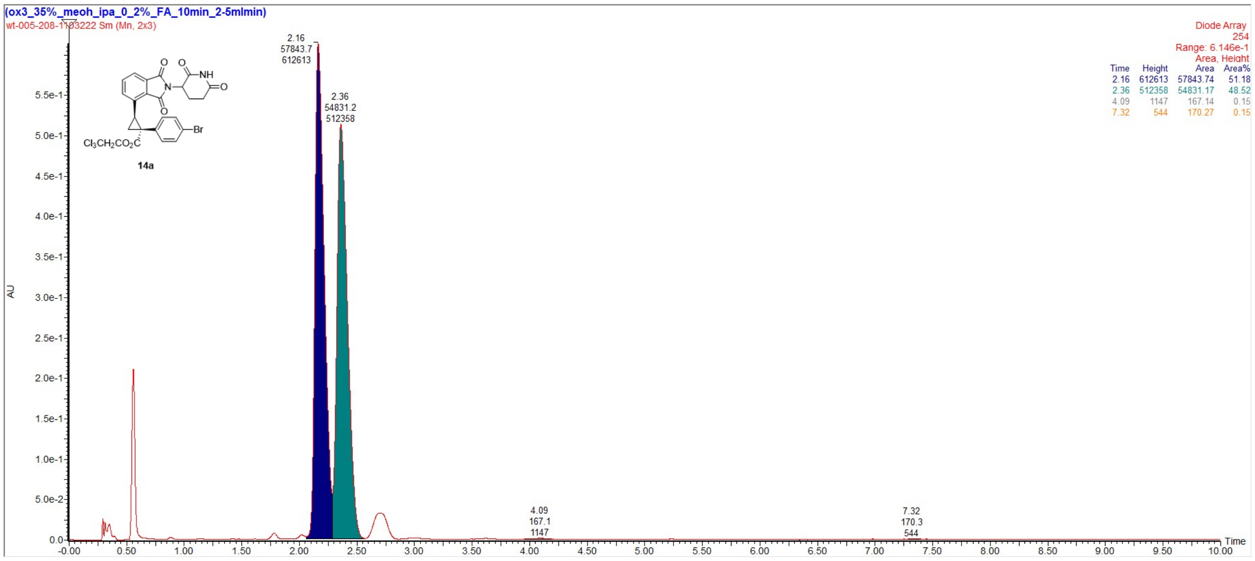
(ox3_35%_meoh_ipa_0_2%_FA_10min_2-5mlmin)
Diode Array
254
Range: 6.146e-1
Area, Height
Time Height Area Area%
2.16 612613 57843.74 51.18
2.36 512358 54831.17 48.52
4.09 1147 167.14 0.15
7.32 544 170.27 0.15
14a
AU
Time

14b

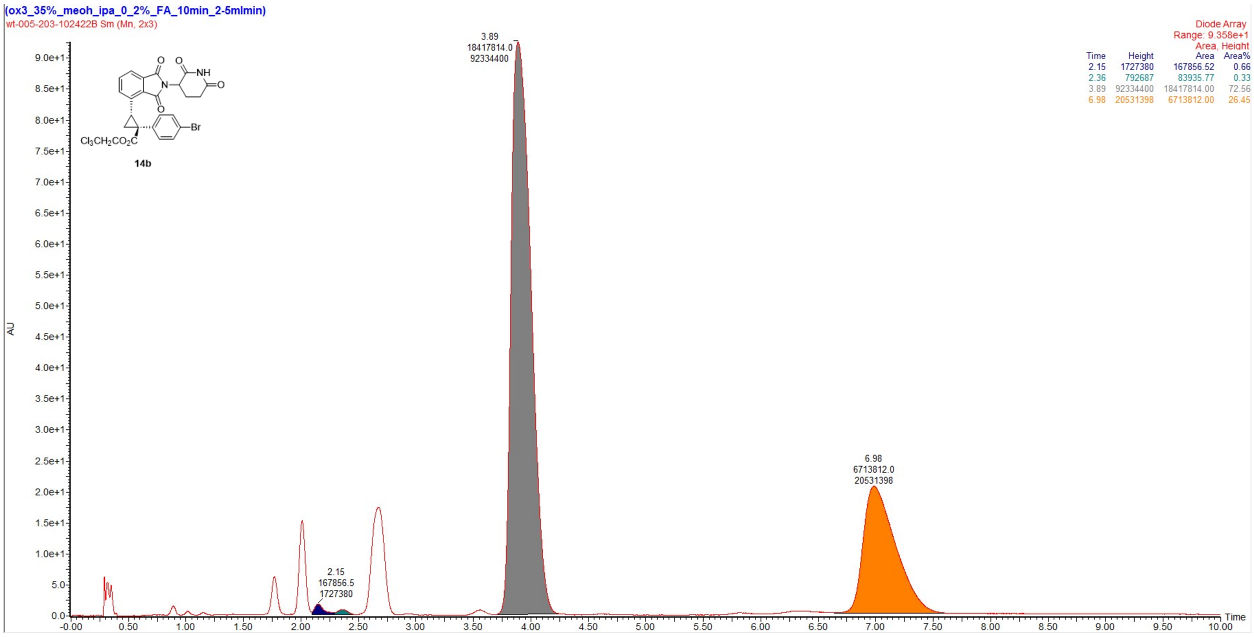
(ox3_35%_meoh_ipa_0_2%_FA_10min_2-5mlmin)
wt-005-203-102422B Sm (Mn, 2x3)
Diode Array
Range: 9.358e+1
Area, Height
Time Height Area Area%
2.15 1727380 167856.52 0.66
2.36 792687 83935.77 0.33
3.89 92334400 18417814.00 72.56
6.98 20531398 6713812.00 26.45
14b
AU
Time

Racemic Chromatogram, 15

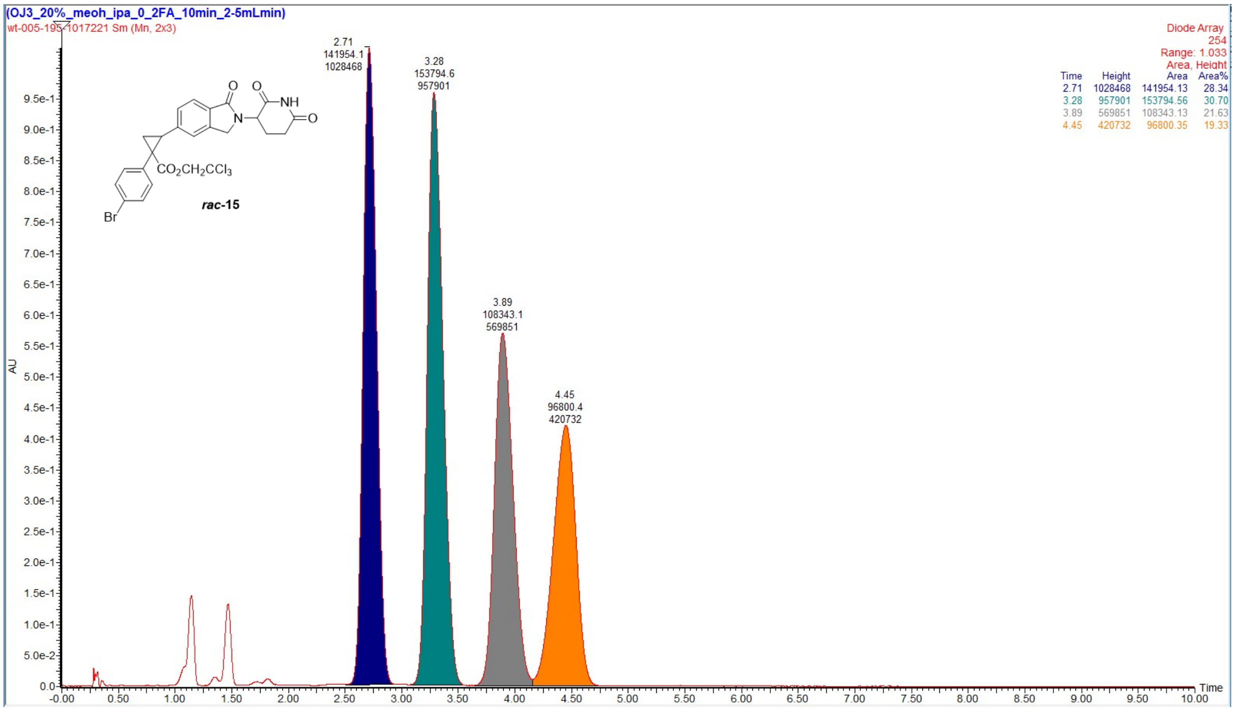

15a

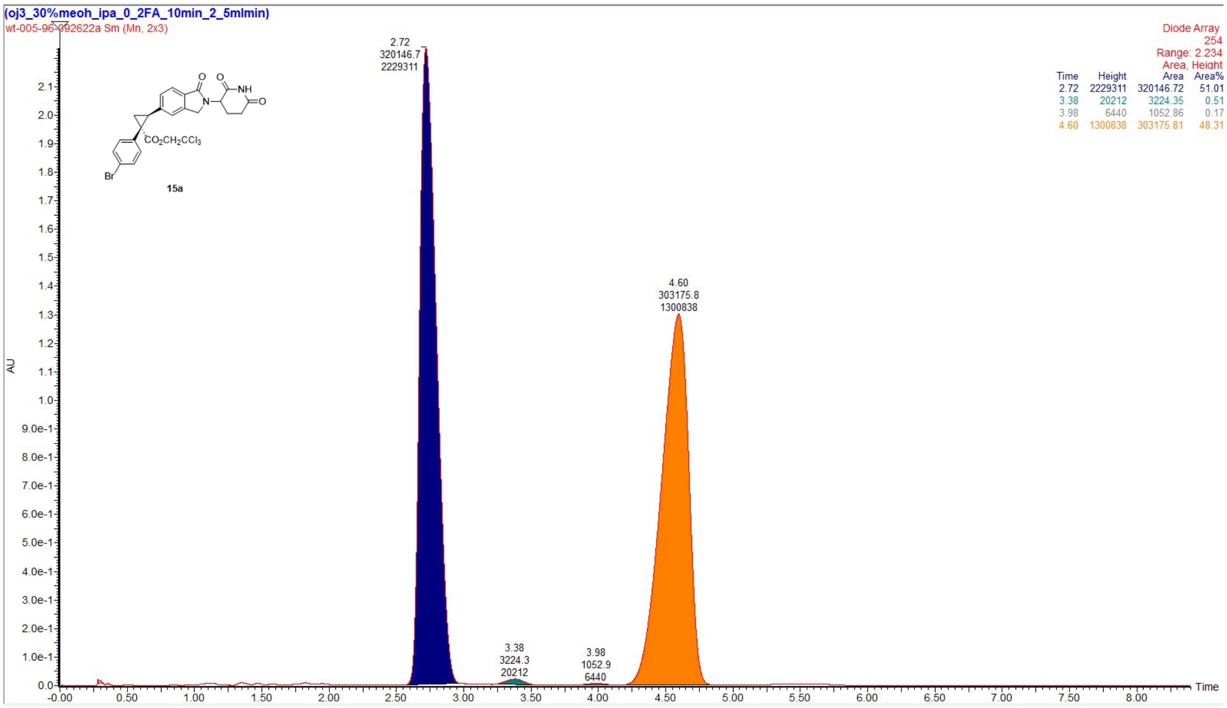

15b

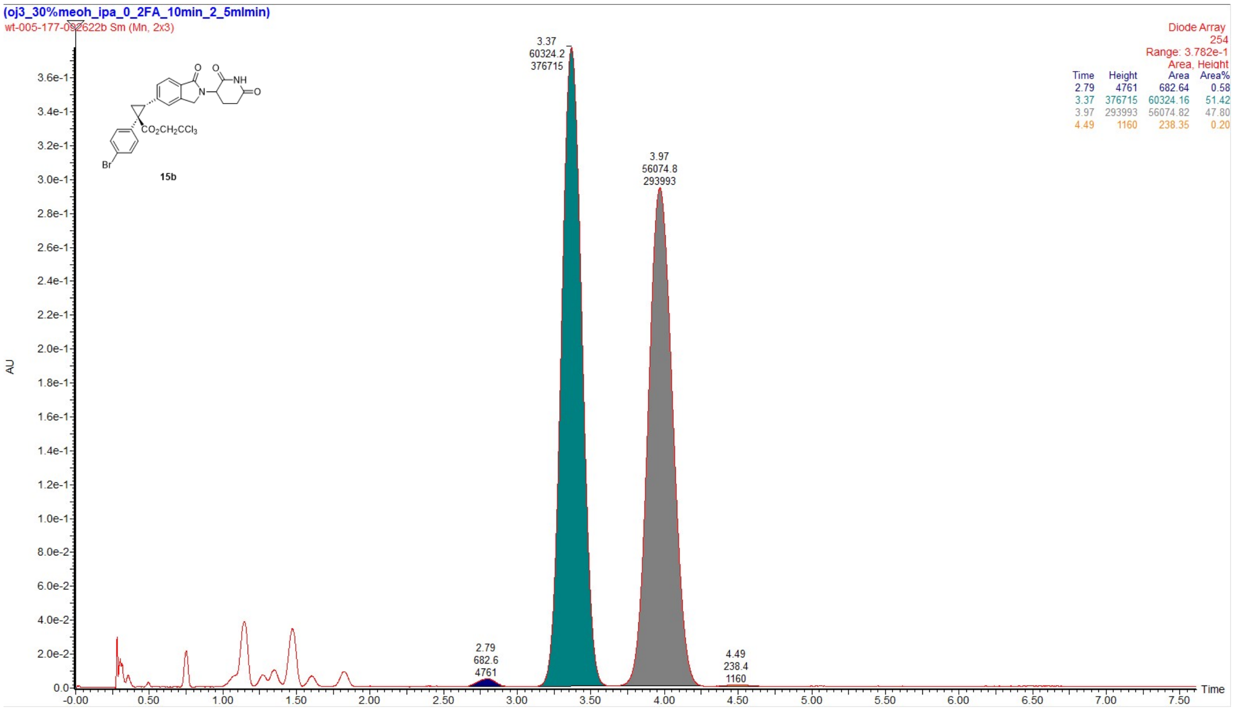

(oj3_30%meoh_ipa_0_2FA_10min_2_5mlmin)
wt-005-177-092622b Sm (Mn, 2x3)
Diode Array
254
Range: 3.782e-1
Area, Height
Time Height Area Area%
2.79 4761 682.64 0.58
3.37 376715 60324.16 51.42
3.97 293993 56074.82 47.80
4.49 1160 238.35 0.20
3.37
60324.2
376715
3.97
56074.8
293993
2.79
682.6
4761
4.49
238.4
1160
AU
Time
15b

***S*-15a**

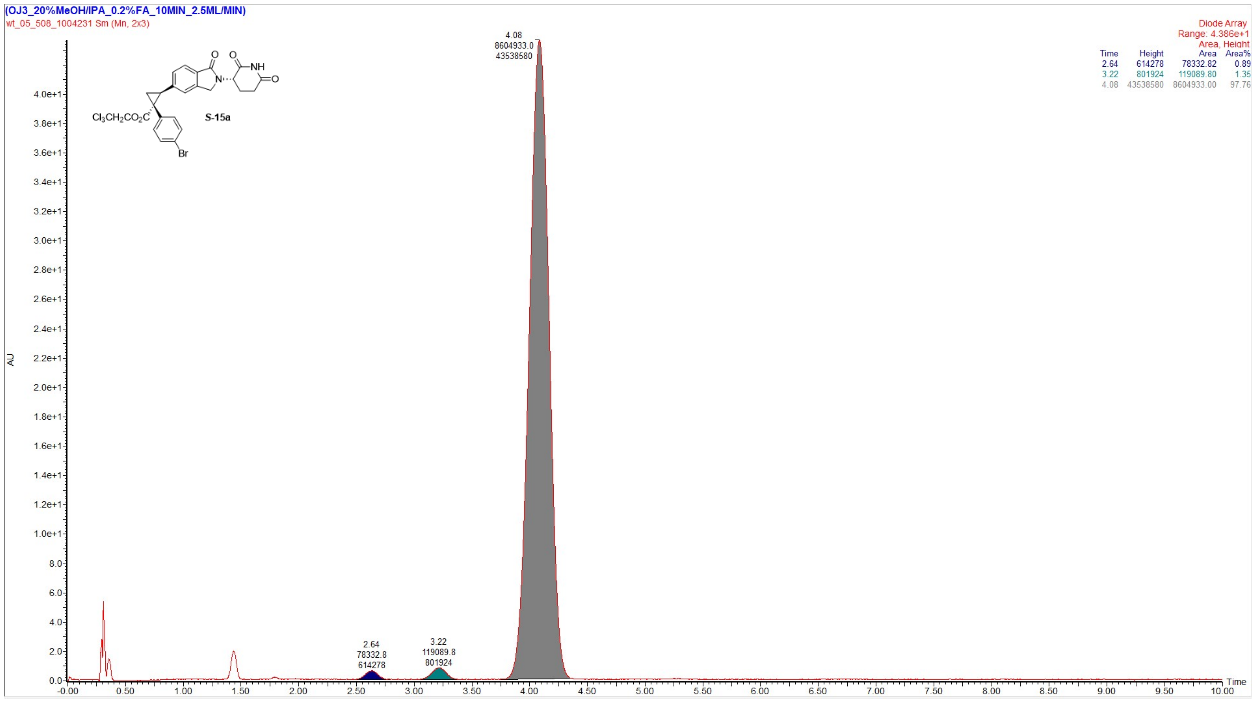

(OJ3_20%MeOH/IPA_0.2%FA_10MIN_2.5ML/MIN)
wt_05_508_1004231 Sm (Mn, 2x3)
Diode Array
Range: 4.386e+1
Area, Height
Time Height Area Area%
2.64 614278 78332.82 0.89
3.22 801924 119089.80 1.35
4.08 43538580 8604933.00 97.76
4.08
8604933.0
43538580
2.64
78332.8
614278
3.22
119089.8
801924
AU
Time
S-15a

S-15b

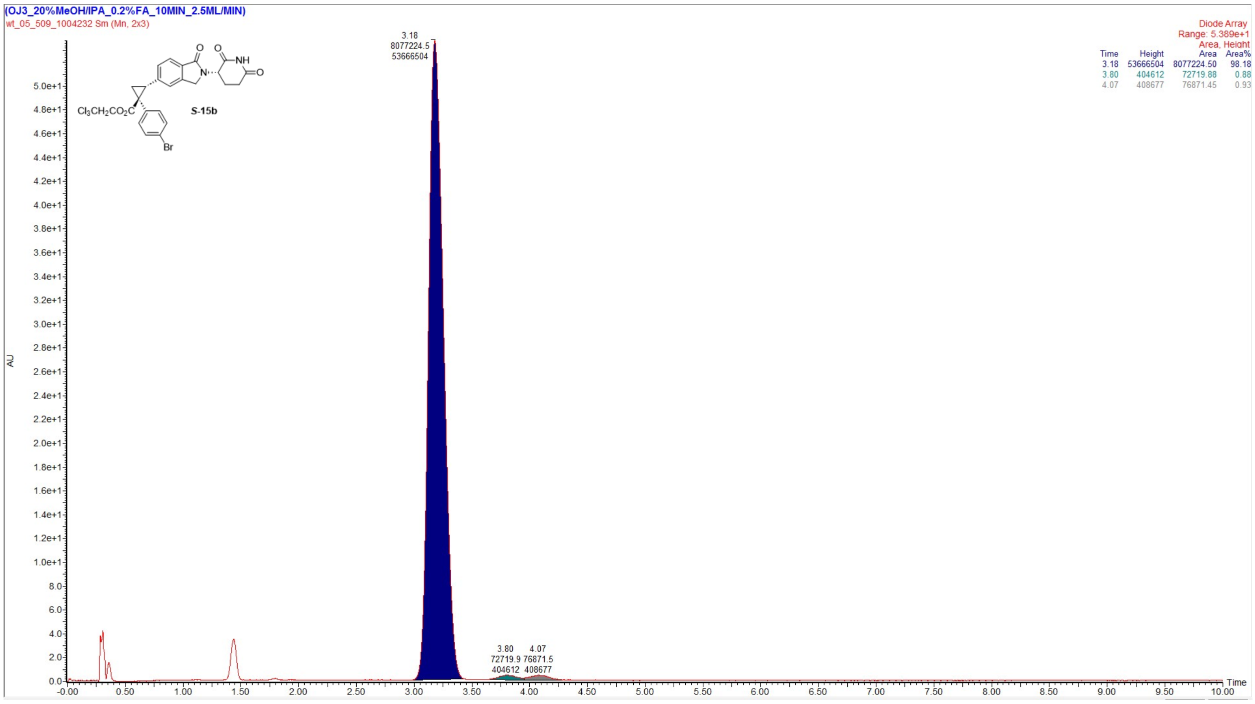
(OJ3_20%MeOH/IPA_0.2%FA_10MIN_2.5ML/MIN)
wt_05_509_1004232 Sm (Mn, 2x3)
Diode Array
Range: 5.389e+1
Area, Height
Time Height Area Area%
3.18 53666504 8077224.50 98.18
3.80 404612 72719.88 0.88
4.07 408677 76871.45 0.93
3.18
8077224.5
53666504
3.80
72719.9
404612
4.07
76871.5
408677
S-15b
AU
Time

R-15a

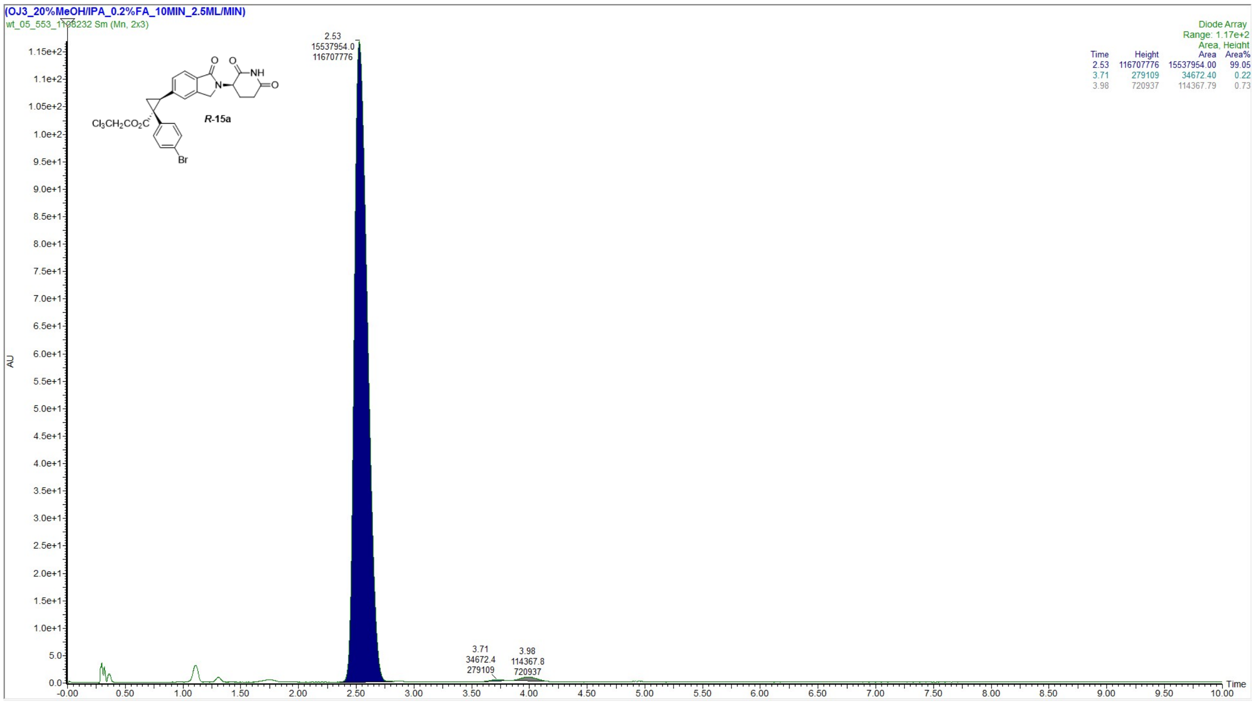
(OJ3_20%MeOH/IPA_0.2%FA_10MIN_2.5ML/MIN)
Diode Array
Range: 1.17e+2
Area, Height
Time Height Area Area%
2.53 116707776 15537954.00 99.05
3.71 279109 34672.40 0.22
3.98 720937 114367.79 0.73
2.53
15537954.0
116707776
3.71
34672.4
279109
3.98
114367.8
720937
R-15a
AU
Time

R-15b

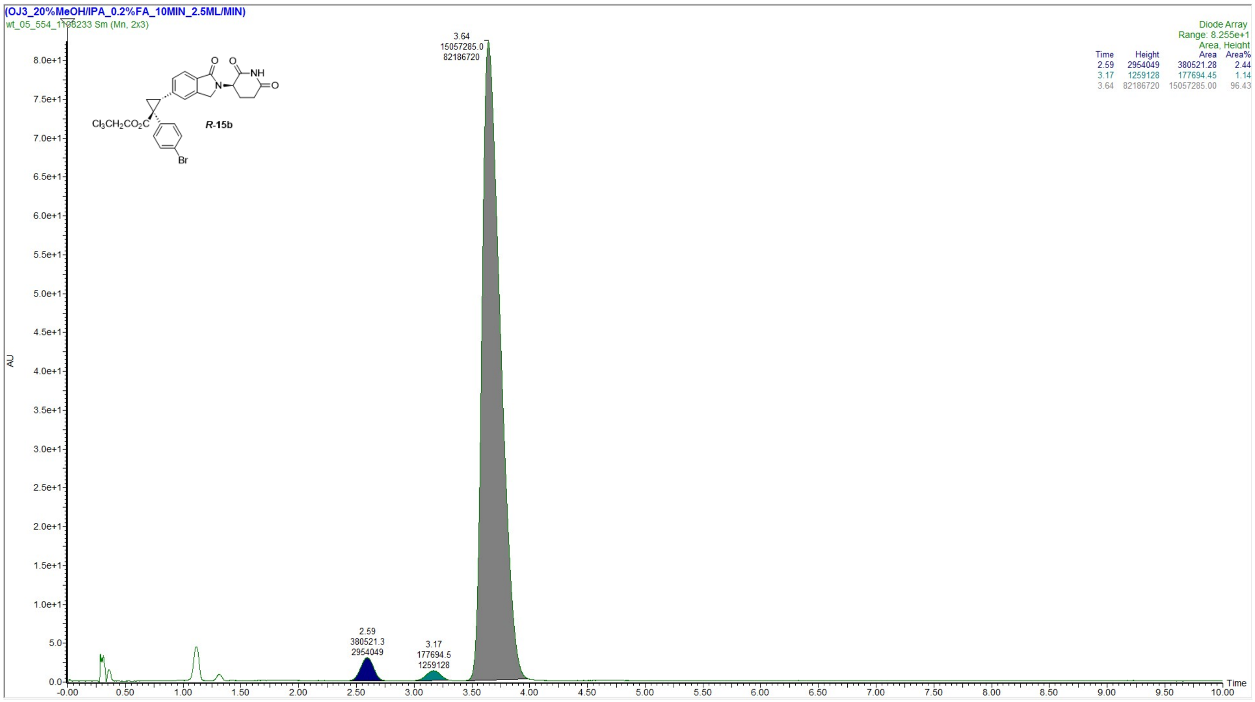

Racemic Chromatogram, 16

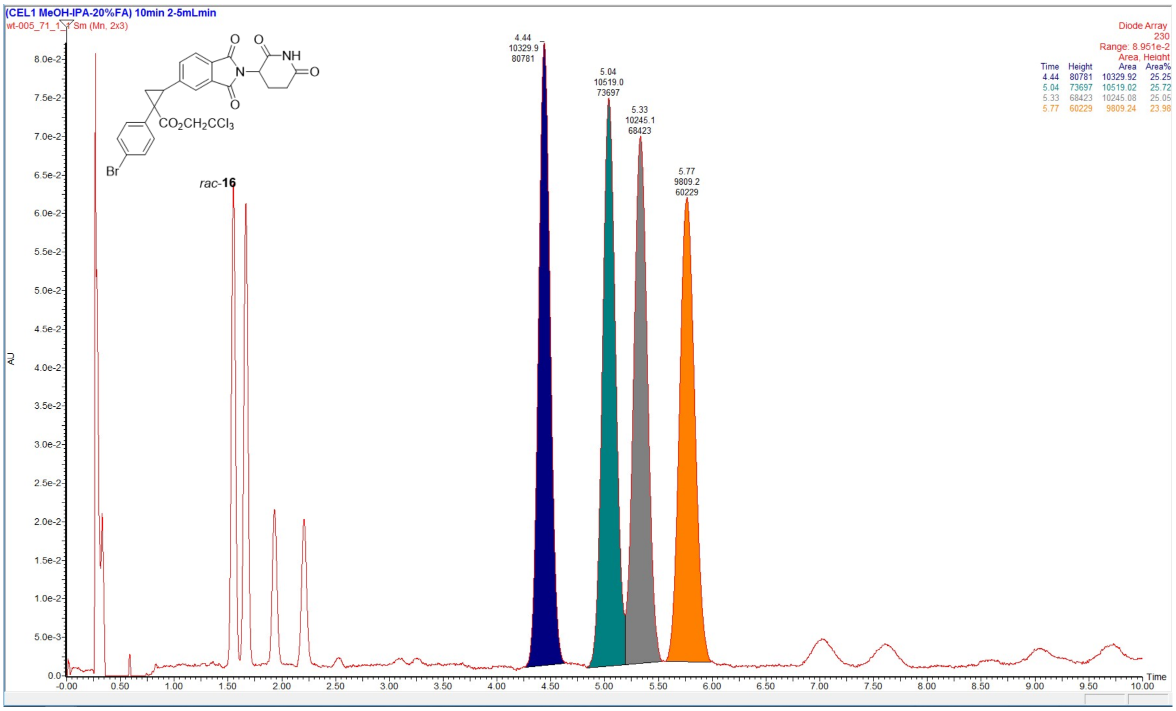

16a

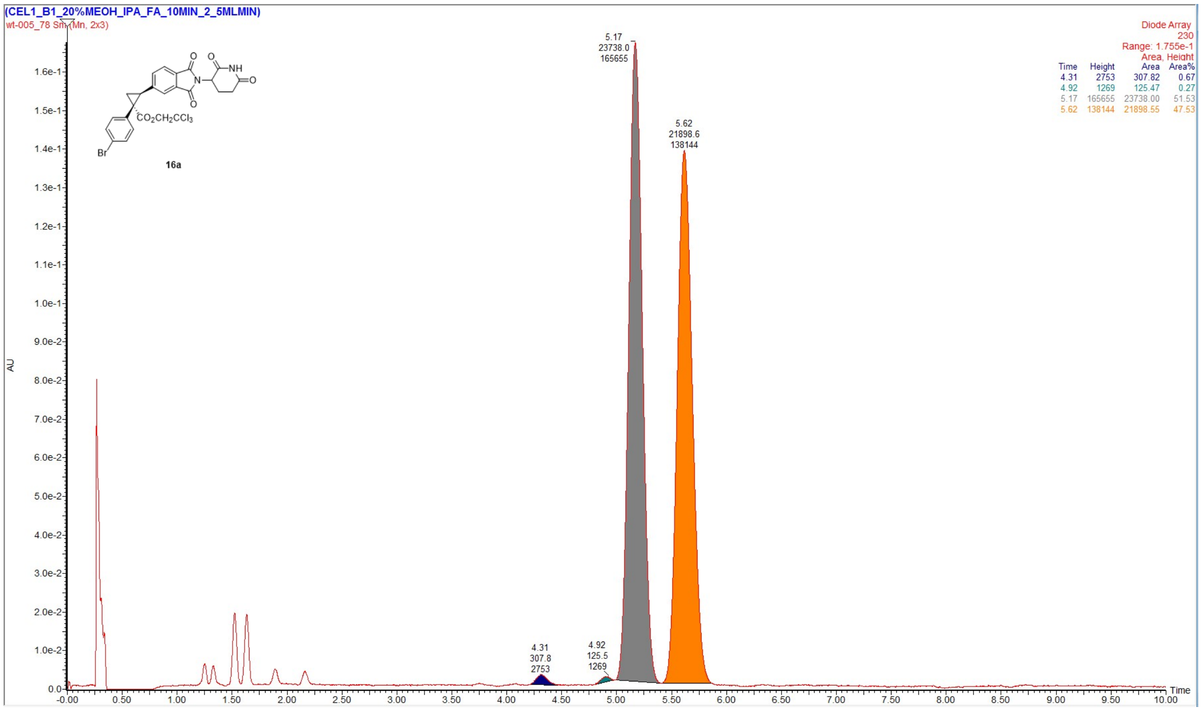
(CEL1_B1_20%MEOH_IPA_FA_10MIN_2_5MLMIN)
wt-005_78 Sm (Mn, 2x3)
Diode Array
230
Range: 1.755e-1
Area, Height
Time Height Area Area%
4.31 2753 307.82 0.67
4.92 1269 125.47 0.27
5.17 165655 23738.00 51.53
5.62 138144 21898.55 47.53
16a
AU
Time

16b

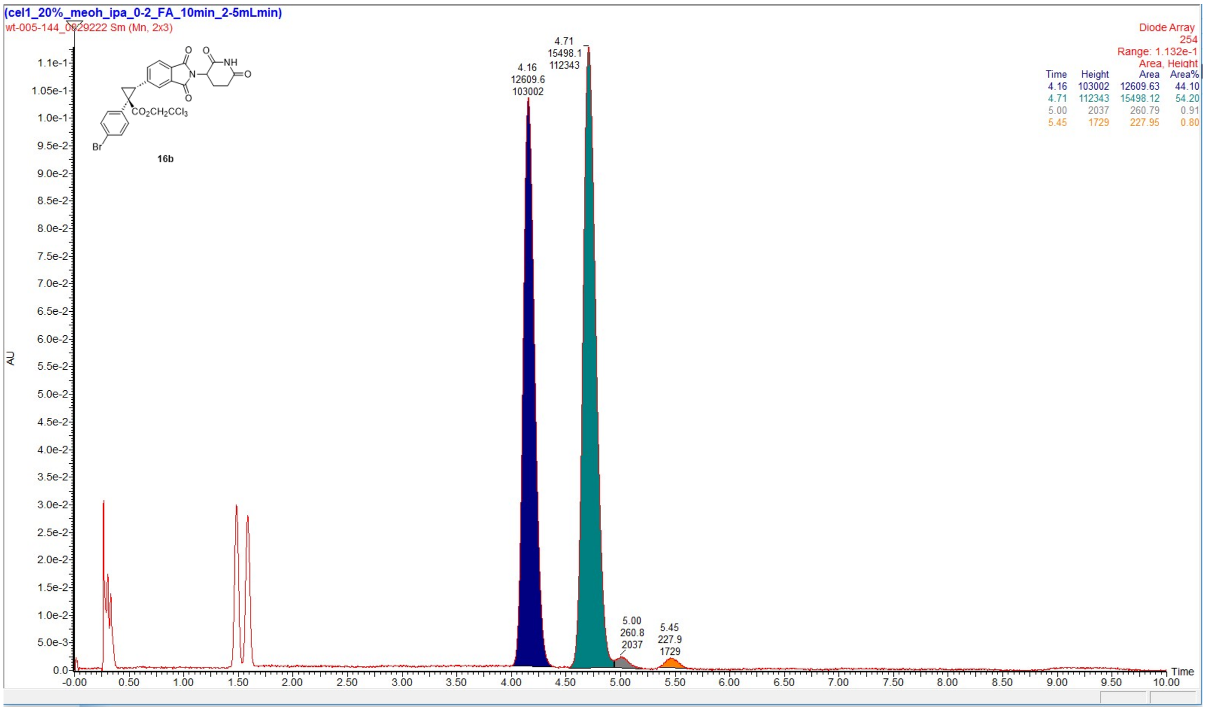
(cel1_20%_meoh_ipa_0-2_FA_10min_2-5mLmin)
wt-005-144_0929222 Sm (Mn, 2x3)
Diode Array
254
Range: 1.132e-1
Area, Height
Time Height Area Area%
4.16 103002 12609.63 44.10
4.71 112343 15498.12 54.20
5.00 2037 260.79 0.91
5.45 1729 227.95 0.80
16b
AU
Time

Racemic Chromatogram, 17

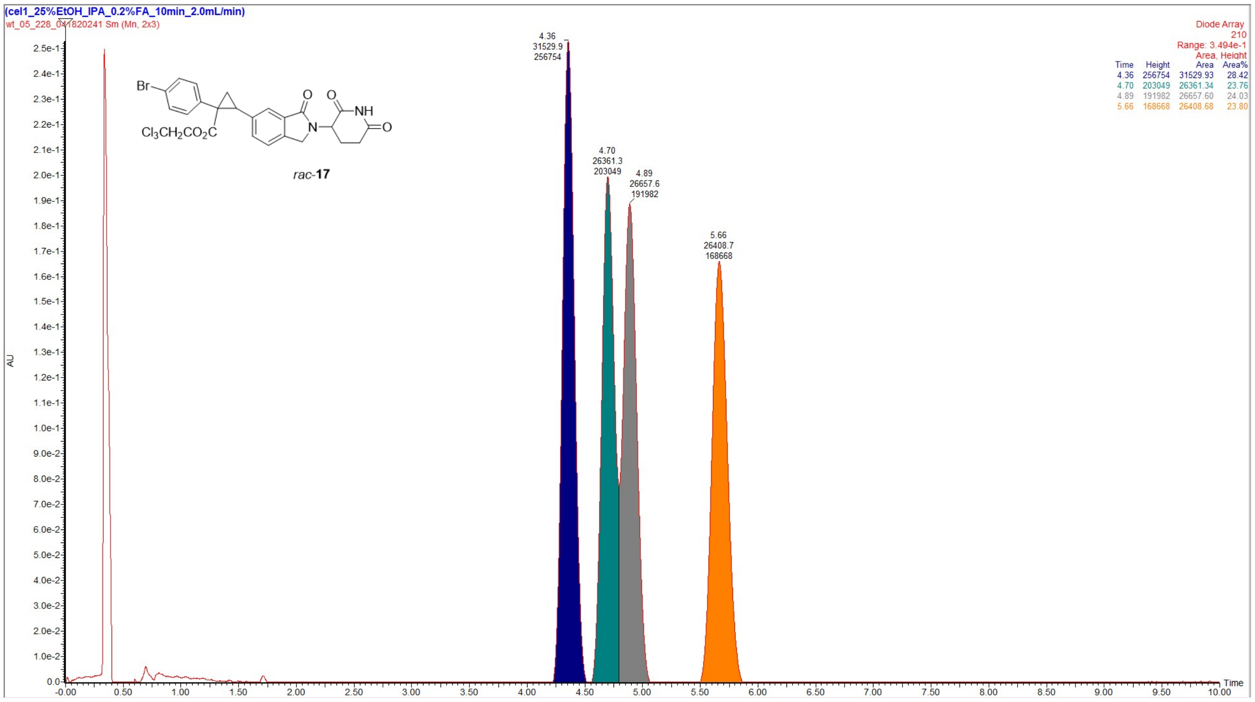

17a

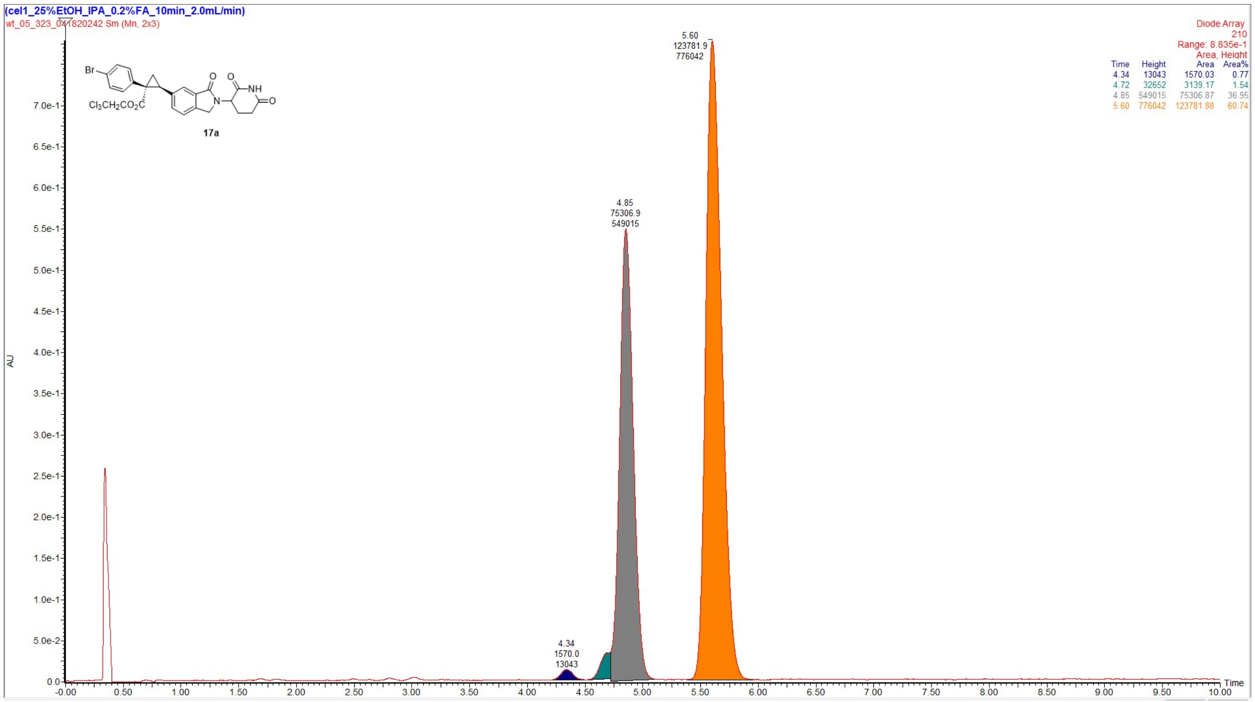

17b

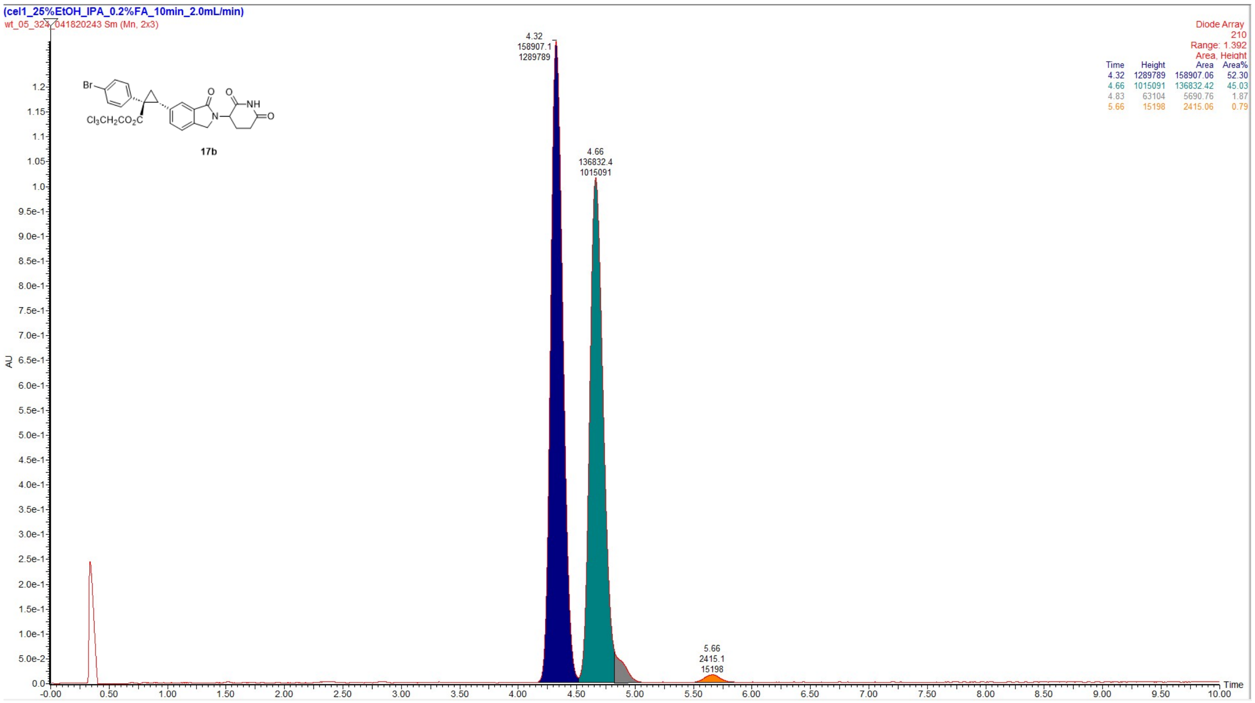

Racemic Chromatogram, 18

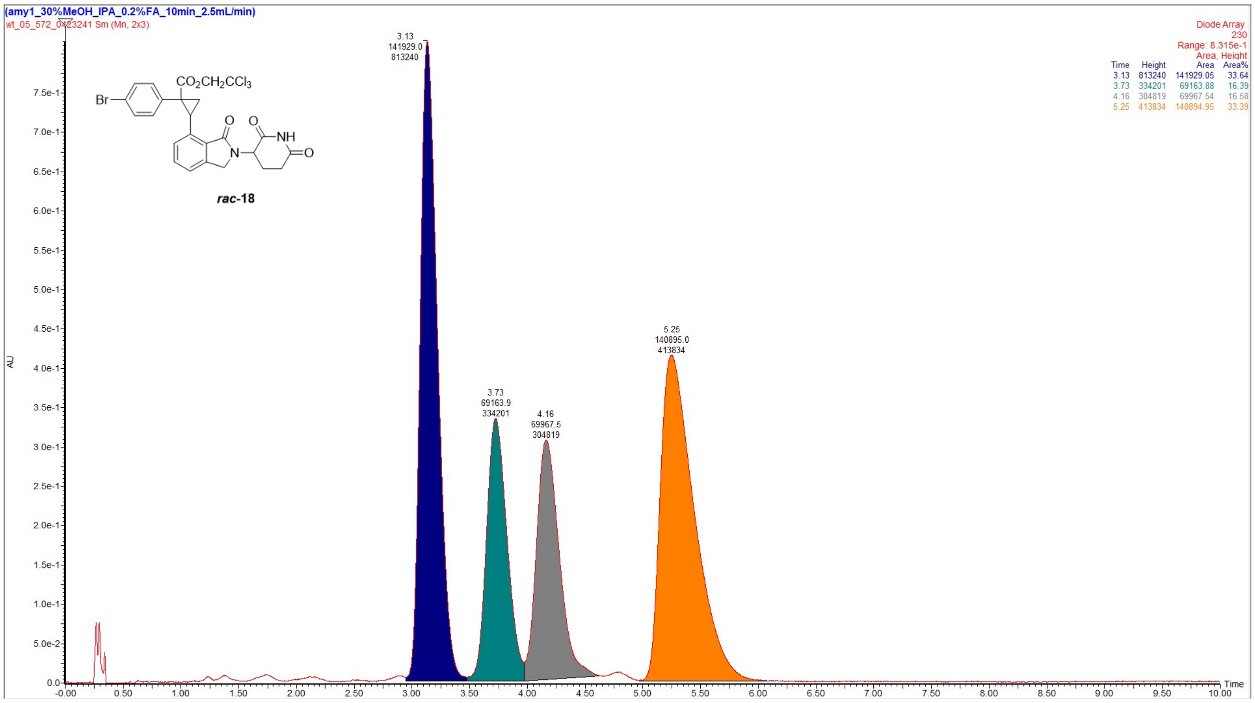

18a

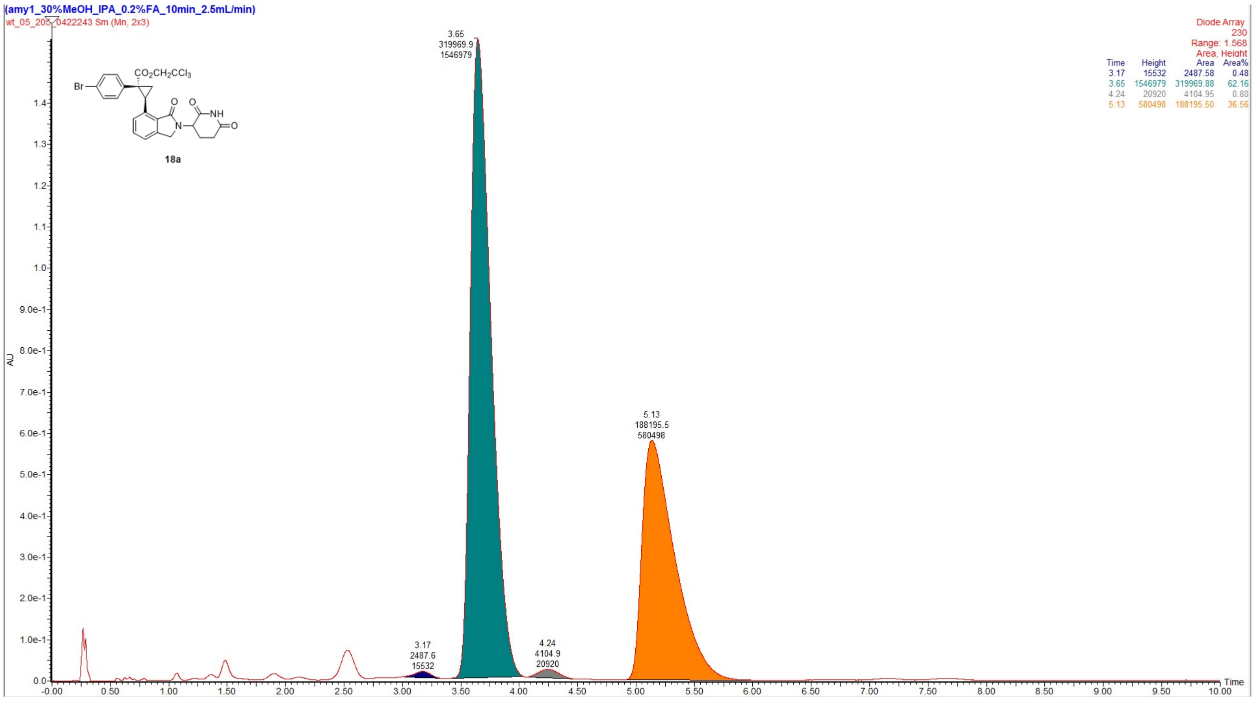

amy1_30%MeOH_IPA_0.2%FA_10min_2.5mL/min)
wt_05_205_0422243 Sm (Mn, 2x3)
18a
Diode Array
230
Range: 1.568
Area, Height
Time Height Area Area%
3.17 15532 2487.58 0.48
3.65 1546979 319969.88 62.16
4.24 20920 4104.95 0.80
5.13 580498 188195.50 36.56
3.65
319969.9
1546979
5.13
188195.5
580498
3.17
2487.6
15532
4.24
4104.9
20920
AU
Time

18b

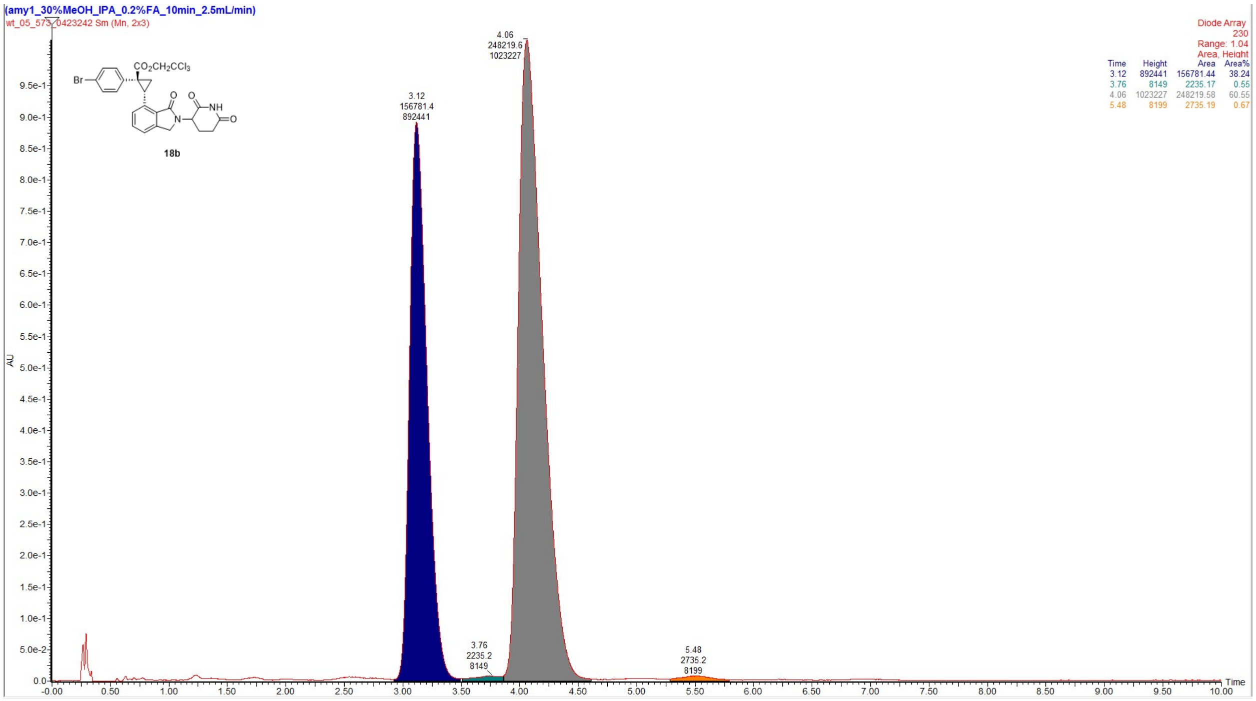

amy1_30%MeOH_IPA_0.2%FA_10min_2.5mL/min)
wt_05_573_0423242 Sm (Mn, 2x3)
18b
Diode Array
230
Range: 1.04
Area, Height
Time Height Area Area%
3.12 892441 156781.44 38.24
3.76 8149 2235.17 0.55
4.06 1023227 248219.58 60.55
5.48 8199 2735.19 0.67
4.06
248219.6
1023227
3.12
156781.4
892441
3.76
2235.2
8149
5.48
2735.2
8199
AU
Time

Racemic Chromatogram, 19

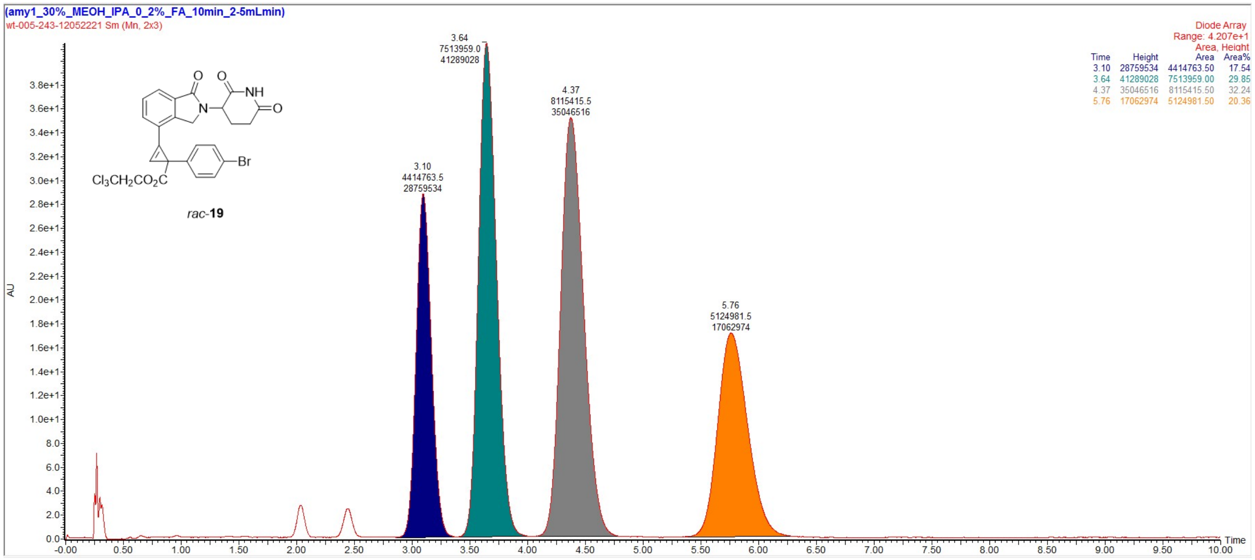

19a

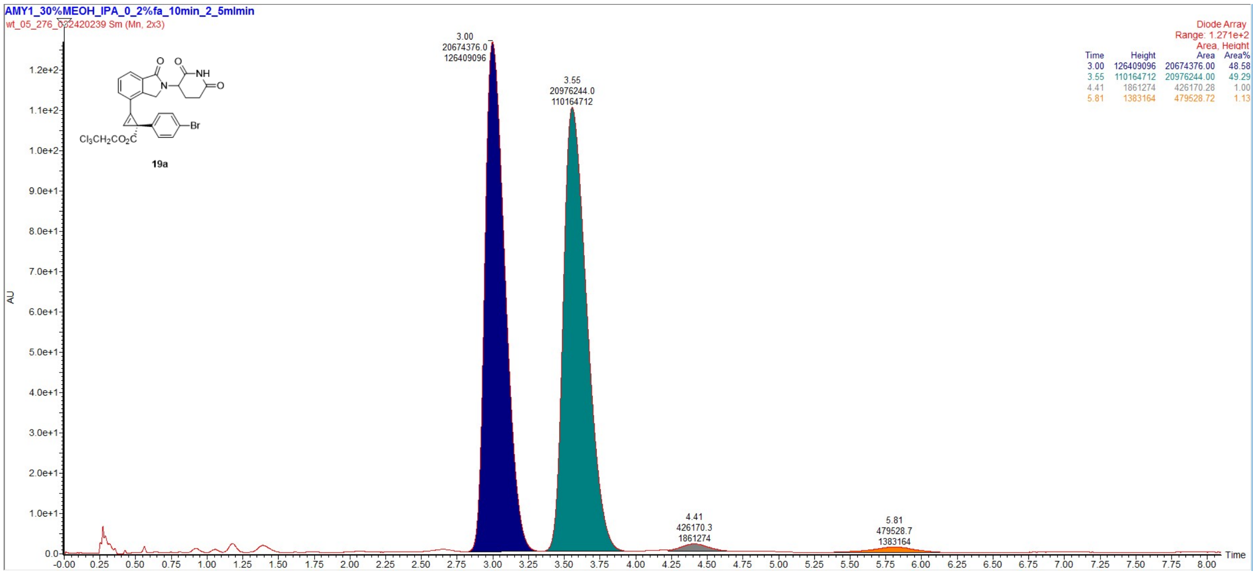

19b

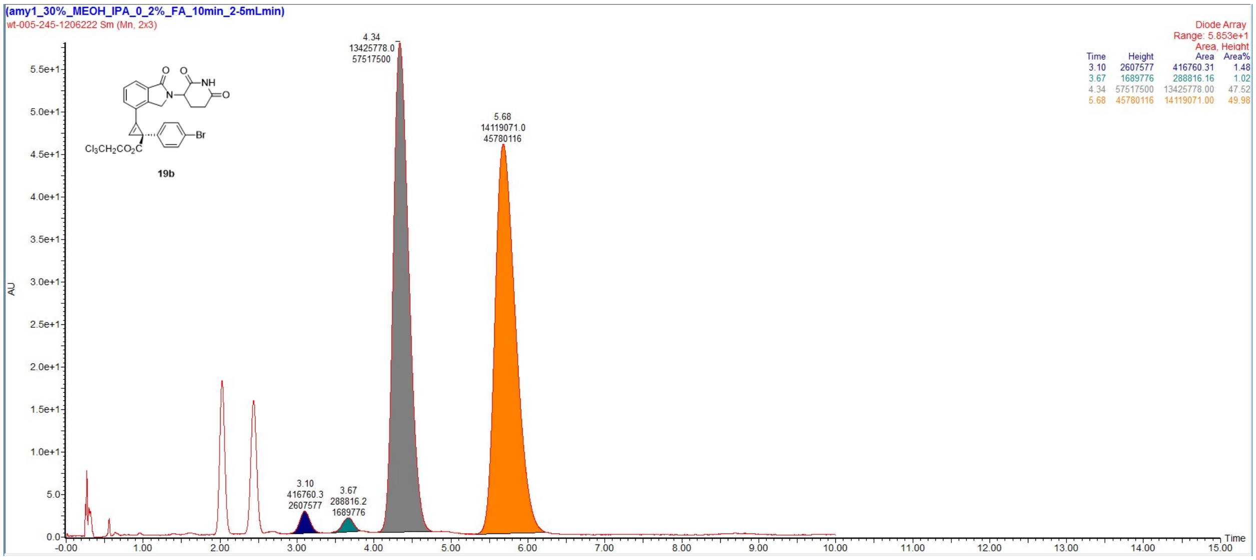

Racemic Chromatogram, 20

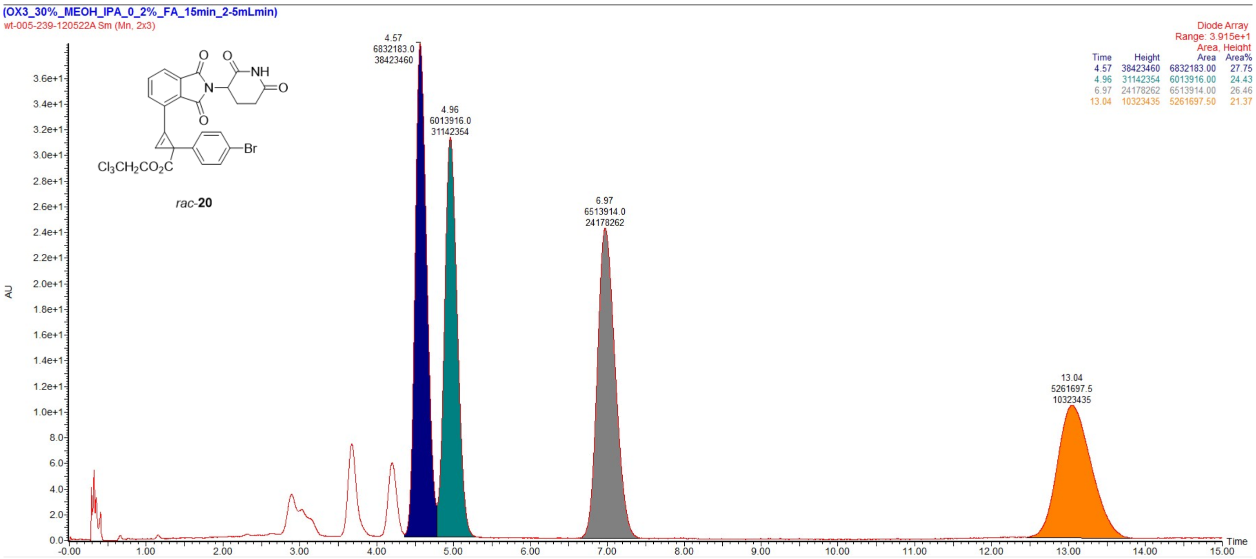

20a

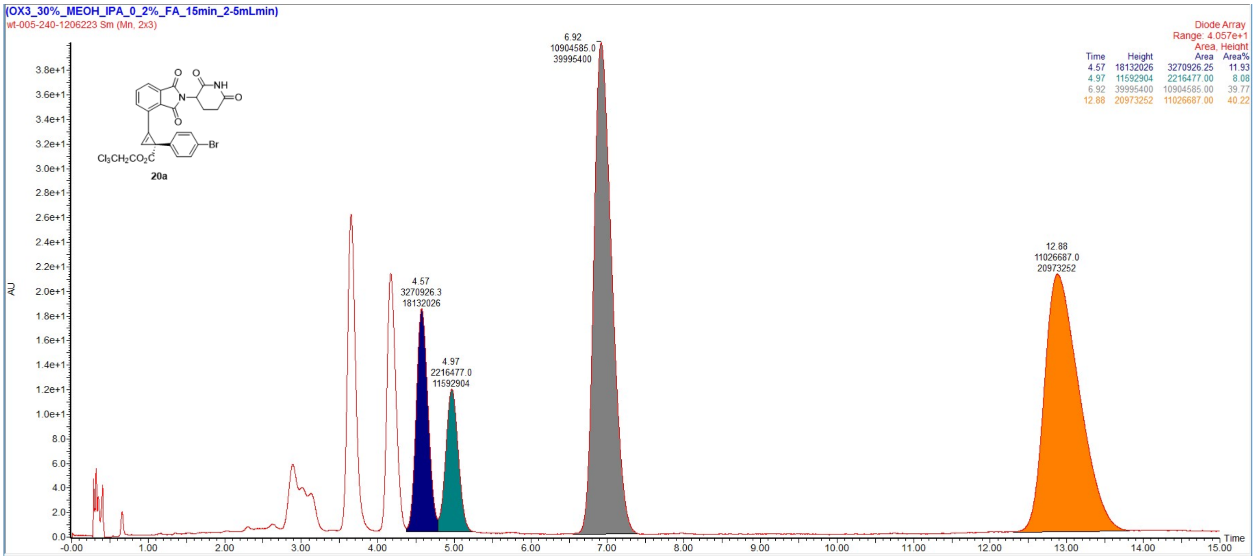
(OX3_30%_MEOH_IPA_0_2%_FA_15min_2-5mLmin)
wt-005-240-1206223 Sm (Mn, 2x3)
Diode Array
Range: 4.057e+1
Area, Height
Time Height Area Area%
4.57 18132026 3270926.25 11.93
4.97 11592904 2216477.00 8.08
6.92 39995400 10904585.00 39.77
12.88 20973252 11026687.00 40.22
20a

20b

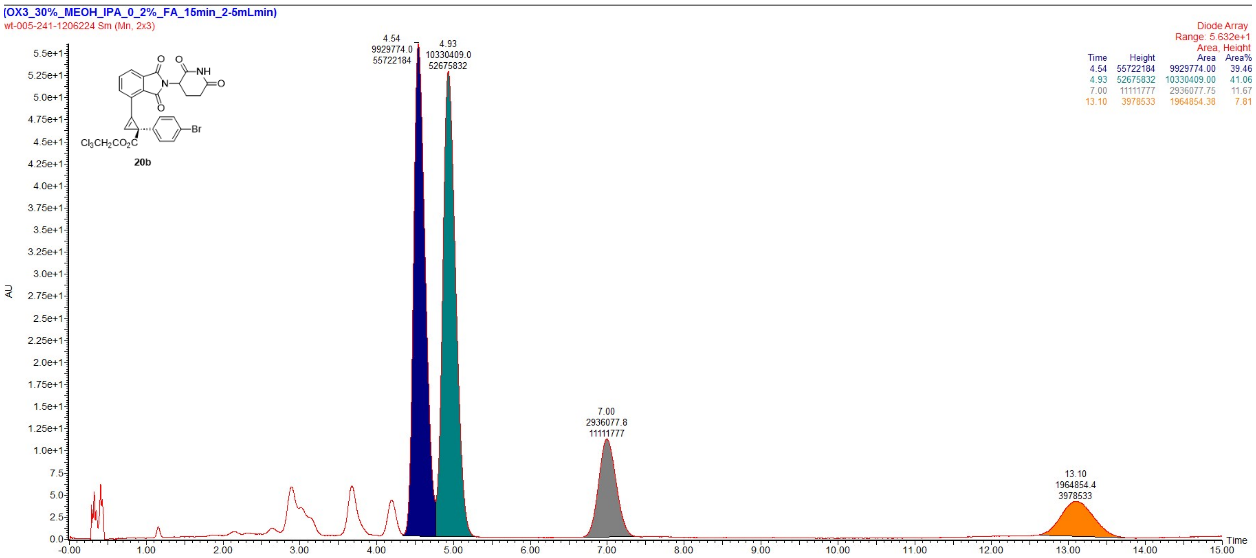
(OX3_30%_MEOH_IPA_0_2%_FA_15min_2-5mLmin)
wt-005-241-1206224 Sm (Mn, 2x3)
Diode Array
Range: 5.632e+1
Area, Height
Time Height Area Area%
4.54 55722184 9929774.00 39.46
4.93 52675832 10330409.00 41.06
7.00 11111777 2936077.75 11.67
13.10 3978533 1964854.38 7.81
20b

Racemic Chromatogram, 21

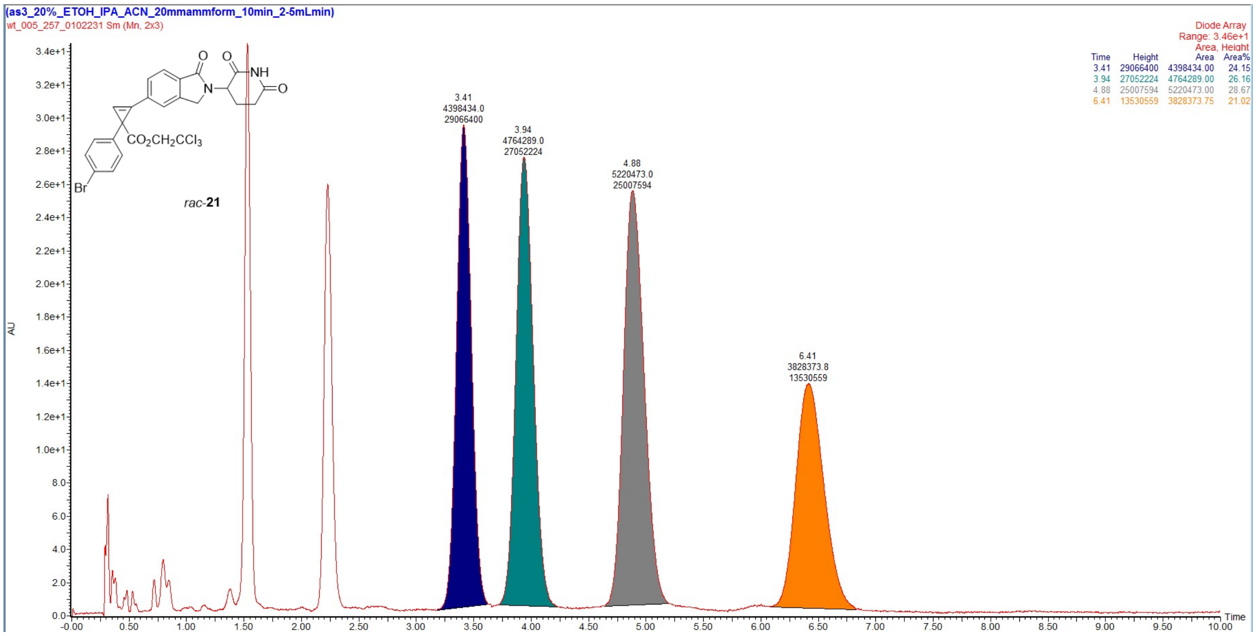

21a

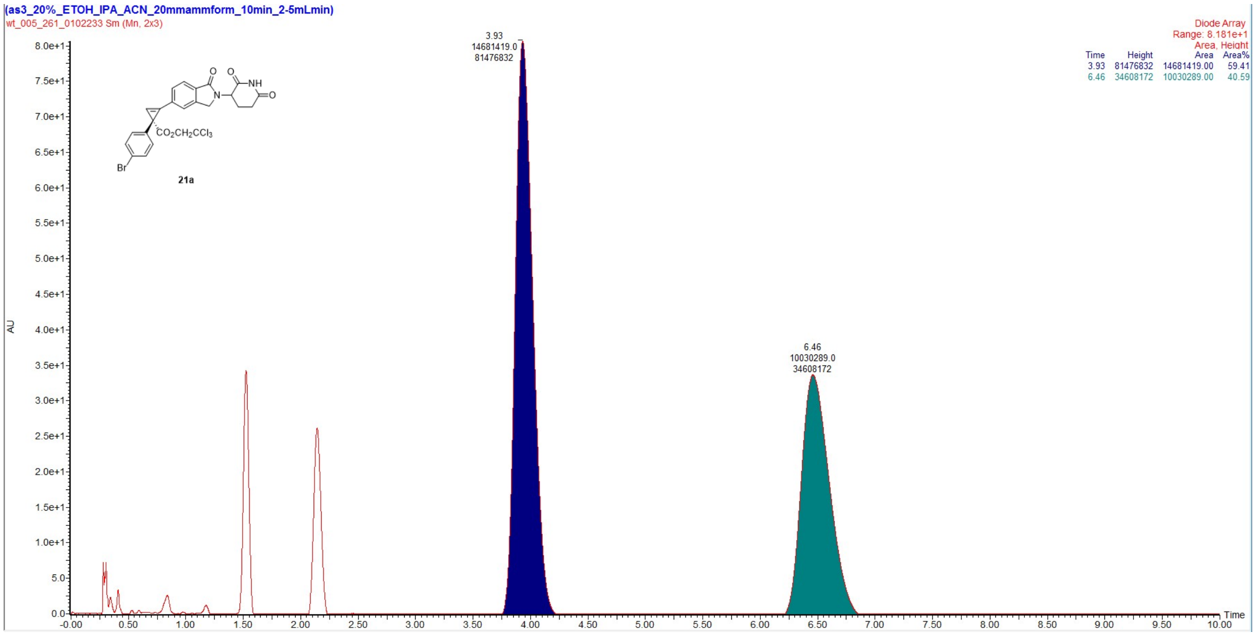

21b

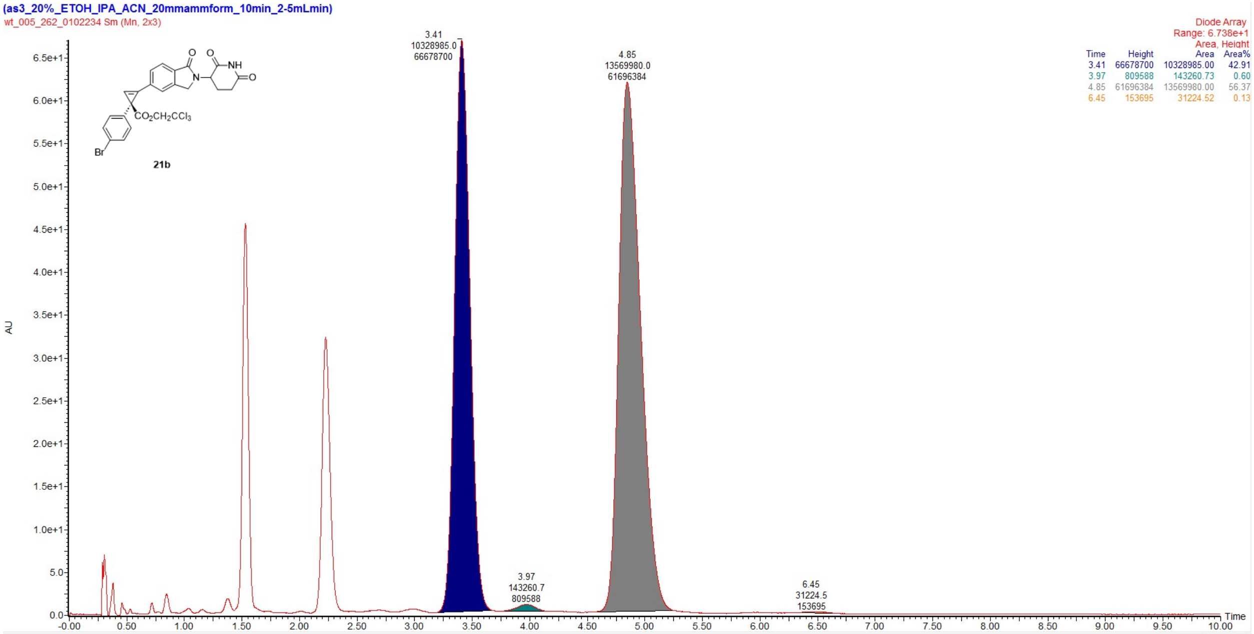

Racemic Chromatogram, 22

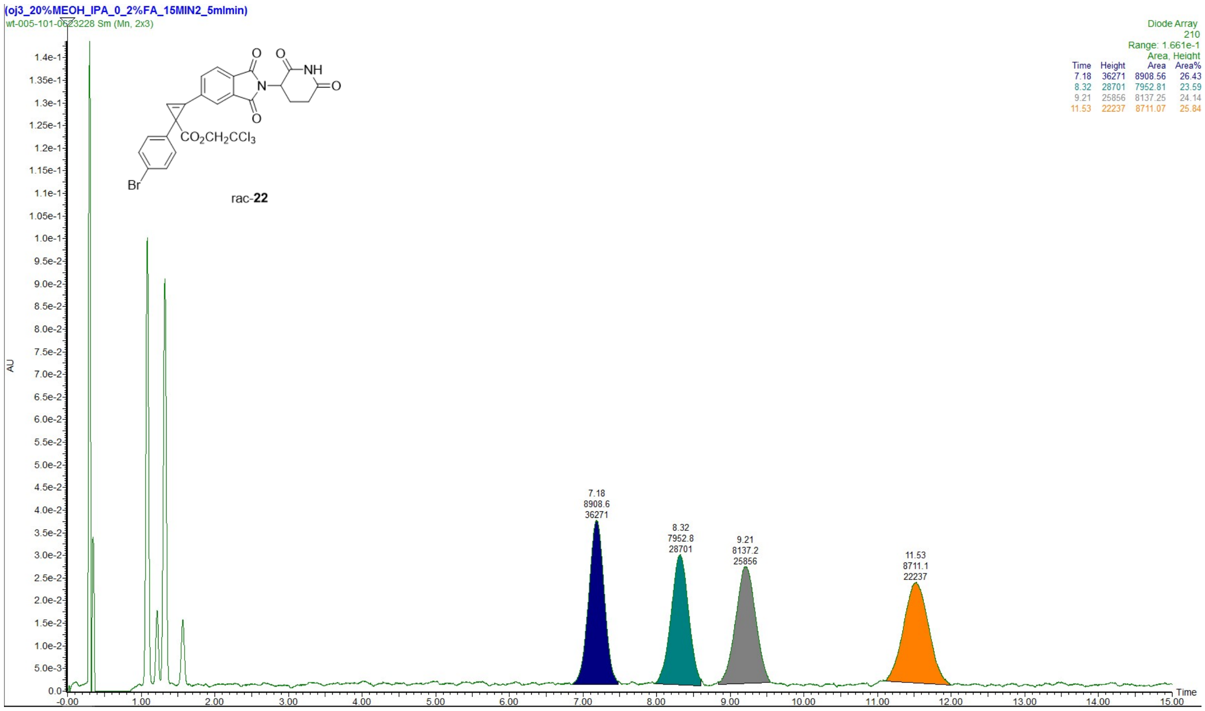

22a

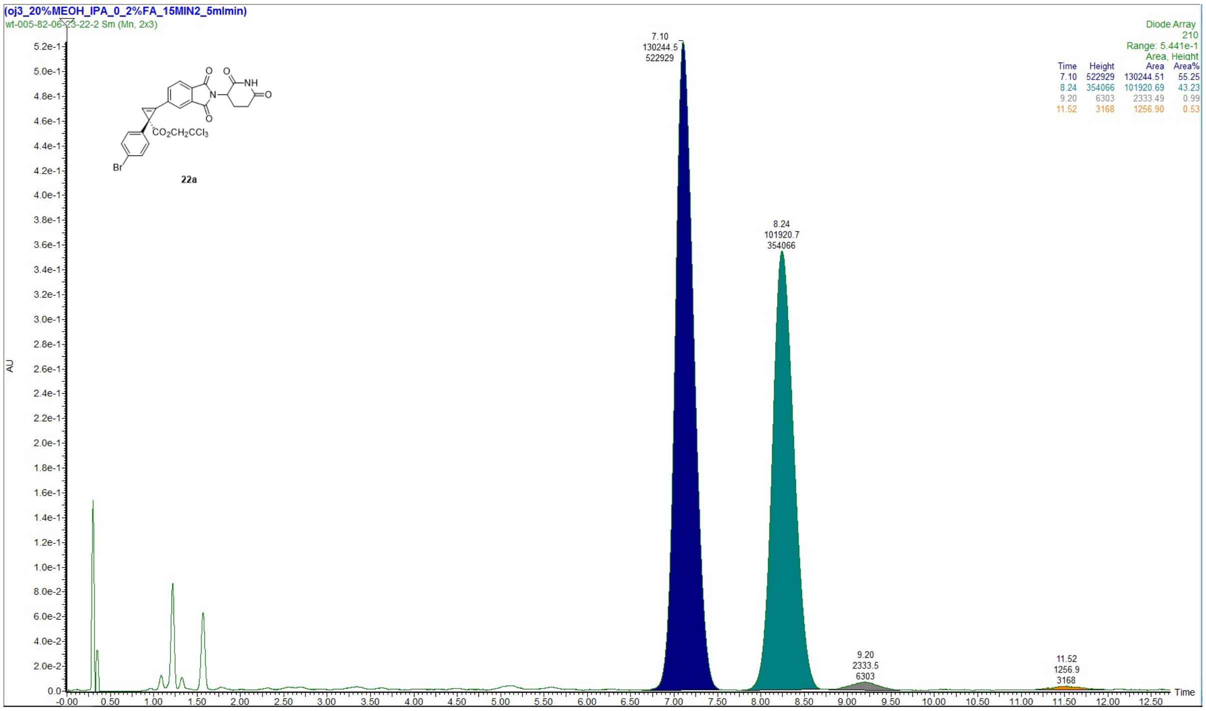
(oj3_20%MEOH_IPA_0_2%FA_15MIN2_5mlmin)
wt-005-82-06-23-22-2 Sm (Mn, 2x3)
Diode Array
210
Range: 5.441e-1
Area, Height
Time Height Area Area%
7.10 522929 130244.51 55.25
8.24 354066 101920.69 43.23
9.20 6303 2333.49 0.99
11.52 3168 1256.90 0.53
7.10
130244.5
522929
8.24
101920.7
354066
9.20
2333.5
6303
11.52
1256.9
3168
AU
Time
22a

22b

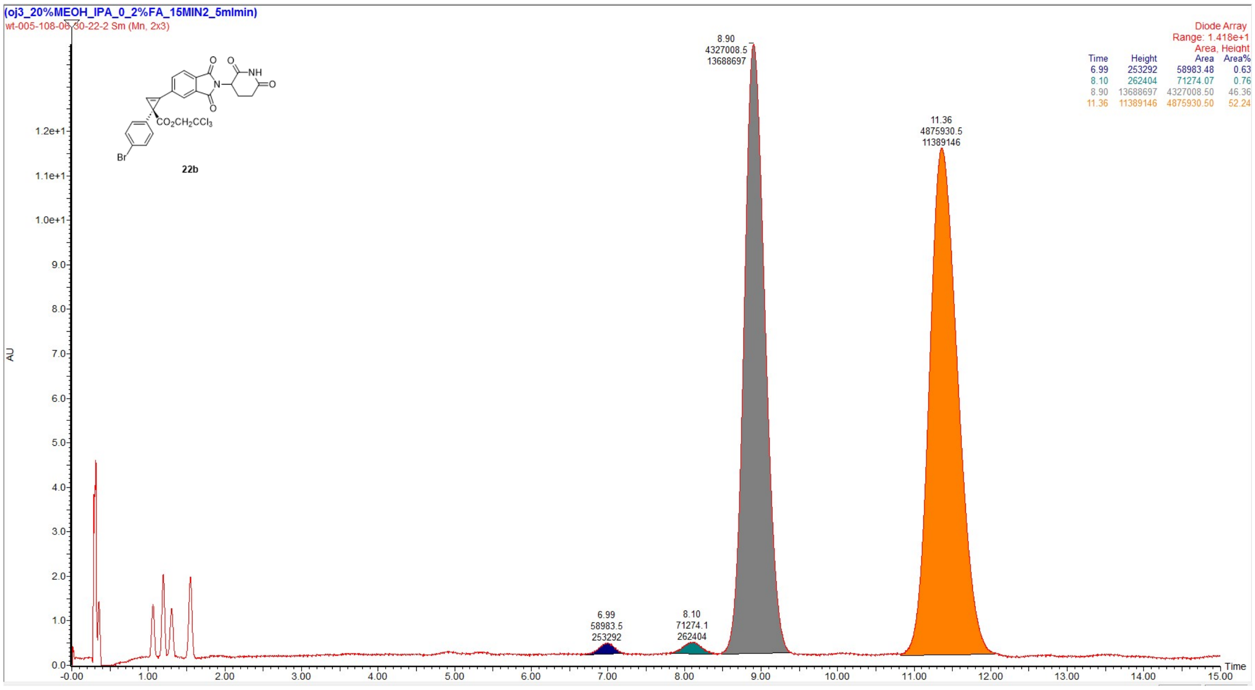
(oj3_20%MEOH_IPA_0_2%FA_15MIN2_5mlmin)
wt-005-108-06-30-22-2 Sm (Mn, 2x3)
Diode Array
Range: 1.418e+1
Area, Height
Time Height Area Area%
6.99 253292 58983.48 0.63
8.10 262404 71274.07 0.76
8.90 13688697 4327008.50 46.36
11.36 11389146 4875930.50 52.24
8.90
4327008.5
13688697
11.36
4875930.5
11389146
6.99
58983.5
253292
8.10
71274.1
262404
AU
Time
22b

Racemic Chromatogram, 23

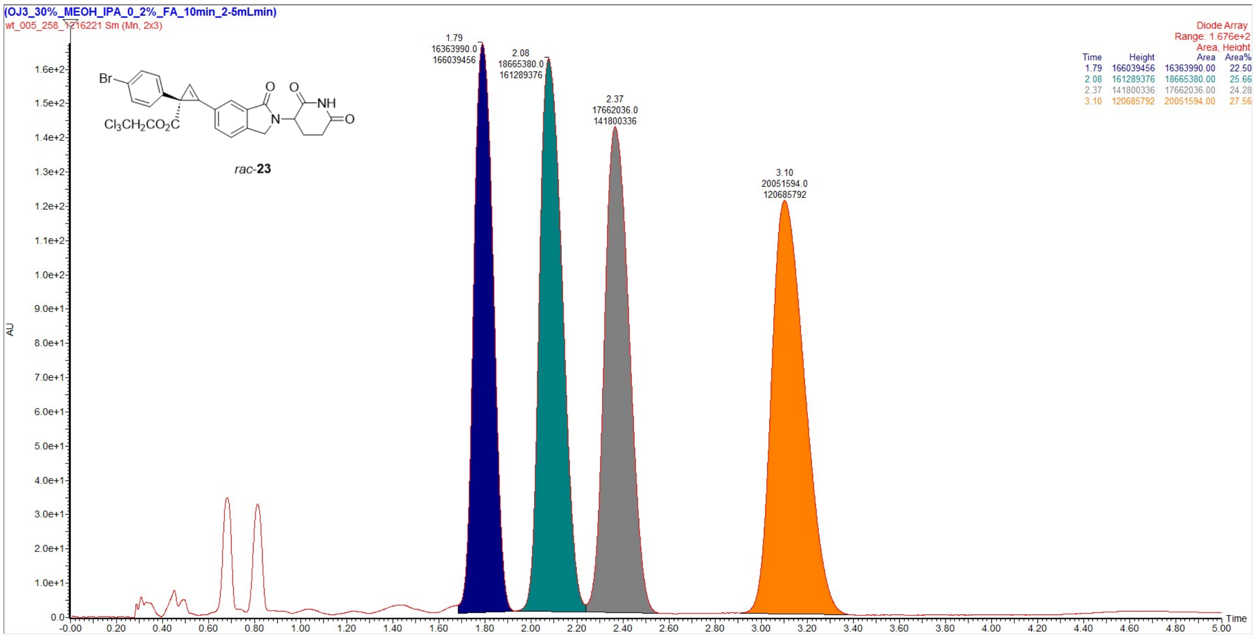

23a

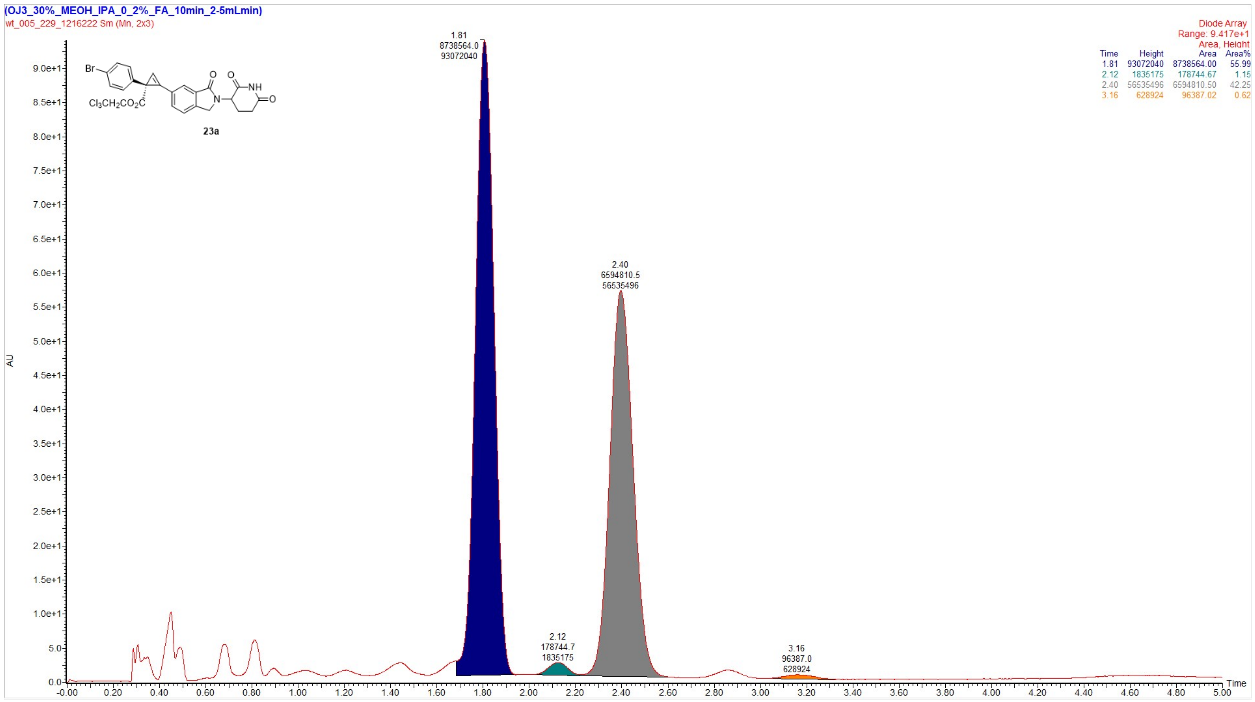

23b

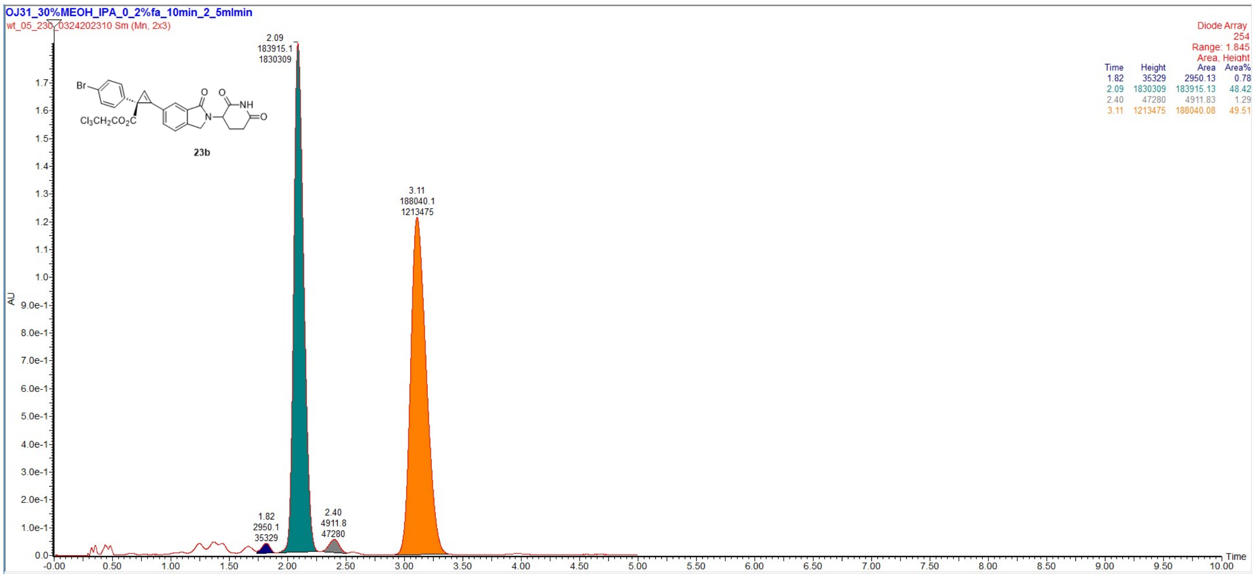

Racemic Chromatogram, 24

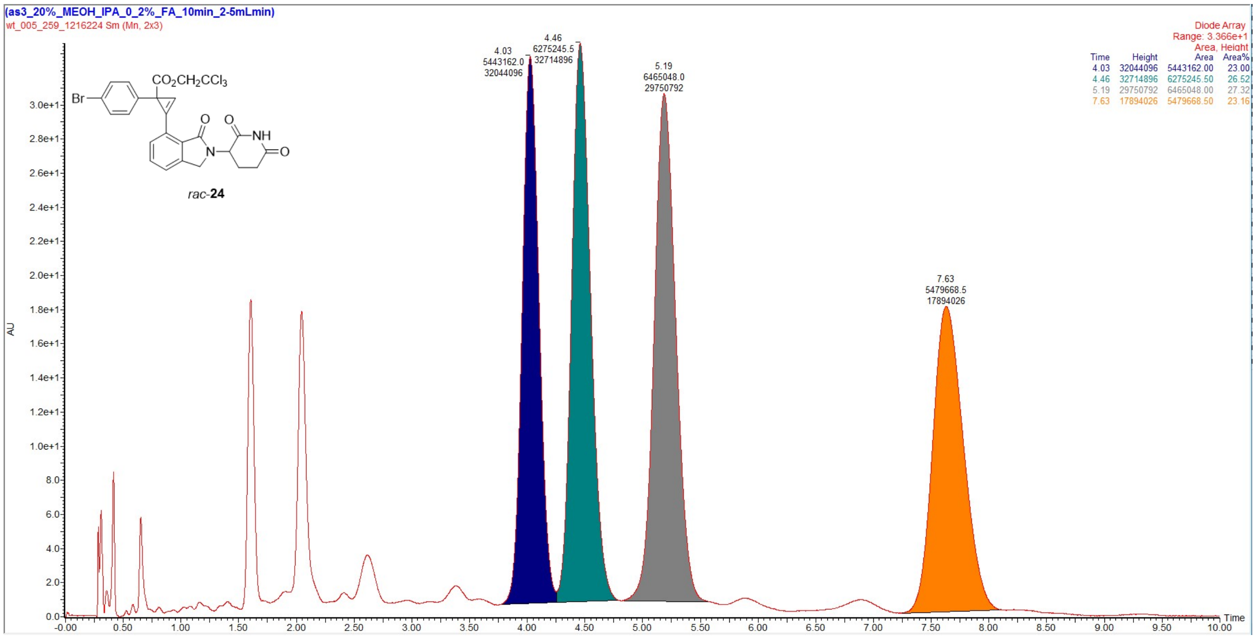

24a

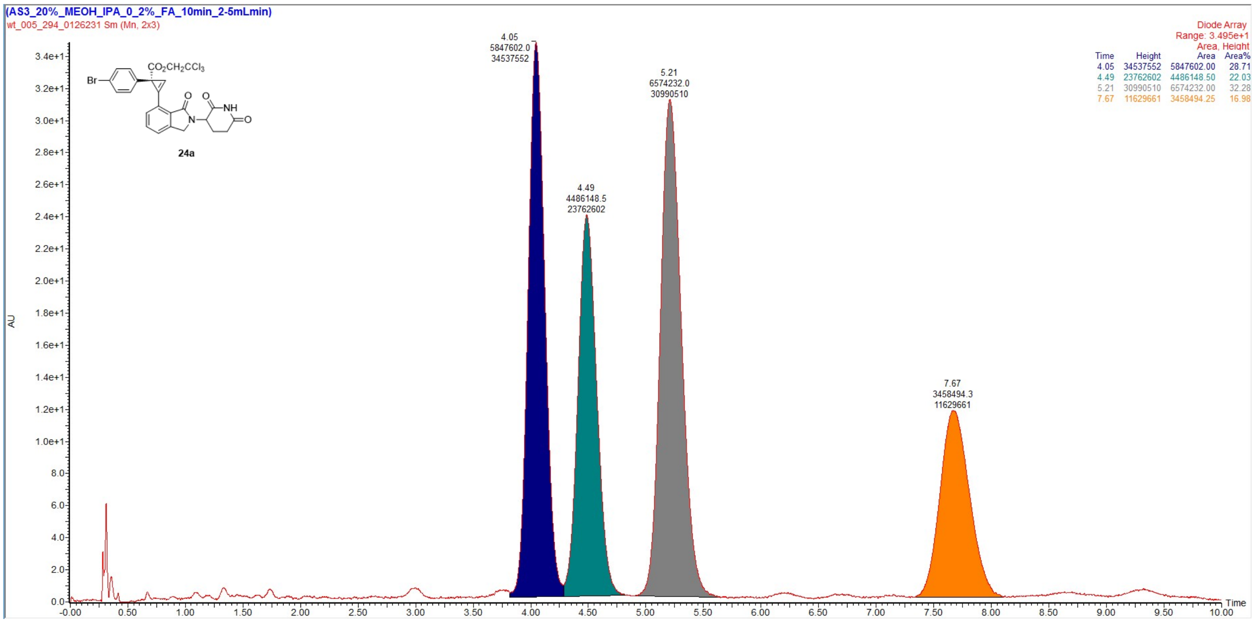
(AS3_20%_MEOH_IPA_0_2%_FA_10min_2-5mLmin)
wt_005_294_0126231 Sm (Mn, 2x3)
Diode Array
Range: 3.495e+1
Area, Height
Time Height Area Area%
4.05 34537552 5847602.00 28.71
4.49 23762602 4486148.50 22.03
5.21 30990510 6574232.00 32.28
7.67 11629661 3458494.25 16.98
4.05
5847602.0
34537552
4.49
4486148.5
23762602
5.21
6574232.0
30990510
7.67
3458494.3
11629661
24a
AU
Time

24b

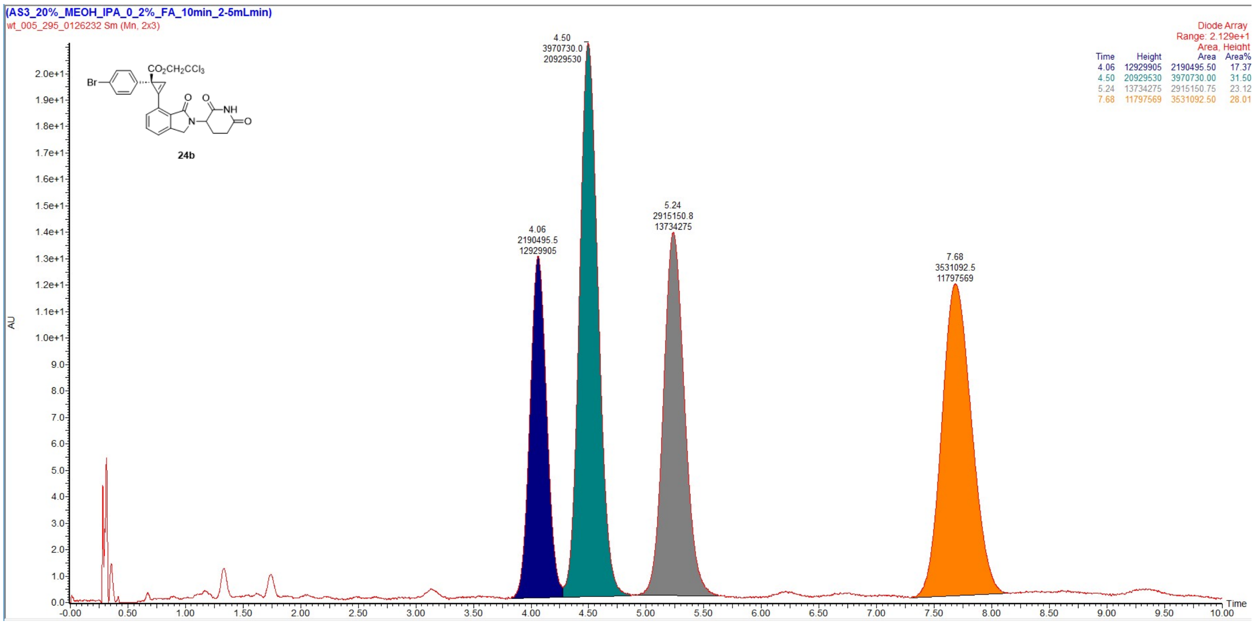
(AS3_20%_MEOH_IPA_0_2%_FA_10min_2-5mLmin)
wt_005_295_0126232 Sm (Mn, 2x3)
Diode Array
Range: 2.129e+1
Area, Height
Time Height Area Area%
4.06 12929905 2190495.50 17.37
4.50 20929530 3970730.00 31.50
5.24 13734275 2915150.75 23.12
7.68 11797569 3531092.50 28.01
4.50
3970730.0
20929530
4.06
2190495.5
12929905
5.24
2915150.8
13734275
7.68
3531092.5
11797569
24b
AU
Time

Racemic Chromatogram, 25

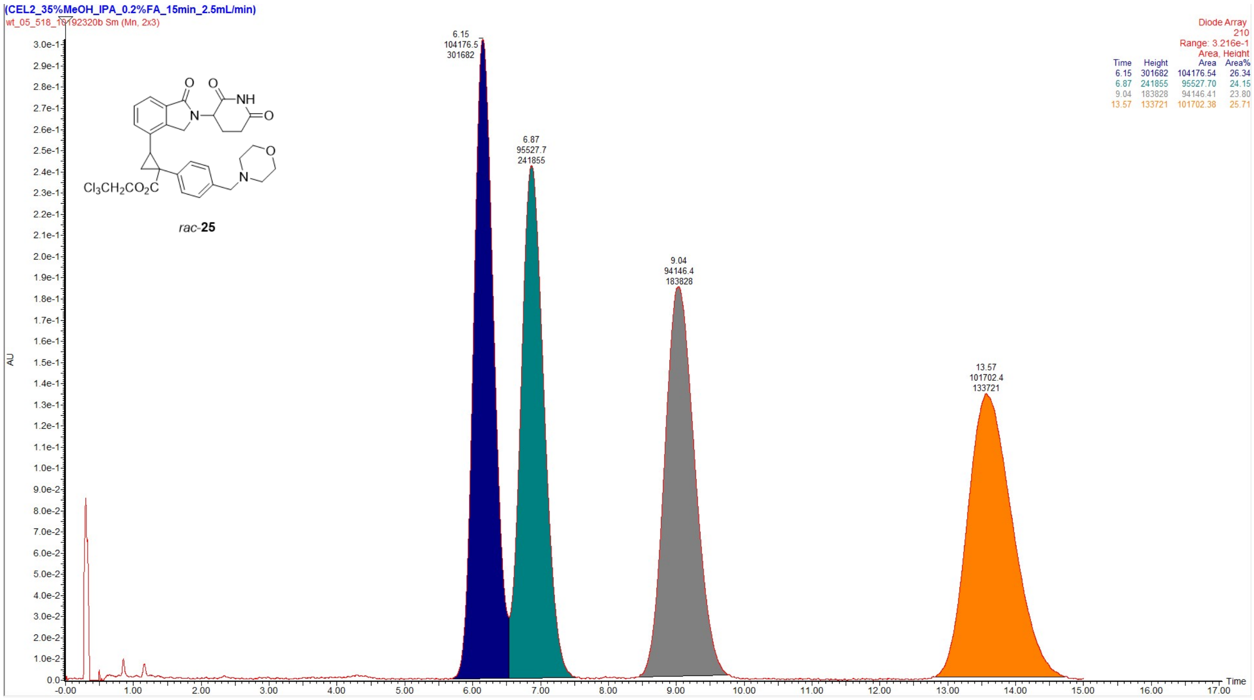

25a

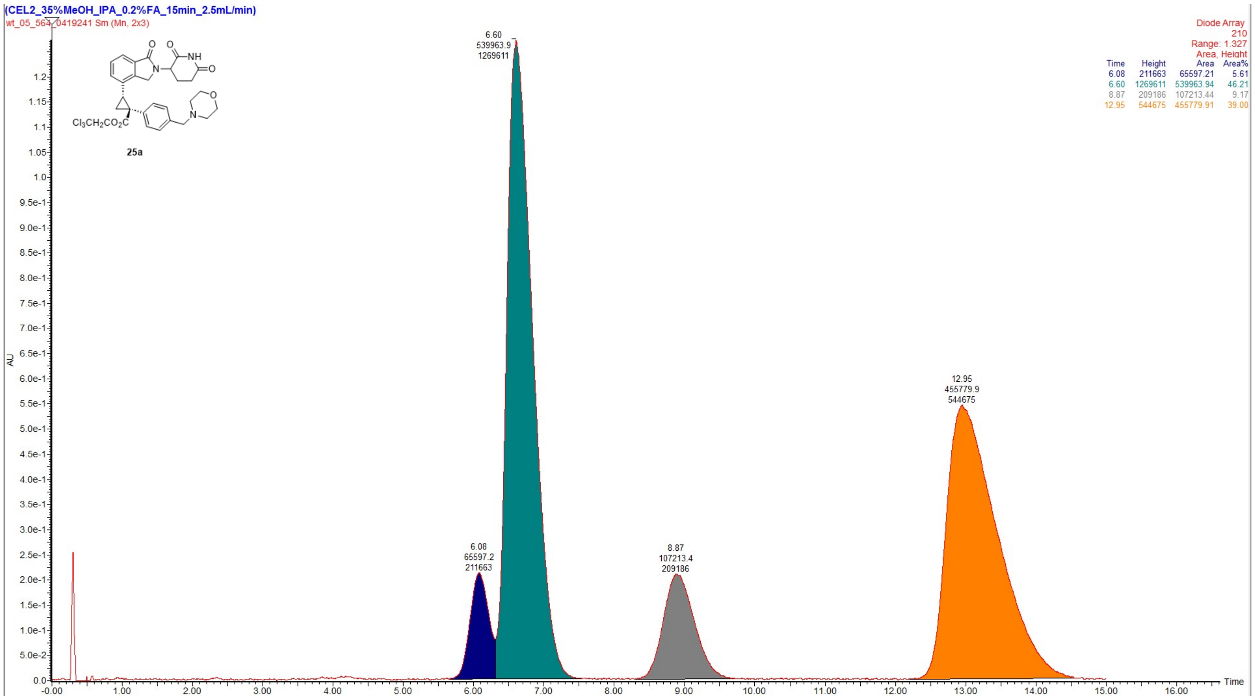

25b

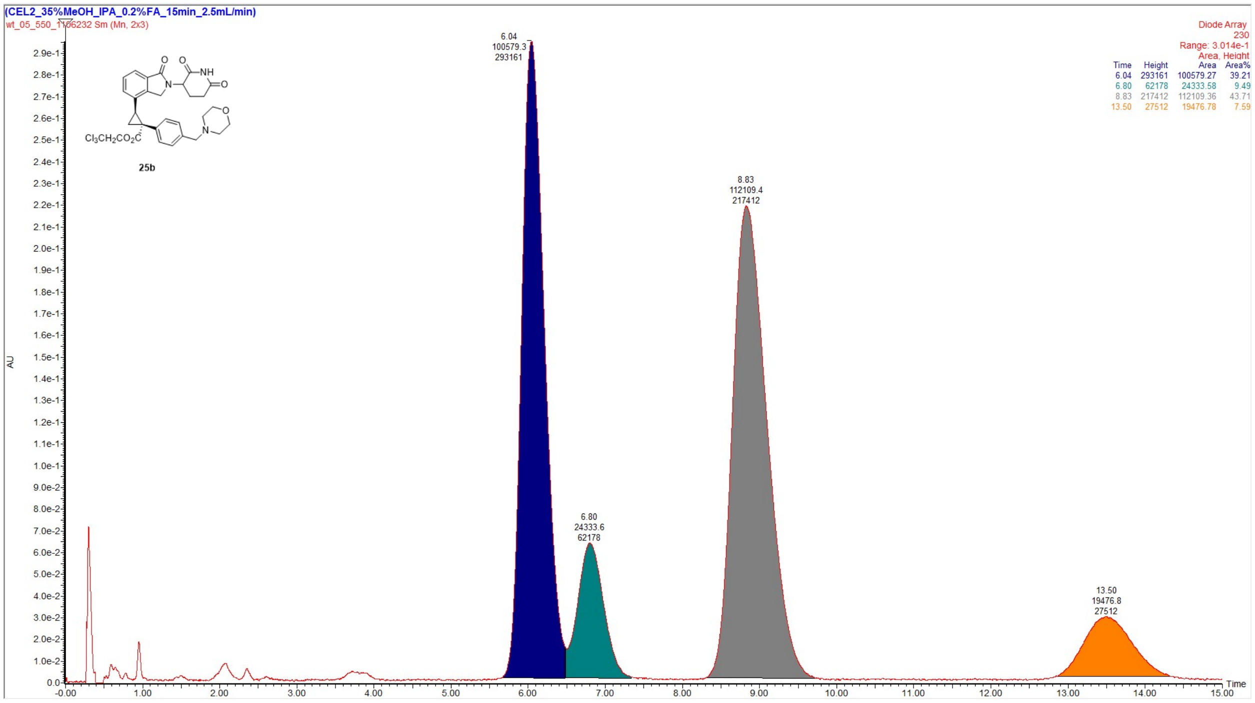

Racemic Chromatogram, 26

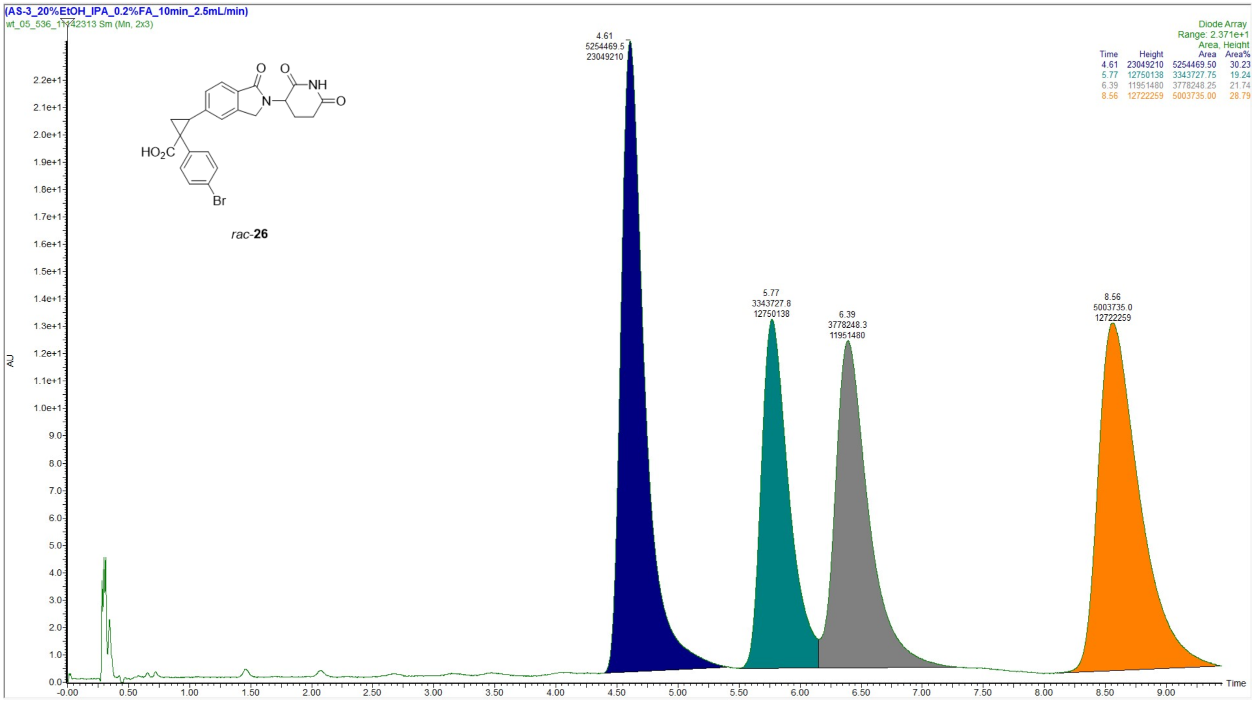

26a

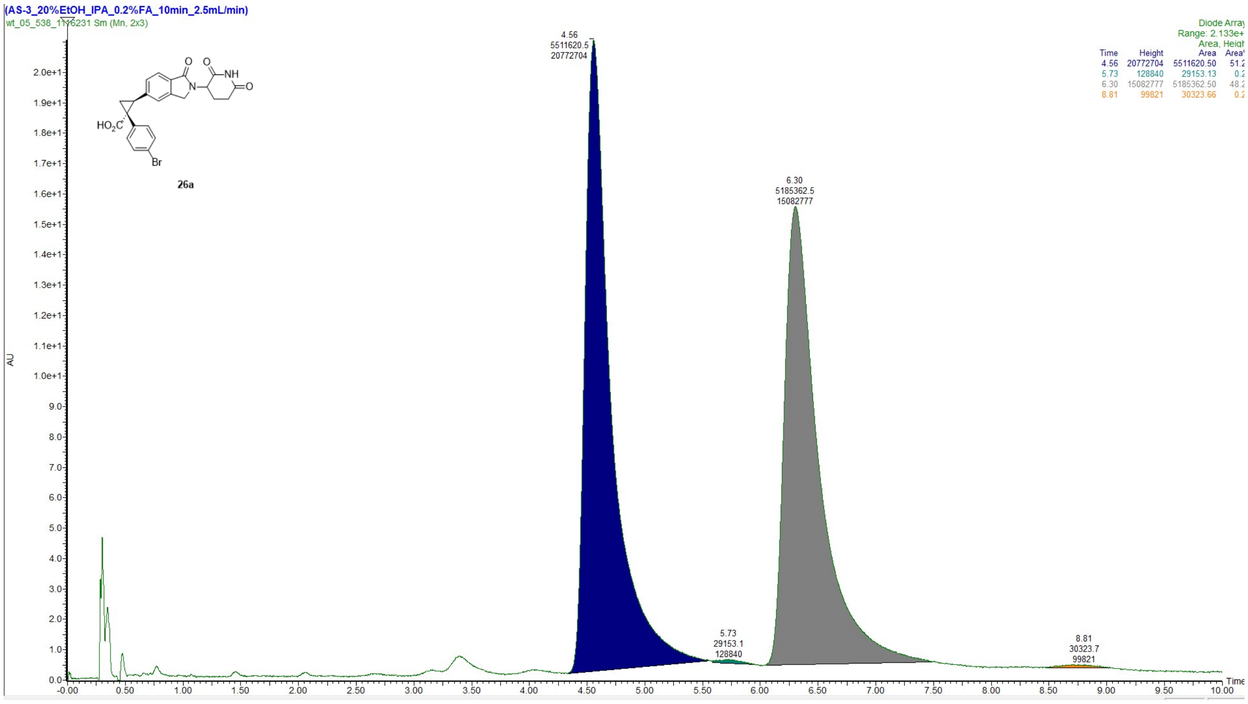

26b

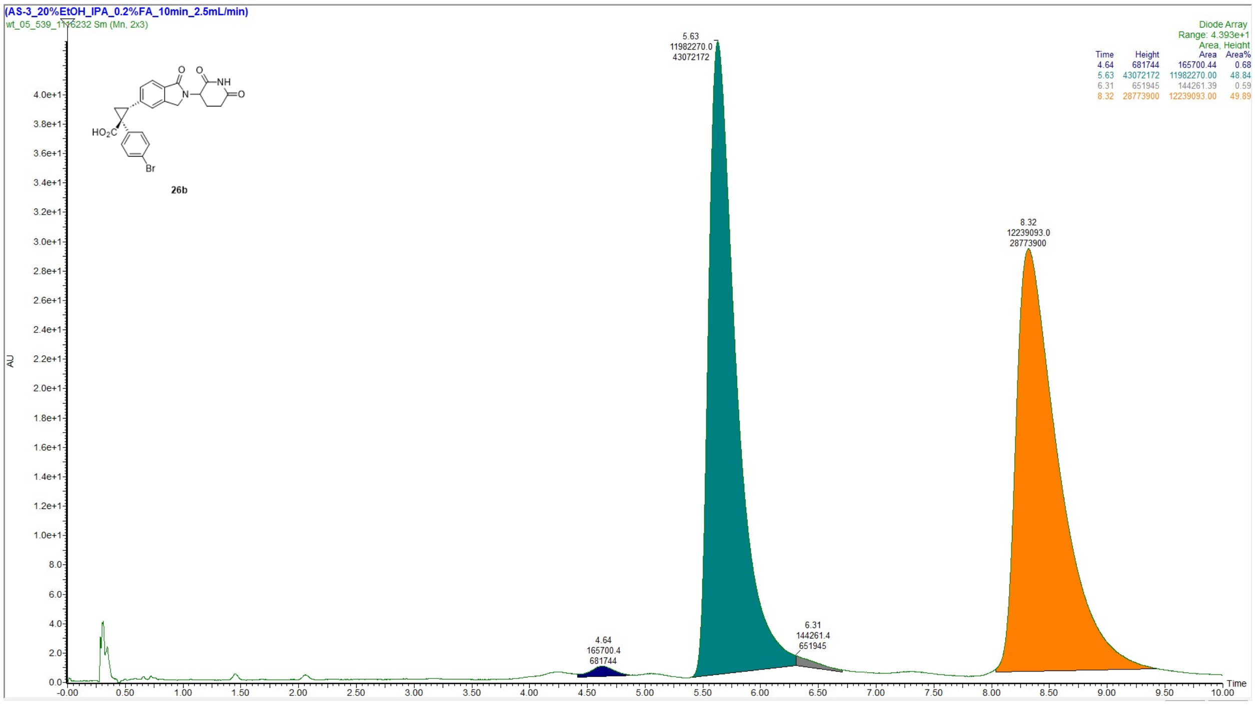

Racemic Chromatogram, 27

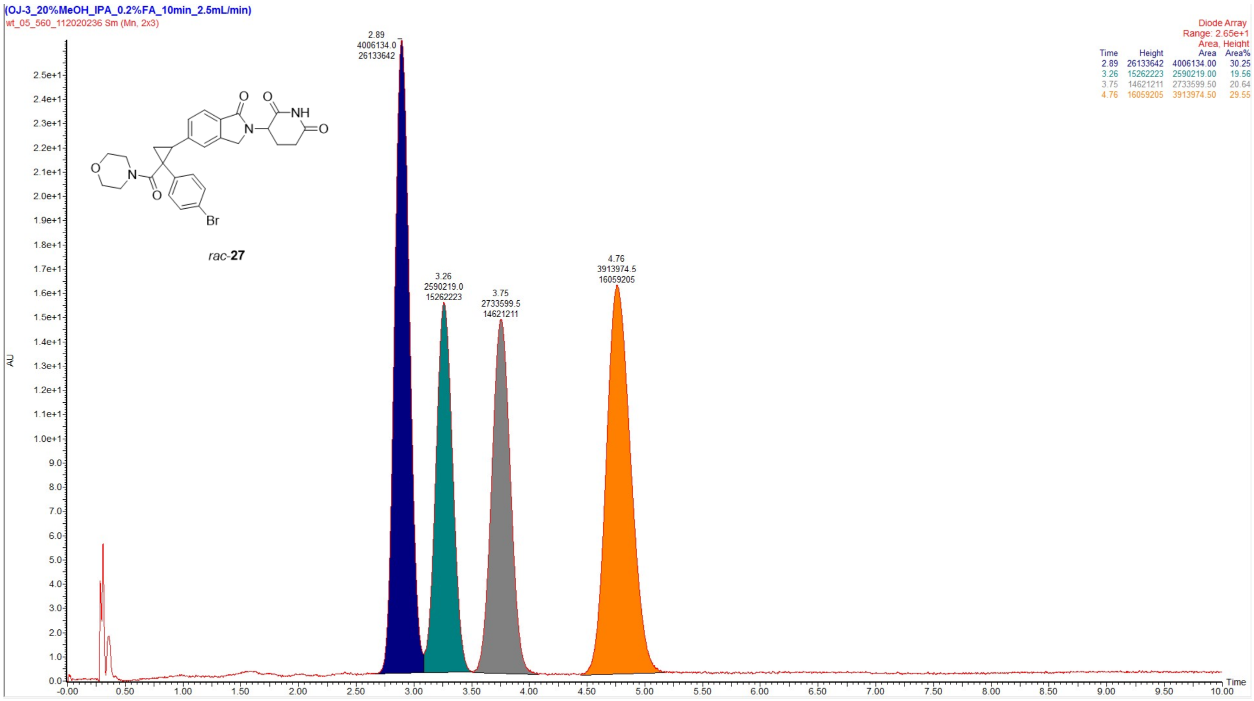

27a

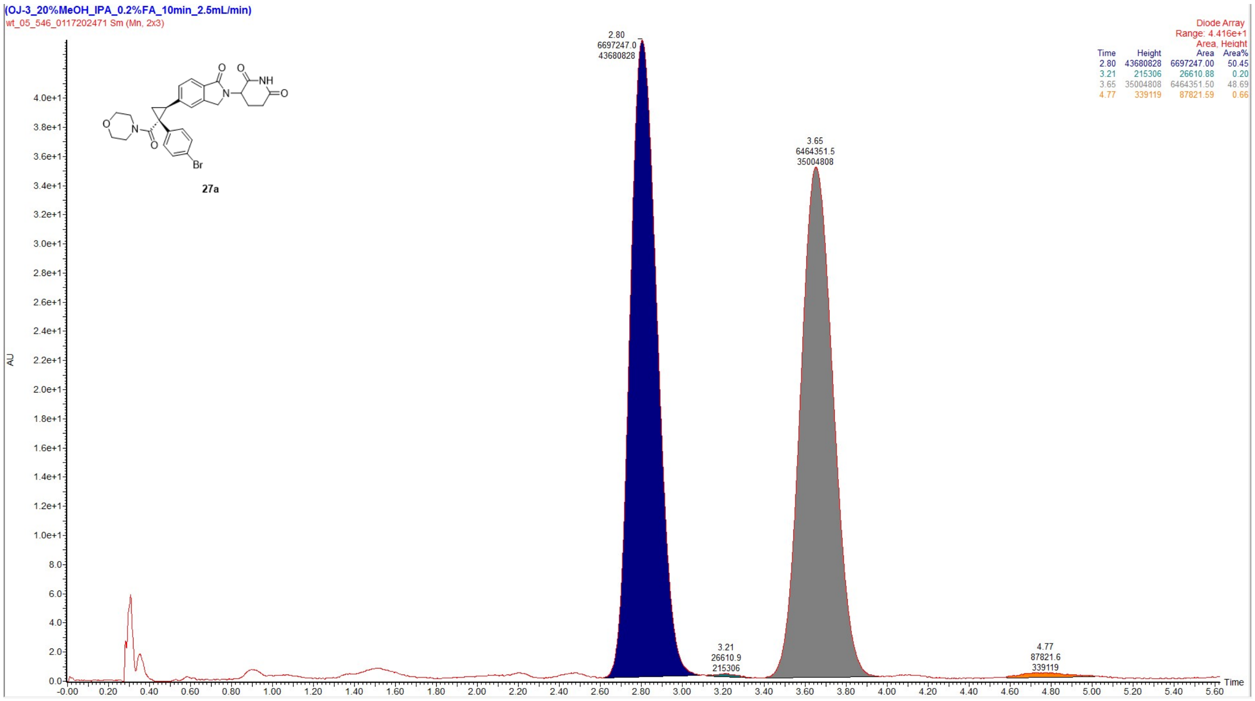

27b

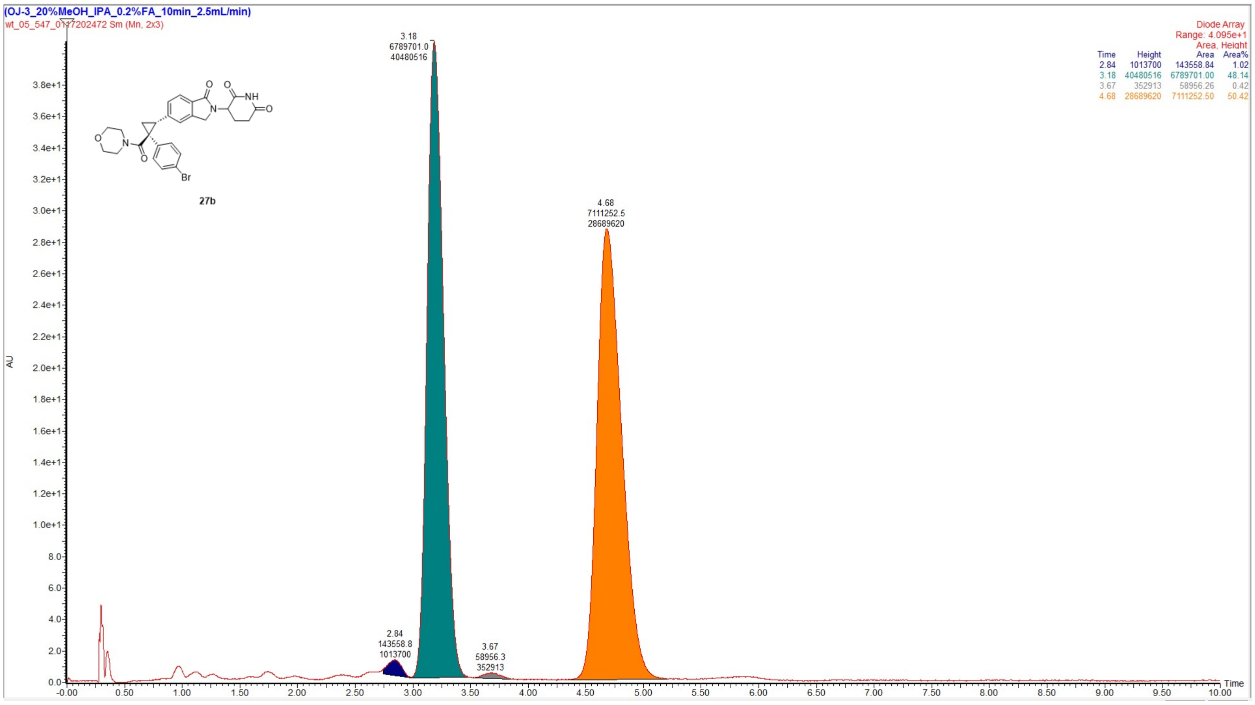

Purity Chromatograms

13a

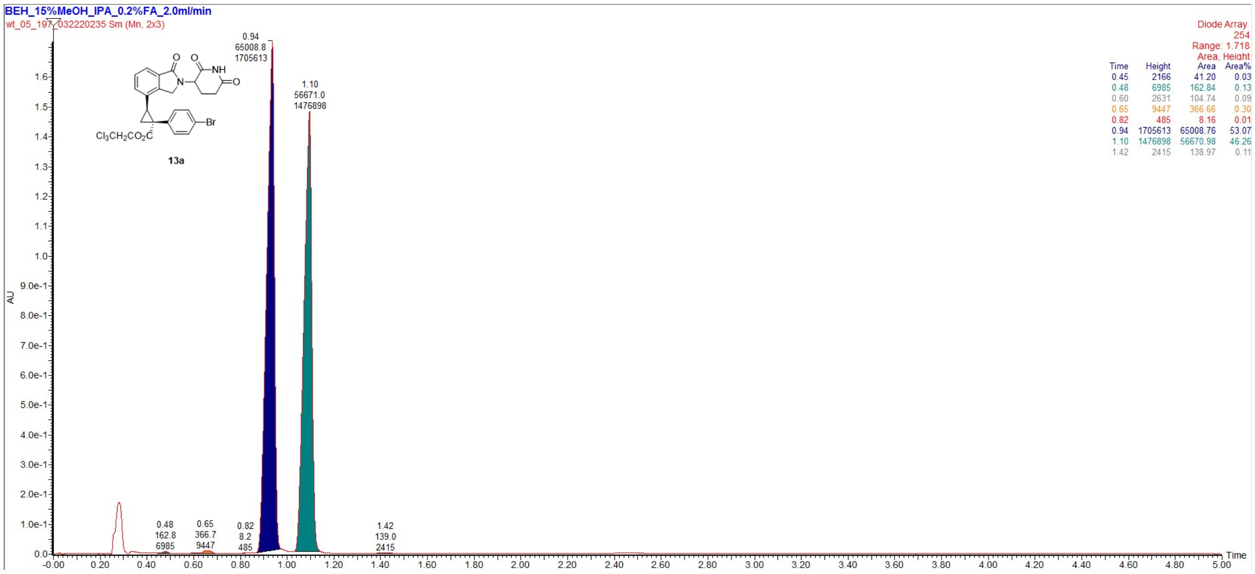

13b

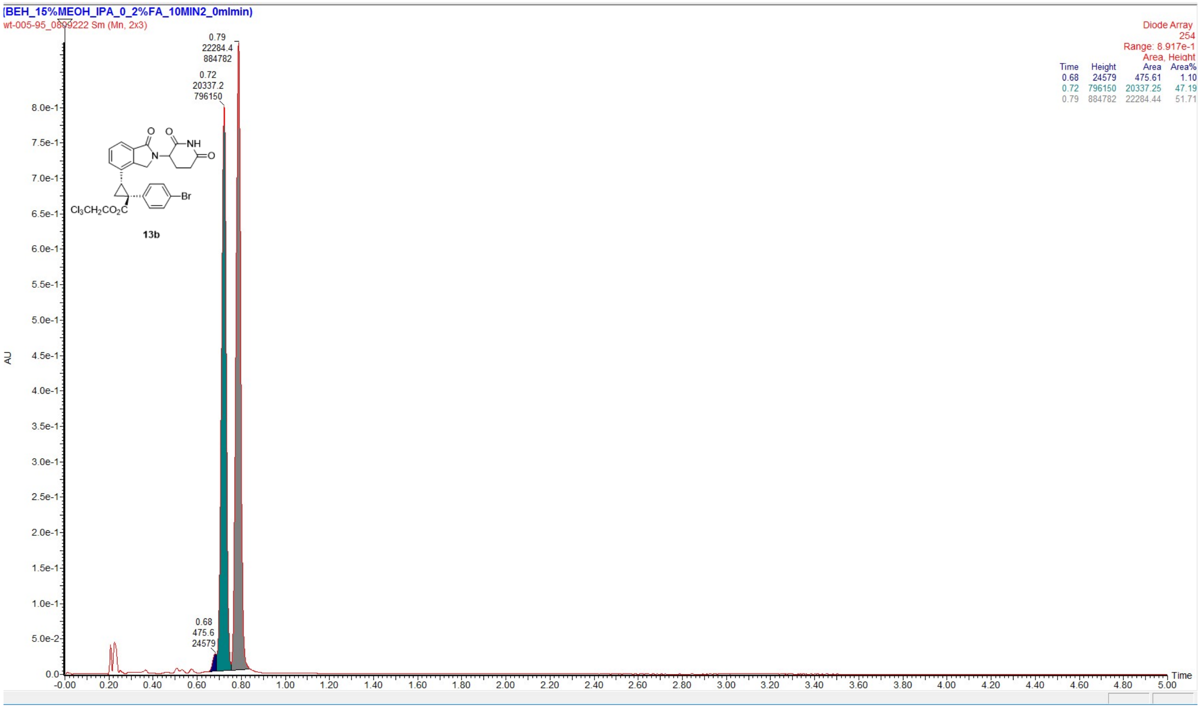

(BEH_15%MEOH_IPA_0_2%FA_10MIN2_0mlmin)
Diode Array
254
Area, Height
Time Height Area Area%
0.68 24579 475.61 1.10
0.72 796150 20337.25 47.19
0.79 884782 22284.44 51.71
13b

14a

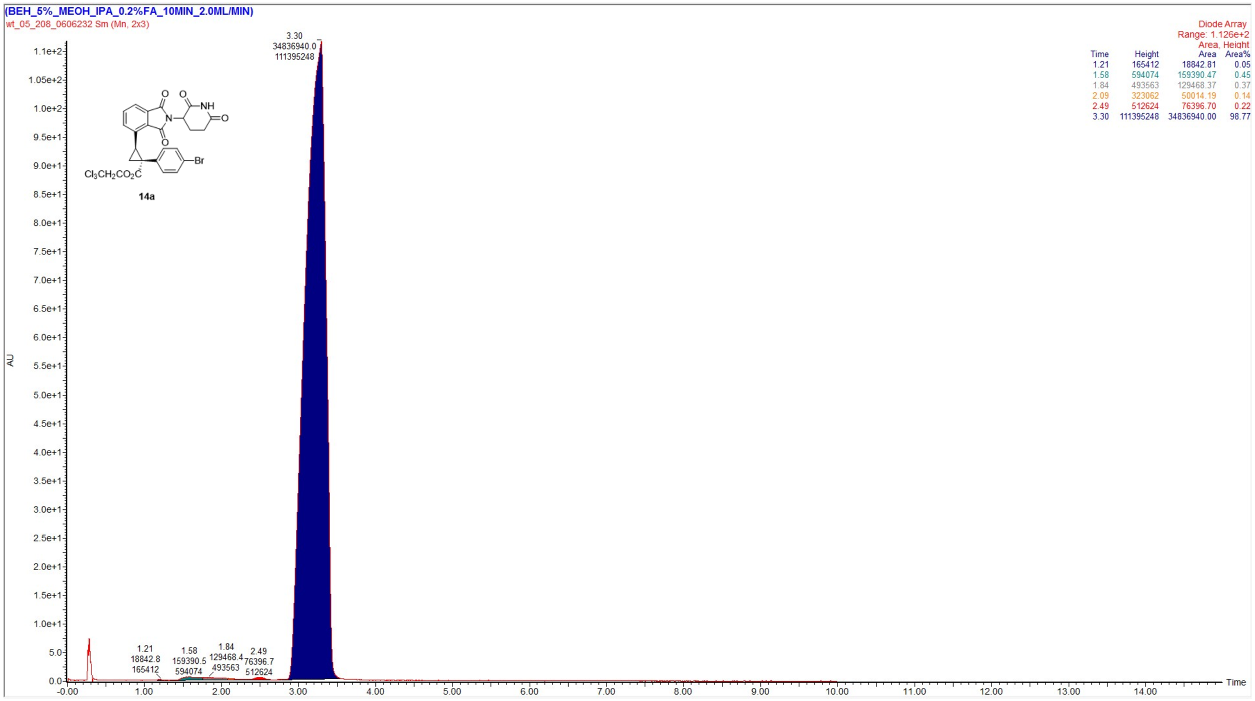

(BEH_5%_MEOH_IPA_0.2%FA_10MIN_2.0ML/MIN)
wt_05_208_0606232 Sm (Mn, 2x3)
Diode Array
Range: 1.126e+2
Area, Height
Time Height Area Area%
1.21 165412 18842.81 0.05
1.58 594074 159390.47 0.45
1.84 493563 129468.37 0.37
2.09 323062 50014.19 0.14
2.49 512624 76396.70 0.22
3.30 111395248 34836940.00 98.77
14a

14b

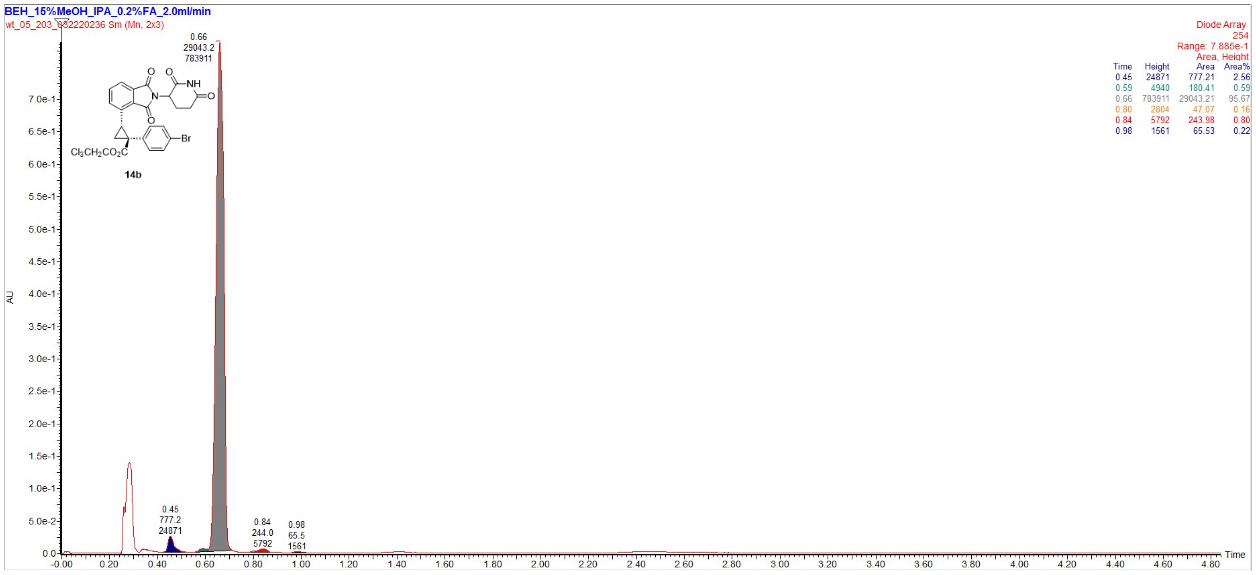
BEH_15%MeOH_IPA_0.2%FA_2.0ml/min
wt_05_203_032220236 Sm (Mn, 2x3)
Diode Array
254
Range: 7.885e-1
Area, Height
Time Height Area Area%
0.45 24871 777.21 2.56
0.59 4940 180.41 0.59
0.66 783911 29043.21 95.67
0.80 2804 47.07 0.16
0.84 5792 243.98 0.80
0.98 1561 65.53 0.22
0.66
29043.2
783911
0.45
777.2
24871
0.84
244.0
5792
0.98
65.5
1561
14b
AU
Time

15a

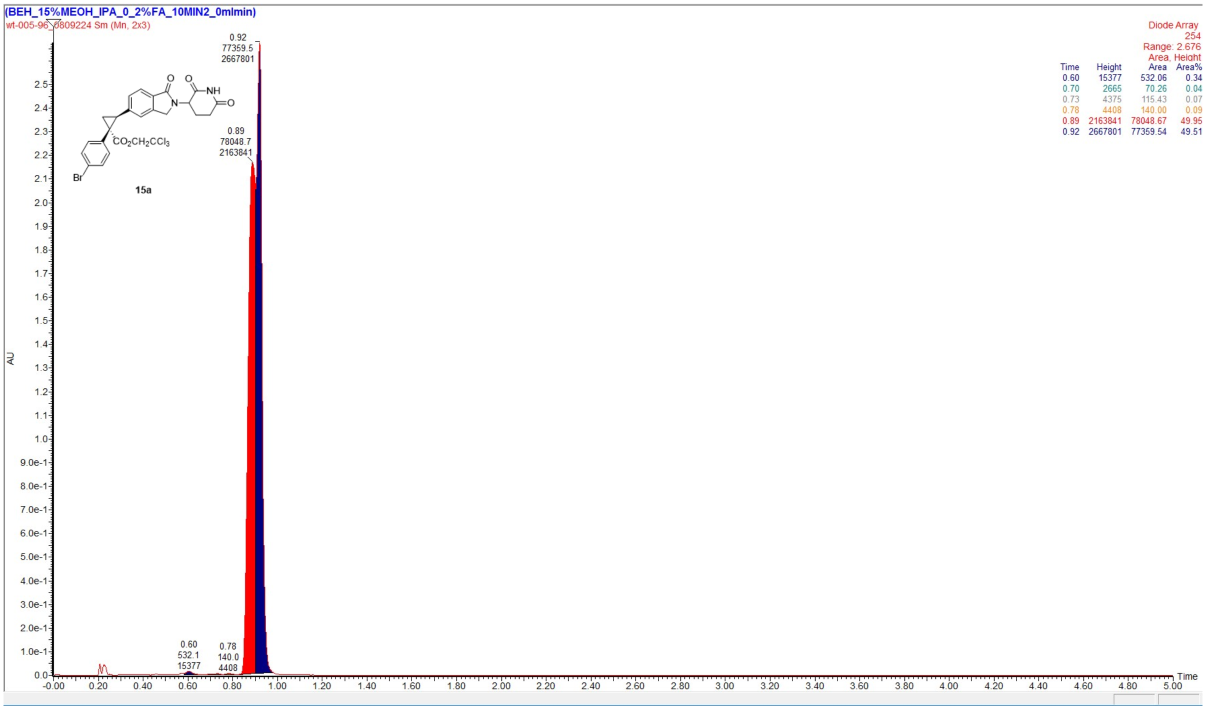
(BEH_15%MEOH_IPA_0_2%FA_10MIN2_0mlmin)
wt-005-96_0809224 Sm (Mn, 2x3)
Diode Array
254
Range: 2.676
Area, Height
Time Height Area Area%
0.60 15377 532.06 0.34
0.70 2665 70.26 0.04
0.73 4375 115.43 0.07
0.78 4408 140.00 0.09
0.89 2163841 78048.67 49.95
0.92 2667801 77359.54 49.51
0.92
77359.5
2667801
0.89
78048.7
2163841
0.60
532.1
15377
0.78
140.0
4408
15a
AU
Time

15b

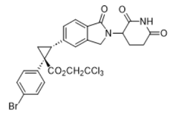

15b

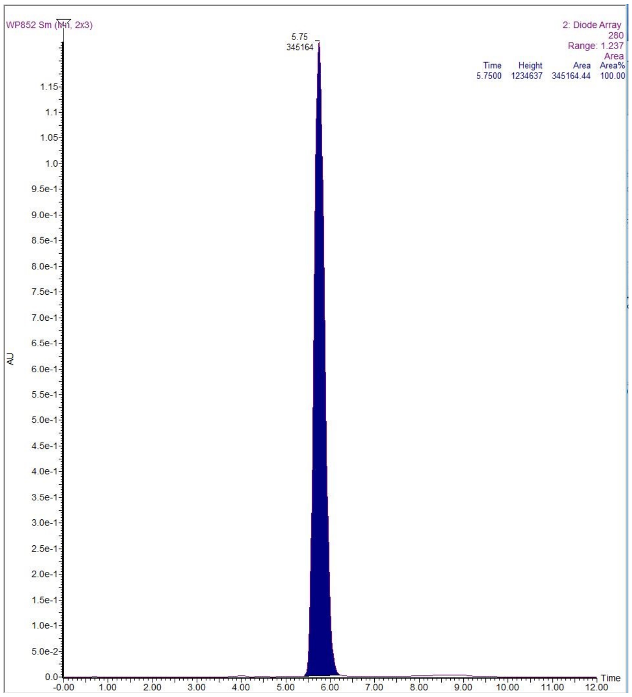

***S*-15a**

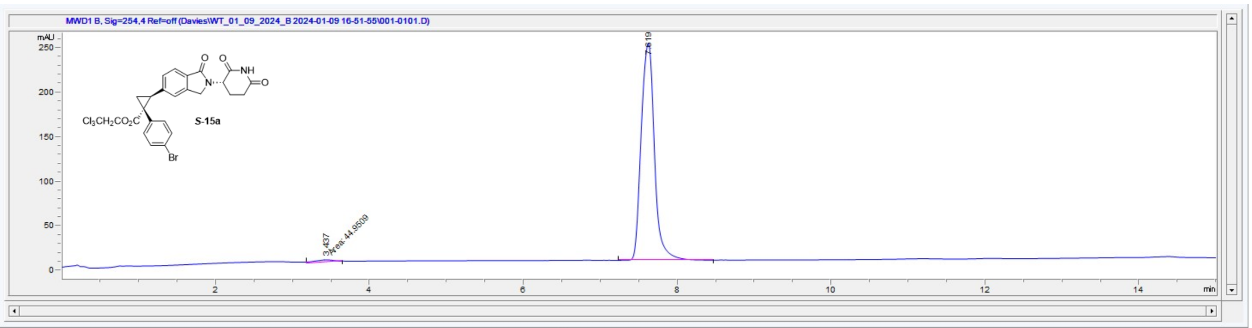

File Information

LC-File	001-0101.D
File Path	C:\Chem32\1\Data\Davies\WT_01_09_2024_B 2024-01-09 16-51-5
Date	09-Jan-24, 16:53:42
Sample	WT-05-508
Sample Info	
Barcode	
Operator	SYSTEM
Method	hdcf104_purity.M
Reference	
Analysis Time	15 min

#	Time	Area	Height	Width	Area%	Symmetry
1	3.437	45	2.5	0.2985	1.525	1.688
2	7.619	2903.3	242.8	0.1915	98.475	1.032

S-15b

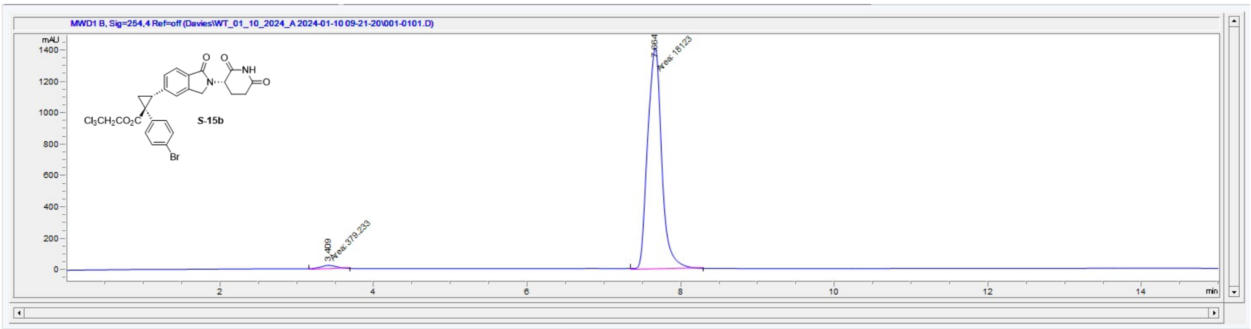

File Information

LC-File	001-0101.D
File Path	C:\Chem32\1\Data\Davies\WT_01_10_2024_A 2024-01-10 09-21-2
Date	10-Jan-24, 09:23:06
Sample	WT-05-509
Sample Info	
Barcode	
Operator	SYSTEM
Method	hdcf104_purity.M
Reference	
Analysis Time	15 min

#	Time	Area	Height	Width	Area%	Symmetry
1	3.409	379.2	23.8	0.265	2.050	0.986
2	7.664	18123	1418.4	0.213	97.950	1.084

R-15a

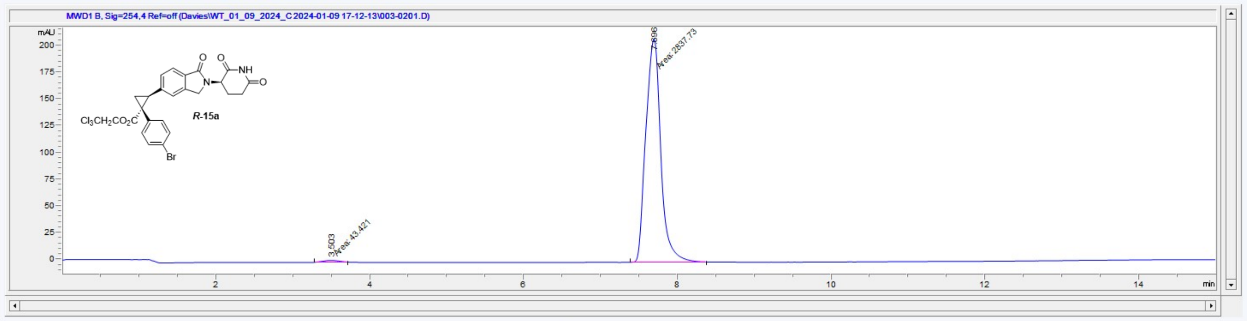

File Information

LC-File	003-0201.D
File Path	C:\Chem32\1\Data\Davies\WT_01_09_2024_C 2024-01-09 17-12-1
Date	09-Jan-24, 17:29:54
Sample	WT-05-553
Sample Info	
Barcode	
Operator	SYSTEM
Method	hdcf104_purity.M
Reference	
Analysis Time	14.993 min

#	Time	Area	Height	Width	Area%	Symmetry
1	3.503	43.4	2.4	0.3032	1.507	1.037
2	7.696	2837.7	209.1	0.2262	98.493	1.106

R-15b

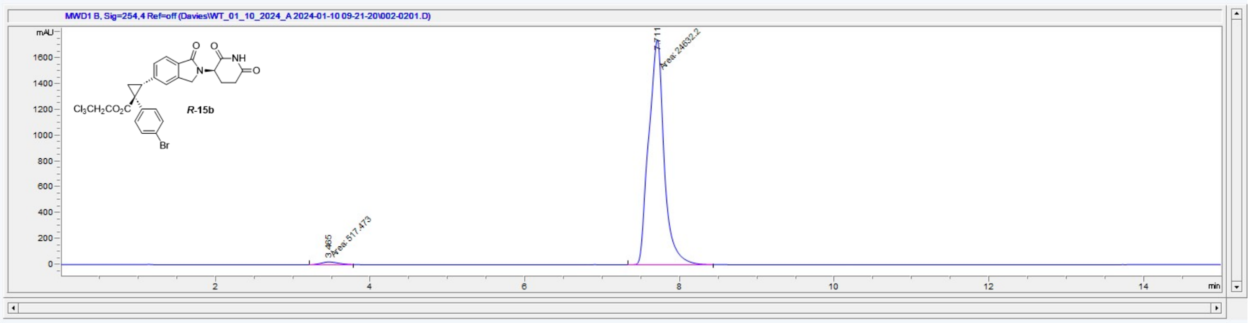

	File Information
LC-File	002-0201.D
File Path	C:\Chem32\1\Data\Davies\WT_01_10_2024_A 2024-01-10 09-21-2
Date	10-Jan-24, 09:39:06
Sample	WT-05-554
Sample Info	
Barcode	
Operator	SYSTEM
Method	hdcf104_purity.M
Reference	
Analysis Time	14.993 min

#	Time	Area	Height	Width	Area%	Symmetry
1	3.465	517.5	25.7	0.3351	2.058	0.578
2	7.711	24632.2	1755.3	0.2339	97.942	1.141

16a

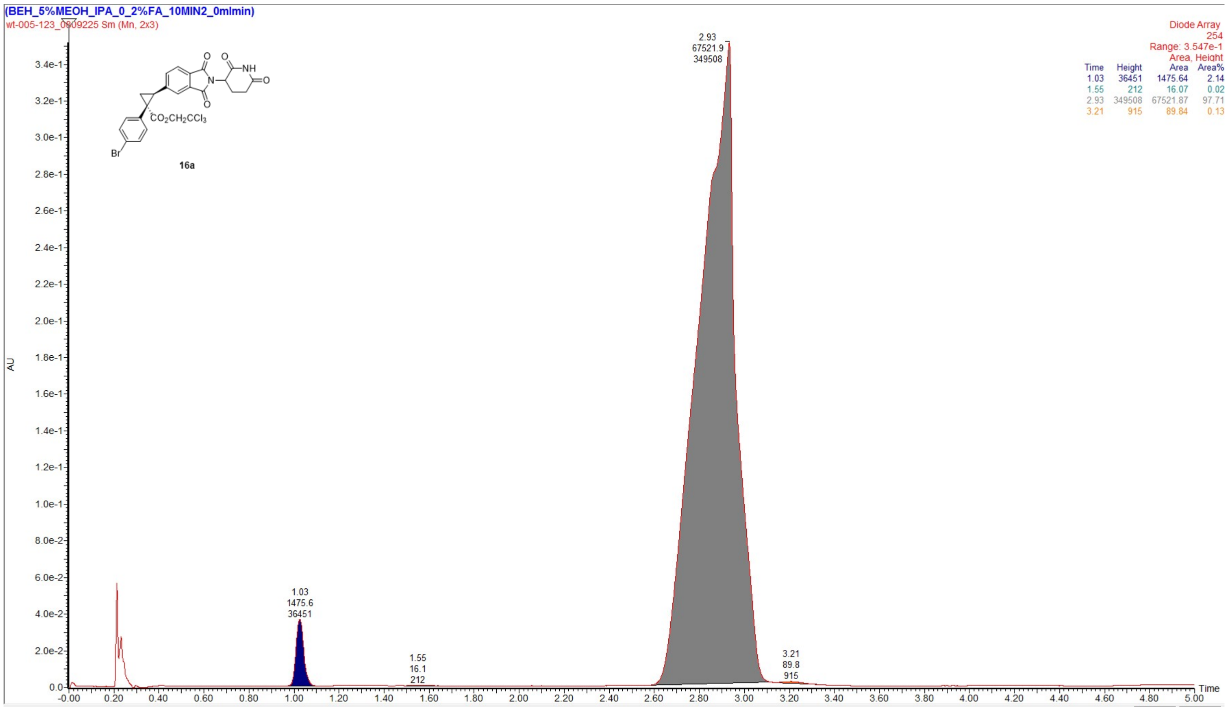

16b

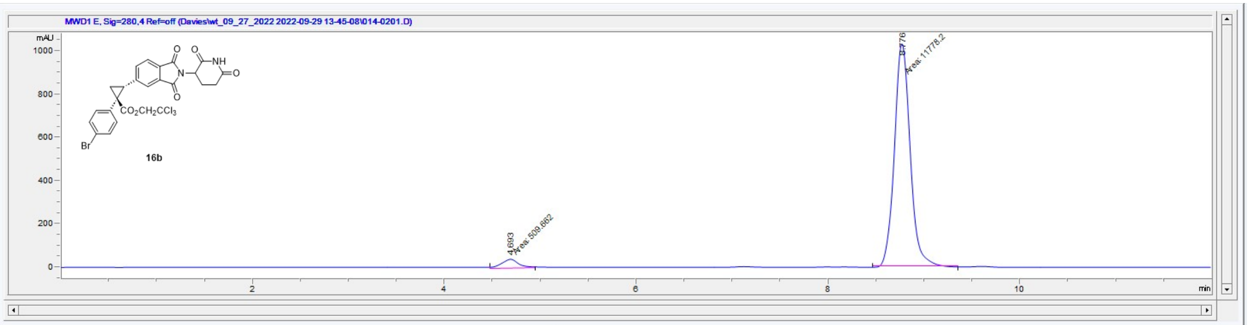

File Information	
LC-File	014-0201.D
File Path	C:\Chem32\1\Data\Davies\wt_09_27_2022 2022-09-29 13-45-08
Date	29-Sep-22, 14:00:54
Sample	wt-05-144-2
Sample Info	
Barcode	
Operator	SYSTEM
Method	30-90_30ml_12min.M
Reference	
Analysis Time	11.993 min

#	Time	Area	Height	Width	Area%	Symmetry
1	4.693	509.7	40.6	0.2092	4.148	1.096
2	8.776	11778.2	1027.3	0.1911	95.852	0.874

17a

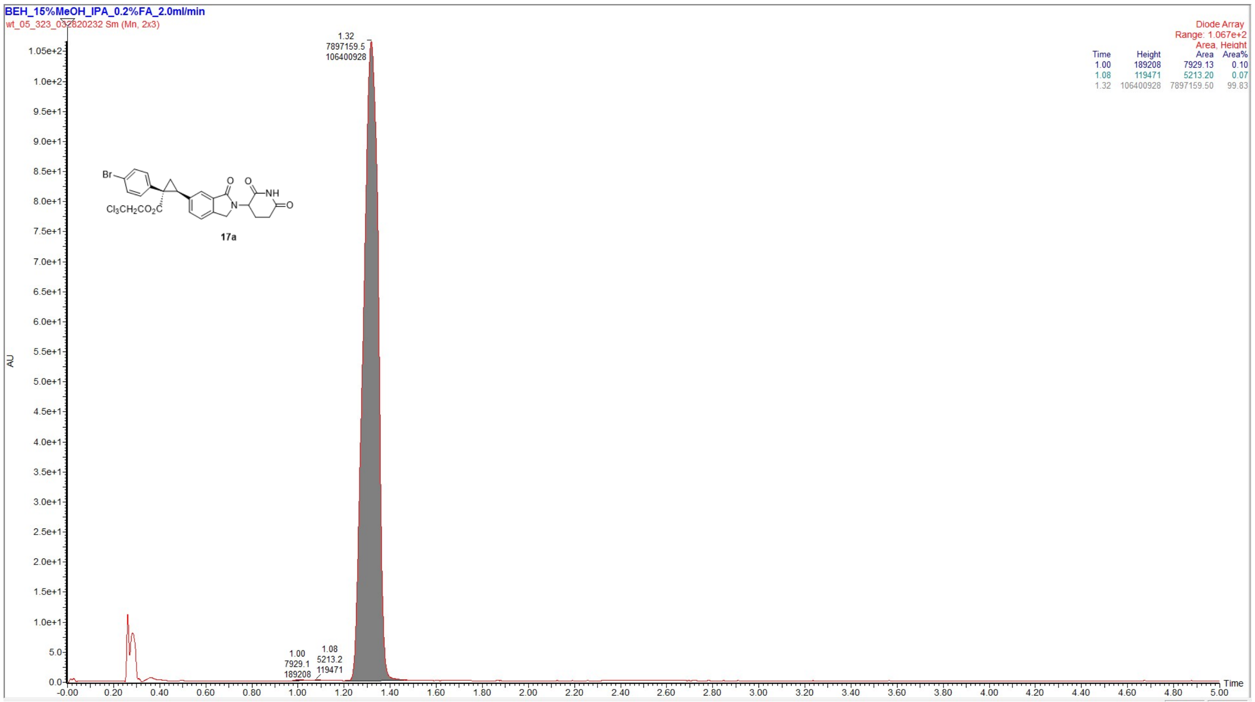

17b

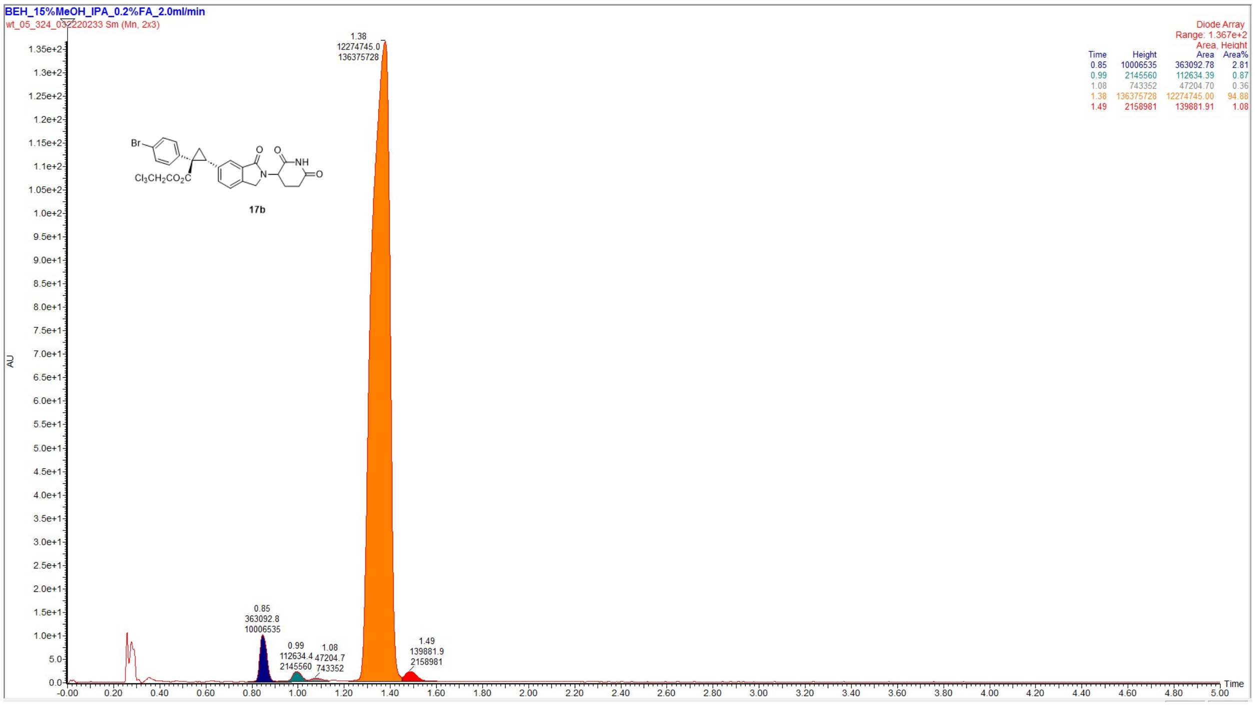

BEH_15%MeOH_IPA_0.2%FA_2.0ml/min
wt_05_324_032220233 Sm (Mn, 2x3)
Diode Array
Range: 1.367e+2
Area, Height
Time Height Area Area%
0.85 10006535 363092.78 2.81
0.99 2145560 112634.39 0.87
1.08 743352 47204.70 0.36
1.38 136375728 12274745.00 94.88
1.49 2158981 139881.91 1.08
17b
AU
Time

18a

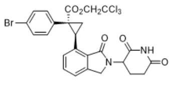

18a

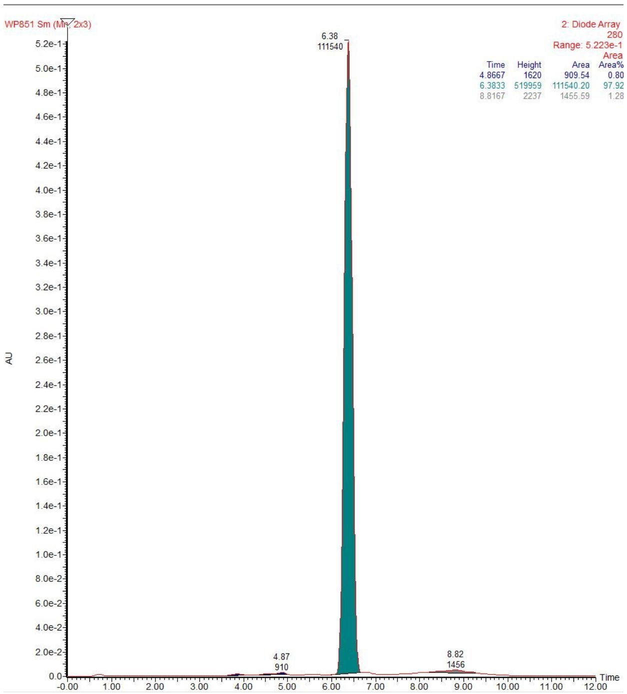

WP851 Sm (Mn, 2x3)
2: Diode Array
280
Range: 5.223e-1
Area
Time Height Area Area%
4.8667 1620 909.54 0.80
6.3833 519959 111540.20 97.92
8.8167 2237 1455.59 1.28
AU
Time

18b

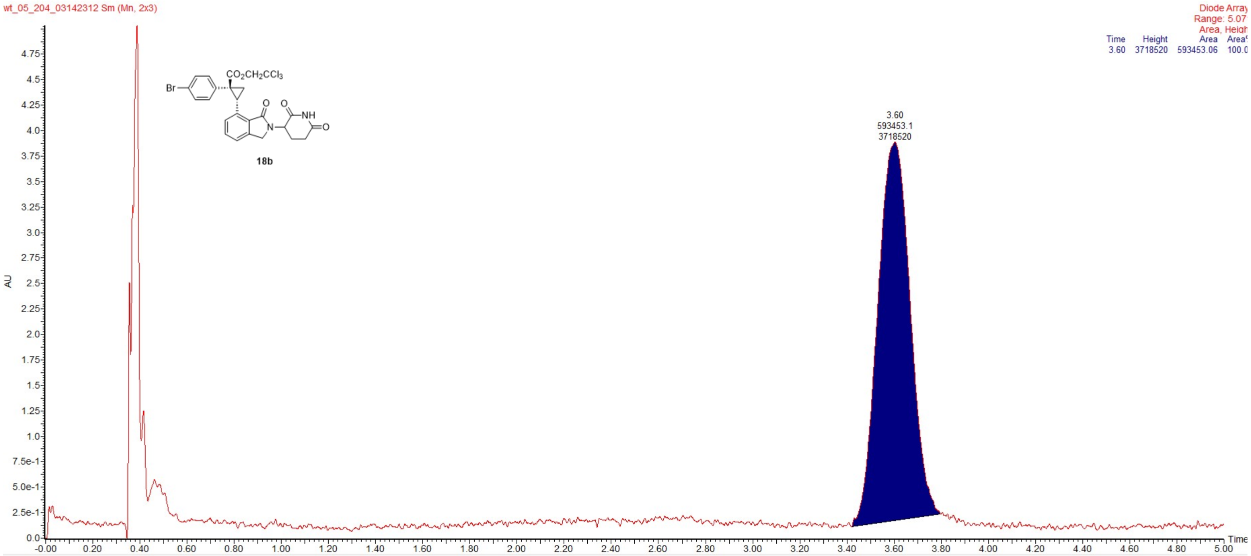
wt_05_204_03142312 Sm (Mn, 2x3)
Diode Array
Range: 5.07
Area, Height
Time Height Area Area%
3.60 3718520 593453.06 100.0
3.60
593453.1
3718520
18b
AU
Time

19a

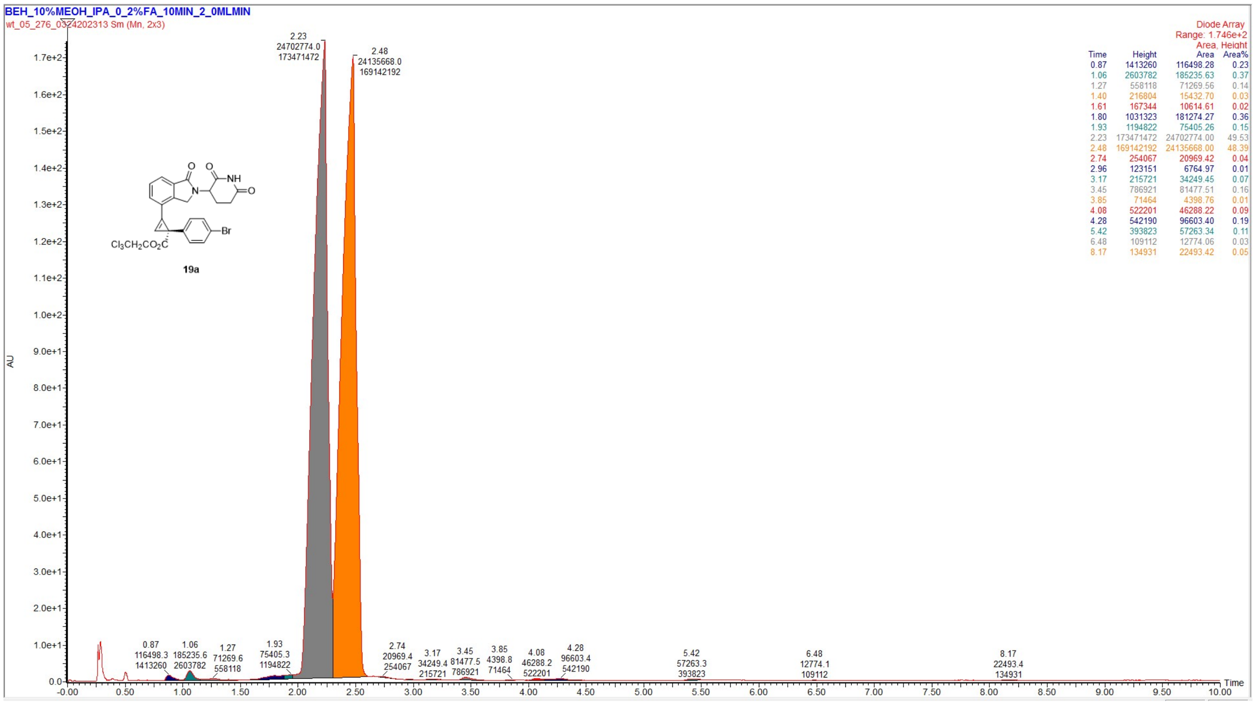
BEH_10%MEOH_IPA_0_2%FA_10MIN_2_0MLMIN
wt_05_276_03142023 Sm (Mn, 2x3)
Diode Array
Range: 1.746e+2
Area, Height
Time Height Area Area%
0.87 1413260 116498.28 0.23
1.06 2603782 185235.63 0.37
1.27 558118 71269.56 0.14
1.40 216804 15432.70 0.03
1.61 167344 10614.61 0.02
1.80 1031323 181274.27 0.36
1.93 1194822 75405.26 0.15
2.23 173471472 24702774.00 49.53
2.48 169142192 24135668.00 48.39
2.74 254067 20969.42 0.04
2.96 123151 6764.97 0.01
3.17 215721 34249.45 0.07
3.45 786921 81477.51 0.16
3.85 71464 4398.76 0.01
4.08 522201 46288.22 0.09
4.28 542190 96603.40 0.19
5.42 393823 57263.34 0.11
6.48 109112 12774.06 0.03
8.17 134931 22493.42 0.05
19a
AU
Time

19b

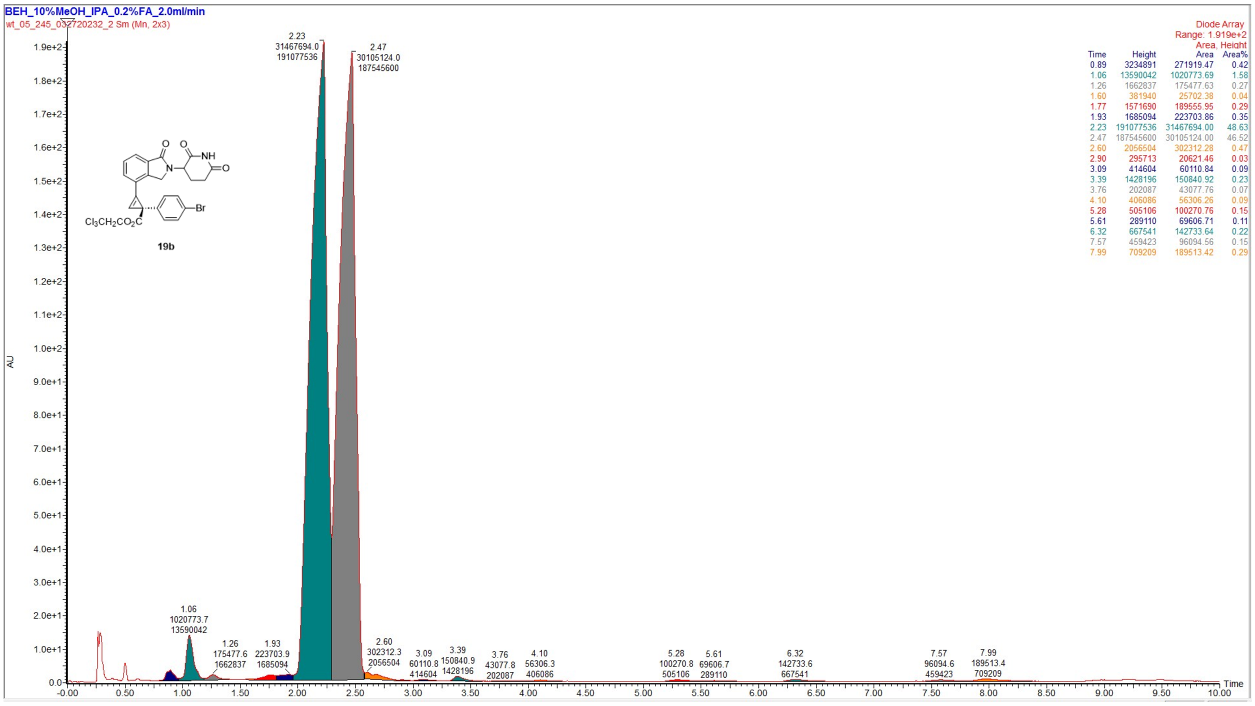
BEH_10%MeOH_IPA_0.2%FA_2.0ml/min
wt_05_245_032720232_2 Sm (Mn, 2x3)
Diode Array
Range: 1.919e+2
Area, Height
Time Height Area Area%
0.89 3234891 271919.47 0.42
1.06 13590042 1020773.69 1.58
1.26 1662837 175477.63 0.27
1.60 381940 25702.38 0.04
1.77 1571690 189555.95 0.29
1.93 1685094 223703.86 0.35
2.23 191077536 31467694.00 48.63
2.47 187545600 30105124.00 46.52
2.60 2056504 302312.28 0.47
2.90 295713 20621.46 0.03
3.09 414604 60110.84 0.09
3.39 1428196 150840.92 0.23
3.76 202087 43077.76 0.07
4.10 406086 56306.26 0.09
5.28 505106 100270.76 0.15
5.61 289110 69606.71 0.11
6.32 667541 142733.64 0.22
7.57 459423 96094.56 0.15
7.99 709209 189513.42 0.29
AU
Time

20a

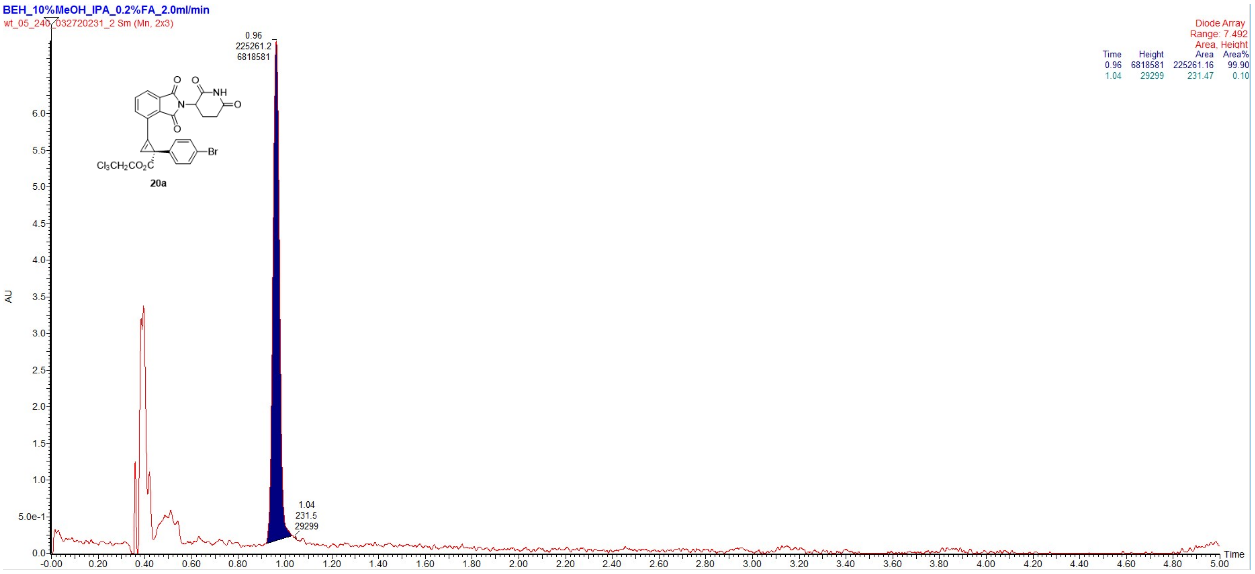
BEH_10%MeOH_IPA_0.2%FA_2.0ml/min
wt_05_240_032720231_2 Sm (Mn, 2x3)
Diode Array
Range: 7.492
Area, Height
Time Height Area Area%
0.96 6818581 225261.16 99.90
1.04 29299 231.47 0.10
AU
Time

20b

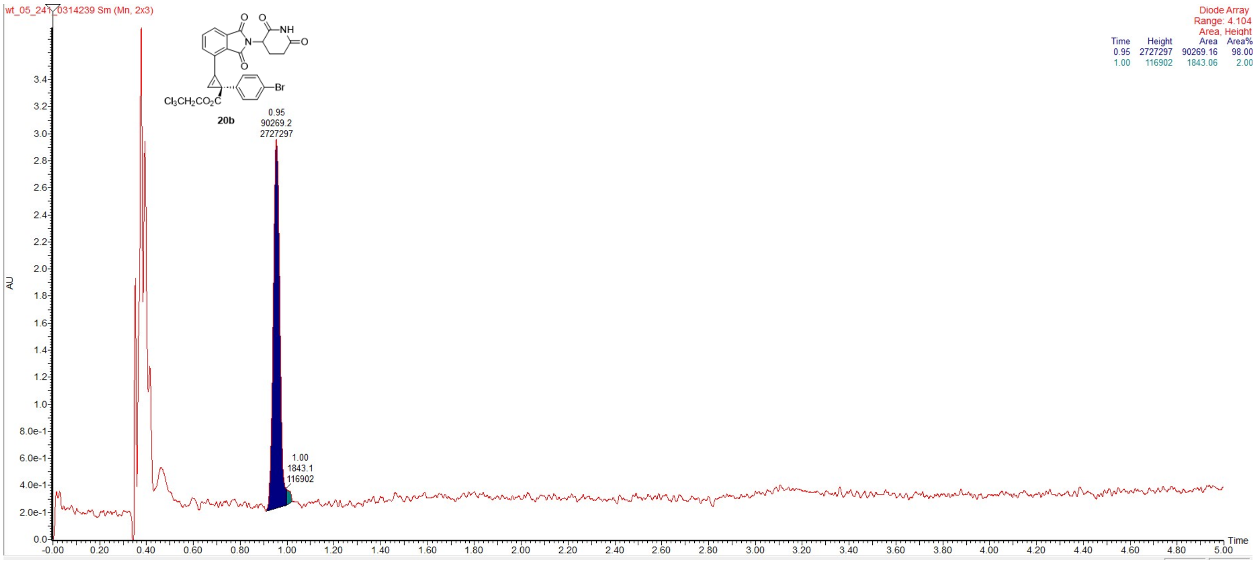

wt_05_241_0314239 Sm (Mn, 2x3)
Diode Array
Range: 4.104
Area, Height
Time Height Area Area%
0.95 2727297 90269.16 98.00
1.00 116902 1843.06 2.00
20b
0.95
90269.2
2727297
1.00
1843.1
116902
AU
Time

21a

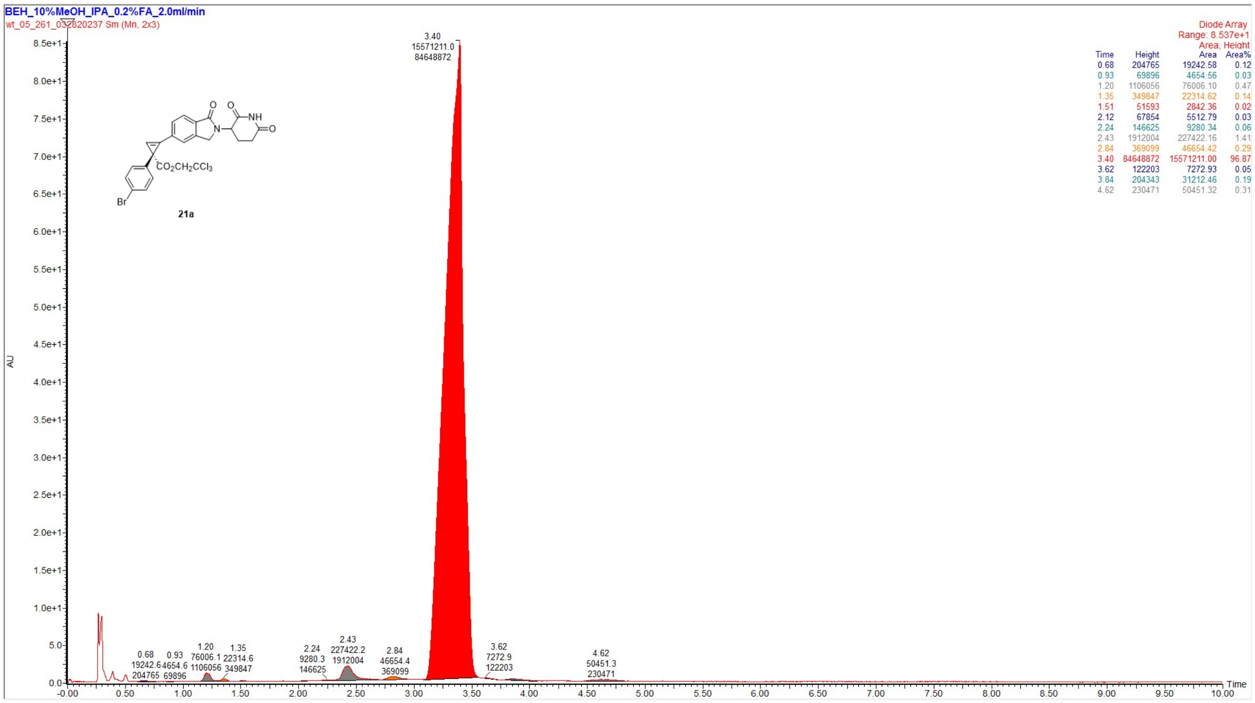

BEH_10%MeOH_IPA_0.2%FA_2.0ml/min
Diode Array
Range: 8.537e+1
Area, Height
Time Height Area Area%
0.68 204765 19242.58 0.12
0.93 69896 4654.56 0.03
1.20 1106056 76006.10 0.47
1.35 349847 22314.62 0.14
1.51 51593 2842.36 0.02
2.12 67854 5512.79 0.03
2.24 146625 9280.34 0.06
2.43 1912004 227422.16 1.41
2.84 369099 46654.42 0.29
3.40 84648872 15571211.00 96.87
3.62 122203 7272.93 0.05
3.84 204343 31212.46 0.19
4.62 230471 50451.32 0.31
21a
3.40
15571211.0
84648872
AU
Time

21b

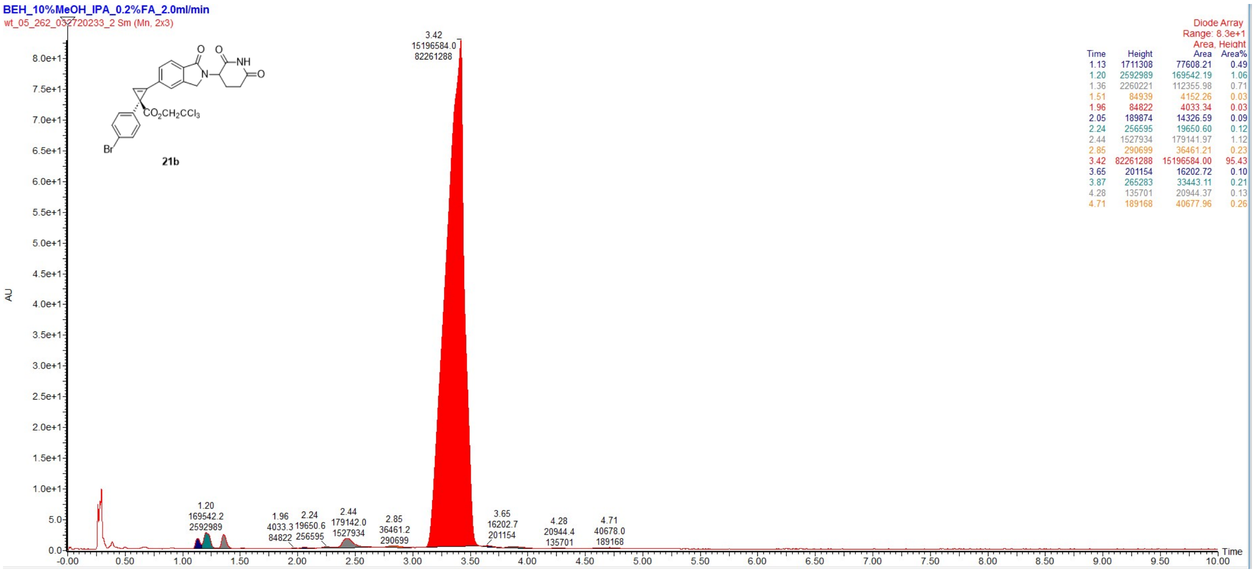
BEH_10%MeOH_IPA_0.2%FA_2.0ml/min
wt_05_262_032720233_2 Sm (Mn, 2x3)
Diode Array
Range: 8.3e+1
Area, Height
Time Height Area Area%
1.13 1711308 77608.21 0.49
1.20 2592989 169542.19 1.06
1.36 2260221 112355.98 0.71
1.51 84939 4152.26 0.03
1.96 84822 4033.34 0.03
2.05 189874 14326.59 0.09
2.24 256595 19650.60 0.12
2.44 1527934 179141.97 1.12
2.85 290699 36461.21 0.23
3.42 82261288 15196584.00 95.43
3.65 201154 16202.72 0.10
3.87 265283 33443.11 0.21
4.28 135701 20944.37 0.13
4.71 189168 40677.96 0.26
21b
$CO_2CH_2CCl_3$
AU
Time

22a

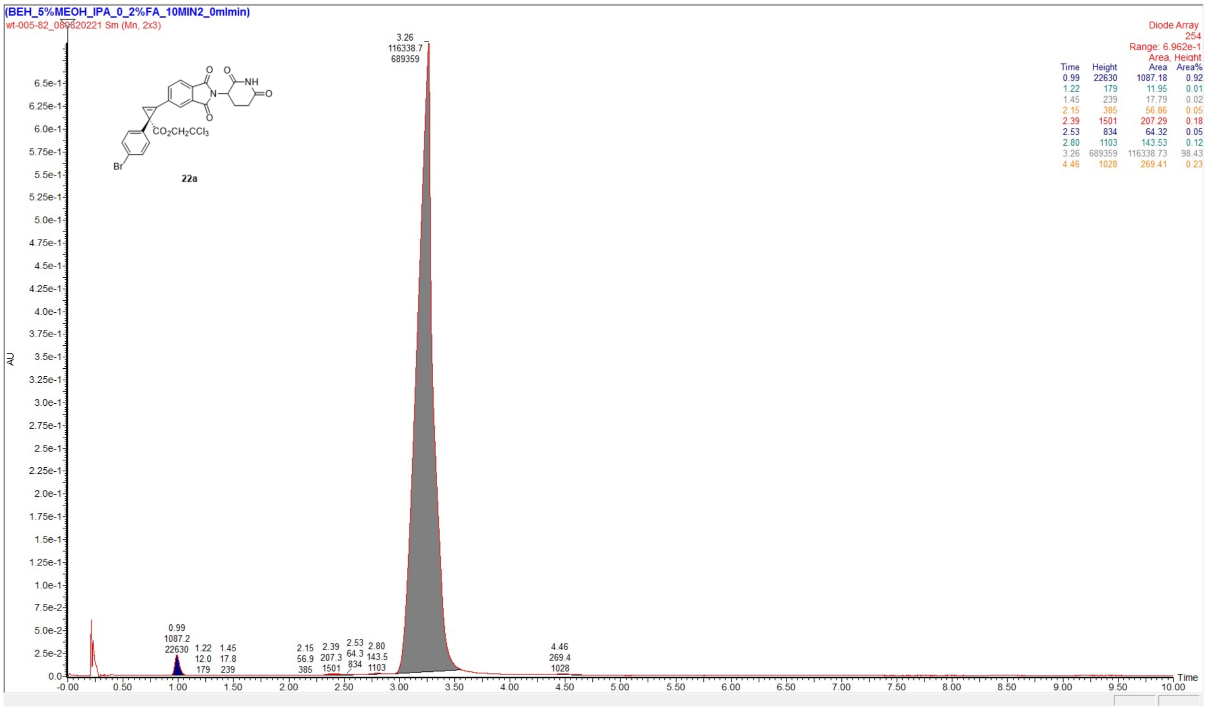
(BEH_5%MEOH_IPA_0_2%FA_10MIN2_0mlmin)
wt-005-82_080820221 Sm (Mn, 2x3)
Diode Array
254
Range: 6.962e-1
Area, Height
Time Height Area Area%
0.99 22630 1087.18 0.92
1.22 179 11.95 0.01
1.45 239 17.79 0.02
2.15 385 56.86 0.05
2.39 1501 207.29 0.18
2.53 834 64.32 0.05
2.80 1103 143.53 0.12
3.26 689359 116338.73 98.43
4.46 1028 269.41 0.23
22a
$CO_2CH_2CCl_3$
AU
Time

22b

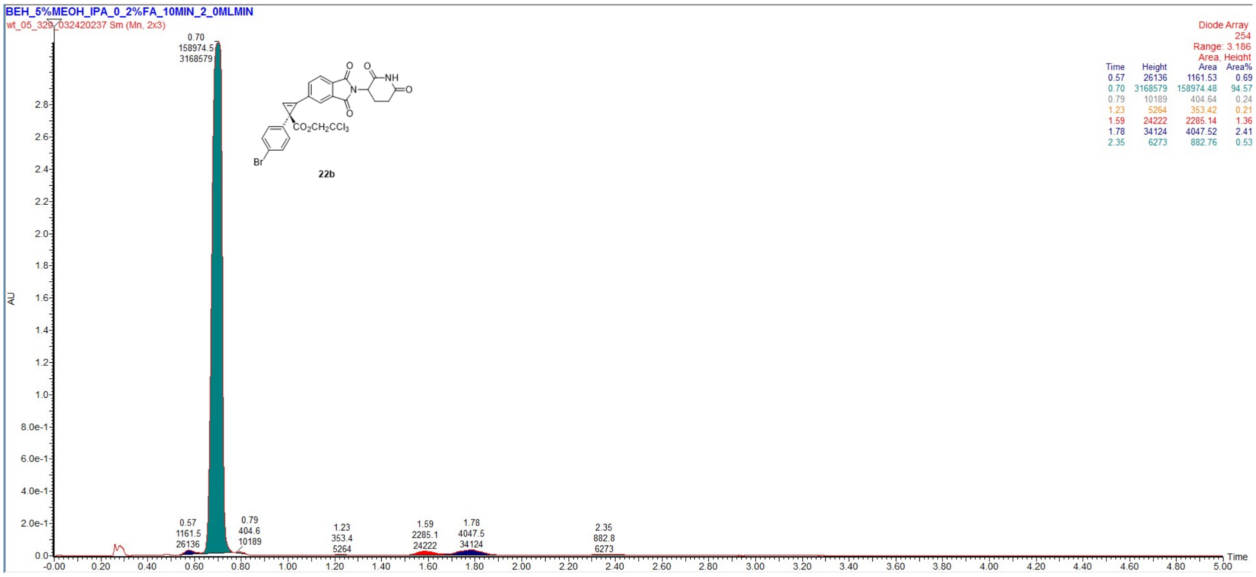
BEH_5%MEOH_IPA_0_2%FA_10MIN_2_0MLMIN
wt_05_329_032420237 Sm (Mn, 2x3)
Diode Array
254
Range: 3.186
Area, Height
Time Height Area Area%
0.57 26136 1161.53 0.69
0.70 3168579 158974.48 94.57
0.79 10189 404.64 0.24
1.23 5264 353.42 0.21
1.59 24222 2285.14 1.36
1.78 34124 4047.52 2.41
2.35 6273 882.76 0.53
22b
AU
Time

23a

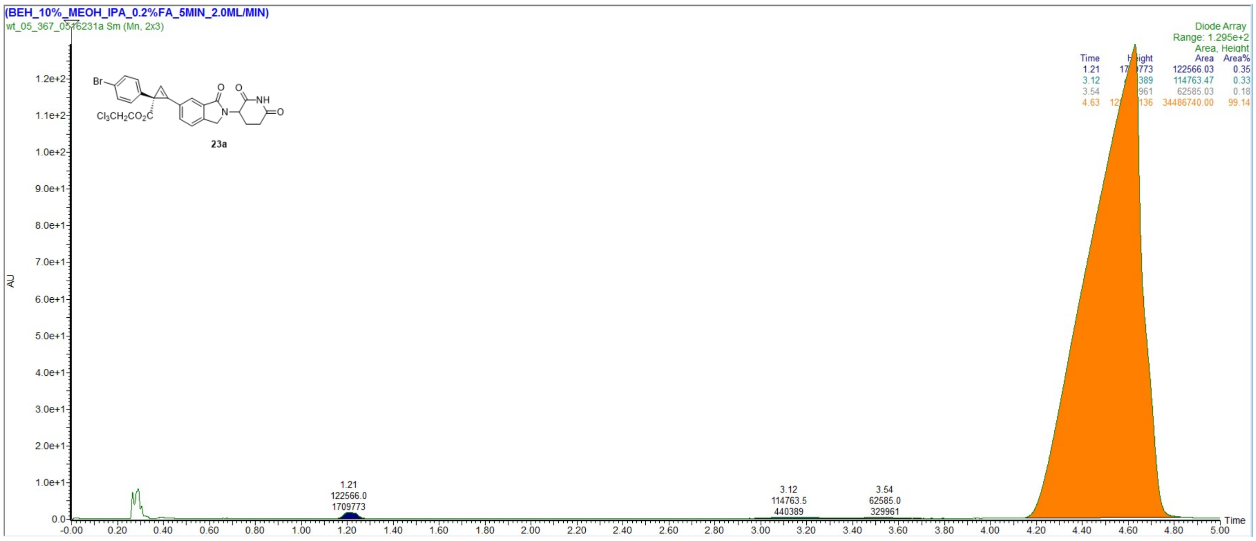
(BEH_10%_MEOH_IPA_0.2%FA_5MIN_2.0ML/MIN)
Diode Array
Range: 1.295e+2
Area, Height
Time Height Area Area%
1.21 122566.03 0.35
3.12 114763.47 0.33
3.54 62585.03 0.18
4.63 34486740.00 99.14
1.21
122566.0
1709773
3.12
114763.5
440389
3.54
62585.0
329961
23a
AU
Time

23b

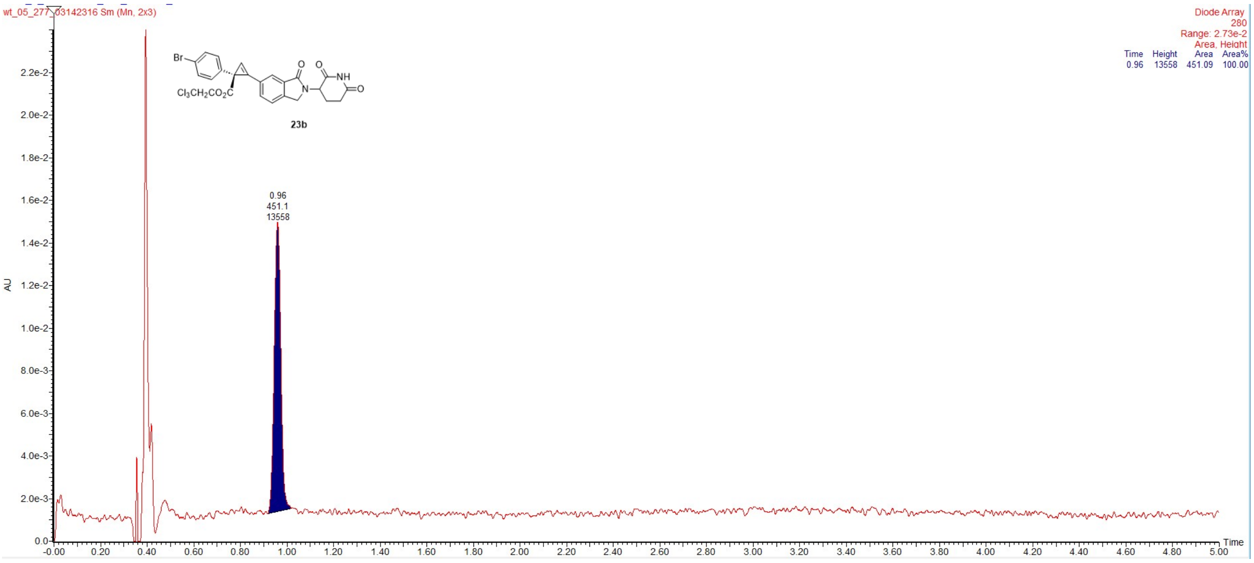
wt_05_277_03142316 Sm (Mn, 2x3)
Diode Array
280
Range: 2.73e-2
Area, Height
Time Height Area Area%
0.96 13558 451.09 100.00
23b
0.96
451.1
13558
AU
Time

24a

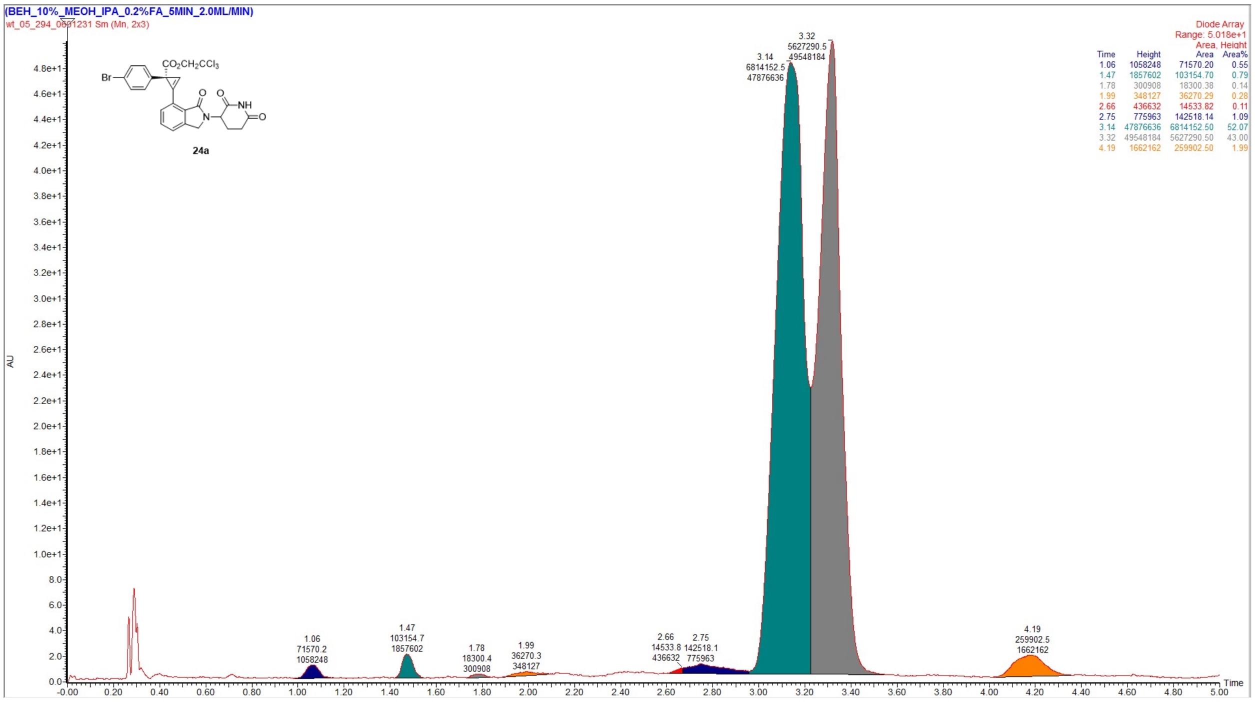
(BEH_10%_MEOH_IPA_0.2%FA_5MIN_2.0ML/MIN)
wt_05_294_0601231 Sm (Mn, 2x3)
Diode Array
Range: 5.018e+1
Area, Height
Time Height Area Area%
1.06 1058248 71570.20 0.55
1.47 1857602 103154.70 0.79
1.78 300908 18300.38 0.14
1.99 348127 36270.29 0.28
2.66 436632 14533.82 0.11
2.75 775963 142518.14 1.09
3.14 47876636 6814152.50 52.07
3.32 49548184 5627290.50 43.00
4.19 1662162 259902.50 1.99
24a
AU
Time

24b

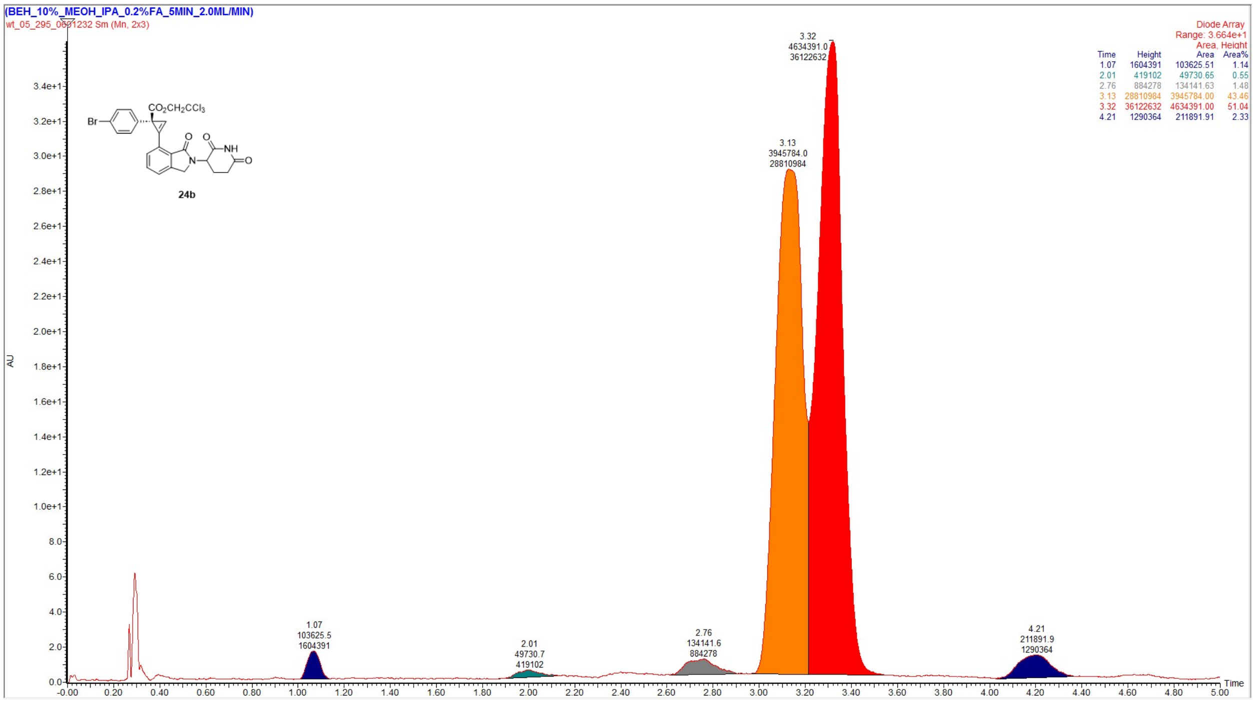

25a

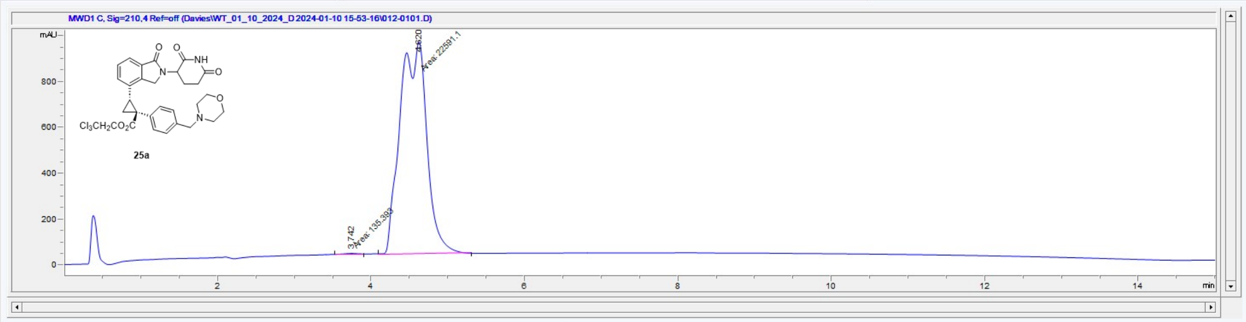

	File Information
LC-File	012-0101.D
File Path	C:\Chem32\1\Data\Davies\WT_01_10_2024_D 2024-01-10 15-53-1
Date	10-Jan-24, 15:55:00
Sample	WT-05-564
Sample Info	
Barcode	
Operator	SYSTEM
Method	hdcf104_purity.M
Reference	
Analysis Time	15 min

#	Time	Area	Height	Width	Area%	Symmetry
1	3.742	135.4	7.8	0.2904	0.596	1.136
2	4.62	22591.1	927	0.4062	99.404	2.017

25b

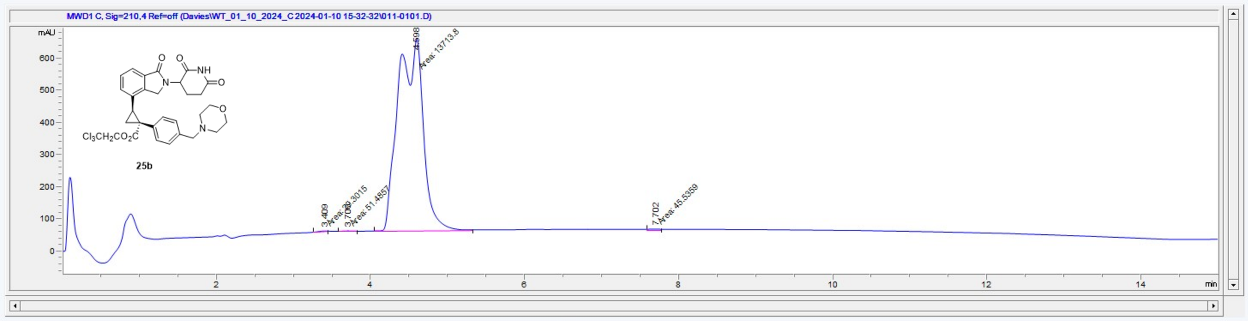

File Information

LC-File	011-0101.D
File Path	C:\Chem32\1\Data\Davies\WT_01_10_2024_C 2024-01-10 15-32-3
Date	10-Jan-24, 15:34:17
Sample	WT-05-550
Sample Info	
Barcode	
Operator	SYSTEM
Method	hdcf104_purity.M
Reference	
Analysis Time	14.993 min
[illegible]	[illegible]

#	Time	Area	Height	Width	Area%	Symmetry
1	3.409	29.3	2.3	0.2082	0.212	5.947
2	3.706	51.5	3.8	0.2259	0.372	0.919
3	4.598	13713.8	597.3	0.3827	99.087	2.343
4	7.702	45.5	3.8	0.1992	0.329	1.331

26a

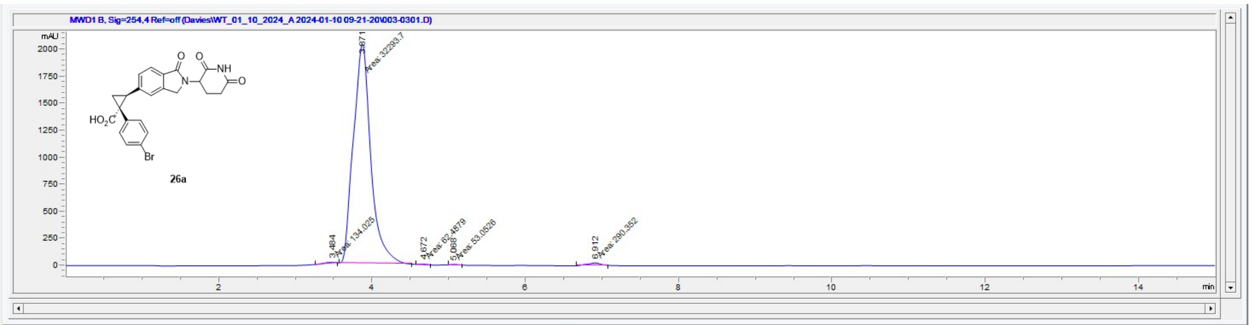

File Information

LC-File	003-0301.D
File Path	C:\Chem32\1\Data\Davies\WT_01_10_2024_A 2024-01-10 09-21-2
Date	10-Jan-24, 09:55:04
Sample	WT-05-538
Sample Info	
Barcode	
Operator	SYSTEM
Method	hdcf104_purity.M
Reference	
Analysis Time	14.993 min
[illegible]	[illegible]

#	Time	Area	Height	Width	Area%	Symmetry
1	3.484	134	11.2	0.1992	0.408	3.036
2	3.871	32293.7	2039.6	0.2639	98.356	0.988
3	4.672	62.5	7.7	0.1349	0.190	0.645
4	5.068	53.1	7.1	0.1237	0.162	0.864
5	6.912	290.4	24.4	0.1982	0.884	1.496

26b

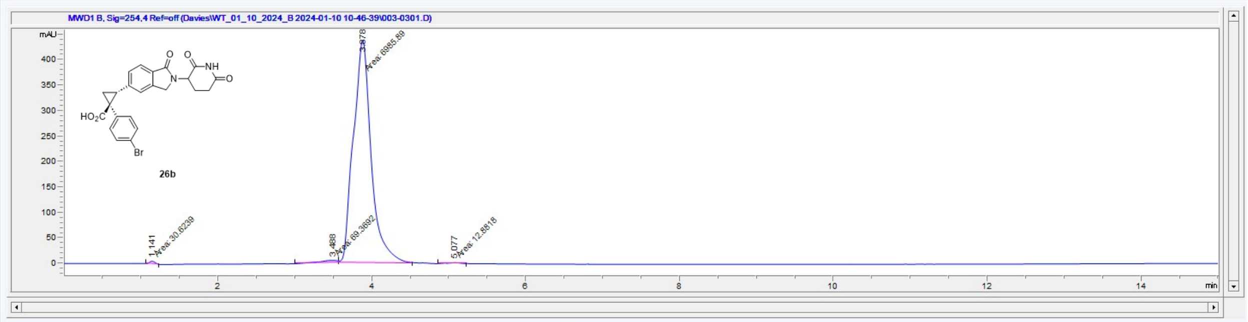

File Information

LC-File	003-0301.D
File Path	C:\Chem32\1\Data\Davies\WT_01_10_2024_B 2024-01-10 10-46-3
Date	10-Jan-24, 11:20:24
Sample	WT-05-539
Sample Info	
Barcode	
Operator	SYSTEM
Method	hdcf104_purity.M
Reference	
Analysis Time	15 min

#	Time	Area	Height	Width	Area%	Symmetry
1	1.141	30.6	5.7	0.0901	0.431	0.677
2	3.488	69.4	3.8	0.3047	0.977	3.234
3	3.878	6985.9	435	0.2677	98.410	1.015
4	5.077	12.9	1.1	0.1936	0.181	3.661

27a

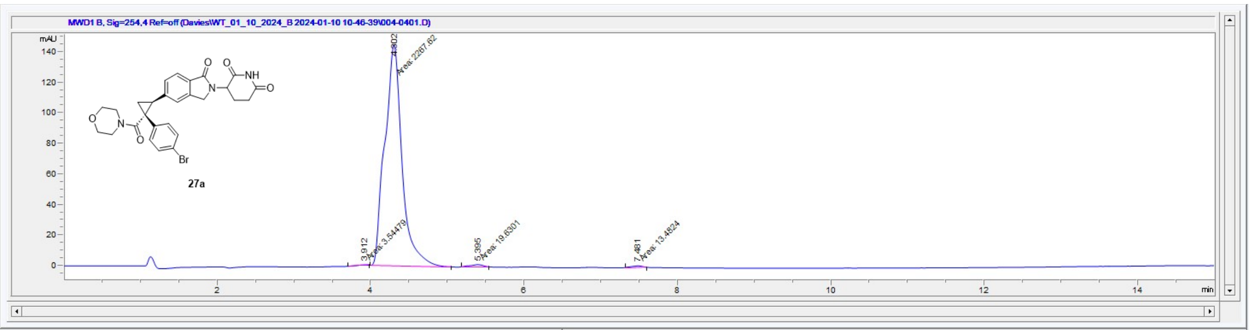

File Information

LC-File	004-0401.D
File Path	C:\Chem32\1\Data\Davies\WT_01_10_2024_B 2024-01-10 10-46-3
Date	10-Jan-24, 11:36:25
Sample	WT-05-546
Sample Info	
Barcode	
Operator	SYSTEM
Method	hdcf104_purity.M
Reference	
Analysis Time	14.993 min

#	Time	Area	Height	Width	Area%	Symmetry
1	3.912	3.5	4E-1	0.1495	0.154	2.991
2	4.302	2267.6	144.3	0.2618	98.409	1.057
3	5.395	19.6	1.6	0.2065	0.852	1.45
4	7.481	13.5	1.2	0.1889	0.585	1.71

27b

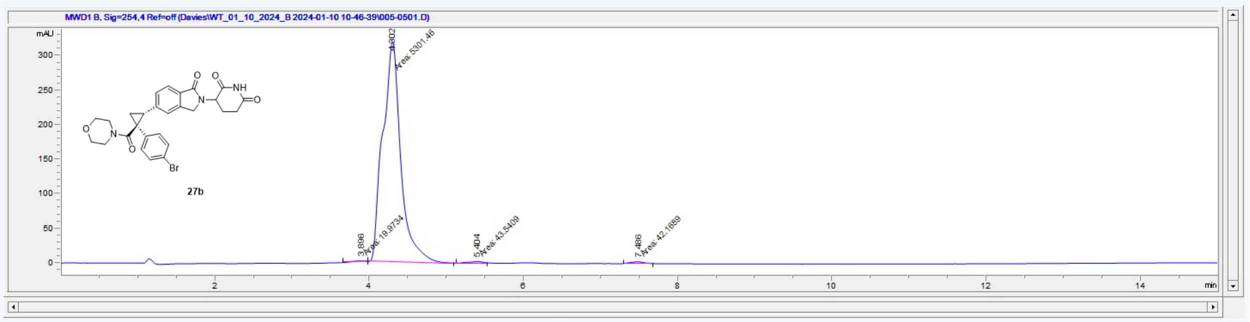

File Information

LC-File	005-0501.D
File Path	C:\Chem32\1\Data\Davies\WT_01_10_2024_B 2024-01-10 10-46-3
Date	10-Jan-24, 11:52:24
Sample	WT-05-547
Sample Info	
Barcode	
Operator	SYSTEM
Method	hdcf104_purity.M
Reference	
Analysis Time	14.993 min

#	Time	Area	Height	Width	Area%	Symmetry
1	3.896	20	1.6	0.2046	0.369	2.504
2	4.302	5301.5	322	0.2744	98.045	1.08
3	5.404	43.5	3	0.2443	0.805	1.771
4	7.486	42.2	3.2	0.2206	0.780	1.673

Section 6: Assay Protocols

Fluorescence Resonance Energy Transfer-Based NSD@ PWWP Cereblon Binding Assay.

A solution containing 0.5 nM purified 6×His-CRBN_005-DDB1_026 (CRBN a.a. 1−442, DDB1 a.a. 1−1140, generated in-house) and 20 nM Tracer compound (CC0782985) was premixed in FRET-assay buffer (50 mM HEPES pH 7.3, 50 mM NaCl, 0.005% Brij35, 1mg/mL BSA and 0.5mM TCEP). Compounds for testing were spotted into a 1536 well plate (Greiner Cat#782075). CRBN and Tracer solution were incubated in compound wells for 20 minutes. Detection mix containing Anti6xHis Tb Crytate (CisBio Cat#61HI2TLF) was then added to assay wells for a final 0.5x assay concentration of detection antibody (stock is provided at 400x). Plates were incubated for 30 minutes before being read on Pherastar FSX plate reader using TR-FRET module (Excitation 340nM, Emssion 615/665nm).

ePL and HiBiT degradation assays

DF15 cells overexpressing ePL tagged GSPT1 (DF15/GSPT1 ePL OE) was used to monitor the degradation of GSPT1 induced by experimental compounds. MDS-L cells overexpressing ePL tagged CK1α and GSPT1^{G575N} (MDS-L/CK1α ePL OE_ GSPT1^{G575N} OE) was used to monitor the degradation of CK1α induced by experimental compounds. NCCIT cells with HiBiT tag knocked in at the c-terminus of *SALL4* (NCCIT/SALL4-HiBiT KI) was used to monitor the degradation of SALL4 induced by experimental compounds. DF15 cells with HiBiT tag knocked in at the n-terminus of *IKZF3* (DF15/HiBiT-IKZF3 KI) was used to monitor the degradation of AIOLOS (IKZF3) induced by experimental compounds.

The cell culture medium recipes, seeding densities and compound incubation time with cells are shown in the table below:

Target	Cell line	Cell culture medium	Seeding density (cells/well)	Compound incubation time (hr)
GSPT1	DF15/GSPT1 ePL OE	RPMI 1640, 10% heat inactivated (HI) FBS, 1mM sodium pyruvate, 25mM Hepes, 0.1% pluronic acid, 1X NEAA	800	20
CK1α	MDS-L/CK1α ePL OE_ GSPT1^{G575N} OE	RPMI 1640, 20% HI FBS and 50 ng/mL of human recombinant IL3	1,000	4
SALL4	NCCIT/SALL4-HiBiT KI	RPMI 1640 and 10% HI FBS	800	2
IKZF3	DF15/HiBiT-IKZF3 KI	RPMI 1640, 10% HI FBS, 1mM sodium pyruvate, 25mM Hepes, 0.1% pluronic acid, 1X NEAA	800	4

For all degradation assays, compounds were pre-spotted in a 1536 well plate (Corning 3727) starting at 10uM with 3-fold serial dilution down 11 points in replicates using an Echo 650 series acoustic liquid handler. 5uL/well cells were seeded in the assay ready plate at the density and in the medium as indicated in the table. The final DMSO concentration in the cell culture was 0.25%. The plates were equilibrated at room temperature for 30 minutes before putting into a 37 °C CO_2 incubator. After appropriate incubation period, plates were retrieved from the incubator and set at room temperature to equilibrate for 30 minutes, before adding ePL or HiBiT detection reagent. For ePL detection, the pre-prepared mixture of EA reagent, lysis buffer, and substrate reagent at ratio 1:1:4 from the DiscoverX InCELL Detection Kit (Eurofins 96-0079L) according to the manufacturer's recommendation was added 3ul/well into the plate. Plates were incubated in the dark for 1hr at room temperature before reading using a BMG PheraStar luminescence reader. For HiBiT detection, Nano-Glo HiBiT Lytic Reagent (Promega, N3050) prepared according to the manufacturer's recommendation was added 3 uL/well into the plate. Plates were incubated 30 minutes at room temperature in the dark before reading using a BMG PheraStar luminescence reader.

To determine EC50 values for degradation, a four parameter logistic model (Sigmoidal Dose-Response Model) (FIT= (A+((B-A)/1+((C/x)^D)))) C is the inflection point (EC50), D is the correlation coefficient , A and B are the low and high limits of the fit respectively) was used to determine the compound's EC50 value, which is the half maximum effective concentration. In the degradation assays, the Yconst of each compound was calculated by normalizing the lowest point of the fitted curve to the media only control, which is 0%, and the cells treated with DMSO control, which is 100%. All degradation curves were processed and evaluated using Dotmatics.

Section 7: Biological Data Tables

Table S7.1: Fluorescence Resonance Energy Transfer-Based Cereblon Binding Assay Data

Compound Number	CRBN HTRF IC_{50} (μM)
**13a**	0.66
**13b**	2.4
**14a**	>10
**14b**	7.9
**15a**	0.39
**15b**	1.3
**16a**	5.3
**16b**	>10
**17a**	2.1
**17b**	2.5
**18a**	>10
**18b**	>10
**19a**	0.16
**19b**	0.35
**20a**	4.6
**20b**	35
**21a**	0.59
**21b**	0.8
**22a**	5.7
**22b**	7.8
**23a**	0.98
**23b**	1.7
**24a**	2.4
**24b**	2.9

Table S7.2: Neosubstrate Degradation Assay Data*

Compound Number	IKZF3 EC_{50} (μM)	IKZF3 Y_{min} (%)	CK1a EC_{50} (μM)	CK1a Y_{min} (%)	GSPT1 EC_{50} (μM)	GSPT1 Y_{min} (%)	SALL4 EC_{50} (μM)	SALL4 Y_{min} (%)
**13a**	>10 ± 0.00	81 ± 3.3	>10 ± 0.00	87 ± 0.55	>10 ± 0.00	92 ± 7.8	>10 ± 0.00	85 ± 7.3
**13b**	>10 ± 0.00	97 ± 4.9	>10 ± 0.00	90 ± 7.1	>10 ± 0.00	99 ± 2.2	>10 ± 0.00	97 ± 3.1
**14a**	>10 ± 0.00	89 ± 3.2	>10 ± 0.00	92 ± 3.8	>10 ± 0.00	91 ± 16	>10 ± 0.00	91 ± 8.0
**14b**	>10 ± 0.00	95 ± 4.3	>10 ± 0.00	94 ± 5.1	>10 ± 0.00	87 ± 11	>10 ± 0.00	87 ± 13
**15a**	>10 ± 0.00	100 ± 4.6	>10 ± 0.00	89 ± 5.1	>10 ± 0.00	96 ± 7.8	>10 ± 0.00	99 ± 1.2
**15b**	>10 ± 0.00	89 ± 5.0	0.22 ± 0.089	47 ± 5.0	>10 ± 0.00	83 ± 7.0	0.16 ± 0.05	67 ± 5.4
**16a**	>10 ± 0.00	94 ± 8.3	>10 ± 0.00	94 ± 9.9	>10 ± 0.00	94 ± 11	>10 ± 0.00	92 ± 8.2
**16b**	>10 ± 0.00	97 ± 3.6	>10 ± 0.00	85 ± 4.1	>10 ± 0.00	86 ± 8.2	0.41 ± 0.16	60 ± 6.1
**17a**	>10 ± 0.00	95 ± 7.6	>10 ± 0.00	98 ± 12	>10 ± 0.00	95 ± 5.4	>10 ± 0.00	97 ± 2.2
**17b**	>10 ± 0.00	89 ± 6.5	>10 ± 0.00	96 ± 12	>10 ± 0.00	93 ± 5.8	>10 ± 0.00	96 ± 3.1
**18a**	>10 ± 0.00	91 ± 0.6	>10 ± 0.00	89 ± 1.7	>10 ± 0.00	87 ± 6.5	>10 ± 0.00	93 ± 3.8

**18b**	>10 ± 0.00	96 ± 5.7	>10 ± 0.00	91 ± 7.1	>10 ± 0.00	83 ± 15	>10 ± 0.00	96 ± 5.4
**19a**	0.012 ± 0.010	7.9 ± 1.8	0.17 ± 0.18	64 ± 4.3	0.17 ± 0.13	64 ± 8.7	0.019 ± 0.010	23 ± 4.5
**19b**	0.11 ± 0.05	69 ± 3.3	1.5 ± 1.1	60 ± 7.4	0.55 ± 0.47	66 ± 5.5	0.17 ± 0.06	35 ± 5.9
**20a**	0.90 ± 0.18	40 ± 2.2	1.5 ± 0.94	56 ± 14	4.3 ± 1.9	12 ± 13	0.56 ± 0.27	34 ± 7.4
**20b**	3.7 ± 0.62	10 ± 8.2	2.1 ± 1.3	54 ± 8.9	5.1 ± 1.4	17 ± 13	2.8 ± 1.6	57 ±7.7
**21a**	>10 ± 0.00	95 ± 7.6	>10 ± 0.00	89 ± 9.3	>10 ± 0.00	81 ± 17	>10 ± 0.00	89 ± 4.2
**21b**	4.8 ± 2.6	79 ± 6.4	0.25 ± 0.37	57 ± 8.0	3.3 ± 3.0	7.7 ± 4.7	0.075 ± 0.050	68 ± 2.1
**22a**	1.3 ± 2.1	73 ± 6.2	1.2 ± 1.2	60 ± 26	1.1 ± 0.69	2.7 ± 0.7	3.6 ± 0.74	67 ± 7.8
**22b**	0.31 ± 0.11	71 ± 9.7	1.6 ± 0.71	55 ± 39	1.4 ± 0.88	1.8 ± 0.86	2.4 ± 1.7	58 ± 3.2
**23a**	>10 ± 0.00	94 ± 2.3	>10 ± 0.00	93 ± 10	>10 ± 0.00	82 ± 7.1	>10 ± 0.00	95 ± 6.5
**23b**	>10 ± 0.00	81 ± 4.6	>10 ± 0.00	95 ± 6.0	>10 ± 0.00	88 ± 10	>10 ± 0.00	91 ± 10
**24a**	>10 ± 0.00	97 ± 0.58	>10 ± 0.00	96 ± 12	>10 ± 0.00	85 ± 24	>10 ± 0.00	95 ± 5.0
**24b**	>10 ± 0.00	93 ± 2.5	>10 ± 0.00	93 ± 6.8	>10 ± 0.00	80 ± 4.3	>10 ± 0.00	97 ± 5.2

* N ≥ 3 for all data

Section 8: References

1. Qin, C.; Davies, H. M. L., Role of Sterically Demanding Chiral Dirhodium Catalysts in Site-Selective C–H Functionalization of Activated Primary C–H Bonds. *Journal of the American Chemical Society* **2014,** *136*, 9792-9796.
2. Garlets, Z. J.; Boni, Y. T.; Sharland, J. C.; Kirby, R. P.; Fu, J.; Bacsa, J.; Davies, H. M. L., Design, Synthesis, and Evaluation of Extended C4–Symmetric Dirhodium Tetracarboxylate Catalysts. *ACS Catalysis* **2022,** *12*, 10841-10848.
3. Tracy, W. F.; Davies, G. H. M.; Grant, L. N.; Ganley, J. M.; Moreno, J.; Cherney, E. C.; Davies, H. M. L., Anhydrous and Stereoretentive Fluoride-Enhanced Suzuki–Miyaura Coupling of Immunomodulatory Imide Drug Derivatives. *The Journal of Organic Chemistry* **2024**.
4. Berlin, M.; Dong, H.; Sherman, D.; Snyder, L.; Wang, J.; Zhang, W. Bifunctional Molecules Containing an E3 Ubiquitin Ligase Binding Moiety Linked to a BCL6 Targeting Moiety. US 2022323547A1
2021.
5. Tortoreto, C.; Rackl, D.; Davies, H. M. L., Metal-Free C–H Functionalization of Alkanes by Aryldiazoacetates. *Organic Letters* **2017,** *19*, 770-773.
6. Tilden, J. A. R.; Lubben, A. T.; Reeksting, S. B.; Kociok-Köhn, G.; Frost, C. G., Pd(II)-Mediated C−H Activation for Cysteine Bioconjugation. *Chemistry – A European Journal* **2022,** *28*, e202104385.
